# Supplementary material for: Comprehensive Species Sampling and Sophisticated Algorithmic Approaches Refute the Monophyly of Arachnida
Source: Mol Biol Evol. 2022 Feb 8;39(2):msac021. doi: 10.1093/molbev/msac021 (PMC8845124; doi:10.1093/molbev/msac021)
Supplement: msac021_Supplementary_Data [file msac021_supplementary_data.pdf]

**Supplementary Material**

# Comprehensive species sampling and sophisticated algorithmic approaches refute the monophyly of Arachnida

Jesús A. Ballesteros[1*], Carlos E. Santibáñez-López[1,2], Caitlin M. Baker[1,3], Ligia R. Benavides[3], Tauana J. Cunha[3,4], Guilherme Gainett[1], Andrew Z. Ontano[1], Emily V.W. Setton[1], Claudia P. Arango[5], Efrat Gavish-Regev[6], Mark S. Harvey[7,8], Ward C. Wheeler[9], Gustavo Hormiga[10], Gonzalo Giribet[3], Prashant P. Sharma[1*]

[1]Department of Integrative Biology; University of Wisconsin-Madison, Madison, WI, USA 53706

[2]Department of Biology, Western Connecticut State University; Danbury, CT, USA, 06810

[3]Department of Organismic and Evolutionary Biology, Museum of Comparative Zoology, Harvard University; Cambridge, MA, USA, 02138

[4]Smithsonian Tropical Research Institute, Panama City, Panama

[5]Office for Research, Griffith University; Nathan, Queensland 4111, Australia

[6]National Natural History Collections, The Hebrew University of Jerusalem; Jerusalem, Israel 9190401

[7]Collections & Research, Western Australian Museum; Welshpool, Western Australia 6106, Australia

[8]School of Biological Sciences, University of Western Australia; Crawley, Western Australia 6009, Australia

[9]Division of Invertebrate Zoology, American Museum of Natural History; New York, NY, USA, 10024

[10]Department of Biological Sciences, George Washington University; Washington, DC, USA, 20052

*Correspondence: jeballes@kean.edu (JAB); prashant.sharma@wisc.edu (PPS)

**Fig. S1.** Summary of *a posteriori* trees from Phylobayes-mpi analysis of Matrix 4 under the CAT-GTR model. Left column: Individual summary trees from chains 1-4. Top right: Summary tree from combining chains 1 and 2 (starting trees using unconstrained ML analysis of Matrix 4). Bottom right: Summary tree from combining chains 3 and 4 (starting trees using ML analysis of Matrix 4, with arachnid monophyly constrained).

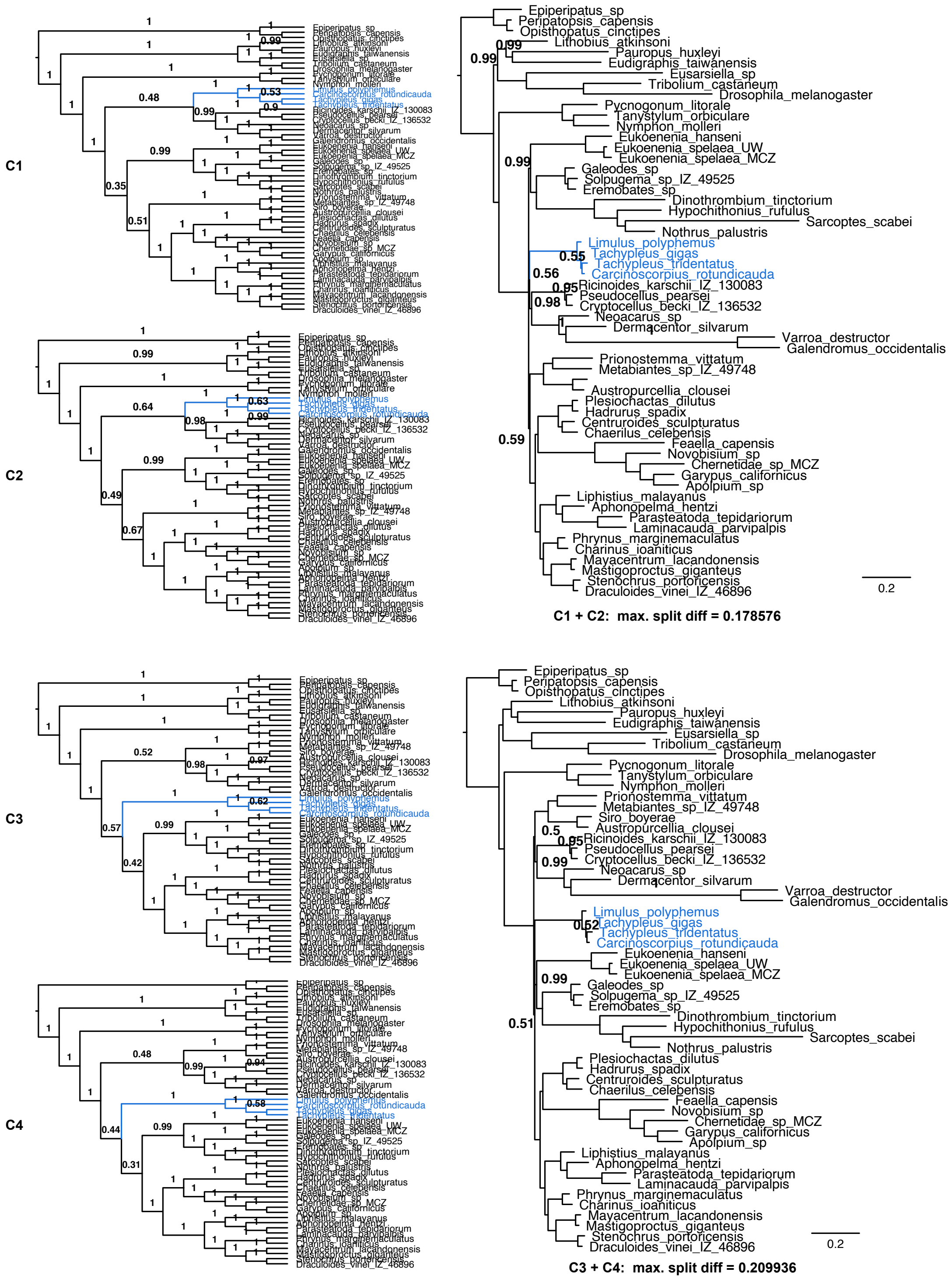

**Fig. S2.** Summary of *a posteriori* trees from Phylobayes-mpi analysis of Matrix 4 under the CAT-GTR model. Left column: Individual summary trees from chains 5-8. Right: Summary tree from combining chains 5-8 (random starting trees).

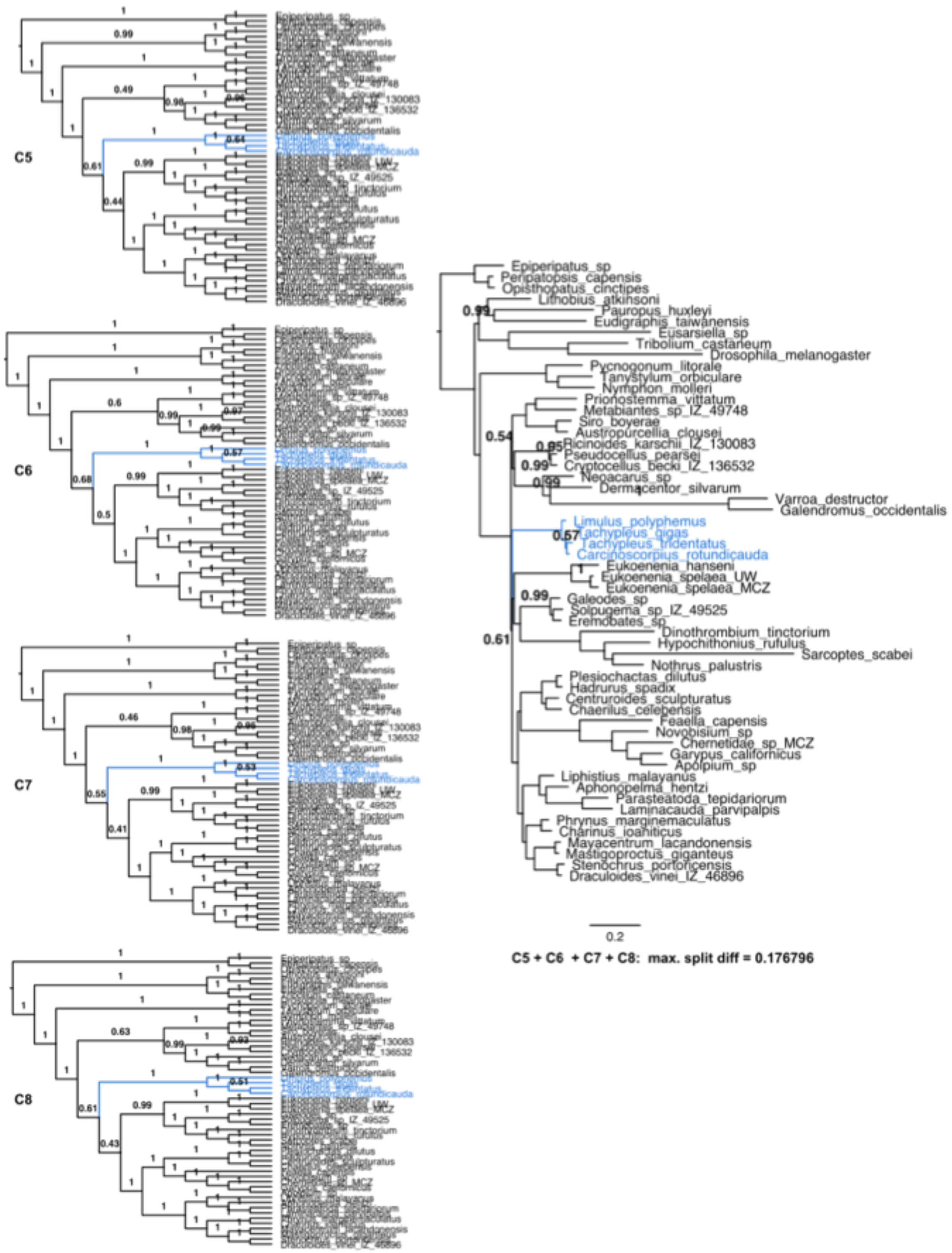

**Fig. S3.** ΔGLS distributions mapping phylogenetic support for the unconstrained tree topology versus trees constrained to recover Dromopoda (Scorpiones + Pseudoscorpiones + Opiliones + Solifugae), for Matrices 1 and 3. A minority of genes (34-36%) support monophyly of Dromopoda, which has been refuted by phylogenomics and rare genomic changes.

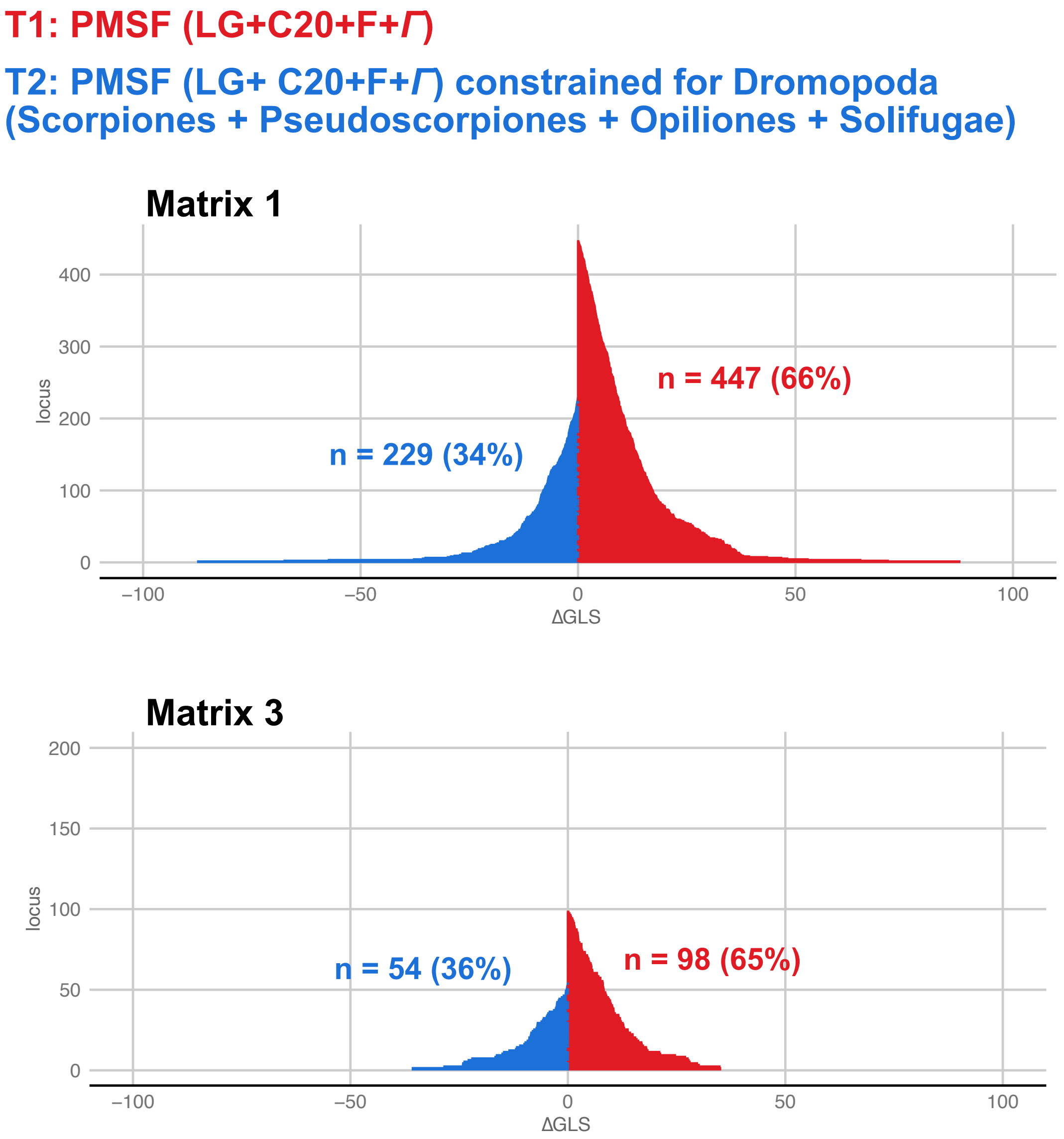

**Fig. S4.** Distributions of Shannon entropy for ΔSLS categories supporting unconstrained topologies (red) versus constrained topologies (blue). Left: Shannon entropy distributions for Dromopoda. Right: Shannon entropy distributions for Arachnida. Note that there are fewer sites supporting Arachnida than Dromopoda, with the latter grouping refuted by phylogenomics and rare genomic changes.

**Distribution of site Shannon entropy sites H > 0**
**between ΔSLS classes contrasting alternative topologies**

**T1: Matrix 1 PMSF (LG+C20+F+Γ) unconstrained**

**T2: Matrix 1 PMSF (LG+C20+F+Γ) constrained on Dromopoda (Scorpiones + Pseudoscorpiones + Opiliones + Solifugae)**

**T1: Matrix 1 PMSF (LG+C20+F+Γ) unconstrained**

**T2: Matrix 1 PMSF (LG+C20+F+Γ) constrained on Arachnida**

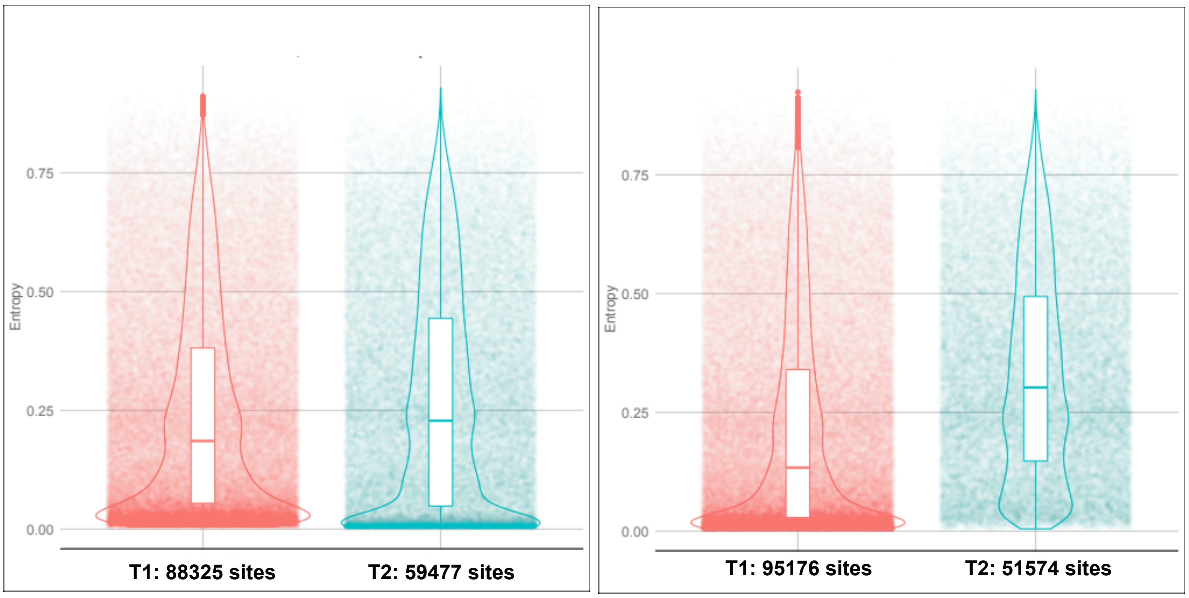

**Fig. S5.** ΔGLS distributions mapping phylogenetic support for the competing hypotheses of pseudoscorpion placement, for Matrices 1 and **3**. Genes filtered only for taxon decisiveness (Matrix 1) have uninformative distributions of gene support for competing topologies. Genes further filtered to retain the least saturated subset (Matrix **3**) show greater support for Panscorpiones (Pseudoscorpiones + Scorpiones), a group that has been validated by rare genomic changes.

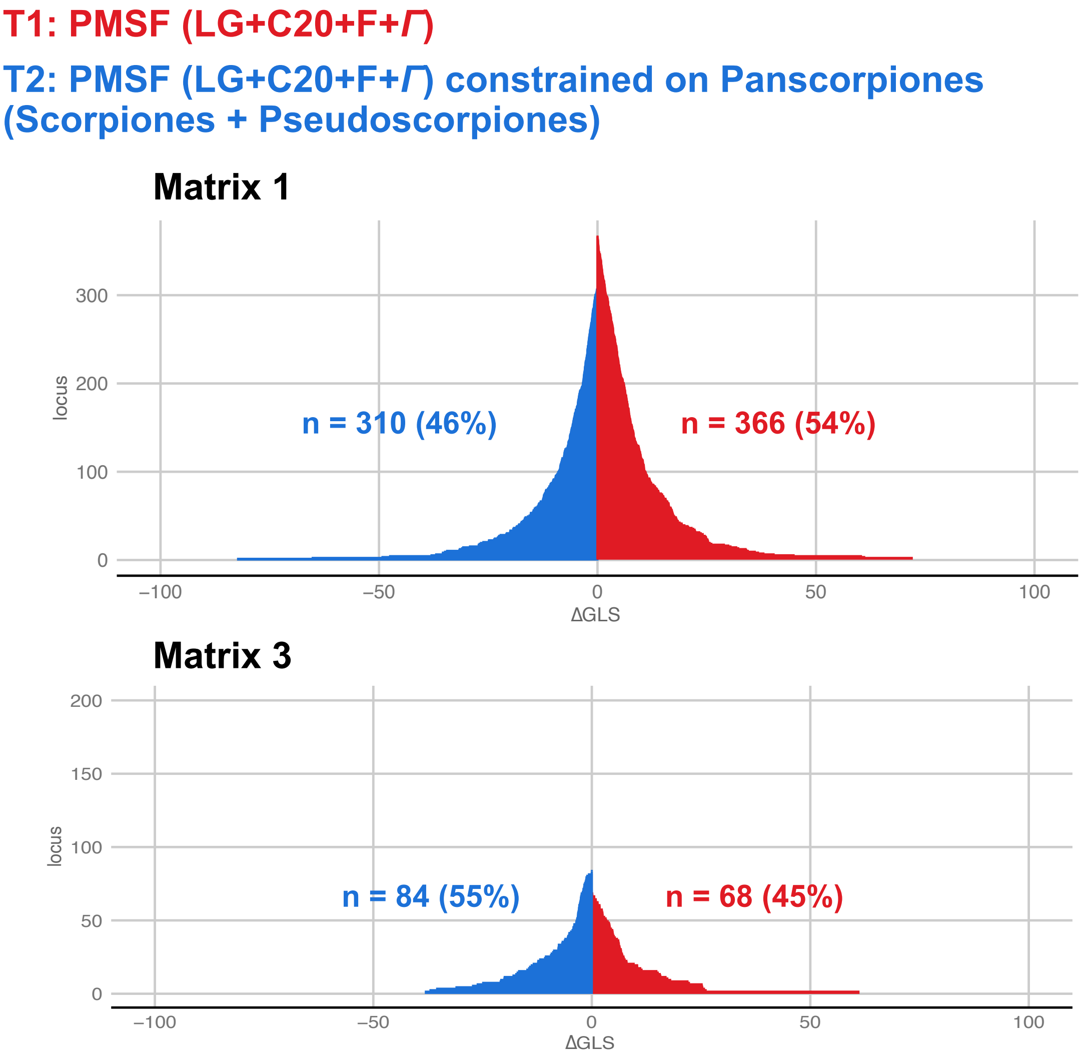

**Fig. S6.** Integration of morphological and molecular datasets refutes arachnid monophyly. (*a*) Strict consensus tree from equal weights parsimony analysis of Bicknell et al. dataset, combined with Matrix 3. (*b*) Implied weights parsimony analysis of Bicknell et al. dataset, combined with Matrix 3. Concavity constant *k* = 6. (*c*) Strict consensus tree from equal weights parsimony analysis of Siveter et al. dataset, combined with Matrix 3. (*d*) Implied weights parsimony analysis of Siveter et al. dataset, combined with Matrix 3. Concavity constant *k* = 6. Colored branches correspond to Pycnogonida (red), Merostomata (blue), and Arachnida (green). Tree files for other *k* values explored in implied weights framework for both datasets are provided on FigShare.

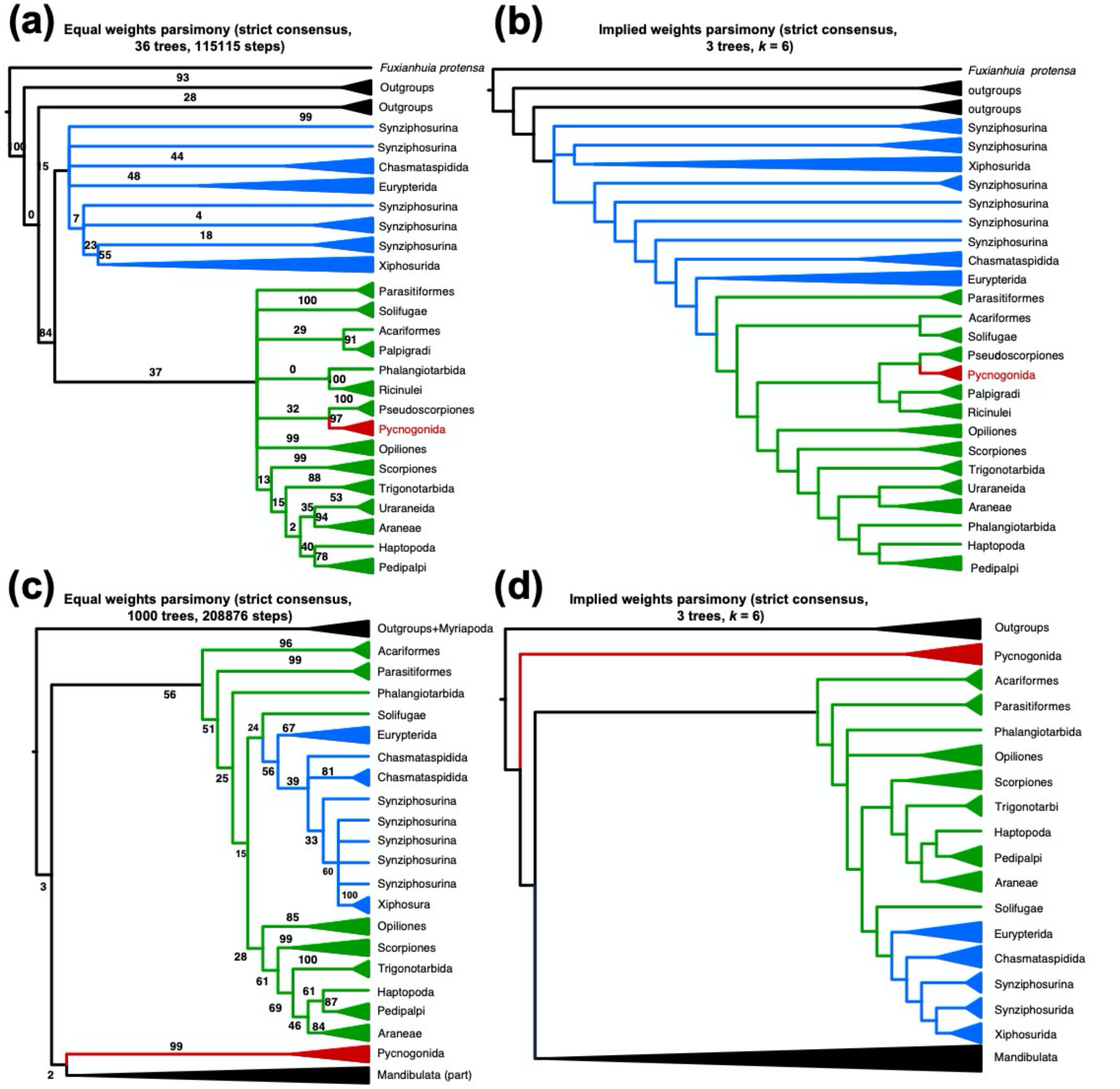

**Table S1.** Species sampling and accession data.

| Terminal | Taxonomy | Accession number | Sampled for Matrix 4 |
|---|---|---|---|
| Epiperipatus sp. MCZ:IZ-141126 | Onychophora | SRR8320992 | X |
| Opisthopatus kwazululandi MCZ:IZ-131434 | Onychophora | SRR8318947 | X |
| Peripatopsis overbergiensis MCZ:IZ-131372 | Onychophora | SRR1145776 | X |
| Eudigraphis taiwaniensis MCZ:IZ-128912 | Myriapoda: Diplopoda | SRR3458640 | X |
| Narceus americanus MCZ:IZ-44069 | Myriapoda: Diplopoda | SRR3233222 | |
| Pauropus huxleyi MCZ:IZ-141222 | Myriapoda: Pauropoda | SRR6145369 | X |
| Symphylella sp. MCZ:IZ-141598 | Myriapoda: Symphyla | SRR6144316 | |
| Scutigera coleoptrata MCZ:IZ-20415 | Myriapoda: Chilopoda | SRR1158078 | |
| Craterostigmus crabilli MCZ:IZ-71256 | Myriapoda: Chilopoda | SRR3232915 | |
| Lithobius atkinsoni | Myriapoda: Chilopoda | SRR7879352 | X |
| Alipes grandidieri MCZ:IZ-130616 | Myriapoda: Chilopoda | SRR619311 | |
| Strigamia maritima | Myriapoda: Chilopoda | GCA_000239455.1 | |
| Eusarsiella sp. | Pancrustacea: Ostracoda | SRR4113497 | X |
| Daphnia magna | Pancrustacea: Branchiopoda | GCA_003990815.1 | |
| Daphnia pulex | Pancrustacea: Branchiopoda | GCA_900092285.2 | |
| Eurytemora affinis | Pancrustacea: Copepoda | GCF000591075.2 | |
| Hyalella azteca | Pancrustacea: Malacostraca | GCA_000764305.3 | |
| Folsomia candida | Pancrustacea: Hexapoda | GCA_002217175.1 | |
| Orchesella cincta | Pancrustacea: Hexapoda | GCA_001718145.1 | |
| Gryllus bimaculatus | Pancrustacea: Hexapoda | SAMN23440570 | |
| Zootermopsis nevadensis | Pancrustacea: Hexapoda | GCF_000696155.1 | |
| Nasonia vitripennis | Pancrustacea: Hexapoda | GCF_009193385.2 | |
| Drosophila melanogaster | Pancrustacea: Hexapoda | GCF_000001215.4 | X |
| Tribolium castaneum | Pancrustacea: Hexapoda | GCA_000002335.3 | X |
| Palaeopantopus | Chelicerata: Pycnogonida | fossil | |
| Palaeoisopus | Chelicerata: Pycnogonida | fossil | |
| Flagellopantopus | Chelicerata: Pycnogonida | fossil | |
| Haliestes dasos | Chelicerata: Pycnogonida | fossil | |
| Tanystylum orbiculare | Chelicerata: Pycnogonida | SRR13153832 | X |
| Nymphon molleri | Chelicerata: Pycnogonida | SRR13153829 | X |
| Anoplodactylus insignis | Chelicerata: Pycnogonida | SRR5237777 | |
| Phoxichilidium femoratum | Chelicerata: Pycnogonida | SRR13153828 | |
| Pycnogonum litorale | Chelicerata: Pycnogonida | SRR7879353 | X |
| Pallenella flava | Chelicerata: Pycnogonida | SRR13153831 | |
| Stylopallene cheilorhynchus | Chelicerata: Pycnogonida | SRR13153830 | |
| Dibasterium | Chelicerata: Synziphosurina | fossil | |
| Offacolus | Chelicerata: Synziphosurina | fossil | |
| Weinbergina | Chelicerata: Synziphosurina | fossil | |
| Limulus polyphemus | Chelicerata: Xiphosura | GCA_000517525.1 | X |
| Carcinoscorpius rotundicauda | Chelicerata: Xiphosura | GCA_011833715.1 | X |
| Tachypleus tridentatus | Chelicerata: Xiphosura | GCA_004210375.1 | X |
| Tachypleus gigas | Chelicerata: Xiphosura | GCA_014155125.1 I | X |
| Parastylonurus | Chelicerata: Eurypterida | fossil | |
| Mixopterus | Chelicerata: Eurypterida | fossil | |
| Eurypterus | Chelicerata: Eurypterida | fossil | |
| Chasmataspis | Chelicerata: Chasmataspidida | fossil | |
| Octoberaspis | Chelicerata: Chasmataspidida | fossil | |
| Eukoenenia hanseni MCZ:IZ-74291 | Chelicerata: Palpigradi | SAMN23438329 | X |
| Eukoenenia spelaea MCZ:IZ-60890 | Chelicerata: Palpigradi | SAMN23438330 | X |
| Eukoenenia spelaea | Chelicerata: Palpigradi | SRR8080647 | X |
| Galeodes sp. | Chelicerata: Solifugae | SRR8080645 | X |
| Solpugema sp. MCZ:IZ-49525 | Chelicerata: Solifugae | SRR9332019 | X |
| Eremobates sp. MCZ:IZ-49755 | Chelicerata: Solifugae | SRR1146672 | |

| | | | |
|---|---|---|---|
| Eremobates sp. | Chelicerata: Solifugae | SAMN23440569 | X |
| Adenacarus sp. | Chelicerata: Parasitiformes | SRR8080646 | |
| Neocarus sp. | Chelicerata: Parasitiformes | SAMN23438331 | X |
| Ricinoides atewa MCZ:IZ-130073 | Chelicerata: Ricinulei | SRR1145743 | |
| Ricinoides karschii MCZ:IZ-130083 | Chelicerata: Ricinulei | SRR1972991 | X |
| Pseudocellus pearsei MCZ:IZ-16426 | Chelicerata: Ricinulei | SRR1146686 | X |
| Pseudocellus sp. MCZ:IZ-140060 | Chelicerata: Ricinulei | SRR13590369 | |
| Cryptocellus sp. MCZ:IZ-143922 | Chelicerata: Ricinulei | SRR13590368 | |
| Cryptocellus becki MCZ:IZ-136532 | Chelicerata: Ricinulei | SRR1979416 | X |
| Cryptocellus sp. MCZ:IZ-30913 | Chelicerata: Ricinulei | SRR1982218 | |
| Galendromus occidentalis | Chelicerata: Parasitiformes | GCA_000255335.1 | X |
| Gromphadorholaelaps schaeferi MCZ:IZ-71202 | Chelicerata: Parasitiformes | SAMN23438332 | |
| Varroa destructor | Chelicerata: Parasitiformes | SRR3927486 | X |
| Pneumolaelaps niutirani | Chelicerata: Parasitiformes | SRR10269756 | |
| Tropilaelaps mercedesae | Chelicerata: Parasitiformes | GCA_002081605.1 | |
| Orthinodoros rostratus | Chelicerata: Parasitiformes | SRR1732011 | |
| Ixodes scapularis | Chelicerata: Parasitiformes | GCA_000208615.1 | |
| Haemaphysalis longicornis | Chelicerata: Parasitiformes | SRR7754709 | |
| Amblyomma americanum | Chelicerata: Parasitiformes | SRR4416251 SRR4416250 | |
| Dermacentor silvarum | Chelicerata: Parasitiformes | GCA_013339745.1 | X |
| Hyalomma excavatum | Chelicerata: Parasitiformes | SRR3157672 | |
| Rhipicephalus microplus | Chelicerata: Parasitiformes | SRR1186998 | |
| Labidostommatidae gen. sp. MCZ:IZ-67347 | Chelicerata: Acariformes | SAMN23438333 | |
| Dinothrombium tinctorium | Chelicerata: Acariformes | GCA_003675995.1 | X |
| Panonychus citri | Chelicerata: Acariformes | SRR341928 | |
| Tetranychus cinnabarinus | Chelicerata: Acariformes | SRR519097 | |
| Tetranychus urticae | Chelicerata: Acariformes | GCA_000239435.1 | |
| Rhizoglyphus robini | Chelicerata: Acariformes | SRR946953 | |
| Sarcoptes scabiei | Chelicerata: Acariformes | GCA_014595675.1 | X |
| Dermatophagoides farinae | Chelicerata: Acariformes | SRR1016494 | |
| Dermatophagoides pteronyssinus | Chelicerata: Acariformes | GCA_001901225.2 | |
| Euroglyphus maynei | Chelicerata: Acariformes | GCA_002135145.1 | |
| Hypochthonius rufulus | Chelicerata: Acariformes | SRR4039020 | X |
| Steganacarus magnus | Chelicerata: Acariformes | SRR4039023 | |
| Achipteria coleoptrata | Chelicerata: Acariformes | SRR4039018 | |
| Alaskozetes antarcticus | Chelicerata: Acariformes | SRR6451453, SRR6451455, SRR6451457 | |
| Hermannia gibba | Chelicerata: Acariformes | SRR4039019 | |
| Archegozetes longisetosus | Chelicerata: Acariformes | SAMN23438334 | |
| Nothrus palustris | Chelicerata: Acariformes | SRR4039021 | X |
| Platynothrus peltifer | Chelicerata: Acariformes | SRR4039022 | |
| Idmonarachne | Chelicerata: Uraraneida | fossil | |
| Plesiosiro | Chelicerata: Haptopoda | fossil | |
| Eophrynus | Chelicerata: Trigonotarbida | fossil | |
| Palaeocharinus | Chelicerata: Trigonotarbida | fossil | |
| Anthracomartus | Chelicerata: Trigonotarbida | fossil | |
| Mastigoproctus giganteus | Chelicerata: Uropygi | SAMN23440568 | X |
| Mayacentrum lacandonensis MCZ:IZ-71253 | Chelicerata: Uropygi | SRR9332012 | X |
| Draculoides vinei MCZ:IZ-46896 | Chelicerata: Schizomida | SAMN23438335 | X |
| Stenochrus portoricensis | Chelicerata: Schizomida | SRR6997625 | X |
| Stenochrus sp. MCZ:IZ-74289 | Chelicerata: Schizomida | SAMN23438336 | |
| Graeophonus | Chelicerata: Amblypygi | fossil | |
| Paracharonopsis | Chelicerata: Amblypygi | fossil | |
| Kronocharon | Chelicerata: Amblypygi | fossil | |
| Sarax ioanniticus | Chelicerata: Amblypygi | SRR12389147-148 | X |
| Sarax israelensis | Chelicerata: Amblypygi | SRR12389135-140 | |
| Damon variegatus MCZ:IZ-29740 | Chelicerata: Amblypygi | SRR1145694 | |
| Acanthophrynus coronatus MCZ:IZ-71252 | Chelicerata: Amblypygi | SAMN23438337 | |
| Phrynus marginemaculatus | Chelicerata: Amblypygi | SRR12232018 | X |

| | | | |
|---|---|---|---|
| Attercopus fimbriunguis | Chelicerata: Uraraneida | fossil | |
| Permarachne novokshonovi | Chelicerata: Uraraneida | fossil | |
| Chimerarachne yingi | Chelicerata: Uraraneida | fossil | |
| Liphistius malayanus | Chelicerata: Araneae | SRR1145736 | X |
| Liphistius sp. | Chelicerata: Araneae | SRR1514873 | |
| Sphodros rufipes | Chelicerata: Araneae | SRR1514908 | |
| Megahexura fulva | Chelicerata: Araneae | SRR1514891 | |
| Aliatypus coylei | Chelicerata: Araneae | SRR1514876 | |
| Antrodiaetus unicolor | Chelicerata: Araneae | SRR1514897 | |
| Porrhothele sp. | Chelicerata: Araneae | SRR6997604 | |
| Microhexura montivaga | Chelicerata: Araneae | SRR1514890 | |
| Macrothele calpeiana | Chelicerata: Araneae | SRR6994009 | |
| Paratropis sp. | Chelicerata: Araneae | SRR1514893 | |
| Cyclocosmia truncata | Chelicerata: Araneae | SRR1514884 | |
| Hebestatis theveneti | Chelicerata: Araneae | SRR1514887 | |
| Idiops bersebaensis | Chelicerata: Araneae | SRR1514907 | |
| Aptostichus stephencolberti | Chelicerata: Araneae | SRR1514874 | |
| Promyrmekiaphila clathrata | Chelicerata: Araneae | SRR1514896 | |
| Brachythele longitarsis | Chelicerata: Araneae | SRR1514875 | |
| Pionothele sp. | Chelicerata: Araneae | SRR1514906 | |
| Stanwellia sp. | Chelicerata: Araneae | SRR6997603 | |
| Damarchus sp. | Chelicerata: Araneae | SRR3144092 | |
| Trichopelma laselva | Chelicerata: Araneae | SRR1514881 | |
| Acanthoscurria geniculata | Chelicerata: Araneae | GCA_000661875.1 | |
| Aphonopelma hentzi | Chelicerata: Araneae | SRX10000054, SRX10000055 | X |
| Aphonopelma iviei | Chelicerata: Araneae | SRR1514871 | |
| Filistata insidiatrix | Chelicerata: Araneae | SRR6997865 | |
| Kukulcania hibernalis | Chelicerata: Araneae | SRR1514878 | |
| Hypochilus gertschi | Chelicerata: Araneae | SRR6997860 | |
| Hypochilus pococki | Chelicerata: Araneae | SRR1514889 | |
| Calponia harrisonfordi | Chelicerata: Araneae | SRR3144089 | |
| Segestria sp. | Chelicerata: Araneae | SRR3144084 | |
| Maoriata sp. | Chelicerata: Araneae | SRR6997868 | |
| Orsolobidae sp. | Chelicerata: Araneae | SRR6998651 | |
| Dysdera crocata | Chelicerata: Araneae | SRR1328258 | |
| Ischnothyreus sp. | Chelicerata: Araneae | SRR6997859 | |
| Opopaea sp. | Chelicerata: Araneae | SRR6998659 | |
| Diguetia sp. | Chelicerata: Araneae | SRR3144093 | |
| Pholcus manueli | Chelicerata: Araneae | SRR1365208 | |
| Pholcus phalangioides | Chelicerata: Araneae | SRR1514900 | |
| Loxosceles deserta | Chelicerata: Araneae | SRR3144077 | |
| Drymusa sp. | Chelicerata: Araneae | SRR6997739 | |
| Periegops suterii | Chelicerata: Araneae | SRR6998656 | |
| Ochyrocera sp. | Chelicerata: Araneae | SRR7028536 | |
| Scytodes globula | Chelicerata: Araneae | SRR6998911 | |
| Scytodes thoracica | Chelicerata: Araneae | SRR1514872 | |
| Archoleptoneta sp. | Chelicerata: Araneae | SRR1514872 | |
| Calileptoneta californica | Chelicerata: Araneae | SRR3144085 | |
| Austrochilus forsteri | Chelicerata: Araneae | SRR6997749 | |
| Hickmania troglodytes | Chelicerata: Araneae | SRR6997862 | |
| Progradungula otwayensis | Chelicerata: Araneae | SRR6998916 | |
| Tarlina sp. | Chelicerata: Araneae | SRR6998918 | |
| Gradungula sorenseni | Chelicerata: Araneae | SRR6997863 | |
| Pianoa isolata | Chelicerata: Araneae | SRR6998914 | |
| Austrarchaea sp. | Chelicerata: Araneae | SRR6997750 | |
| Mecysmauchenius sp. | Chelicerata: Araneae | SRR6997871 | |
| Huttonia palpimanoides | Chelicerata: Araneae | SRR6997861 | |
| Otiothops birabeni | Chelicerata: Araneae | SRR6998652 | |

| | | | |
|---|---|---|---|
| Palpimanus gibbulus | Chelicerata: Araneae | SRR6998653 | |
| Stegodyphus mimosarum | Chelicerata: Araneae | GCA_000611955.2 | |
| Megadictyna thilenii | Chelicerata: Araneae | SRR6997870 | |
| Nicodamidae sp. 2 | Chelicerata: Araneae | SRR6998661 | |
| Nicodamidae sp. 1 | Chelicerata: Araneae | SRR7028539 | |
| Mysmenella jobi | Chelicerata: Araneae | SRR6998907 | |
| Novanapis sp. | Chelicerata: Araneae | SRR6998663 | |
| Anelosimus eximius | Chelicerata: Araneae | SRR6997627 | |
| Latrodectus tredecimguttatus | Chelicerata: Araneae | SRR954929 | |
| Euryopis sp. | Chelicerata: Araneae | SRR6997867 | |
| Parasteatoda tepidariorum | Chelicerata: Araneae | GCA_000365465.2 | X |
| Pararchaea alba | Chelicerata: Araneae | SRR6998655 | |
| Malkara sp. | Chelicerata: Araneae | SRR6997869 | |
| Malkaridae sp. | Chelicerata: Araneae | SRR6997874 | |
| Perissopmeros sp. | Chelicerata: Araneae | SRR6998657 | |
| Australomimetus clean | Chelicerata: Araneae | SRR6997751 | |
| Ero leonina | Chelicerata: Araneae | SRR1514886 | |
| Arkys sp. | Chelicerata: Araneae | SRR6997752 | |
| Demadiana sp. | Chelicerata: Araneae | SRR6997740 | |
| Leucauge venusta | Chelicerata: Araneae | SRR1145740 | |
| Meta ovalis | Chelicerata: Araneae | SRR6997876 | |
| Nanometa sp. | Chelicerata: Araneae | SRR6998667 | |
| Tetragnatha tantalus | Chelicerata: Araneae | SRR1427108 | |
| Cepheia longiseta | Chelicerata: Araneae | SRR6997742 | |
| Mysmena leichardti | Chelicerata: Araneae | SRR6998665 | |
| Microdipoena guttata | Chelicerata: Araneae | SRR1333842 | |
| Spinanapis sp. | Chelicerata: Araneae | SRR6998666 | |
| Tekelloides sp. 2 | Chelicerata: Araneae | SRR6998920 | |
| Laminacauda parvipalpis | Chelicerata: Araneae | SRR6997873 | X |
| Frontinella communis | Chelicerata: Araneae | SRR1145739 | |
| Novafroneta gladiatrix | Chelicerata: Araneae | SRR6998662 | |
| Synotaxus cf turbinatus | Chelicerata: Araneae | SRR6998908 | |
| Nesticus bishopi | Chelicerata: Araneae | SRR1655191 | |
| Nesticus cooperi | Chelicerata: Araneae | SRR1514892 | |
| Meringa sp. | Chelicerata: Araneae | SRR6997877 | |
| Runga sp. | Chelicerata: Araneae | SRR6998910 | |
| Physoglenes sp. | Chelicerata: Araneae | SRR6998913 | |
| Physoglenidae sp. | Chelicerata: Araneae | SRR7028534 | |
| Ogulnius sp. | Chelicerata: Araneae | SRR7028537 | |
| Baalzebub sp. | Chelicerata: Araneae | SRR6997748 | |
| Theridiosoma gemmosum | Chelicerata: Araneae | SRR6998922 | |
| Theridiosoma savannum | Chelicerata: Araneae | SRR7028533 | |
| Trichonephila clavipes | Chelicerata: Araneae | GCA_002102615.1 | |
| Micrathena gracilis | Chelicerata: Araneae | SRR1514882 | |
| Verrucosa arenata | Chelicerata: Araneae | SRR3144087 | |
| Cyrtophora sp. | Chelicerata: Araneae | SRR6997738 | |
| Neoscona arabesca | Chelicerata: Araneae | SRR1145741 | |
| Gasteracantha hasselti | Chelicerata: Araneae | SRR1048659 | |
| Macracantha arcuata | Chelicerata: Araneae | SRR1048826 | |
| Menneus sp. | Chelicerata: Araneae | SRR6997878 | |
| Deinopis sp. | Chelicerata: Araneae | SRR6997737 | |
| Deinopis longipes | Chelicerata: Araneae | SRR1514879 | |
| Tamopsis sp. | Chelicerata: Araneae | SRR6998917 | |
| Oecobius navus | Chelicerata: Araneae | SRR1514899 | |
| Oecobius cellariorum | Chelicerata: Araneae | SRR1365089 | |
| Cybaeodamus taim | Chelicerata: Araneae | SRR6997744 | |
| Forsterella sp. | Chelicerata: Araneae | SRR6997864 | |
| Caayguara ybytyriguara | Chelicerata: Araneae | SRR6997747 | |

| | | |
|---|---|---|
| Amaurobius ferox | Chelicerata: Araneae | SRR1329250 |
| Callobius sp. | Chelicerata: Araneae | SRR3144088 |
| Neoramia sp. | Chelicerata: Araneae | SRR6998660 |
| Amphinecta sp. | Chelicerata: Araneae | SRR6997628 |
| Metaltella simoni | Chelicerata: Araneae | SRR3144078 |
| Cambridgea sp. | Chelicerata: Araneae | SRR6997745 |
| Paramatachia sp. | Chelicerata: Araneae | SRR6998654 |
| Agelenopsis emertoni | Chelicerata: Araneae | SRR1514895 |
| Agelenopsis pennsylvanica | Chelicerata: Araneae | SRR1329248 |
| Calymmaria sp. | Chelicerata: Araneae | SRR6997746 |
| Calymmaria persica | Chelicerata: Araneae | SRR3144091 |
| Cicurina travisae | Chelicerata: Araneae | SRR1654705 |
| Cicurina vibora | Chelicerata: Araneae | SRR1514883 |
| Homalonychus theologus | Chelicerata: Araneae | SRR3144075 |
| Tengella radiata | Chelicerata: Araneae | SRR7028532 |
| Uliodon sp. | Chelicerata: Araneae | SRR6998923 |
| Misumenoides formosipes | Chelicerata: Araneae | SRR3144080 |
| Sidymella sp. | Chelicerata: Araneae | SRR6998912 |
| Anahita punctulata | Chelicerata: Araneae | SRR3144072 |
| Peucetia longipalpis | Chelicerata: Araneae | SRR1514898 |
| Dolomedes triton | Chelicerata: Araneae | SRR3144094 |
| Pisaurina mira | Chelicerata: Araneae | SRR1365651 |
| Cupiennius sp. | Chelicerata: Araneae | SRR7028538 |
| Allocosa alticeps | Chelicerata: Araneae | SRR6997626 |
| Schizocosa rovneri | Chelicerata: Araneae | SRR1514894 |
| Habronattus signatus | Chelicerata: Araneae | SRR1514888 |
| Habronattus ustulatus | Chelicerata: Araneae | SRR1656783 |
| Teminius sp. | Chelicerata: Araneae | SRR6998921 |
| Karaops raveni | Chelicerata: Araneae | SRR6997858 |
| Falconina gracilis | Chelicerata: Araneae | SRR6997866 |
| Nyssus sp. | Chelicerata: Araneae | SRR6998658 |
| Clubiona sp. | Chelicerata: Araneae | SRR6997741 |
| Rebilus sp. | Chelicerata: Araneae | SRR6998909 |
| Liocranidae sp. | Chelicerata: Araneae | SRR6997875 |
| Trachelas tranquillus | Chelicerata: Araneae | SRR1329247 |
| Sergiolus capulatus | Chelicerata: Araneae | SRR1514903 |
| Molycria sp. | Chelicerata: Araneae | SRR6998664 |
| Anzacia sp. | Chelicerata: Araneae | SRR6997629 |
| Lampona sp. | Chelicerata: Araneae | SRR6997872 |
| Zosis sp. | Chelicerata: Araneae | SRR6998924 |
| Philoponella herediae | Chelicerata: Araneae | SRR1514880 |
| Uloborus glomosus | Chelicerata: Araneae | SRR1328334 |
| Eophalangium sheari | Chelicerata: Opiliones | fossil |
| Hastocularis argus | Chelicerata: Opiliones | fossil |
| Leptopsalis sp. MCZ:IZ-141287 | Chelicerata: Opiliones | SAMN23391046 |
| Miopsalis sp. MCZ:IZ-140867 | Chelicerata: Opiliones | SRR5234539 |
| Parapurcellia silvicola MCZ:IZ-134743 | Chelicerata: Opiliones | SRX6374208 |
| Purcellia illustrans MCZ:IZ-49518 | Chelicerata: Opiliones | SRX6374206 |
| Chileogovea oedipus MCZ:IZ-138106 | Chelicerata: Opiliones | SRR9611065 |
| Rakaia minutissima MCZ:IZ-143240 | Chelicerata: Opiliones | SRR9611073 |
| Rakaia stewartiensis MCZ:IZ-49801 | Chelicerata: Opiliones | SRR9611075 |
| Rakaia magna australis MCZ:IZ-29212 | Chelicerata: Opiliones | SRR5181449 |
| Rakaia pauli MCZ:IZ-144621 | Chelicerata: Opiliones | SRR9611074 |
| Rakaia pauli reassembled | Chelicerata: Opiliones | SRR9611074 |
| Pettalus thwaitesi MCZ:IZ-132349 | Chelicerata: Opiliones | SRX6374207 |
| Austropurcellia alata MCZ:IZ-141418 | Chelicerata: Opiliones | SRR9611069 |
| Austropurcellia despectata MCZ:IZ-141417 | Chelicerata: Opiliones | SRR9611064 |
| Austropurcellia cf acuta MCZ:IZ-141419 | Chelicerata: Opiliones | SRR9611062 |

| | | | |
|---|---|---|---|
| Austropurcellia acuta MCZ:IZ-44060 | Chelicerata: Opiliones | SRR9611068 | |
| Austropurcellia clousei MCZ:IZ-141420 | Chelicerata: Opiliones | SRR9611063 | X |
| Neopurcellia salmoni MCZ:IZ-144625 | Chelicerata: Opiliones | SRR9611061 | |
| Neopurcellia salmoni MCZ:IZ-29310 | Chelicerata: Opiliones | SRR5181448 | |
| Karripurcellia peckorum MCZ:IZ-49989 | Chelicerata: Opiliones | SRR9611060 | |
| Aoraki denticulata Northern MCZ:IZ-141172 | Chelicerata: Opiliones | SRR9611067 | |
| Aoraki denticulata Southern MCZ:IZ-141173 | Chelicerata: Opiliones | SRR9611066 | |
| Aoraki denticulata Southern reassembled | Chelicerata: Opiliones | SRR9611066 | |
| Aoraki longitarsa MCZ:IZ-137238 | Chelicerata: Opiliones | SRR5181577 | |
| Siro boyerae MCZ:IZ-137238 | Chelicerata: Opiliones | SRR1145699 | X |
| Metagovea oviformis MCZ:IZ-136533 | Chelicerata: Opiliones | SRR5230765 | |
| Metasiro savannahensis MCZ:IZ-133799 | Chelicerata: Opiliones | SRX205297 | |
| Brasilogovea microphaga MCZ:IZ-136559 | Chelicerata: Opiliones | SRR5185916 | |
| Neogovea matawai MCZ:IZ-133922 | Chelicerata: Opiliones | SRX6098501 | |
| Acropsopilio neozealandiae MCZ:IZ-30457 | Chelicerata: Opiliones | SRR5235984 | |
| Hesperonemastoma modestum | Chelicerata: Opiliones | SRX450937 | |
| Ortholasma coronadense | Chelicerata: Opiliones | SRX451776 | |
| Trogulus martensi | Chelicerata: Opiliones | SRX450964 | |
| Caddo agilis MCZ:IZ-40415 | Chelicerata: Opiliones | SRR17057468 | |
| Thrasychirus modestus MCZ:IZ-49762 | Chelicerata: Opiliones | SRR5234747 | |
| Forsteropsalis pureora MCZ:IZ-29216 | Chelicerata: Opiliones | SRR5234746 | |
| Pantopsalis cheliferoides MCZ:IZ-133328 | Chelicerata: Opiliones | SRR17057465 | |
| Mangatangi parvum MCZ:IZ-133381 | Chelicerata: Opiliones | SAMN23435449 | |
| Megalopsalis triascuta MCZ:IZ-133420 | Chelicerata: Opiliones | SRR17057470 | |
| Diguetinus raptator MCZ:IZ-143123 | Chelicerata: Opiliones | SAMN23435445 | |
| Phalangium opilio MCZ:IZ-29189 | Chelicerata: Opiliones | SRR1145735 | |
| Protolophus singularis | Chelicerata: Opiliones | SRX450934 | |
| Prionostemma vittatum MCZ:IZ-144108 | Chelicerata: Opiliones | SAMN23435457 | X |
| Leiobunum verrucosum | Chelicerata: Opiliones | SRX450936 | |
| Paranelima sp. MCZ:IZ-143124 | Chelicerata: Opiliones | SAMN23435455 | |
| Sclerobunus robustus | Chelicerata: Opiliones | SRX647390 | |
| Synthetonychia glacialis MCZ:IZ-137212 | Chelicerata: Opiliones | SRR5236909 | |
| Larifuga capensis MCZ:IZ-49747 | Chelicerata: Opiliones | SRR1145742 | |
| Nuncia sp. MCZ:IZ-133135 | Chelicerata: Opiliones | SAMN23435453 | |
| Pristobunus sp. MCZ:IZ-152391 | Chelicerata: Opiliones | SAMN23435458 | |
| Scotolemon lespesi MCZ:IZ-43672 | Chelicerata: Opiliones | SRR17068839 | |
| Sitalcina lobata | Chelicerata: Opiliones | SRX451777 | |
| Gnomulus rostratus MCZ:IZ-141286 | Chelicerata: Opiliones | SRX2549521 | |
| Dibunus sp. MCZ:IZ-141285 | Chelicerata: Opiliones | SRX2549524 | |
| Epedanus pinangensis MCZ:IZ-141288 | Chelicerata: Opiliones | SAMN23435446 | |
| Haasus judaeus | Chelicerata: Opiliones | SRR17027468 | |
| Haasus naasane | Chelicerata: Opiliones | SRX5786020 | |
| Dampetrus sp. MCZ:IZ-140868 | Chelicerata: Opiliones | SRX2549519 | |
| Metabiantes sp. MCZ:IZ-49748 | Chelicerata: Opiliones | SRX451770 | X |
| Stygnomma sp. MCZ:IZ-144060 | Chelicerata: Opiliones | SAMN23435460 | |
| Pellobunus sp. | Chelicerata: Opiliones | SRX2549520 | |
| Kimulidae sp. MCZ:IZ-143960 | Chelicerata: Opiliones | SAMN23435448 | |
| Tegipiolus pachypus MZSP71018 | Chelicerata: Opiliones | SRR17068835 | |
| Zalmoxidae gen. sp. MCZ:IZ-144055 | Chelicerata: Opiliones | SAMN23435456 | |
| Pachylicus acutus MCZ:IZ-49749 | Chelicerata: Opiliones | SRX451775 | |
| Maracaynatum trinidaense MCZ:IZ-144057 | Chelicerata: Opiliones | SAMN23435450 | |
| Avima matintaperera MCZ:IZ-136560 | Chelicerata: Opiliones | SRX2548330 | |
| Karos barbarikos MCZ:IZ-46444 | Chelicerata: Opiliones | SRR17057455 | |
| Chapulobunus sp. MCZ:IZ-46443 | Chelicerata: Opiliones | SRX2548334 | |
| Stygnoplus clavotibialis MCZ:IZ-143966 | Chelicerata: Opiliones | SAMN23435461 | |
| Protimesius longipalpis MCZ:IZ-136522 | Chelicerata: Opiliones | SRX2548335 | |
| Auranus hoeferscovitorum MCZ:IZ-136531 | Chelicerata: Opiliones | SRR17068838 | |
| Pickeliana pickeli MCZ:IZ-139249 | Chelicerata: Opiliones | SRR17068850 | |

| | | | |
|---|---|---|---|
| Gonycranaus pluto MCZ:IZ-139251 | Chelicerata: Opiliones | SRR17068869 | |
| Camarana flavipalpi MCZ:IZ-139271 | Chelicerata: Opiliones | SRR17068833 | |
| Pseudopachylus longipes MCZ:IZ-32171 | Chelicerata: Opiliones | SRR5241471 | |
| Bissula sp. MCZ:IZ-139255 | Chelicerata: Opiliones | SRR17068834 | |
| Pseudopachylus eximius MCZ:IZ-139280 | Chelicerata: Opiliones | SRR17068846 | |
| Vonones ornata MCZ:IZ-49750 | Chelicerata: Opiliones | SRR1145738 | |
| Incasarcus argenteus MCZ:IZ-139250 | Chelicerata: Opiliones | SRR17068861 | |
| Quindina limbata MZSP-71772 | Chelicerata: Opiliones | SRR17068842 | |
| Saramacia lucasae MCZ:IZ-136518 | Chelicerata: Opiliones | SRR5241473 | |
| Ampycinae sp. MCZ:IZ-46455 | Chelicerata: Opiliones | SRR17068849 | |
| Glysterus sp. MCZ:IZ-140069 | Chelicerata: Opiliones | SRR17068830 | |
| Panalus robustus MZSP-71019 | Chelicerata: Opiliones | SRR17068852 | |
| Phalangodus cottus MZSP-71020 | Chelicerata: Opiliones | SRR17068851 | |
| Santinezia serratotibialis MCZ:IZ-144078 | Chelicerata: Opiliones | SRR17057460 | |
| Phareicranaus manauara MCZ:IZ-136554 | Chelicerata: Opiliones | SRX2548333 | |
| Pseudopucrolia discrepans MCZ:IZ-139277 | Chelicerata: Opiliones | SRR17068845 | |
| Sadocus polyacanthus MCZ:IZ-138113 | Chelicerata: Opiliones | SRR17068840 | |
| Eubalta planiceps MCZ:IZ-49760 | Chelicerata: Opiliones | SRR17068832 | |
| Metagyndes innata MCZ:IZ-29745 | Chelicerata: Opiliones | SRR5241474 | |
| Hernandaria una MCZ:IZ-139258 | Chelicerata: Opiliones | SRR17068862 | |
| Graphinotus therezopolis MCZ:IZ-139273 | Chelicerata: Opiliones | SRR17068863 | |
| Meteusarcoides sp. MCZ:IZ-139264 | Chelicerata: Opiliones | SAMN23435452 | |
| Meteusarcoides caudatus MZSP-71024 | Chelicerata: Opiliones | SRR17068856 | |
| Acutisoma longipes MCZ:IZ-139261 | Chelicerata: Opiliones | SRR17068871 | |
| Goniosoma sp. MCZ:IZ-139266 | Chelicerata: Opiliones | SRR17068870 | |
| Roeweria virescens MCZ:IZ-139279 | Chelicerata: Opiliones | SRR17068841 | |
| Longiperna concolor MCZ:IZ-139260 | Chelicerata: Opiliones | SRR17068857 | |
| Gonyleptidae sp. MCZ IZ-139272 | Chelicerata: Opiliones | SAMN23435462 | |
| Mitobates conspersus MCZ:IZ-139263 | Chelicerata: Opiliones | SRR17068854 | |
| Promitobates ornatus MZSP-71022 | Chelicerata: Opiliones | SRR17068847 | |
| Gonyleptellus pustulosus MCZ:IZ-139269 | Chelicerata: Opiliones | SRR17068868 | |
| Gonyleptes fragilis MZSP-71023 | Chelicerata: Opiliones | SRR17068866 | |
| Pseudotrogulus mirim MCZ:IZ-139270 | Chelicerata: Opiliones | SRR17068844 | |
| Pseudotrogulus trostkyi MCZ:IZ-139278 | Chelicerata: Opiliones | SRR17068843 | |
| Ampheres leucopheus MCZ:IZ-139267 | Chelicerata: Opiliones | SRR17068860 | |
| Acanthogonyleptes aff fulvigranulatus MZSP-71027 | Chelicerata: Opiliones | SRR17068872 | |
| Gonyleptes atrus MZSP-71025 | Chelicerata: Opiliones | SRR17068867 | |
| Gonyleptes sp. MCZ:IZ-139262 | Chelicerata: Opiliones | SRR17068865 | |
| Geraecormobius bispinifrons MCZ:IZ-139274 | Chelicerata: Opiliones | SRR17068831 | |
| Mischonyx cuspidatus MCZ:IZ-139265 | Chelicerata: Opiliones | SRR17068855 | |
| Sodreana leprevosti MCZ:IZ-139256 | Chelicerata: Opiliones | SRR17068837 | |
| Sodreana sodrena MCZ:IZ-139268 | Chelicerata: Opiliones | SRR17068836 | |
| Progonyleptoidellus striatus MZSP-71021 | Chelicerata: Opiliones | SRR17068848 | |
| Iporangaia pustulosa MCZ:IZ-139275 | Chelicerata: Opiliones | SRR17068858 | |
| Gonyleptoides marumbiensis MCZ:IZ-139254 | Chelicerata: Opiliones | SRR17068864 | |
| Neosadocus maximus MCZ:IZ-139257 | Chelicerata: Opiliones | SRR17068853 | |
| Pseudotyrannochthonius sp. B MCZ:IZ-144011 | Chelicerata: Pseudoscorpiones | SRR9331997 | |
| Pseudotyrannochthonius sp. MCZ:IZ-149275 | Chelicerata: Pseudoscorpiones | SRR9331998 | |
| Lechytia hoffi MCZ:IZ-139248 | Chelicerata: Pseudoscorpiones | SRR9331999 | |
| Lagynochthonius australicus MCZ:IZ-143136 | Chelicerata: Pseudoscorpiones | SRR9332016 | |
| Ephippiochthonius tetrachelatus MCZ:IZ-140293 | Chelicerata: Pseudoscorpiones | SRR9331983 | |
| Lechytia hoffi MCZ:IZ-139248 | Chelicerata: Pseudoscorpiones | SRR9332011 | |
| Feaella capensis MCZ:IZ-139240 | Chelicerata: Pseudoscorpiones | SRR9331986 | X |
| Pseudogarypus banksi MCZ:IZ-140301 | Chelicerata: Pseudoscorpiones | SRR9331993 | |
| Bochica withi MCZ:IZ-144332 | Chelicerata: Pseudoscorpiones | SRR9332002 | |
| Dhanus sumatranus MCZ:IZ-49986 | Chelicerata: Pseudoscorpiones | SRR9331984 | |
| Indohya sp. MCZ:IZ-151267 | Chelicerata: Pseudoscorpiones | SRR9332015 | |
| Ideobisium crassimanum MCZ:IZ-144356 | Chelicerata: Pseudoscorpiones | SRR9331979 | |

| | | | |
|---|---|---|---|
| Parahya submersa MCZ:IZ-139241 | Chelicerata: Pseudoscorpiones | SRR9331989 | |
| Gymnobisium sp. MCZ:IZ-139238 | Chelicerata: Pseudoscorpiones | SRR9331987 | |
| Microcreagrinae sp. MCZ:IZ-60626 | Chelicerata: Pseudoscorpiones | SRR9332009 | |
| Microbisium brunneum MCZ:IZ-141276 | Chelicerata: Pseudoscorpiones | SRR9332013 | |
| Microbisium sp. MCZ:IZ-60599 | Chelicerata: Pseudoscorpiones | SRR9332014 | |
| Novobisium sp. MCZ:IZ-141855 | Chelicerata: Pseudoscorpiones | SRR9331992 | X |
| Afrogarypus subimpressus MCZ:IZ-139239 | Chelicerata: Pseudoscorpiones | SRR9332004 | |
| Geogarypus maculatus MCZ:IZ-144335 | Chelicerata: Pseudoscorpiones | SRR9331988 | |
| Apolpium sp. MCZ:IZ-144360 | Chelicerata: Pseudoscorpiones | SRR9332003 | X |
| Anchigarypus californicus MCZ:IZ-149276 | Chelicerata: Pseudoscorpiones | SRR9331985 | X |
| Synsphyronus apimelus | Chelicerata: Pseudoscorpiones | SRR7062201 | |
| Pseudogarypinus frontalis MCZ:IZ-139246 | Chelicerata: Pseudoscorpiones | SRR9331994 | |
| Larca granulata MCZ:IZ-140297 | Chelicerata: Pseudoscorpiones | SRR9332018 | |
| Protogarypinus giganteus | Chelicerata: Pseudoscorpiones | SRR9331995 | |
| Cheiridiidae sp. MCZ:IZ-140294 | Chelicerata: Pseudoscorpiones | SRR9332008 | |
| Sternophoridae sp. MCZ:IZ-133496 | Chelicerata: Pseudoscorpiones | SRR9332020 | |
| Cacodemonius segmentidentatus MCZ:IZ-144363 | Chelicerata: Pseudoscorpiones | SRR9332001 | |
| Oratemnus curtus MCZ:IZ-49983 | Chelicerata: Pseudoscorpiones | SRR9331991 | |
| Protochelifer sp. MCZ:IZ-49984 | Chelicerata: Pseudoscorpiones | SRR9331996 | |
| Cheliferidae sp. MCZ:IZ-40982 | Chelicerata: Pseudoscorpiones | SRR9332007 | |
| Parachelifer persimilis MCZ:IZ-139247 | Chelicerata: Pseudoscorpiones | SRR9331990 | |
| Lamprochernes savignyi MCZ:IZ-149278 | Chelicerata: Pseudoscorpiones | SRR9332017 | |
| Conicochernes crassus MCZ:IZ-49985 | Chelicerata: Pseudoscorpiones | SRR9331982 | |
| Hesperochernes sp. | Chelicerata: Pseudoscorpiones | SRR1514877 | |
| Haplochernes kraepelini | Chelicerata: Pseudoscorpiones | SRR1767661 | |
| Chernetidae sp. MCZ:IZ-142323 | Chelicerata: Pseudoscorpiones | SRR9332006 | X |
| Chernetidae sp. JC032 | Chelicerata: Pseudoscorpiones | SRR9332005 | |
| Waeringoscorpio | Chelicerata: Scorpiones | fossil | |
| Pulmonoscorpius | Chelicerata: Scorpiones | fossil | |
| Palaeoscorpius | Chelicerata: Scorpiones | fossil | |
| Compsoscorpius | Chelicerata: Scorpiones | fossil | |
| Proscorpius | Chelicerata: Scorpiones | fossil | |
| Chaerilus celebensis | Chelicerata: Scorpiones | SRR1721804 | X |
| Lychas variatus | Chelicerata: Scorpiones | SAMN23426204 | |
| Mesobuthus martensii | Chelicerata: Scorpiones | GCA_000484575.1 | |
| Birulatus israelensis | Chelicerata: Scorpiones | SAMN23426190 | |
| Compsobuthus levyi | Chelicerata: Scorpiones | SAMN23426194 | |
| Compsobuthus sp. | Chelicerata: Scorpiones | SAMN23426196 | |
| Compsobuthus schmiedeknechti | Chelicerata: Scorpiones | SAMN23426195 | |
| Androctonus amoreuxi | Chelicerata: Scorpiones | SAMN09939440 | |
| Androctonus australis | Chelicerata: Scorpiones | SRR1724216 | |
| Hottentotta trilineatus | Chelicerata: Scorpiones | SRR1721800 | |
| Orthochirus scrobiculosus | Chelicerata: Scorpiones | SAMN23426205 | |
| Androctonus crassicauda | Chelicerata: Scorpiones | SAMN23426188 | |
| Buthus israelis | Chelicerata: Scorpiones | SAMN23426192 | |
| Buthacus cf. arenicola | Chelicerata: Scorpiones | SAMN23426191 | |
| Leiurus hebraeus | Chelicerata: Scorpiones | SAMN23426202 | |
| Leiurus quinquestriatus | Chelicerata: Scorpiones | SAMN23426203 | |
| Ananteris balzani | Chelicerata: Scorpiones | SAMN09907396 | |
| Babycurus gigas | Chelicerata: Scorpiones | SAMN23426189 | |
| Grosphus grandidieri | Chelicerata: Scorpiones | SAMN23426197 | |
| Parabuthus transvaalicus | Chelicerata: Scorpiones | SRR1721799 | |
| Uroplectes olivaceus | Chelicerata: Scorpiones | SAMN23426211 | |
| Uropolectes vittatus | Chelicerata: Scorpiones | SAMN23426212 | |
| Tityus smithi | Chelicerata: Scorpiones | SAMN23426209 | |
| Tityus maranhensis | Chelicerata: Scorpiones | SAMN23426208 | |
| Tityus costatus | Chelicerata: Scorpiones | SAMN23426207 | |
| Tityus serrulatus | Chelicerata: Scorpiones | SRX701006, SRX708114 | |

| | | | |
|---|---|---|---|
| Troglorhopalurus lacrau | Chelicerata: Scorpiones | SAMN23426210 | |
| Physoctonus debilis | Chelicerata: Scorpiones | SAMN23426206 | |
| Jaguajir agamemnon | Chelicerata: Scorpiones | SAMN23426200 | |
| Jaguajir rochai | Chelicerata: Scorpiones | SAMN23426201 | |
| Heteroctenus junceus | Chelicerata: Scorpiones | SAMN23426199 | |
| Heteroctenus garridoi | Chelicerata: Scorpiones | SAMN23426198 | |
| Centruroides caribensis | Chelicerata: Scorpiones | SAMN23426193 | |
| Centruroides hentzi | Chelicerata: Scorpiones | SRX3189904, SRX3189905 | |
| Centruroides vittatus | Chelicerata: Scorpiones | SRR1515193 | |
| Centruroides sculpturatus | Chelicerata: Scorpiones | SRR1515193 | X |
| Iurus dekanum | Chelicerata: Scorpiones | SRR1721734 | |
| Bothriurus coriaceus | Chelicerata: Scorpiones | SRR6467511 | |
| Bothriurus burmeisteri | Chelicerata: Scorpiones | SRR1721670 | |
| Centromachetes sp. | Chelicerata: Scorpiones | SRR6467879 | |
| Cercophonius squama | Chelicerata: Scorpiones | SRR6470446 | |
| Cercophomius queenslandae | Chelicerata: Scorpiones | SRR6466561 | |
| Cercophonius sulcatus | Chelicerata: Scorpiones | SRR6470146 | |
| Caraboctonus keyserlingii | Chelicerata: Scorpiones | SRX5829265 | |
| Superstitiona donensis | Chelicerata: Scorpiones | SRR1721951 | |
| Anuroctonus phaiodactylus | Chelicerata: Scorpiones | SRR1721879 | |
| Brotheas granulatus | Chelicerata: Scorpiones | SRR1721887 | |
| Scorpiops sp. | Chelicerata: Scorpiones | SRR1767662 | |
| Euscorpius italicus | Chelicerata: Scorpiones | SRR1721892 | |
| Plesiochactas dilutus | Chelicerata: Scorpiones | SRR7250103 | X |
| Megacormus gertschi | Chelicerata: Scorpiones | SRR3657526 | |
| Megacormus sp. | Chelicerata: Scorpiones | SRR1767669 | |
| Uroctonus mordax | Chelicerata: Scorpiones | SRR8518581 | |
| Belisarius xambeui | Chelicerata: Scorpiones | SRR1721953 | |
| Hoffmannihadrurus aztecus | Chelicerata: Scorpiones | ERX3551392 | |
| Hadrurus arizonensis | Chelicerata: Scorpiones | SRR1721733 | |
| Hadrurus concolorous | Chelicerata: Scorpiones | ERX3560276 | |
| Hadrurus spadix | Chelicerata: Scorpiones | SRX2056046 | X |
| Uroctonites huachuca | Chelicerata: Scorpiones | SRR8518582 | |
| Kovarikia boggerti | Chelicerata: Scorpiones | SRX5322100 | |
| Pseudouroctonus apacheanus | Chelicerata: Scorpiones | SRR8518585 | |
| Vaejovis cashi | Chelicerata: Scorpiones | SRR8518583 | |
| Paruroctonus baergi | Chelicerata: Scorpiones | SRR7443668 | |
| Smeringurus mesaensis | Chelicerata: Scorpiones | SRR7473845 | |
| Smeringurus vachoni | Chelicerata: Scorpiones | SRR7474136 | |
| Vaejovis mexicanus | Chelicerata: Scorpiones | SRR7421527 | |
| Serradigitus gertschi | Chelicerata: Scorpiones | ERX2850109 | |
| Konetontil acapulco | Chelicerata: Scorpiones | SRR7422029 | |
| Konetontli chamelaensis | Chelicerata: Scorpiones | SRR7427084 | |
| Mesomexovis aff variegatus | Chelicerata: Scorpiones | SRR7439652 | |
| Paravaejovis schwenkmeyeri | Chelicerata: Scorpiones | ERX2670405 | |
| Paravaejovis spinigerus | Chelicerata: Scorpiones | SRR1721954 | |
| Kuarapu purepecha | Chelicerata: Scorpiones | SRR7439043 | |
| Chihuahuanus coahuilae | Chelicerata: Scorpiones | SRR7439185 | |
| Mesovexovis occidentalis | Chelicerata: Scorpiones | SRR7439610 | |
| Thorellius intrepidus | Chelicerata: Scorpiones | SRR7427141 | |
| Urodacus yaschenkoi | Chelicerata: Scorpiones | SRX685583 | |
| Urodacus elongatus | Chelicerata: Scorpiones | SRX4723123 | |
| Urodacus planimanus | Chelicerata: Scorpiones | SAMN06114579 | |
| Hormiops davidovi | Chelicerata: Scorpiones | SAMN23438338 | |
| Hormurus weberi | Chelicerata: Scorpiones | SAMN23438339 | |
| Liocheles australasiae | Chelicerata: Scorpiones | SRR1721664 | |
| Liocheles sp. KhaoDeng | Chelicerata: Scorpiones | SAMN23438340 | |
| Nebo hierichonticus | Chelicerata: Scorpiones | SAMN23438341 | |

| | | |
|---|---|---|
| Diplocentrus diablo | Chelicerata: Scorpiones | SRR1721672 |
| Diplocentrus zacatecanus | Chelicerata: Scorpiones | SAMN23438342 |
| Kolotl magnus | Chelicerata: Scorpiones | SRX4717881 |
| Kolotl poncei | Chelicerata: Scorpiones | SAMN23438343 |
| Iomachus politus | Chelicerata: Scorpiones | SAMN23438344 |
| Hadogenes troglodytes | Chelicerata: Scorpiones | SRR1721665 |
| Hadogenes paucidens | Chelicerata: Scorpiones | SAMN23438345 |
| Opisthacanthus asper | Chelicerata: Scorpiones | SAMN23438346 |
| Opisthacanthus validus | Chelicerata: Scorpiones | SAMN23438347 |
| Chiromachus ochropus | Chelicerata: Scorpiones | SAMN23438348 |
| Opisthacanthus madagascarensis | Chelicerata: Scorpiones | SRR1721668 |
| Palaeocheloctonus pauliani | Chelicerata: Scorpiones | SAMN23438349 |
| Opistophthalmus glabrifrons | Chelicerata: Scorpiones | SAMN23438350 |
| Pandinus imperator | Chelicerata: Scorpiones | SRR1721600 |
| Opistophthalmus walhbergii | Chelicerata: Scorpiones | SAMN23438351 |
| Opistophthalmus boehmi | Chelicerata: Scorpiones | SAMN23438352 |
| Scorpio cf. maurus | Chelicerata: Scorpiones | SRR7249741 |
| Troglokhammouanus steineri | Chelicerata: Scorpiones | SRR1721739 |
| Vietbocap lao | Chelicerata: Scorpiones | SRR1721740 |
| Goniotarbus | Chelicerata: Phalangiotarbida | fossil |
| Bonatarbus | Chelicerata: Phalangiotarbida | fossil |

**Table S2.** Species sampling and BUSCO completeness.

| Terminal | Taxonomy | Complete | Fragmented | Duplicated | Missing | BUSCO completeness (%) |
|---|---|---|---|---|---|---|
| *Epiperipatus sp.* | Onychophora | 510 | 185 | 260 | 111 | 89.59 |
| *Opisthopatus kwazululandi* | Onychophora | 340 | 178 | 473 | 75 | 92.96 |
| *Peripatopsis overbergiensis* | Onychophora | 381 | 269 | 73 | 343 | 67.82 |
| *Eudigraphis taiwaniensis* | Myriapoda: Diplopoda | 435 | 93 | 493 | 45 | 95.78 |
| *Narceus americanus* | Myriapoda: Diplopoda | 320 | 290 | 217 | 239 | 77.58 |
| *Pauropus huxleyi* | Myriapoda: Pauropoda | 260 | 119 | 639 | 48 | 95.50 |
| *Symphylella sp.* | Myriapoda: Symphyla | 383 | 207 | 363 | 113 | 89.40 |
| *Scutigera coleoptrata* | Myriapoda: Chilopoda | 194 | 370 | 381 | 121 | 88.65 |
| *Craterostigmus crabilli* | Myriapoda: Chilopoda | 291 | 304 | 199 | 272 | 74.48 |
| *Lithobius atkinsoni* | Myriapoda: Chilopoda | 491 | 68 | 477 | 30 | 97.19 |
| *Alipes grandidieri* | Myriapoda: Chilopoda | 550 | 211 | 242 | 63 | 94.09 |
| *Strigamia maritima* | Myriapoda: Chilopoda | 951 | 19 | 40 | 56 | 94.75 |
| *Eusarsiella sp.* | Pancrustacea: Ostracoda | 284 | 247 | 440 | 95 | 91.09 |
| *Daphnia magna* | Pancrustacea: Branchiopoda | 974 | 40 | 10 | 42 | 96.06 |
| *Daphnia pulex* | Pancrustacea: Branchiopoda | 1000 | 15 | 24 | 27 | 97.47 |
| *Eurytemora affinis* | Pancrustacea: Copepoda | 770 | 41 | 224 | 31 | 97.09 |
| *Hyalella azteca* | Pancrustacea: Malacostraca | 811 | 28 | 193 | 34 | 96.81 |
| *Folsomia candida* | Pancrustacea: Hexapoda | 768 | 9 | 275 | 14 | 98.69 |
| *Orchesella cincta* | Pancrustacea: Hexapoda | 870 | 39 | 74 | 83 | 92.21 |
| *Gryllus bimaculatus* | Pancrustacea: Hexapoda | 450 | 20 | 543 | 53 | 95.03 |
| *Zootermopsis nevadensis* | Pancrustacea: Hexapoda | 579 | 3 | 476 | 8 | 99.25 |
| *Nasonia vitripennis* | Pancrustacea: Hexapoda | 714 | 5 | 341 | 6 | 99.44 |
| *Drosophila melanogaster* | Pancrustacea: Hexapoda | 540 | 0 | 526 | 0 | 100.00 |
| *Tribolium castaneum* | Pancrustacea: Hexapoda | 807 | 2 | 253 | 4 | 99.62 |
| *Tanystylum orbiculare* | Chelicerata: Pycnogonida | 726 | 20 | 269 | 51 | 95.22 |
| *Nymphon molleri* | Chelicerata: Pycnogonida | 743 | 53 | 228 | 42 | 96.06 |
| *Anoplodactylus insignis* | Chelicerata: Pycnogonida | 525 | 173 | 269 | 99 | 90.71 |
| *Phoxichilidium femoratum* | Chelicerata: Pycnogonida | 547 | 13 | 462 | 44 | 95.87 |
| *Pycnogonum litorale* | Chelicerata: Pycnogonida | 882 | 122 | 14 | 48 | 95.50 |
| *Pallenella flava* | Chelicerata: Pycnogonida | 620 | 78 | 317 | 51 | 95.22 |
| *Stylopallene cheilorhynchus* | Chelicerata: Pycnogonida | 545 | 9 | 483 | 29 | 97.28 |
| *Limulus polyphemus* | Chelicerata: Xiphosura | 781 | 148 | 47 | 90 | 91.56 |
| *Carcinoscorpius rotundicauda* | Chelicerata: Xiphosura | 666 | 65 | 301 | 34 | 96.81 |

| | | | | | | |
|---|---|---|---|---|---|---|
| *Tachypleus tridentatus* | Chelicerata: Xiphosura | 696 | 94 | 155 | 121 | 88.65 |
| *Tachypleus gigas* | Chelicerata: Xiphosura | 492 | 320 | 28 | 226 | 78.80 |
| *Eukoenenia hanseni* | Chelicerata: Palpigradi | 267 | 254 | 3 | 542 | 49.16 |
| *Eukoenenia spelaea MCZ* | Chelicerata: Palpigradi | 37 | 80 | 6 | 943 | 11.54 |
| *Eukoenenia spelaea UW* | Chelicerata: Palpigradi | 143 | 237 | 14 | 672 | 36.96 |
| *Galeodes sp.* | Chelicerata: Solifugae | 536 | 182 | 175 | 173 | 83.77 |
| *Solpugema sp IZ49525* | Chelicerata: Solifugae | 701 | 60 | 277 | 28 | 97.37 |
| *Eremobates sp IZ49755* | Chelicerata: Solifugae | 468 | 310 | 82 | 206 | 80.68 |
| *Eremobates sp.* | Chelicerata: Solifugae | 541 | 8 | 500 | 17 | 98.41 |
| *Adenacarus sp.* | Chelicerata: Parasitiformes | 507 | 202 | 94 | 263 | 75.33 |
| *Neocarus sp.* | Chelicerata: Parasitiformes | 724 | 194 | 21 | 127 | 88.09 |
| *Ricinoides atewa* | Chelicerata: Ricinulei | 618 | 235 | 97 | 116 | 89.12 |
| *Ricinoides karschii* | Chelicerata: Ricinulei | 463 | 201 | 234 | 168 | 84.24 |
| *Pseudocellus pearsei* | Chelicerata: Ricinulei | 212 | 209 | 20 | 625 | 41.37 |
| *Pseudocellus sp.* | Chelicerata: Ricinulei | 131 | 215 | 23 | 697 | 34.62 |
| *Cryptocellus sp IZ143922* | Chelicerata: Ricinulei | 219 | 338 | 98 | 411 | 61.44 |
| *Cryptocellus becki* | Chelicerata: Ricinulei | 488 | 180 | 333 | 65 | 93.90 |
| *Cryptocellus sp IZ30913* | Chelicerata: Ricinulei | 558 | 202 | 219 | 87 | 91.84 |
| *Galendromus occidentalis* | Chelicerata: Parasitiformes | 957 | 8 | 67 | 34 | 96.81 |
| *Gromphadorholaelaps schaeferi* | Chelicerata: Parasitiformes | 685 | 85 | 255 | 41 | 96.15 |
| *Varroa destructor* | Chelicerata: Parasitiformes | 394 | 219 | 49 | 404 | 62.10 |
| *Pneumolaelaps niutirani* | Chelicerata: Parasitiformes | 652 | 35 | 325 | 54 | 94.93 |
| *Tropilaelaps mercedesae* | Chelicerata: Parasitiformes | 930 | 64 | 14 | 58 | 94.56 |
| *Orthinodoros rostratus* | Chelicerata: Parasitiformes | 906 | 72 | 11 | 77 | 92.78 |
| *Ixodes scapularis* | Chelicerata: Parasitiformes | 892 | 106 | 18 | 50 | 95.31 |
| *Haemaphysalis longicornis* | Chelicerata: Parasitiformes | 69 | 24 | 932 | 41 | 96.15 |
| *Amblyomma americanum* | Chelicerata: Parasitiformes | 176 | 106 | 699 | 85 | 92.03 |
| *Dermacentor silvarum* | Chelicerata: Parasitiformes | 103 | 20 | 915 | 28 | 97.37 |
| *Hyalomma excavatum* | Chelicerata: Parasitiformes | 740 | 76 | 170 | 80 | 92.50 |
| *Rhipicephalus microplus* | Chelicerata: Parasitiformes | 110 | 126 | 267 | 563 | 47.19 |
| *Labidostommatidae IZ67347* | Chelicerata: Acariformes | 682 | 110 | 212 | 62 | 94.18 |
| *Dinothrombium tinctorium* | Chelicerata: Acariformes | 554 | 24 | 383 | 105 | 90.15 |
| *Panonychus citri* | Chelicerata: Acariformes | 623 | 162 | 191 | 90 | 91.56 |
| *Tetranychus cinnabarinus* | Chelicerata: Acariformes | 913 | 55 | 12 | 86 | 91.93 |
| *Tetranychus urticae* | Chelicerata: Acariformes | 798 | 12 | 203 | 53 | 95.03 |
| *Rhizoglyphus robini* | Chelicerata: Acariformes | 122 | 33 | 755 | 156 | 85.37 |
| *Sarcoptes scabiei* | Chelicerata: Acariformes | 850 | 71 | 12 | 133 | 87.52 |

| | | | | | | |
|---|---|---|---|---|---|---|
| *Dermatophagoides farinae* | Chelicerata: Acariformes | 250 | 259 | 348 | 209 | 80.39 |
| *Dermatophagoides pteronyssinus* | Chelicerata: Acariformes | 894 | 14 | 95 | 63 | 94.09 |
| *Euroglyphus maynei* | Chelicerata: Acariformes | 672 | 228 | 11 | 155 | 85.46 |
| *Hypochthonius rufulus* | Chelicerata: Acariformes | 644 | 26 | 342 | 54 | 94.93 |
| *Steganacarus magnus* | Chelicerata: Acariformes | 145 | 15 | 841 | 65 | 93.90 |
| *Achipteria coleoptrata* | Chelicerata: Acariformes | 575 | 16 | 398 | 77 | 92.78 |
| *Alaskozetes antarcticus* | Chelicerata: Acariformes | 141 | 23 | 810 | 92 | 91.37 |
| *Hermannia gibba* | Chelicerata: Acariformes | 532 | 10 | 477 | 47 | 95.59 |
| *Archegozetes longisetosus* | Chelicerata: Acariformes | 646 | 137 | 233 | 50 | 95.31 |
| *Nothrus palustris* | Chelicerata: Acariformes | 530 | 7 | 487 | 42 | 96.06 |
| *Platynothrus peltifer* | Chelicerata: Acariformes | 310 | 126 | 537 | 93 | 91.28 |
| *Mastigoproctus giganteus* | Chelicerata: Uropygi | 632 | 42 | 370 | 22 | 97.94 |
| *Mayacentrum lacandonensis* | Chelicerata: Uropygi | 254 | 347 | 57 | 408 | 61.73 |
| *Draculoides vinei* | Chelicerata: Schizomida | 712 | 127 | 177 | 50 | 95.31 |
| *Stenochrus portoricensis* | Chelicerata: Schizomida | 826 | 98 | 96 | 46 | 95.68 |
| *Stenochrus sp.* | Chelicerata: Schizomida | 754 | 202 | 22 | 88 | 91.74 |
| *Sarax ioanniticus* | Chelicerata: Amblypygi | 633 | 33 | 379 | 21 | 98.03 |
| *Sarax israelensis* | Chelicerata: Amblypygi | 359 | 7 | 686 | 14 | 98.69 |
| *Damon variegatus* | Chelicerata: Amblypygi | 472 | 288 | 6 | 300 | 71.86 |
| *Acanthophrynus coronatus* | Chelicerata: Amblypygi | 499 | 325 | 19 | 223 | 79.08 |
| *Phrynus marginemaculatus* | Chelicerata: Amblypygi | 543 | 7 | 499 | 17 | 98.41 |
| *Liphistius malayanus* | Chelicerata: Araneae | 551 | 174 | 136 | 205 | 80.77 |
| *Liphistius sp.* | Chelicerata: Araneae | 70 | 94 | 1 | 901 | 15.48 |
| *Sphodros rufipes* | Chelicerata: Araneae | 904 | 68 | 45 | 49 | 95.40 |
| *Megahexura fulva* | Chelicerata: Araneae | 925 | 36 | 88 | 17 | 98.41 |
| *Aliatypus coylei* | Chelicerata: Araneae | 871 | 79 | 47 | 69 | 93.53 |
| *Antrodiaetus unicolor* | Chelicerata: Araneae | 745 | 136 | 35 | 150 | 85.93 |
| *Porrhothele sp.* | Chelicerata: Araneae | 620 | 191 | 167 | 88 | 91.74 |
| *Microhexura montivaga* | Chelicerata: Araneae | 719 | 124 | 44 | 179 | 83.21 |
| *Macrothele calpeiana* | Chelicerata: Araneae | 136 | 220 | 3 | 707 | 33.68 |
| *Paratropis sp.* | Chelicerata: Araneae | 444 | 148 | 24 | 450 | 57.79 |
| *Cyclocosmia truncata* | Chelicerata: Araneae | 596 | 170 | 67 | 233 | 78.14 |
| *Hebestatis theveneti* | Chelicerata: Araneae | 701 | 106 | 35 | 224 | 78.99 |
| *Idiops bersebaensis* | Chelicerata: Araneae | 610 | 148 | 26 | 282 | 73.55 |
| *Aptostichus stephencolberti* | Chelicerata: Araneae | 361 | 140 | 63 | 502 | 52.91 |
| *Promyrmekiaphila clathrata* | Chelicerata: Araneae | 690 | 158 | 33 | 185 | 82.65 |
| *Brachythele longitarsis* | Chelicerata: Araneae | 698 | 149 | 37 | 182 | 82.93 |
| *Pionothele sp.* | Chelicerata: Araneae | 556 | 164 | 14 | 332 | 68.86 |
| *Stanwellia sp.* | Chelicerata: Araneae | 527 | 263 | 185 | 91 | 91.46 |
| *Damarchus sp.* | Chelicerata: Araneae | 555 | 130 | 12 | 369 | 65.38 |

| | | | | | | |
|---|---|---|---|---|---|---|
| *Trichopelma laselva* | Chelicerata: Araneae | 670 | 140 | 23 | 233 | 78.14 |
| *Acanthoscurria geniculata* | Chelicerata: Araneae | 591 | 181 | 71 | 223 | 79.08 |
| *Aphonopelma hentzi* | Chelicerata: Araneae | 584 | 16 | 443 | 23 | 97.84 |
| *Aphonopelma iviei* | Chelicerata: Araneae | 593 | 113 | 18 | 342 | 67.92 |
| *Filistata insidiatrix* | Chelicerata: Araneae | 295 | 263 | 415 | 93 | 91.28 |
| *Kukulcania hibernalis* | Chelicerata: Araneae | 800 | 115 | 51 | 100 | 90.62 |
| *Hypochilus gertschi* | Chelicerata: Araneae | 397 | 362 | 14 | 293 | 72.51 |
| *Hypochilus pococki* | Chelicerata: Araneae | 142 | 127 | 169 | 628 | 41.09 |
| *Calponia harrisonfordi* | Chelicerata: Araneae | 712 | 125 | 20 | 209 | 80.39 |
| *Segestria sp.* | Chelicerata: Araneae | 651 | 193 | 126 | 96 | 90.99 |
| *Maoriata sp.* | Chelicerata: Araneae | 446 | 189 | 376 | 55 | 94.84 |
| *Orsolobidae sp.* | Chelicerata: Araneae | 553 | 232 | 220 | 61 | 94.28 |
| *Dysdera crocata* | Chelicerata: Araneae | 678 | 246 | 33 | 109 | 89.77 |
| *Ischnothyreus sp.* | Chelicerata: Araneae | 210 | 344 | 131 | 381 | 64.26 |
| *Opopaea sp.* | Chelicerata: Araneae | 632 | 192 | 187 | 55 | 94.84 |
| *Diguetia sp.* | Chelicerata: Araneae | 499 | 265 | 54 | 248 | 76.74 |
| *Pholcus manueli* | Chelicerata: Araneae | 807 | 173 | 20 | 66 | 93.81 |
| *Pholcus phalangioides* | Chelicerata: Araneae | 821 | 132 | 39 | 74 | 93.06 |
| *Loxosceles deserta* | Chelicerata: Araneae | 857 | 95 | 49 | 65 | 93.90 |
| *Drymusa sp.* | Chelicerata: Araneae | 411 | 128 | 482 | 45 | 95.78 |
| *Periegops suterii* | Chelicerata: Araneae | 478 | 304 | 144 | 140 | 86.87 |
| *Ochyrocera sp.* | Chelicerata: Araneae | 404 | 157 | 447 | 58 | 94.56 |
| *Scytodes globula* | Chelicerata: Araneae | 186 | 379 | 385 | 116 | 89.12 |
| *Scytodes thoracica* | Chelicerata: Araneae | 841 | 61 | 123 | 41 | 96.15 |
| *Archoleptoneta sp.* | Chelicerata: Araneae | 627 | 139 | 269 | 31 | 97.09 |
| *Calileptoneta californica* | Chelicerata: Araneae | 801 | 140 | 29 | 96 | 90.99 |
| *Austrochilus forsteri* | Chelicerata: Araneae | 690 | 114 | 218 | 44 | 95.87 |
| *Hickmania troglodytes* | Chelicerata: Araneae | 518 | 334 | 18 | 196 | 81.61 |
| *Progradungula otwayensis* | Chelicerata: Araneae | 165 | 340 | 38 | 523 | 50.94 |
| *Tarlina sp.* | Chelicerata: Araneae | 690 | 113 | 220 | 43 | 95.97 |
| *Gradungula sorenseni* | Chelicerata: Araneae | 559 | 205 | 253 | 49 | 95.40 |
| *Pianoa isolata* | Chelicerata: Araneae | 374 | 406 | 162 | 124 | 88.37 |
| *Austrarchaea sp.* | Chelicerata: Araneae | 103 | 291 | 32 | 640 | 39.96 |
| *Mecysmauchenius sp.* | Chelicerata: Araneae | 490 | 333 | 11 | 232 | 78.24 |
| *Huttonia palpimanoides* | Chelicerata: Araneae | 392 | 401 | 127 | 146 | 86.30 |
| *Otiothops birabeni* | Chelicerata: Araneae | 383 | 365 | 196 | 122 | 88.56 |
| *Palpimanus gibbulus* | Chelicerata: Araneae | 582 | 266 | 119 | 99 | 90.71 |
| *Stegodyphus mimosarum* | Chelicerata: Araneae | 636 | 100 | 23 | 307 | 71.20 |
| *Megadictyna thilenii* | Chelicerata: Araneae | 500 | 269 | 216 | 81 | 92.40 |
| *Nicodamidae sp2* | Chelicerata: Araneae | 664 | 214 | 131 | 57 | 94.65 |
| *Nicodamidae sp1* | Chelicerata: Araneae | 580 | 193 | 232 | 61 | 94.28 |
| *Mysmenella jobi* | Chelicerata: Araneae | 658 | 176 | 179 | 53 | 95.03 |
| *Novanapis sp.* | Chelicerata: Araneae | 516 | 205 | 305 | 40 | 96.25 |
| *Anelosimus eximius* | Chelicerata: Araneae | 222 | 195 | 596 | 53 | 95.03 |
| *Latrodectus tredecimguttatus* | Chelicerata: Araneae | 623 | 178 | 101 | 164 | 84.62 |
| *Euryopis sp.* | Chelicerata: Araneae | 283 | 320 | 234 | 229 | 78.52 |
| *Parasteatoda tepidariorum* | Chelicerata: Araneae | 738 | 5 | 315 | 8 | 99.25 |
| *Pararchaea alba* | Chelicerata: Araneae | 414 | 298 | 291 | 63 | 94.09 |
| *Malkara sp.* | Chelicerata: Araneae | 871 | 120 | 27 | 48 | 95.50 |

| | | | | | | |
|---|---|---|---|---|---|---|
| *Malkaridae sp.* | Chelicerata: Araneae | 338 | 230 | 410 | 88 | 91.74 |
| *Perissopmeros sp.* | Chelicerata: Araneae | 520 | 293 | 106 | 147 | 86.21 |
| *Australomimetus clean* | Chelicerata: Araneae | 766 | 199 | 28 | 73 | 93.15 |
| *Ero leonina* | Chelicerata: Araneae | 707 | 47 | 281 | 31 | 97.09 |
| *Arkys sp.* | Chelicerata: Araneae | 423 | 228 | 323 | 92 | 91.37 |
| *Demadiana sp.* | Chelicerata: Araneae | 338 | 222 | 432 | 74 | 93.06 |
| *Leucauge venusta* | Chelicerata: Araneae | 436 | 68 | 518 | 44 | 95.87 |
| *Meta ovalis* | Chelicerata: Araneae | 812 | 138 | 27 | 89 | 91.65 |
| *Nanometa sp.* | Chelicerata: Araneae | 403 | 351 | 81 | 231 | 78.33 |
| *Tetragnatha tantalus* | Chelicerata: Araneae | 390 | 188 | 49 | 439 | 58.82 |
| *Cepheia longiseta* | Chelicerata: Araneae | 509 | 347 | 16 | 194 | 81.80 |
| *Mysmena leichardti* | Chelicerata: Araneae | 756 | 102 | 169 | 39 | 96.34 |
| *Microdipoena guttata* | Chelicerata: Araneae | 799 | 125 | 16 | 126 | 88.18 |
| *Spinanapis sp.* | Chelicerata: Araneae | 726 | 109 | 195 | 36 | 96.62 |
| *Tekelloides sp2* | Chelicerata: Araneae | 583 | 144 | 307 | 32 | 97.00 |
| *Laminacauda parvipalpis* | Chelicerata: Araneae | 770 | 180 | 32 | 84 | 92.12 |
| *Frontinella communis* | Chelicerata: Araneae | 542 | 75 | 401 | 48 | 95.50 |
| *Novafroneta gladiatrix* | Chelicerata: Araneae | 638 | 230 | 131 | 67 | 93.71 |
| *Synotaxus cf turbinatus* | Chelicerata: Araneae | 458 | 174 | 368 | 66 | 93.81 |
| *Nesticus bishopi* | Chelicerata: Araneae | 879 | 74 | 50 | 63 | 94.09 |
| *Nesticus cooperi* | Chelicerata: Araneae | 113 | 124 | 143 | 686 | 35.65 |
| *Meringa sp.* | Chelicerata: Araneae | 414 | 336 | 204 | 112 | 89.49 |
| *Runga sp.* | Chelicerata: Araneae | 611 | 219 | 181 | 55 | 94.84 |
| *Physoglenes sp.* | Chelicerata: Araneae | 254 | 192 | 558 | 62 | 94.18 |
| *Physoglenidae sp.* | Chelicerata: Araneae | 537 | 311 | 15 | 203 | 80.96 |
| *Ogulnius sp.* | Chelicerata: Araneae | 417 | 254 | 318 | 77 | 92.78 |
| *Baalzebub sp.* | Chelicerata: Araneae | 198 | 410 | 272 | 186 | 82.55 |
| *Theridiosoma gemmosum* | Chelicerata: Araneae | 407 | 277 | 278 | 104 | 90.24 |
| *Theridiosoma savannum* | Chelicerata: Araneae | 547 | 158 | 314 | 47 | 95.59 |
| *Trichonephila clavipes* | Chelicerata: Araneae | 571 | 110 | 57 | 328 | 69.23 |
| *Micrathena gracilis* | Chelicerata: Araneae | 199 | 197 | 370 | 300 | 71.86 |
| *Verrucosa arenata* | Chelicerata: Araneae | 663 | 200 | 98 | 105 | 90.15 |
| *Cyrtophora sp.* | Chelicerata: Araneae | 439 | 206 | 346 | 75 | 92.96 |
| *Neoscona arabesca* | Chelicerata: Araneae | 472 | 135 | 378 | 81 | 92.40 |
| *Gasteracantha hasselti* | Chelicerata: Araneae | 849 | 45 | 141 | 31 | 97.09 |
| *Macracantha arcuata* | Chelicerata: Araneae | 853 | 71 | 82 | 60 | 94.37 |
| *Menneus sp.* | Chelicerata: Araneae | 527 | 262 | 161 | 116 | 89.12 |
| *Deinopis sp.* | Chelicerata: Araneae | 296 | 395 | 5 | 370 | 65.29 |
| *Deinopis longipes* | Chelicerata: Araneae | 138 | 130 | 252 | 546 | 48.78 |
| *Tamopsis sp.* | Chelicerata: Araneae | 552 | 258 | 160 | 96 | 90.99 |
| *Oecobius navus* | Chelicerata: Araneae | 796 | 160 | 32 | 78 | 92.68 |
| *Oecobius cellariorum* | Chelicerata: Araneae | 900 | 87 | 30 | 49 | 95.40 |
| *Cybaeodamus taim* | Chelicerata: Araneae | 264 | 369 | 76 | 357 | 66.51 |
| *Forsterella sp.* | Chelicerata: Araneae | 464 | 318 | 205 | 79 | 92.59 |
| *Caayguara ybytyriguara* | Chelicerata: Araneae | 292 | 376 | 7 | 391 | 63.32 |
| *Amaurobius ferox* | Chelicerata: Araneae | 462 | 316 | 15 | 273 | 74.39 |
| *Callobius sp.* | Chelicerata: Araneae | 533 | 236 | 64 | 233 | 78.14 |
| *Neoramia sp.* | Chelicerata: Araneae | 496 | 287 | 123 | 160 | 84.99 |
| *Amphinecta sp.* | Chelicerata: Araneae | 437 | 269 | 259 | 101 | 90.53 |

| | | | | | | |
|---|---|---|---|---|---|---|
| *Metaltella simoni* | Chelicerata: Araneae | 558 | 228 | 40 | 240 | 77.49 |
| *Cambridgea sp.* | Chelicerata: Araneae | 461 | 261 | 258 | 86 | 91.93 |
| *Paramatachia sp.* | Chelicerata: Araneae | 589 | 237 | 172 | 68 | 93.62 |
| *Agelenopsis emertoni* | Chelicerata: Araneae | 740 | 117 | 29 | 180 | 83.11 |
| *Agelenopsis pennsylvanica* | Chelicerata: Araneae | 741 | 212 | 31 | 82 | 92.31 |
| *Calymmaria sp.* | Chelicerata: Araneae | 625 | 286 | 23 | 132 | 87.62 |
| *Calymmaria persica* | Chelicerata: Araneae | 613 | 234 | 80 | 139 | 86.96 |
| *Cicurina travisae* | Chelicerata: Araneae | 619 | 79 | 191 | 177 | 83.40 |
| *Cicurina vibora* | Chelicerata: Araneae | 508 | 120 | 169 | 269 | 74.77 |
| *Homalonychus theologus* | Chelicerata: Araneae | 544 | 209 | 200 | 113 | 89.40 |
| *Tengella radiata* | Chelicerata: Araneae | 545 | 58 | 433 | 30 | 97.19 |
| *Uliodon sp.* | Chelicerata: Araneae | 619 | 124 | 275 | 48 | 95.50 |
| *Misumenoides formosipes* | Chelicerata: Araneae | 270 | 248 | 17 | 531 | 50.19 |
| *Sidymella sp.* | Chelicerata: Araneae | 497 | 244 | 245 | 80 | 92.50 |
| *Anahita punctulata* | Chelicerata: Araneae | 714 | 162 | 99 | 91 | 91.46 |
| *Peucetia longipalpis* | Chelicerata: Araneae | 729 | 134 | 26 | 177 | 83.40 |
| *Dolomedes triton* | Chelicerata: Araneae | 488 | 277 | 85 | 216 | 79.74 |
| *Pisaurina mira* | Chelicerata: Araneae | 416 | 322 | 13 | 315 | 70.45 |
| *Cupiennius sp.* | Chelicerata: Araneae | 363 | 337 | 157 | 209 | 80.39 |
| *Allocosa alticeps* | Chelicerata: Araneae | 495 | 256 | 255 | 60 | 94.37 |
| *Schizocosa rovneri* | Chelicerata: Araneae | 862 | 85 | 54 | 65 | 93.90 |
| *Habronattus signatus* | Chelicerata: Araneae | 782 | 141 | 41 | 102 | 90.43 |
| *Habronattus ustulatus* | Chelicerata: Araneae | 883 | 93 | 41 | 49 | 95.40 |
| *Teminius sp.* | Chelicerata: Araneae | 439 | 329 | 190 | 108 | 89.87 |
| *Karaops raveni* | Chelicerata: Araneae | 321 | 329 | 234 | 182 | 82.93 |
| *Falconina gracilis* | Chelicerata: Araneae | 132 | 443 | 74 | 417 | 60.88 |
| *Nyssus sp.* | Chelicerata: Araneae | 522 | 262 | 193 | 89 | 91.65 |
| *Clubiona sp.* | Chelicerata: Araneae | 579 | 307 | 19 | 161 | 84.90 |
| *Rebilus sp.* | Chelicerata: Araneae | 571 | 251 | 142 | 102 | 90.43 |
| *Liocranidae sp.* | Chelicerata: Araneae | 780 | 172 | 27 | 87 | 91.84 |
| *Trachelas tranquillus* | Chelicerata: Araneae | 545 | 331 | 10 | 180 | 83.11 |
| *Sergiolus capulatus* | Chelicerata: Araneae | 754 | 161 | 39 | 112 | 89.49 |
| *Molycria sp.* | Chelicerata: Araneae | 453 | 314 | 143 | 156 | 85.37 |
| *Anzacia sp.* | Chelicerata: Araneae | 372 | 263 | 325 | 106 | 90.06 |
| *Lampona sp.* | Chelicerata: Araneae | 408 | 254 | 293 | 111 | 89.59 |
| *Zosis sp.* | Chelicerata: Araneae | 650 | 180 | 171 | 65 | 93.90 |
| *Philoponella herediae* | Chelicerata: Araneae | 136 | 147 | 155 | 628 | 41.09 |
| *Uloborus glomosus* | Chelicerata: Araneae | 608 | 232 | 22 | 204 | 80.86 |
| *Leptopsalis sp.* | Chelicerata: Opiliones | 723 | 126 | 161 | 56 | 94.75 |
| *Miopsalis sp.* | Chelicerata: Opiliones | 641 | 245 | 14 | 166 | 84.43 |
| *Parapurcellia silvicola* | Chelicerata: Opiliones | 894 | 75 | 17 | 80 | 92.50 |
| *Purcellia illustrans* | Chelicerata: Opiliones | 808 | 145 | 21 | 92 | 91.37 |
| *Chileogovea oedipus* | Chelicerata: Opiliones | 901 | 88 | 9 | 68 | 93.62 |
| *Rakaia minutissima* | Chelicerata: Opiliones | 864 | 106 | 13 | 83 | 92.21 |
| *Rakaia stewartiensis* | Chelicerata: Opiliones | 743 | 165 | 12 | 146 | 86.30 |
| *Rakaia magna australis* | Chelicerata: Opiliones | 610 | 290 | 10 | 156 | 85.37 |
| *Rakaia pauli IZ144621* | Chelicerata: Opiliones | 825 | 152 | 25 | 64 | 94.00 |
| *Rakaia pauli reassembled* | Chelicerata: Opiliones | 663 | 153 | 205 | 45 | 95.78 |
| *Pettalus thwaitesi* | Chelicerata: Opiliones | 183 | 264 | 4 | 615 | 42.31 |

| | | | | | | |
|---|---|---|---|---|---|---|
| *Austropurcellia alata IZ14141* | Chelicerata: Opiliones | 535 | 296 | 9 | 226 | 78.80 |
| *Austropurcellia despectata* | Chelicerata: Opiliones | 972 | 29 | 16 | 49 | 95.40 |
| *Austropurcellia cf acuta* | Chelicerata: Opiliones | 679 | 207 | 15 | 165 | 84.52 |
| *Austropurcellia acuta* | Chelicerata: Opiliones | 812 | 164 | 15 | 75 | 92.96 |
| *Austropurcellia clousei* | Chelicerata: Opiliones | 922 | 70 | 24 | 50 | 95.31 |
| *Neopurcellia salmoni IZ144625* | Chelicerata: Opiliones | 820 | 159 | 17 | 70 | 93.43 |
| *Neopurcellia salmoni IZ29310* | Chelicerata: Opiliones | 858 | 121 | 19 | 68 | 93.62 |
| *Karripurcellia peckorum* | Chelicerata: Opiliones | 805 | 155 | 14 | 92 | 91.37 |
| *Aoraki denticulata Northern* | Chelicerata: Opiliones | 943 | 49 | 16 | 58 | 94.56 |
| *Aoraki denticulata Southern* | Chelicerata: Opiliones | 917 | 63 | 17 | 69 | 93.53 |
| *Aoraki denticulata Southern reassembled* | Chelicerata: Opiliones | 696 | 134 | 184 | 52 | 95.12 |
| *Aoraki longitarsa* | Chelicerata: Opiliones | 548 | 310 | 8 | 200 | 81.24 |
| *Siro boyerae* | Chelicerata: Opiliones | 694 | 55 | 204 | 113 | 89.40 |
| *Metagovea oviformis* | Chelicerata: Opiliones | 779 | 207 | 11 | 69 | 93.53 |
| *Metasiro savannahensis* | Chelicerata: Opiliones | 560 | 214 | 217 | 75 | 92.96 |
| *Brasilogovea microphaga* | Chelicerata: Opiliones | 800 | 174 | 8 | 84 | 92.12 |
| *Neogovea matawai* | Chelicerata: Opiliones | 368 | 316 | 155 | 227 | 78.71 |
| *Acropsopilio neozealandiae* | Chelicerata: Opiliones | 942 | 55 | 9 | 60 | 94.37 |
| *Hesperonemastoma modestum* | Chelicerata: Opiliones | 706 | 33 | 288 | 39 | 96.34 |
| *Ortholasma coronadense* | Chelicerata: Opiliones | 654 | 23 | 268 | 121 | 88.65 |
| *Trogulus martensi* | Chelicerata: Opiliones | 764 | 34 | 209 | 59 | 94.47 |
| *Caddo agilis* | Chelicerata: Opiliones | 272 | 328 | 313 | 153 | 85.65 |
| *Thrasychirus modestus* | Chelicerata: Opiliones | 721 | 148 | 130 | 67 | 93.71 |
| *Forsteropsalis pureora* | Chelicerata: Opiliones | 645 | 252 | 7 | 162 | 84.80 |
| *Pantopsalis cheliferoides* | Chelicerata: Opiliones | 825 | 13 | 194 | 34 | 96.81 |
| *Mangatangi parvum* | Chelicerata: Opiliones | 664 | 153 | 169 | 80 | 92.50 |
| *Megalopsalis triascuta* | Chelicerata: Opiliones | 440 | 277 | 86 | 263 | 75.33 |
| *Diguetinus raptator* | Chelicerata: Opiliones | 146 | 181 | 13 | 726 | 31.89 |
| *Phalangium opilio* | Chelicerata: Opiliones | 403 | 247 | 229 | 187 | 82.46 |
| *Protolophus singularis* | Chelicerata: Opiliones | 441 | 120 | 260 | 245 | 77.02 |
| *Prionostemma vittatum* | Chelicerata: Opiliones | 873 | 14 | 155 | 24 | 97.75 |
| *Leiobunum verrucosum* | Chelicerata: Opiliones | 726 | 19 | 252 | 69 | 93.53 |
| *Paranelima sp.* | Chelicerata: Opiliones | 660 | 165 | 173 | 68 | 93.62 |
| *Sclerobunus robustus* | Chelicerata: Opiliones | 674 | 23 | 217 | 152 | 85.74 |
| *Synthetonychia glacialis* | Chelicerata: Opiliones | 693 | 247 | 12 | 114 | 89.31 |
| *Larifuga capensis* | Chelicerata: Opiliones | 284 | 204 | 171 | 407 | 61.82 |
| *Nuncia sp.* | Chelicerata: Opiliones | 865 | 16 | 156 | 29 | 97.28 |
| *Pristobunus sp.* | Chelicerata: Opiliones | 215 | 345 | 24 | 482 | 54.78 |
| *Scotolemon lespesi* | Chelicerata: Opiliones | 654 | 221 | 129 | 62 | 94.18 |
| *Sitalcina lobata* | Chelicerata: Opiliones | 746 | 28 | 224 | 68 | 93.62 |
| *Gnomulus rostratus* | Chelicerata: Opiliones | 806 | 77 | 115 | 68 | 93.62 |
| *Dibunus sp.* | Chelicerata: Opiliones | 641 | 202 | 131 | 92 | 91.37 |
| *Epedanus pinangensis* | Chelicerata: Opiliones | 598 | 223 | 86 | 159 | 85.08 |
| *Haasus judaeus* | Chelicerata: Opiliones | 619 | 143 | 171 | 133 | 87.52 |
| *Haasus naasane* | Chelicerata: Opiliones | 689 | 60 | 260 | 57 | 94.65 |
| *Dampetrus sp.* | Chelicerata: Opiliones | 838 | 93 | 89 | 46 | 95.68 |
| *Metabiantes sp.* | Chelicerata: Opiliones | 662 | 33 | 314 | 57 | 94.65 |

| | | | | | | |
|---|---|---|---|---|---|---|
| *Stygnomma sp.* | Chelicerata: Opiliones | 881 | 35 | 113 | 37 | 96.53 |
| *Zalmoxidae gen. sp.* | Chelicerata: Opiliones | 768 | 99 | 109 | 90 | 91.56 |
| *Kimulidae sp.* | Chelicerata: Opiliones | 758 | 94 | 78 | 136 | 87.24 |
| *Tegipiolus pachypus* | Chelicerata: Opiliones | 583 | 216 | 50 | 217 | 79.64 |
| *Pellobunus sp.* | Chelicerata: Opiliones | 865 | 70 | 92 | 39 | 96.34 |
| *Pachylicus acutus* | Chelicerata: Opiliones | 499 | 196 | 163 | 208 | 80.49 |
| *Maracaynatum trinidaense* | Chelicerata: Opiliones | 878 | 39 | 109 | 40 | 96.25 |
| *Avima matintaperera* | Chelicerata: Opiliones | 216 | 311 | 11 | 528 | 50.47 |
| *Karos barbarikos* | Chelicerata: Opiliones | 671 | 154 | 161 | 80 | 92.50 |
| *Chapulobunus sp.* | Chelicerata: Opiliones | 437 | 333 | 87 | 209 | 80.39 |
| *Stygnoplus clavotibialis* | Chelicerata: Opiliones | 744 | 86 | 130 | 106 | 90.06 |
| *Protimesius longipalpis* | Chelicerata: Opiliones | 758 | 117 | 135 | 56 | 94.75 |
| *Auranus hoeferscovitorum* | Chelicerata: Opiliones | 758 | 99 | 149 | 60 | 94.37 |
| *Pickeliana pickeli* | Chelicerata: Opiliones | 572 | 245 | 83 | 166 | 84.43 |
| *Gonycranaus pluto* | Chelicerata: Opiliones | 218 | 247 | 26 | 575 | 46.06 |
| *Camarana flavipalpi* | Chelicerata: Opiliones | 771 | 103 | 141 | 51 | 95.22 |
| *Pseudopachylus longipes* | Chelicerata: Opiliones | 318 | 325 | 38 | 385 | 63.88 |
| *Bissula sp.* | Chelicerata: Opiliones | 594 | 241 | 81 | 150 | 85.93 |
| *Pseudopachylus eximius* | Chelicerata: Opiliones | 512 | 284 | 87 | 183 | 82.83 |
| *Vonones ornata* | Chelicerata: Opiliones | 633 | 27 | 366 | 40 | 96.25 |
| *Incasarcus argenteus* | Chelicerata: Opiliones | 466 | 287 | 59 | 254 | 76.17 |
| *Quindina limbata* | Chelicerata: Opiliones | 267 | 277 | 14 | 508 | 52.35 |
| *Saramacia lucasae* | Chelicerata: Opiliones | 752 | 103 | 149 | 62 | 94.18 |
| *Ampycinae sp.* | Chelicerata: Opiliones | 693 | 156 | 144 | 73 | 93.15 |
| *Glysterus sp.* | Chelicerata: Opiliones | 421 | 348 | 78 | 219 | 79.46 |
| *Panalus robustus* | Chelicerata: Opiliones | 465 | 284 | 54 | 263 | 75.33 |
| *Phalangodus cottus* | Chelicerata: Opiliones | 217 | 257 | 32 | 560 | 47.47 |
| *Santinezia serratotibialis* | Chelicerata: Opiliones | 779 | 43 | 167 | 77 | 92.78 |
| *Phareicranaus manauara* | Chelicerata: Opiliones | 646 | 180 | 151 | 89 | 91.65 |
| *Pseudopucrolia discrepans* | Chelicerata: Opiliones | 681 | 163 | 105 | 117 | 89.02 |
| *Sadocus polyacanthus* | Chelicerata: Opiliones | 629 | 205 | 149 | 83 | 92.21 |
| *Chilegyndes phillipsoni* | Chelicerata: Opiliones | 521 | 286 | 75 | 184 | 82.74 |
| *Metagyndes innata* | Chelicerata: Opiliones | 256 | 212 | 24 | 574 | 46.15 |
| *Hernandaria una* | Chelicerata: Opiliones | 581 | 233 | 79 | 173 | 83.77 |
| *Graphinotus therezopolis* | Chelicerata: Opiliones | 497 | 230 | 52 | 287 | 73.08 |
| *Meteusarcoides sp IZ139264* | Chelicerata: Opiliones | 661 | 179 | 153 | 73 | 93.15 |
| *Meteusarcoides caudatus* | Chelicerata: Opiliones | 83 | 148 | 3 | 832 | 21.95 |
| *Acutisoma longipes* | Chelicerata: Opiliones | 555 | 268 | 75 | 168 | 84.24 |
| *Goniosoma sp.* | Chelicerata: Opiliones | 690 | 172 | 119 | 85 | 92.03 |
| *Roeweria virescens* | Chelicerata: Opiliones | 581 | 246 | 98 | 141 | 86.77 |
| *Longiperna concolor* | Chelicerata: Opiliones | 452 | 317 | 57 | 240 | 77.49 |
| *Gonyleptidae sp IZ139272* | Chelicerata: Opiliones | 519 | 285 | 83 | 179 | 83.21 |
| *Mitobates conspersus* | Chelicerata: Opiliones | 434 | 327 | 64 | 241 | 77.39 |
| *Promitobates ornatus* | Chelicerata: Opiliones | 603 | 222 | 86 | 155 | 85.46 |
| *Gonyleptellus pustulosus* | Chelicerata: Opiliones | 520 | 280 | 80 | 186 | 82.55 |
| *Gonyleptes fragilis* | Chelicerata: Opiliones | 566 | 204 | 108 | 188 | 82.36 |
| *Pseudotrogulus mirim* | Chelicerata: Opiliones | 647 | 205 | 100 | 114 | 89.31 |
| *Pseudotrogulus trostkyi* | Chelicerata: Opiliones | 706 | 142 | 143 | 75 | 92.96 |
| *Ampheres leucopheus* | Chelicerata: Opiliones | 606 | 227 | 84 | 149 | 86.02 |

| | | | | | | |
|---|---|---|---|---|---|---|
| *Acanthogonyleptes aff fulvigranulatus* | Chelicerata: Opiliones | 547 | 240 | 69 | 210 | 80.30 |
| *Gonyleptes atrus* | Chelicerata: Opiliones | 513 | 256 | 94 | 203 | 80.96 |
| *Gonyleptes sp.* | Chelicerata: Opiliones | 177 | 288 | 16 | 585 | 45.12 |
| *Geraecormobius bispinifrons* | Chelicerata: Opiliones | 523 | 221 | 73 | 249 | 76.64 |
| *Mischonyx cuspidatus* | Chelicerata: Opiliones | 750 | 135 | 121 | 60 | 94.37 |
| *Sodreana leprevosti* | Chelicerata: Opiliones | 664 | 192 | 107 | 103 | 90.34 |
| *Sodreana sodrena* | Chelicerata: Opiliones | 541 | 251 | 74 | 200 | 81.24 |
| *Progonyleptoidellus striatus* | Chelicerata: Opiliones | 584 | 218 | 83 | 181 | 83.02 |
| *Iporangaia pustulosa* | Chelicerata: Opiliones | 731 | 121 | 119 | 95 | 91.09 |
| *Gonyleptoides marumbiensis* | Chelicerata: Opiliones | 502 | 282 | 87 | 195 | 81.71 |
| *Neosadocus maximus* | Chelicerata: Opiliones | 490 | 296 | 66 | 214 | 79.92 |
| *Pseudotyrannochthonius sp. B14401* | Chelicerata: Pseudoscorpiones | 260 | 6 | 789 | 11 | 98.97 |
| *Pseudotyrannochthonius sp. MCZ* | Chelicerata: Pseudoscorpiones | 613 | 8 | 415 | 30 | 97.19 |
| *Chthoniidae sp.* | Chelicerata: Pseudoscorpiones | 160 | 248 | 31 | 627 | 41.18 |
| *Lagynochthonius australicus* | Chelicerata: Pseudoscorpiones | 663 | 32 | 346 | 25 | 97.65 |
| *Ephippiochthonius tetrachelatus* | Chelicerata: Pseudoscorpiones | 593 | 213 | 166 | 94 | 91.18 |
| *Lechytia hoffi* | Chelicerata: Pseudoscorpiones | 541 | 198 | 235 | 92 | 91.37 |
| *Feaella capensis* | Chelicerata: Pseudoscorpiones | 572 | 208 | 202 | 84 | 92.12 |
| *Pseudogarypus banksi* | Chelicerata: Pseudoscorpiones | 230 | 321 | 40 | 475 | 55.44 |
| *Bochica withi* | Chelicerata: Pseudoscorpiones | 751 | 34 | 220 | 61 | 94.28 |
| *Dhanus sumatranus* | Chelicerata: Pseudoscorpiones | 177 | 268 | 162 | 459 | 56.94 |
| *Indohya sp.* | Chelicerata: Pseudoscorpiones | 88 | 142 | 4 | 832 | 21.95 |
| *Ideobisium crassimanum* | Chelicerata: Pseudoscorpiones | 740 | 43 | 250 | 33 | 96.90 |
| *Parahya submersa* | Chelicerata: Pseudoscorpiones | 605 | 169 | 227 | 65 | 93.90 |
| *Gymnobisium sp.* | Chelicerata: Pseudoscorpiones | 592 | 144 | 266 | 64 | 94.00 |
| *Microcreagrinae sp.* | Chelicerata: Pseudoscorpiones | 374 | 209 | 369 | 114 | 89.31 |
| *Microbisium brunneum* | Chelicerata: Pseudoscorpiones | 401 | 341 | 94 | 230 | 78.42 |
| *Microbisium sp.* | Chelicerata: Pseudoscorpiones | 416 | 187 | 350 | 113 | 89.40 |
| *Novobisium sp.* | Chelicerata: Pseudoscorpiones | 354 | 263 | 223 | 226 | 78.80 |
| *Afrogarypus subimpressus* | Chelicerata: Pseudoscorpiones | 567 | 207 | 189 | 103 | 90.34 |
| *Geogarypus maculatus* | Chelicerata: Pseudoscorpiones | 688 | 25 | 319 | 34 | 96.81 |
| *Apolpium sp.* | Chelicerata: Pseudoscorpiones | 734 | 73 | 182 | 77 | 92.78 |
| *Anchigarypus californicus* | Chelicerata: Pseudoscorpiones | 656 | 21 | 358 | 31 | 97.09 |
| *Synsphyronus apimelus* | Chelicerata: Pseudoscorpiones | 581 | 158 | 220 | 107 | 89.96 |
| *Pseudogarypinus frontalis* | Chelicerata: Pseudoscorpiones | 434 | 279 | 131 | 222 | 79.17 |
| *Larca granulata* | Chelicerata: Pseudoscorpiones | 249 | 291 | 58 | 468 | 56.10 |

| | | | | | | |
|---|---|---|---|---|---|---|
| *Protogarypinus giganteus* | Chelicerata: Pseudoscorpiones | 284 | 284 | 42 | 456 | 57.22 |
| *Cheiridiidae sp.* | Chelicerata: Pseudoscorpiones | 511 | 270 | 102 | 183 | 82.83 |
| *Sternophoridae sp.* | Chelicerata: Pseudoscorpiones | 471 | 124 | 374 | 97 | 90.90 |
| *Cacodemonius segmentidentatus* | Chelicerata: Pseudoscorpiones | 633 | 19 | 392 | 22 | 97.94 |
| *Oratemnus curtus* | Chelicerata: Pseudoscorpiones | 280 | 165 | 547 | 74 | 93.06 |
| *Protochelifer sp.* | Chelicerata: Pseudoscorpiones | 274 | 150 | 556 | 86 | 91.93 |
| *Cheliferidae sp.* | Chelicerata: Pseudoscorpiones | 385 | 274 | 167 | 240 | 77.49 |
| *Parachelifer persimilis* | Chelicerata: Pseudoscorpiones | 292 | 319 | 86 | 369 | 65.38 |
| *Lamprochernes savignyi* | Chelicerata: Pseudoscorpiones | 611 | 22 | 379 | 54 | 94.93 |
| *Conicochernes crassus* | Chelicerata: Pseudoscorpiones | 259 | 303 | 144 | 360 | 66.23 |
| *Hesperochernes sp.* | Chelicerata: Pseudoscorpiones | 521 | 186 | 157 | 202 | 81.05 |
| *Haplochernes kraepelini* | Chelicerata: Pseudoscorpiones | 242 | 347 | 271 | 206 | 80.68 |
| *Chernetidae sp. MCZ* | Chelicerata: Pseudoscorpiones | 416 | 219 | 261 | 170 | 84.05 |
| *Chernetidae sp. JC032* | Chelicerata: Pseudoscorpiones | 400 | 271 | 145 | 250 | 76.55 |
| *Chaerilus celebensis* | Chelicerata: Scorpiones | 801 | 148 | 56 | 61 | 94.28 |
| *Lychas variatus* | Chelicerata: Scorpiones | 622 | 144 | 258 | 42 | 96.06 |
| *Mesobuthus martensii* | Chelicerata: Scorpiones | 89 | 63 | 7 | 907 | 14.92 |
| *Compsobuthus levyi* | Chelicerata: Scorpiones | 596 | 161 | 264 | 45 | 95.78 |
| *Birulatus israelensis* | Chelicerata: Scorpiones | 692 | 32 | 328 | 14 | 98.69 |
| *Compsobuthus sp.* | Chelicerata: Scorpiones | 695 | 72 | 274 | 25 | 97.65 |
| *Compsobuthus schmiedeknechti* | Chelicerata: Scorpiones | 621 | 153 | 224 | 68 | 93.62 |
| *Androctonus amoreuxi* | Chelicerata: Scorpiones | 580 | 177 | 241 | 68 | 93.62 |
| *Androctonus australis* | Chelicerata: Scorpiones | 835 | 130 | 41 | 60 | 94.37 |
| *Hottentotta trilineatus* | Chelicerata: Scorpiones | 809 | 148 | 35 | 74 | 93.06 |
| *Orthochirus scrobiculosus* | Chelicerata: Scorpiones | 430 | 292 | 86 | 258 | 75.80 |
| *Androctonus crassicauda* | Chelicerata: Scorpiones | 407 | 299 | 77 | 283 | 73.45 |
| *Buthus israelis* | Chelicerata: Scorpiones | 473 | 250 | 91 | 252 | 76.36 |
| *Buthacus cf arenicola* | Chelicerata: Scorpiones | 667 | 104 | 258 | 37 | 96.53 |
| *Leirus hebraeus* | Chelicerata: Scorpiones | 543 | 238 | 181 | 104 | 90.24 |
| *Leirus quinquestriatus* | Chelicerata: Scorpiones | 679 | 109 | 247 | 31 | 97.09 |
| *Ananteris balzani* | Chelicerata: Scorpiones | 672 | 103 | 266 | 25 | 97.65 |
| *Babycurus gigas* | Chelicerata: Scorpiones | 592 | 227 | 179 | 68 | 93.62 |
| *Grosphus cf grandidiri > Grosphus grandidieri* | Chelicerata: Scorpiones | 611 | 133 | 282 | 40 | 96.25 |

| | | | | | | |
|---|---|---|---|---|---|---|
| *Parabuthus transvaalicus* | Chelicerata: Scorpiones | 785 | 155 | 44 | 82 | 92.31 |
| *Uroplectes olivaceus* | Chelicerata: Scorpiones | 581 | 198 | 219 | 68 | 93.62 |
| *Uropolectes vittatus* | Chelicerata: Scorpiones | 539 | 249 | 199 | 79 | 92.59 |
| *Tityus smithi* | Chelicerata: Scorpiones | 683 | 92 | 269 | 22 | 97.94 |
| *Tityus maranhensis* | Chelicerata: Scorpiones | 697 | 93 | 245 | 31 | 97.09 |
| *Tityus costatus* | Chelicerata: Scorpiones | 670 | 104 | 263 | 29 | 97.28 |
| *Tityus serrulatus* | Chelicerata: Scorpiones | 210 | 81 | 746 | 29 | 97.28 |
| *Troglorhopalurus lacrau* | Chelicerata: Scorpiones | 725 | 72 | 250 | 19 | 98.22 |
| *Physoctonus debilis* | Chelicerata: Scorpiones | 102 | 210 | 22 | 732 | 31.33 |
| *Jaguajir agamemnon* | Chelicerata: Scorpiones | 641 | 195 | 170 | 60 | 94.37 |
| *Jaguajir rochai* | Chelicerata: Scorpiones | 521 | 272 | 114 | 159 | 85.08 |
| *Heteroctenus junceus* | Chelicerata: Scorpiones | 536 | 13 | 499 | 18 | 98.31 |
| *Heteroctenus garridoi* | Chelicerata: Scorpiones | 692 | 40 | 317 | 17 | 98.41 |
| *Centruroides caribensis* | Chelicerata: Scorpiones | 596 | 183 | 238 | 49 | 95.40 |
| *Centruroides hentzi* | Chelicerata: Scorpiones | 212 | 127 | 705 | 22 | 97.94 |
| *Centruroides vittatus* | Chelicerata: Scorpiones | 98 | 106 | 3 | 859 | 19.42 |
| *Centruroides sculpturatus* | Chelicerata: Scorpiones | 496 | 96 | 422 | 52 | 95.12 |
| *Iurus dekanum* | Chelicerata: Scorpiones | 514 | 304 | 26 | 222 | 79.17 |
| *Bothriurus coriaceus* | Chelicerata: Scorpiones | 381 | 266 | 281 | 138 | 87.05 |
| *Bothriurus burmeisteri* | Chelicerata: Scorpiones | 879 | 108 | 43 | 36 | 96.62 |
| *Centromachetes sp.* | Chelicerata: Scorpiones | 321 | 238 | 9 | 498 | 53.28 |
| *Cercophonius squama* | Chelicerata: Scorpiones | 755 | 120 | 34 | 157 | 85.27 |
| *Cercophomius queenslandae* | Chelicerata: Scorpiones | 413 | 158 | 391 | 104 | 90.24 |
| *Cercophonius sulcatus* | Chelicerata: Scorpiones | 407 | 127 | 462 | 70 | 93.43 |
| *Caraboctonus keyserlingii* | Chelicerata: Scorpiones | 507 | 219 | 119 | 221 | 79.27 |
| *Superstitiona donensis* | Chelicerata: Scorpiones | 742 | 194 | 32 | 98 | 90.81 |
| *Anuroctonus phaiodactylus* | Chelicerata: Scorpiones | 799 | 156 | 46 | 65 | 93.90 |
| *Brotheas granulatus* | Chelicerata: Scorpiones | 792 | 152 | 36 | 86 | 91.93 |
| *Scorpiops sp.* | Chelicerata: Scorpiones | 854 | 108 | 37 | 67 | 93.71 |
| *Euscorpius italicus* | Chelicerata: Scorpiones | 709 | 213 | 39 | 105 | 90.15 |
| *Plesiochactas dilutus* | Chelicerata: Scorpiones | 589 | 42 | 401 | 34 | 96.81 |
| *Megacormus gertschi* | Chelicerata: Scorpiones | 780 | 28 | 221 | 37 | 96.53 |
| *Megacormus sp.* | Chelicerata: Scorpiones | 781 | 158 | 37 | 90 | 91.56 |

| | | | | | | |
|---|---|---|---|---|---|---|
| *Uroctonus mordax* | Chelicerata: Scorpiones | 532 | 22 | 492 | 20 | 98.12 |
| *Belisarius xambeui* | Chelicerata: Scorpiones | 661 | 229 | 36 | 140 | 86.87 |
| *Hoffmannihadrurus aztecus* | Chelicerata: Scorpiones | 683 | 166 | 160 | 57 | 94.65 |
| *Hadrurus arizonensis* | Chelicerata: Scorpiones | 657 | 245 | 33 | 131 | 87.71 |
| *Hadrurus concolorous* | Chelicerata: Scorpiones | 754 | 21 | 273 | 18 | 98.31 |
| *Hadrurus spadix* | Chelicerata: Scorpiones | 128 | 16 | 897 | 25 | 97.65 |
| *Uroctonites huachuca* | Chelicerata: Scorpiones | 725 | 38 | 278 | 25 | 97.65 |
| *Kovarikia boggerti* | Chelicerata: Scorpiones | 747 | 42 | 242 | 35 | 96.72 |
| *Pseudouroctonus apacheanus* | Chelicerata: Scorpiones | 720 | 42 | 278 | 26 | 97.56 |
| *Vaejovis cashi* | Chelicerata: Scorpiones | 727 | 34 | 281 | 24 | 97.75 |
| *Paruroctonus baergi* | Chelicerata: Scorpiones | 625 | 19 | 407 | 15 | 98.59 |
| *Smeringurus mesaensis* | Chelicerata: Scorpiones | 675 | 27 | 348 | 16 | 98.50 |
| *Smeringurus vachoni* | Chelicerata: Scorpiones | 648 | 22 | 381 | 15 | 98.59 |
| *Vaejovis mexicanus* | Chelicerata: Scorpiones | 640 | 99 | 262 | 65 | 93.90 |
| *Serradigitus gertschi* | Chelicerata: Scorpiones | 673 | 213 | 103 | 77 | 92.78 |
| *Konetontil acapulco* | Chelicerata: Scorpiones | 597 | 61 | 380 | 28 | 97.37 |
| *Konetontli chamelaensis* | Chelicerata: Scorpiones | 633 | 65 | 330 | 38 | 96.44 |
| *Mesomexovis aff variegatus* | Chelicerata: Scorpiones | 585 | 64 | 372 | 45 | 95.78 |
| *Paravaejovis schwenkmeyeri* | Chelicerata: Scorpiones | 648 | 225 | 115 | 78 | 92.68 |
| *Paravaejovis spinigerus* | Chelicerata: Scorpiones | 559 | 294 | 24 | 189 | 82.27 |
| *Kuarapu purepecha* | Chelicerata: Scorpiones | 634 | 97 | 270 | 65 | 93.90 |
| *Chihuahuanus coahuilae* | Chelicerata: Scorpiones | 649 | 112 | 227 | 78 | 92.68 |
| *Mesovexovis occidentalis* | Chelicerata: Scorpiones | 543 | 202 | 179 | 142 | 86.68 |
| *Thorellius intrepidus* | Chelicerata: Scorpiones | 631 | 141 | 196 | 98 | 90.81 |
| *Urodacus yaschenkoi* | Chelicerata: Scorpiones | 89 | 43 | 914 | 20 | 98.12 |
| *Urodacus elongatus* | Chelicerata: Scorpiones | 552 | 101 | 354 | 59 | 94.47 |
| *Urodacus planimanus* | Chelicerata: Scorpiones | 181 | 66 | 766 | 53 | 95.03 |
| *Hormiops davidovi* | Chelicerata: Scorpiones | 446 | 104 | 482 | 34 | 96.81 |
| *Hormurus weberi* | Chelicerata: Scorpiones | 468 | 147 | 393 | 58 | 94.56 |
| *Liocheles australasiae* | Chelicerata: Scorpiones | 513 | 319 | 42 | 192 | 81.99 |
| *Liocheles sp KhaoDeng* | Chelicerata: Scorpiones | 122 | 301 | 103 | 540 | 49.34 |
| *Nebo hierichonticus* | Chelicerata: Scorpiones | 402 | 140 | 480 | 44 | 95.87 |
| *Diplocentrus diablo* | Chelicerata: Scorpiones | 839 | 130 | 32 | 65 | 93.90 |

| | | | | | | |
|---|---|---|---|---|---|---|
| *Diplocentrus zacatecanus* | Chelicerata: Scorpiones | 376 | 306 | 157 | 227 | 78.71 |
| *Kolotl magnus* | Chelicerata: Scorpiones | 418 | 261 | 218 | 169 | 84.15 |
| *Kolotl poncei* | Chelicerata: Scorpiones | 237 | 305 | 135 | 389 | 63.51 |
| *Iomachus politus* | Chelicerata: Scorpiones | 429 | 210 | 345 | 82 | 92.31 |
| *Hadogenes troglodytes* | Chelicerata: Scorpiones | 760 | 179 | 37 | 90 | 91.56 |
| *Hadogenes paucidens* | Chelicerata: Scorpiones | 386 | 278 | 234 | 168 | 84.24 |
| *Opisthacanthus asper* | Chelicerata: Scorpiones | 388 | 142 | 494 | 42 | 96.06 |
| *Opisthacanthus validus* | Chelicerata: Scorpiones | 244 | 349 | 201 | 272 | 74.48 |
| *Chiromachus ochropus* | Chelicerata: Scorpiones | 378 | 258 | 319 | 111 | 89.59 |
| *Opisthacanthus madagascarensis* | Chelicerata: Scorpiones | 804 | 143 | 39 | 80 | 92.50 |
| *Palaeocheloctonus pauliani* | Chelicerata: Scorpiones | 295 | 325 | 239 | 207 | 80.58 |
| *Opistophthalmus glabrifrons* | Chelicerata: Scorpiones | 485 | 130 | 398 | 53 | 95.03 |
| *Pandinus imperator* | Chelicerata: Scorpiones | 776 | 158 | 44 | 88 | 91.74 |
| *Opistophthalmus walhbergii* | Chelicerata: Scorpiones | 396 | 224 | 331 | 115 | 89.21 |
| *Opistophthalmus boehmi* | Chelicerata: Scorpiones | 121 | 283 | 44 | 618 | 42.03 |
| *Scorpio cf. maurus* | Chelicerata: Scorpiones | 428 | 235 | 142 | 261 | 75.52 |
| *Troglokhammouanus steineri* | Chelicerata: Scorpiones | 875 | 90 | 57 | 44 | 95.87 |
| *Vietbocap lao* | Chelicerata: Scorpiones | 887 | 89 | 39 | 51 | 95.22 |

**Table S3.** Tests of monophyly for Matrices 1-4.

| | | Arachnida | Acari | Panscorpiones | Poecilophysidea | Dromopoda |
|---|---|---|---|---|---|---|
| AU test | Matrix 1 PMSF | 6.09E-07 | 1.91E-09 | 7.48E-08 | 9.95E-74 | 5.65E-06 |
| | Matrix 2 PMSF | 1.69E-04 | 0.000237 | 0.000178 | 0.000311 | 0.000144 |
| | Matrix 3 PMSF | 4.33E-19 | 6.36E-09 | 0.968 | 1.10E-07 | 3.68E-54 |
| | | | | | | |
| Posterior probability | PhyloBayes-mpi | 0.02813567 | 0 | 1 | 1 | 0 |

**Table S4.** Taxonomic groupings used for defining taxon decisiveness.

| *Taxon* | *Number of exemplars* |
|---|---|
| Onychophora | 3 |
| Pancrustacea | 12 |
| Myriapoda | 9 |
| Pycnogonida | 7 |
| Acariformes | 18 |
| Parasitiformes | 14 |
| Palpigradi | 3 |
| Opiliones | 118 |
| Solifugae | 4 |
| Xiphosura | 4 |
| Ricinulei | 7 |
| Pseudoscorpiones | 39 |
| Scorpiones | 103 |
| Amblypygi | 5 |
| Uropygi | 2 |
| Schizomida | 3 |
| Araneae | 155 |

**Table S5.** Chain lengths for Bayesian inference analyses using PhyloBayes-mpi.

| ***chain*** | ***states*** |
|---|---|
| C1 | 20374 |
| C2 | 20181 |
| C3 | 20284 |
| C4 | 20393 |
| C5 | 20820 |
| C6 | 20669 |
| C7 | 20029 |
| C8 | 20244 |

**Table S6.** Summary statistics for Bayesian inference analyses using Phylobayes-mpi, based on all eight chains.

| ***parameter*** | ***mean*** | ***ESS*** |
|---|---|---|
| loglik | -3.101E5 | 206 |
| length | 11.873 | 985 |
| alpha | 0.689 | 162 |
| Nmode | 602.002 | 2794 |
| statent | 1.537 | 172 |
| statalpha | 5.822 | 543 |
| rrent | 4.223 | 405 |
| rrmean | 1.002 | 1.437E5 |

**Table S7.** Detection of paralogs in the 233-locus Matrix A of Lozano *et al.* (2019).

| Locus | Number of sequences with at least one hit | Number of orthogroups detected | Mappings in *D. melanogaster* proteome |
|---|---|---|---|
| ar21 | 84 | 2 | +Drosophila_melanogaster\|G022873\|GNP_650498.3+Drosophila_melanogaster\|G028454\|GNP_732075.3:81 |
| arp23 | 82 | 1 | +Drosophila_melanogaster\|G013712\|GNP_476596.1+Drosophila_melanogaster\|G024891\|GNP_723845.1:82 |
| atpsynthalphaamt | 87 | 1 | +Drosophila_melanogaster\|G026041\|GNP_726243.1:87 |
| cctA | 88 | 2 | +Drosophila_melanogaster\|G015590\|GNP_524450.2+Drosophila_melanogaster\|G028743\|GNP_732748.1:87 |
| cctB | 87 | 2 | +Drosophila_melanogaster\|G016727\|GNP_572524.1:85 |
| cctD | 87 | 2 | +Drosophila_melanogaster\|G018473\|GNP_609579.1:86 |
| cctE | 87 | 3 | +Drosophila_melanogaster\|G014897\|GNP_523707.1+Drosophila_melanogaster\|G025524\|GNP_725107.1:80 |
| cctG | 88 | 1 | +Drosophila_melanogaster\|G004565\|GNP_001189236.1+Drosophila_melanogaster\|G004566\|GNP_001189237.1+Drosophila_melanogaster\|G022932\|GNP_650572.2+Drosophila_melanogaster\|G028499\|GNP_732167.1:88 |
| cctN | 87 | 1 | +Drosophila_melanogaster\|G022361\|GNP_649835.1:87 |
| cctZ | 87 | 1 | +Drosophila_melanogaster\|G017140\|GNP_573066.1:87 |
| cpn60mt | 83 | 3 | +Drosophila_melanogaster\|G014526\|GNP_511115.2+Drosophila_melanogaster\|G015992\|GNP_524925.1+Drosophila_melanogaster\|G017955\|GNP_608948.2+Drosophila_melanogaster\|G024572\|GNP_723104.2+Drosophila_melanogaster\|G024573\|GNP_723105.2+Drosophila_melanogaster\|G026590\|GNP_727489.1:65 |
| crfg | 84 | 1 | +Drosophila_melanogaster\|G011931\|GNP_001286247.1+Drosophila_melanogaster\|G019222\|GNP_610484.1:84 |
| ef1EF1 | 89 | 3 | +Drosophila_melanogaster\|G012005\|GNP_001286321.1+Drosophila_melanogaster\|G012006\|GNP_001286322.1+Drosophila_melanogaster\|G014343\|GNP_477375.1+Drosophila_melanogaster\|G015723\|GNP_524611.1+Drosophila_melanogaster\|G025509\|GNP_725085.1+Drosophila_melanogaster\|G029180\|GNP_733449.1+Drosophila_melanogaster\|G030286\|GNP_996315.1+Drosophila_melanogaster\|G030287\|GNP_996316.1:82 |
| ef1RF3 | 87 | 5 | +Drosophila_melanogaster\|G007742\|GNP_001260415.1+Drosophila_melanogaster\|G007743\|GNP_001260416.1+Drosophila_melanogaster\|G011558\|GNP_001285874.1+Drosophila_melanogaster\|G014251\|GNP_477259.1:67 |
| ef2EF2 | 88 | 2 | +Drosophila_melanogaster\|G016124\|GNP_525105.2+Drosophila_melanogaster\|G023741\|GNP_651605.1+Drosophila_melanogaster\|G025154\|GNP_724357.2+Drosophila_melanogaster\|G025155\|GNP_724358.2:71 |
| eif5a | 82 | 1 | +Drosophila_melanogaster\|G020399\|GNP_611878.1+Drosophila_melanogaster\|G026124\|GNP_726411.1:82 |
| fibri | 76 | 1 | +Drosophila_melanogaster\|G015002\|GNP_523817.1:76 |
| fpps | 74 | 1 | +Drosophila_melanogaster\|G014348\|GNP_477380.1:74 |
| g122 | 79 | 1 | +Drosophila_melanogaster\|G014329\|GNP_477356.1:79 |
| g125 | 20 | 1 | +Drosophila_melanogaster\|G018135\|GNP_609162.1:20 |
| g127 | 69 | 1 | +Drosophila_melanogaster\|G020455\|GNP_611940.1+Drosophila_melanogaster\|G026143\|GNP_726463.1:69 |
| g143 | 79 | 1 | +Drosophila_melanogaster\|G023971\|GNP_651885.1:79 |
| g46 | 77 | 1 | +Drosophila_melanogaster\|G007218\|GNP_001259877.1+Drosophila_melanogaster\|G014046\|GNP_477005.1:77 |
| g51 | 7 | 1 | +Drosophila_melanogaster\|G004466\|GNP_001189124.1+Drosophila_melanogaster\|G004467\|GNP_001189125.1+Drosophila_ |

| | | | |
|---|---|---|---|
| | | | melanogaster\|G009255\|GNP_001261987.1+Drosophila_melanogaster\|G021687\|GNP_648985.1:7 |
| g53 | 10 | 1 | +Drosophila_melanogaster\|G024158\|GNP_652341.1:10 |
| g65 | 73 | 1 | +Drosophila_melanogaster\|G008182\|GNP_001260877.1+Drosophila_melanogaster\|G019335\|GNP_610629.1:73 |
| g7 | 72 | 1 | +Drosophila_melanogaster\|G021580\|GNP_648848.1:72 |
| g70 | 69 | 1 | +Drosophila_melanogaster\|G020884\|GNP_647961.1:69 |
| g78 | 81 | 1 | +Drosophila_melanogaster\|G021751\|GNP_649059.1:81 |
| g82 | 15 | 3 | +Drosophila_melanogaster\|G010811\|GNP_001285120.1+Drosophila_melanogaster\|G016862\|GNP_572713.1:5 |
| grc5 | 86 | 1 | +Drosophila_melanogaster\|G009494\|GNP_001262233.1+Drosophila_melanogaster\|G024018\|GNP_651954.3+Drosophila_melanogaster\|G027922\|GNP_730773.4:86 |
| hsp70E | 88 | 4 | +Drosophila_melanogaster\|G009839\|GNP_001262586.1+Drosophila_melanogaster\|G010830\|GNP_001285139.1+Drosophila_melanogaster\|G012098\|GNP_001286414.1+Drosophila_melanogaster\|G014542\|GNP_511132.2+Drosophila_melanogaster\|G014928\|GNP_523741.2+Drosophila_melanogaster\|G015233\|GNP_524063.1+Drosophila_melanogaster\|G015486\|GNP_524339.1+Drosophila_melanogaster\|G015502\|GNP_524356.1+Drosophila_melanogaster\|G015611\|GNP_524474.1+Drosophila_melanogaster\|G015883\|GNP_524798.2+Drosophila_melanogaster\|G015993\|GNP_524927.2+Drosophila_melanogaster\|G022659\|GNP_650209.1+Drosophila_melanogaster\|G026626\|GNP_727563.1+Drosophila_melanogaster\|G026627\|GNP_727564.1+Drosophila_melanogaster\|G026628\|GNP_727565.1+Drosophila_melanogaster\|G027609\|GNP_729940.1+Drosophila_melanogaster\|G027610\|GNP_729941.1+Drosophila_melanogaster\|G028271\|GNP_731651.1+Drosophila_melanogaster\|G028296\|GNP_731716.1+Drosophila_melanogaster\|G028415\|GNP_731987.1+Drosophila_melanogaster\|G028416\|GNP_731988.1+Drosophila_melanogaster\|G028417\|GNP_731989.1+Drosophila_melanogaster\|G029548\|GNP_788663.1+Drosophila_melanogaster\|G029562\|GNP_788679.1+Drosophila_melanogaster\|G029563\|GNP_788680.1:79 |
| hsp70mt | 88 | 10 | +Drosophila_melanogaster\|G012098\|GNP_001286414.1+Drosophila_melanogaster\|G014928\|GNP_523741.2:15 |
| hsp90C | 83 | 3 | +Drosophila_melanogaster\|G008645\|GNP_001261362.1+Drosophila_melanogaster\|G015079\|GNP_523899.1:81 |
| hsp90E | 87 | 3 | +Drosophila_melanogaster\|G023737\|GNP_651601.1:19 |
| if1a | 83 | 1 | +Drosophila_melanogaster\|G013059\|GNP_001287386.1+Drosophila_melanogaster\|G015820\|GNP_524728.2+Drosophila_melanogaster\|G030219\|GNP_996231.1:83 |
| if2b | 83 | 1 | +Drosophila_melanogaster\|G015215\|GNP_524043.1:83 |
| if2g | 84 | 2 | +Drosophila_melanogaster\|G009840\|GNP_001262587.1+Drosophila_melanogaster\|G028418\|GNP_731993.1+Drosophila_melanogaster\|G028419\|GNP_731994.1:70 |
| if4aa | 88 | 3 | +Drosophila_melanogaster\|G005102\|GNP_001245907.1+Drosophila_melanogaster\|G007455\|GNP_001260117.1+Drosophila_melanogaster\|G013711\|GNP_476595.1+Drosophila_melanogaster\|G022324\|GNP_649788.2+Drosophila_melanogaster\|G024583\|GNP_723137.1+Drosophila_melanogaster\|G024584\|GNP_723138.1+Drosophila_melanogaster\|G024585\|GNP_723139.1:72 |
| if4ab | 86 | 3 | +Drosophila_melanogaster\|G005102\|GNP_001245907.1+Drosophila_melanogaster\|G007455\|GNP_001260117.1+Drosophila_melanogaster\|G013711\|GNP_476595.1+Drosophila_melanogaster\|G022324\|GNP_649788.2+Drosophila_melanogaster\|G024583\|GNP_723137.1+Drosophila_melanogaster\|G024584\|GNP_723138.1+Drosophila_melanogaster\|G024585\|GNP_723139.1:79 |
| if6 | 79 | 1 | +Drosophila_melanogaster\|G024321\|GNP_659573.1:79 |
| l12eA | 84 | 1 | +Drosophila_melanogaster\|G027584\|GNP_729866.1:84 |

| | | | |
|---|---|---|---|
| l12eC | 79 | 1 | +Drosophila_melanogasterlG007624lGNP_001260293.1+Drosophila_melanogasterlG015810lGNP_524714.1:79 |
| l12eD | 84 | 1 | +Drosophila_melanogasterlG010637lGNP_001284944.1+Drosophila_melanogasterlG010638lGNP_001284945.1+Drosophila_melanogasterlG014478lGNP_511063.1+Drosophila_melanogasterlG026444lGNP_727094.1+Drosophila_melanogasterlG026445lGNP_727096.1:84 |
| mcmA | 77 | 9 | +Drosophila_melanogasterlG015455lGNP_524308.2:58 |
| mcmD | 80 | 7 | +Drosophila_melanogasterlG015162lGNP_523984.1:66 |
| nsf1C | 88 | 5 | +Drosophila_melanogasterlG011085lGNP_001285396.1+Drosophila_melanogasterlG017298lGNP_573258.1:82 |
| nsf1G | 90 | 3 | +Drosophila_melanogasterlG017549lGNP_608447.1+Drosophila_melanogasterlG023909lGNP_651811.1:78 |
| nsf1J | 91 | 6 | +Drosophila_melanogasterlG016547lGNP_572308.3:9 |
| nsf1K | 89 | 3 | +Drosophila_melanogasterlG015602lGNP_524464.1:83 |
| nsf1L | 90 | 4 | +Drosophila_melanogasterlG010799lGNP_001285108.1+Drosophila_melanogasterlG016839lGNP_572686.1+Drosophila_melanogasterlG022448lGNP_649938.2+Drosophila_melanogasterlG028180lGNP_731401.3:75 |
| nsf1M | 89 | 3 | +Drosophila_melanogasterlG013165lGNP_001287493.1+Drosophila_melanogasterlG015606lGNP_524469.2:84 |
| nsf2A | 90 | 5 | +Drosophila_melanogasterlG001631lGNP_001097249.1+Drosophila_melanogasterlG001632lGNP_001097250.1+Drosophila_melanogasterlG011945lGNP_001286261.1+Drosophila_melanogasterlG014337lGNP_477369.1:81 |
| orf2 | 82 | 1 | +Drosophila_melanogasterlG017510lGNP_608387.1:82 |
| ornamtransa | 82 | 1 | +Drosophila_melanogasterlG021817lGNP_649139.1:82 |
| pace5A | 82 | 1 | +Drosophila_melanogasterlG008763lGNP_001261482.1+Drosophila_melanogasterlG020960lGNP_648057.2:82 |
| psmaA | 85 | 2 | +Drosophila_melanogasterlG014202lGNP_477202.2+Drosophila_melanogasterlG025806lGNP_725669.1:83 |
| psmaB | 85 | 2 | +Drosophila_melanogasterlG016112lGNP_525092.1:83 |
| psmaC | 85 | 3 | +Drosophila_melanogasterlG013789lGNP_476691.1:80 |
| psmaD | 86 | 2 | +Drosophila_melanogasterlG015475lGNP_524328.1:83 |
| psmaE | 86 | 3 | +Drosophila_melanogasterlG011484lGNP_001285798.1+Drosophila_melanogasterlG014738lGNP_523532.1:81 |
| psmaF | 84 | 2 | +Drosophila_melanogasterlG025407lGNP_724834.1:83 |
| psmaG | 87 | 1 | +Drosophila_melanogasterlG003238lGNP_001163078.1+Drosophila_melanogasterlG015916lGNP_524837.2+Drosophila_melanogasterlG025299lGNP_724614.1+Drosophila_melanogasterlG025301lGNP_724616.1:87 |
| psmbH | 82 | 1 | +Drosophila_melanogasterlG007836lGNP_001260511.1+Drosophila_melanogasterlG018665lGNP_609804.1:82 |
| psmbI | 83 | 2 | +Drosophila_melanogasterlG022377lGNP_649858.1:82 |
| psmbJ | 85 | 1 | +Drosophila_melanogasterlG015280lGNP_524115.1:85 |
| psmbK | 84 | 3 | +Drosophila_melanogasterlG016509lGNP_572267.1:10 |
| psmbL | 83 | 1 | +Drosophila_melanogasterlG024076lGNP_652031.2:83 |
| psmbM | 84 | 1 | +Drosophila_melanogasterlG024061lGNP_652014.1+Drosophila_melanogasterlG025477lGNP_724974.1:84 |
| psmbN | 80 | 1 | +Drosophila_melanogasterlG022121lGNP_649529.1+Drosophila_melanogasterlG027990lGNP_730922.1:80 |
| pyrdehydroe1bmt | 84 | 2 | +Drosophila_melanogasterlG023790lGNP_651668.1+Drosophila_melanogasterlG029027lGNP_733265.1:78 |
| rad23 | 79 | 2 | +Drosophila_melanogasterlG026196lGNP_726561.1:75 |

| | | | |
|---|---|---|---|
| rf1 | 83 | 4 | +Drosophila_melanogasterlG009368lGNP_001262104.1+Drosophila_melanogasterlG009369lGNP_001262105.1+Drosophila_melanogasterlG021874lGNP_649210.1+Drosophila_melanogasterlG027810lGNP_730517.1+Drosophila_melanogasterlG027811lGNP_730518.1+Drosophila_melanogasterlG027812lGNP_730519.1+Drosophila_melanogasterlG027813lGNP_730520.1+Drosophila_melanogasterlG029459lGNP_788547.1:71 |
| rla2A | 80 | 1 | +Drosophila_melanogasterlG014951lGNP_523764.1:80 |
| rla2B | 85 | 1 | +Drosophila_melanogasterlG007165lGNP_001259822.1+Drosophila_melanogasterlG013745lGNP_476630.1:85 |
| rpl1 | 88 | 1 | +Drosophila_melanogasterlG021309lGNP_648514.1:88 |
| rpl11b | 85 | 1 | +Drosophila_melanogasterlG012294lGNP_001286614.1+Drosophila_melanogasterlG014089lGNP_477054.1:85 |
| rpl12b | 86 | 1 | +Drosophila_melanogasterlG015898lGNP_524819.1+Drosophila_melanogasterlG026125lGNP_726412.1+Drosophila_melanogasterlG026126lGNP_726413.1:86 |
| rpl13 | 82 | 1 | +Drosophila_melanogasterlG000791lGNP_001033891.1+Drosophila_melanogasterlG007643lGNP_001260312.1+Drosophila_melanogasterlG014736lGNP_523530.1:82 |
| rpl14a | 81 | 1 | +Drosophila_melanogasterlG015153lGNP_523975.1:81 |
| rpl15a | 82 | 1 | +Drosophila_melanogasterlG000295lGNP_001015155.1+Drosophila_melanogasterlG000296lGNP_001015156.1+Drosophila_melanogasterlG002267lGNP_001104429.2+Drosophila_melanogasterlG002268lGNP_001104430.1+Drosophila_melanogasterlG004644lGNP_001245395.1+Drosophila_melanogasterlG004645lGNP_001245396.1+Drosophila_melanogasterlG004646lGNP_001245397.1+Drosophila_melanogasterlG004647lGNP_001245398.1:82 |
| rpl16b | 82 | 2 | +Drosophila_melanogasterlG022145lGNP_649560.1:80 |
| rpl17 | 85 | 1 | +Drosophila_melanogasterlG010654lGNP_001284961.1+Drosophila_melanogasterlG016578lGNP_572346.1+Drosophila_melanogasterlG026454lGNP_727118.1+Drosophila_melanogasterlG026455lGNP_727119.1+Drosophila_melanogasterlG026456lGNP_727120.1:85 |
| rpl18 | 85 | 1 | +Drosophila_melanogasterlG008782lGNP_001261501.1+Drosophila_melanogasterlG012644lGNP_001286966.1+Drosophila_melanogasterlG020985lGNP_648091.1:85 |
| rpl19a | 84 | 1 | +Drosophila_melanogasterlG013746lGNP_476631.1+Drosophila_melanogasterlG029972lGNP_995941.1:84 |
| rpl2 | 84 | 1 | +Drosophila_melanogasterlG015819lGNP_524726.1+Drosophila_melanogasterlG027101lGNP_728756.1:84 |
| rpl20 | 85 | 1 | +Drosophila_melanogasterlG014960lGNP_523774.1:85 |
| rpl21 | 80 | 1 | +Drosophila_melanogasterlG008006lGNP_001260689.1+Drosophila_melanogasterlG011817lGNP_001286133.1+Drosophila_melanogasterlG018941lGNP_610144.1:80 |
| rpl22 | 74 | 2 | +Drosophila_melanogasterlG014147lGNP_477134.1:70 |
| rpl23a | 80 | 1 | +Drosophila_melanogasterlG014998lGNP_523813.1:80 |
| rpl24A | 84 | 1 | +Drosophila_melanogasterlG011579lGNP_001285895.1+Drosophila_melanogasterlG011580lGNP_001285896.1+Drosophila_melanogasterlG018540lGNP_609649.1:84 |
| rpl24B | 85 | 2 | +Drosophila_melanogasterlG022556lGNP_650073.1:80 |
| rpl25 | 81 | 1 | +Drosophila_melanogasterlG015066lGNP_523886.1:81 |
| rpl26 | 80 | 1 | +Drosophila_melanogasterlG009293lGNP_001262025.1+Drosophila_melanogasterlG013388lGNP_001303372.1+Drosophila_melanogasterlG021760lGNP_649070.1:80 |
| rpl27 | 80 | 1 | +Drosophila_melanogasterlG004071lGNP_001188689.2+Drosophila_melanogasterlG011288lGNP_001285601.1+Drosophila_melanogasterlG014009lGNP_476963.1:80 |
| rpl3 | 88 | 1 | +Drosophila_melanogasterlG015463lGNP_524316.1+Drosophila_melanogasterlG028229lGNP_731548.2:88 |

| | | | |
|---|---|---|---|
| rpl30 | 78 | 1 | +Drosophila_melanogasterIG007871IGNP_001260550.2+Drosophila_melanogasterIG015787IGNP_524687.1+Drosophila_melanogasterIG025054IGNP_724149.1:78 |
| rpl31 | 83 | 1 | +Drosophila_melanogasterIG019233IGNP_610503.1+Drosophila_melanogasterIG025392IGNP_724804.1+Drosophila_melanogasterIG025393IGNP_724805.1:83 |
| rpl32 | 80 | 3 | +Drosophila_melanogasterIG029085IGNP_733339.1:23 |
| rpl33a | 76 | 1 | +Drosophila_melanogasterIG022130IGNP_649539.1:76 |
| rpl34 | 74 | 2 | +Drosophila_melanogasterIG012924IGNP_001287249.1+Drosophila_melanogasterIG012925IGNP_001287250.1+Drosophila_melanogasterIG022403IGNP_649887.1+Drosophila_melanogasterIG028158IGNP_731345.1:67 |
| rpl35 | 74 | 1 | +Drosophila_melanogasterIG010593IGNP_001284900.1+Drosophila_melanogasterIG016491IGNP_572243.1+Drosophila_melanogasterIG026428IGNP_727016.1:74 |
| rpl36 | 72 | 1 | +Drosophila_melanogasterIG010443IGNP_001284750.1+Drosophila_melanogasterIG013744IGNP_476629.1+Drosophila_melanogasterIG026269IGNP_726686.1+Drosophila_melanogasterIG026270IGNP_726687.1+Drosophila_melanogasterIG026271IGNP_726688.1:72 |
| rpl37a | 57 | 1 | +Drosophila_melanogasterIG006918IGNP_001259561.1+Drosophila_melanogasterIG017091IGNP_573005.1:57 |
| rpl42 | 73 | 1 | +Drosophila_melanogasterIG011423IGNP_001285737.1+Drosophila_melanogasterIG018149IGNP_609179.2:73 |
| rpl43b | 17 | 1 | +Drosophila_melanogasterIG004073IGNP_001188691.1+Drosophila_melanogasterIG015868IGNP_524781.1+Drosophila_melanogasterIG024557IGNP_723060.1:17 |
| rpl4B | 86 | 1 | +Drosophila_melanogasterIG015671IGNP_524538.2:86 |
| rpl6 | 80 | 1 | +Drosophila_melanogasterIG029168IGNP_733433.1:80 |
| rpl7A | 83 | 1 | +Drosophila_melanogasterIG011480IGNP_001285794.1+Drosophila_melanogasterIG014737IGNP_523531.1:83 |
| rpl9 | 84 | 1 | +Drosophila_melanogasterIG011510IGNP_001285826.1+Drosophila_melanogasterIG014169IGNP_477161.1+Drosophila_melanogasterIG024814IGNP_723644.1:84 |
| rpp0 | 85 | 1 | +Drosophila_melanogasterIG009463IGNP_001262202.1+Drosophila_melanogasterIG015364IGNP_524211.1:85 |
| rps1 | 88 | 2 | +Drosophila_melanogasterIG004652IGNP_001245404.1+Drosophila_melanogasterIG015730IGNP_524618.1:85 |
| rps10 | 82 | 1 | +Drosophila_melanogasterIG004973IGNP_001245758.1+Drosophila_melanogasterIG011134IGNP_001285445.1+Drosophila_melanogasterIG017453IGNP_608324.1+Drosophila_melanogasterIG026905IGNP_728273.1:82 |
| rps11 | 84 | 1 | +Drosophila_melanogasterIG025525IGNP_725114.1:84 |
| rps13a | 82 | 1 | +Drosophila_melanogasterIG000790IGNP_001033885.1+Drosophila_melanogasterIG011433IGNP_001285747.1+Drosophila_melanogasterIG013986IGNP_476938.1:82 |
| rps14 | 87 | 1 | +Drosophila_melanogasterIG010687IGNP_001284995.1+Drosophila_melanogasterIG015957IGNP_524884.1+Drosophila_melanogasterIG016143IGNP_536352.1+Drosophila_melanogasterIG026490IGNP_727218.1:87 |
| rps15 | 83 | 2 | +Drosophila_melanogasterIG025778IGNP_725591.1:29 |
| rps16 | 83 | 1 | +Drosophila_melanogasterIG012411IGNP_001286731.1+Drosophila_melanogasterIG020236IGNP_611685.1:83 |
| rps17 | 81 | 1 | +Drosophila_melanogasterIG015178IGNP_524002.1:81 |
| rps18 | 82 | 1 | +Drosophila_melanogasterIG014010IGNP_476964.1+Drosophila_melanogasterIG025922IGNP_725943.1:82 |
| rps19 | 82 | 1 | +Drosophila_melanogasterIG011028IGNP_001285338.1+Drosophila_melanogasterIG011029IGNP_001285339.1+Drosophila_melanogasterIG014600IGNP_523376.1+Drosophila_melanogasterIG026800IGNP_727992.1+Drosophila_melanogasterIG026801IGNP_727993.1:82 |

| | | | |
|---|---|---|---|
| rps2 | 86 | 1 | +Drosophila_melanogasterlG007634lGNP_001260303.1+Drosophila_melanogasterlG011471lGNP_001285785.1+Drosophila_melanogasterlG013939lGNP_476874.1:86 |
| rps20 | 77 | 1 | +Drosophila_melanogasterlG015562lGNP_524421.1:77 |
| rps22a | 83 | 1 | +Drosophila_melanogasterlG002938lGNP_001162738.1+Drosophila_melanogasterlG015806lGNP_524709.1+Drosophila_melanogasterlG026672lGNP_727690.1+Drosophila_melanogasterlG026673lGNP_727692.1+Drosophila_melanogasterlG026674lGNP_727693.1:83 |
| rps23 | 85 | 1 | +Drosophila_melanogasterlG012094lGNP_001286410.1+Drosophila_melanogasterlG019604lGNP_610939.2:85 |
| rps24 | 81 | 1 | +Drosophila_melanogasterlG020242lGNP_611693.1:81 |
| rps25 | 79 | 1 | +Drosophila_melanogasterlG015462lGNP_524315.2+Drosophila_melanogasterlG028227lGNP_731544.1:79 |
| rps26 | 83 | 1 | +Drosophila_melanogasterlG007859lGNP_001260537.1+Drosophila_melanogasterlG011726lGNP_001286042.1+Drosophila_melanogasterlG014796lGNP_523595.1+Drosophila_melanogasterlG025038lGNP_724109.1+Drosophila_melanogasterlG025039lGNP_724110.1:83 |
| rps27 | 19 | 1 | +Drosophila_melanogasterlG006339lGNP_001247301.1+Drosophila_melanogasterlG023548lGNP_651359.1:19 |
| rps27a | 88 | 2 | +Drosophila_melanogasterlG007358lGNP_001260018.1+Drosophila_melanogasterlG013862lGNP_476776.1:28 |
| rps3 | 85 | 1 | +Drosophila_melanogasterlG013153lGNP_001287481.1+Drosophila_melanogasterlG013747lGNP_476632.1:85 |
| rps4 | 88 | 1 | +Drosophila_melanogasterlG012732lGNP_001287055.1+Drosophila_melanogasterlG015223lGNP_524053.2+Drosophila_melanogasterlG027585lGNP_729871.1:88 |
| rps5 | 86 | 1 | +Drosophila_melanogasterlG011049lGNP_001285360.1+Drosophila_melanogasterlG011050lGNP_001285361.1+Drosophila_melanogasterlG011051lGNP_001285362.1+Drosophila_melanogasterlG014605lGNP_523382.1:86 |
| rps6 | 83 | 2 | +Drosophila_melanogasterlG014487lGNP_511073.1:76 |
| rps7 | 85 | 1 | +Drosophila_melanogasterlG023887lGNP_651782.1+Drosophila_melanogasterlG029096lGNP_733355.1+Drosophila_melanogasterlG029097lGNP_733356.2+Drosophila_melanogasterlG030283lGNP_996312.1:85 |
| rps8 | 86 | 1 | +Drosophila_melanogasterlG006396lGNP_001247362.1+Drosophila_melanogasterlG013266lGNP_001287596.1+Drosophila_melanogasterlG013267lGNP_001287597.1+Drosophila_melanogasterlG023853lGNP_651740.1:86 |
| rps9 | 83 | 2 | +Drosophila_melanogasterlG012686lGNP_001287008.1+Drosophila_melanogasterlG015180lGNP_524004.2+Drosophila_melanogasterlG027431lGNP_729506.1:78 |
| sadhchydrolaseE1 | 86 | 4 | +Drosophila_melanogasterlG006927lGNP_001259573.1+Drosophila_melanogasterlG010959lGNP_001285268.1+Drosophila_melanogasterlG014572lGNP_511164.2:70 |
| sap40 | 83 | 1 | +Drosophila_melanogasterlG010478lGNP_001284785.1+Drosophila_melanogasterlG013837lGNP_476750.1+Drosophila_melanogasterlG026303lGNP_726744.1+Drosophila_melanogasterlG026304lGNP_726745.3:83 |
| srp54 | 83 | 2 | +Drosophila_melanogasterlG015110lGNP_523931.1:78 |
| srs | 82 | 1 | +Drosophila_melanogasterlG007323lGNP_001259982.1+Drosophila_melanogasterlG017783lGNP_608743.2:82 |
| stbproptase2ab | 85 | 2 | +Drosophila_melanogasterlG011411lGNP_001285724.1+Drosophila_melanogasterlG011412lGNP_001285725.1+Drosophila_melanogasterlG013884lGNP_476805.1:78 |
| stcproptase2ac | 85 | 3 | +Drosophila_melanogasterlG006647lGNP_001259272.1+Drosophila_melanogasterlG006648lGNP_001259273.1+Drosophila_melanogasterlG006649lGNP_001259274.1+Drosophila_melanogasterlG014476lGNP_511061.1:57 |
| suca | 86 | 3 | +Drosophila_melanogasterlG004391lGNP_001189044.1+Drosophila_melanogasterlG015085lGNP_523905.2:80 |

| | | | |
|---|---|---|---|
| tif2a | 81 | 1 | +Drosophila_melanogasterlG011019lGNP_001285329.1+Drosophila_melanogasterlG017192lGNP_573130.1:81 |
| tribe1006 | 67 | 1 | +Drosophila_melanogasterlG023403lGNP_651181.1:67 |
| tribe1007 | 77 | 1 | +Drosophila_melanogasterlG007218lGNP_001259877.1+Drosophila_melanogasterlG014046lGNP_477005.1:77 |
| tribe1009 | 82 | 2 | +Drosophila_melanogasterlG022273lGNP_649725.2:49 |
| tribe1015 | 79 | 2 | +Drosophila_melanogasterlG023176lGNP_650887.1:48 |
| tribe1026 | 82 | 1 | +Drosophila_melanogasterlG023164lGNP_650864.1:82 |
| tribe1046 | 46 | 1 | +Drosophila_melanogasterlG009727lGNP_001262472.1+Drosophila_melanogasterlG022532lGNP_650047.3:46 |
| tribe1047 | 77 | 2 | +Drosophila_melanogasterlG027357lGNP_729358.1:74 |
| tribe1050 | 76 | 1 | +Drosophila_melanogasterlG010636lGNP_001284943.1+Drosophila_melanogasterlG013574lGNP_001303559.1+Drosophila_melanogasterlG016555lGNP_572318.1:76 |
| tribe1054 | 79 | 1 | +Drosophila_melanogasterlG022119lGNP_649527.1:79 |
| tribe1081 | 81 | 1 | +Drosophila_melanogasterlG009380lGNP_001262116.1+Drosophila_melanogasterlG021894lGNP_649234.1:81 |
| tribe1099 | 71 | 1 | +Drosophila_melanogasterlG014015lGNP_476969.1:71 |
| tribe1118 | 80 | 1 | +Drosophila_melanogasterlG018369lGNP_609444.1:80 |
| tribe1121 | 14 | 1 | +Drosophila_melanogasterlG024118lGNP_652189.1:14 |
| tribe1132 | 80 | 1 | +Drosophila_melanogasterlG003428lGNP_001163296.1+Drosophila_melanogasterlG020469lGNP_611954.2+Drosophila_melanogasterlG026145lGNP_726470.1+Drosophila_melanogasterlG026146lGNP_726471.1:80 |
| tribe1136 | 73 | 1 | +Drosophila_melanogasterlG008182lGNP_001260877.1+Drosophila_melanogasterlG019335lGNP_610629.1:73 |
| tribe1165 | 73 | 1 | +Drosophila_melanogasterlG021227lGNP_648409.1:73 |
| tribe1169 | 81 | 2 | +Drosophila_melanogasterlG005655lGNP_001246538.1:80 |
| tribe1170 | 78 | 1 | +Drosophila_melanogasterlG011573lGNP_001285889.1+Drosophila_melanogasterlG018533lGNP_609642.1:78 |
| tribe1181 | 79 | 1 | +Drosophila_melanogasterlG013404lGNP_001303388.1+Drosophila_melanogasterlG022038lGNP_649424.1:79 |
| tribe1190 | 77 | 1 | +Drosophila_melanogasterlG015557lGNP_524416.1:77 |
| tribe1200 | 75 | 1 | +Drosophila_melanogasterlG015351lGNP_524197.1:75 |
| tribe1204 | 77 | 1 | +Drosophila_melanogasterlG003783lGNP_001163688.1+Drosophila_melanogasterlG023303lGNP_651053.1:77 |
| tribe1244 | 79 | 1 | +Drosophila_melanogasterlG017650lGNP_608575.1:79 |
| tribe1245 | 81 | 2 | +Drosophila_melanogasterlG006072lGNP_001247010.1+Drosophila_melanogasterlG022438lGNP_649925.1:80 |
| tribe1294 | 79 | 1 | +Drosophila_melanogasterlG022551lGNP_650067.1:79 |
| tribe1335 | 78 | 1 | +Drosophila_melanogasterlG006320lGNP_001247279.1+Drosophila_melanogasterlG023451lGNP_651234.1:78 |
| tribe1378 | 75 | 1 | +Drosophila_melanogasterlG019718lGNP_611078.1:75 |
| tribe1381A | 80 | 1 | +Drosophila_melanogasterlG014381lGNP_477419.1:80 |
| tribe1409 | 82 | 1 | +Drosophila_melanogasterlG009017lGNP_001261742.1+Drosophila_melanogasterlG024087lGNP_652042.1:82 |
| tribe281 | 77 | 1 | +Drosophila_melanogasterlG009743lGNP_001262488.1+Drosophila_melanogasterlG013501lGNP_001303486.1+Drosophila_melanogasterlG014217lGNP_477217.1+Drosophila_melanogasterlG028266lGNP_731641.1:77 |
| tribe320 | 86 | 1 | +Drosophila_melanogasterlG013106lGNP_001287433.1+Drosophila_melanogasterlG023202lGNP_650922.1+Drosophila_melanogasterlG028666lGNP_732566.1:86 |

| | | | |
|---|---|---|---|
| tribe333 | 81 | 1 | +Drosophila_melanogaster\|G011668\|GNP_001285984.1+Drosophila_melanogaster\|G014183\|GNP_477176.1:81 |
| tribe340 | 14 | 2 | +Drosophila_melanogaster\|G007311\|GNP_001259970.1+Drosophila_melanogaster\|G024469\|GNP_722853.1+Drosophila_melanogaster\|G024470\|GNP_722854.1+Drosophila_melanogaster\|G024471\|GNP_722855.1:12 |
| tribe351 | 29 | 1 | +Drosophila_melanogaster\|G003466\|GNP_001163336.1+Drosophila_melanogaster\|G003467\|GNP_001163337.1+Drosophila_melanogaster\|G020751\|GNP_647791.2+Drosophila_melanogaster\|G027146\|GNP_728839.1+Drosophila_melanogaster\|G027147\|GNP_728840.1+Drosophila_melanogaster\|G027148\|GNP_728841.2:29 |
| tribe368 | 23 | 1 | +Drosophila_melanogaster\|G030477\|GYP_009047273.1:23 |
| tribe369 | 22 | 1 | +Drosophila_melanogaster\|G030474\|GYP_009047270.1:22 |
| tribe460 | 81 | 1 | +Drosophila_melanogaster\|G013446\|GNP_001303431.1+Drosophila_melanogaster\|G022533\|GNP_650048.1:81 |
| tribe495 | 82 | 1 | +Drosophila_melanogaster\|G015683\|GNP_524550.1+Drosophila_melanogaster\|G029059\|GNP_733304.1+Drosophila_melanogaster\|G029060\|GNP_733305.1:82 |
| tribe508 | 81 | 1 | +Drosophila_melanogaster\|G029626\|GNP_788765.1+Drosophila_melanogaster\|G029627\|GNP_788766.1:81 |
| tribe532 | 83 | 1 | +Drosophila_melanogaster\|G015504\|GNP_524358.2:83 |
| tribe542 | 79 | 1 | +Drosophila_melanogaster\|G020752\|GNP_647792.1:79 |
| tribe550 | 81 | 3 | +Drosophila_melanogaster\|G008021\|GNP_001260708.1:5 |
| tribe572 | 81 | 1 | +Drosophila_melanogaster\|G007217\|GNP_001259876.1+Drosophila_melanogaster\|G015839\|GNP_524748.2+Drosophila_melanogaster\|G024401\|GNP_722715.1:81 |
| tribe585A | 85 | 5 | +Drosophila_melanogaster\|G014677\|GNP_523463.2:80 |
| tribe586 | 84 | 1 | +Drosophila_melanogaster\|G015268\|GNP_524103.2:84 |
| tribe593 | 85 | 1 | +Drosophila_melanogaster\|G006762\|GNP_001259397.1+Drosophila_melanogaster\|G010765\|GNP_001285074.1+Drosophila_melanogaster\|G016785\|GNP_572610.1:85 |
| tribe613 | 14 | 1 | +Drosophila_melanogaster\|G023745\|GNP_651614.1:14 |
| tribe619 | 16 | 1 | +Drosophila_melanogaster\|G024082\|GNP_652037.1:16 |
| tribe622 | 81 | 1 | +Drosophila_melanogaster\|G014020\|GNP_476975.1+Drosophila_melanogaster\|G025507\|GNP_725073.1:81 |
| tribe629A | 80 | 1 | +Drosophila_melanogaster\|G025523\|GNP_725106.1:80 |
| tribe632 | 82 | 1 | +Drosophila_melanogaster\|G000743\|GNP_001033818.1+Drosophila_melanogaster\|G010448\|GNP_001284755.1+Drosophila_melanogaster\|G014357\|GNP_477390.1+Drosophila_melanogaster\|G026272\|GNP_726690.1+Drosophila_melanogaster\|G026273\|GNP_726691.1+Drosophila_melanogaster\|G026274\|GNP_726692.1+Drosophila_melanogaster\|G026275\|GNP_726693.1+Drosophila_melanogaster\|G026276\|GNP_726694.1+Drosophila_melanogaster\|G026277\|GNP_726695.1:82 |
| tribe646 | 74 | 1 | +Drosophila_melanogaster\|G011300\|GNP_001285613.1+Drosophila_melanogaster\|G011301\|GNP_001285614.1+Drosophila_melanogaster\|G017921\|GNP_608909.1:74 |
| tribe647 | 75 | 1 | +Drosophila_melanogaster\|G003594\|GNP_001163480.1+Drosophila_melanogaster\|G003595\|GNP_001163481.1+Drosophila_melanogaster\|G009366\|GNP_001262101.1+Drosophila_melanogaster\|G015334\|GNP_524178.2:75 |
| tribe683 | 27 | 1 | +Drosophila_melanogaster\|G007791\|GNP_001260465.1+Drosophila_melanogaster\|G018615\|GNP_609747.1:27 |
| tribe686 | 58 | 2 | +Drosophila_melanogaster\|G025733\|GNP_725504.1:24 |
| tribe700 | 79 | 1 | +Drosophila_melanogaster\|G005875\|GNP_001246795.1+Drosophila_melanogaster\|G005876\|GNP_001246796.1+Drosophila_ |

| | | | |
|---|---|---|---|
| | | | melanogasterlG015275lGNP_524110.1+Drosophila_melanogasterlG027677lGNP_730178.1:79 |
| tribe716 | 78 | 1 | +Drosophila_melanogasterlG015186lGNP_524011.1:78 |
| tribe717 | 78 | 1 | +Drosophila_melanogasterlG021397lGNP_648622.1:78 |
| tribe726 | 11 | 2 | +Drosophila_melanogasterlG004981lGNP_001245767.1+Drosophila_melanogasterlG026911lGNP_728295.1+Drosophila_melanogasterlG026912lGNP_728296.1:10 |
| tribe739 | 80 | 1 | +Drosophila_melanogasterlG011365lGNP_001285678.1+Drosophila_melanogasterlG018038lGNP_609046.1:80 |
| tribe740 | 56 | 1 | +Drosophila_melanogasterlG018191lGNP_609222.1:56 |
| tribe756 | 76 | 2 | +Drosophila_melanogasterlG012731lGNP_001287054.1+Drosophila_melanogasterlG021399lGNP_648624.1:73 |
| tribe764 | 67 | 1 | +Drosophila_melanogasterlG011999lGNP_001286315.1+Drosophila_melanogasterlG019380lGNP_610679.1:67 |
| tribe765 | 20 | 1 | +Drosophila_melanogasterlG018135lGNP_609162.1:20 |
| tribe768 | 82 | 1 | +Drosophila_melanogasterlG022365lGNP_649840.1:82 |
| tribe801mt | 78 | 1 | +Drosophila_melanogasterlG011003lGNP_001285313.1+Drosophila_melanogasterlG017164lGNP_573097.1+Drosophila_melanogasterlG026754lGNP_727921.1:78 |
| tribe831 | 86 | 1 | +Drosophila_melanogasterlG010749lGNP_001285058.1+Drosophila_melanogasterlG016754lGNP_572567.1:86 |
| tribe832 | 84 | 1 | +Drosophila_melanogasterlG013966lGNP_476905.1+Drosophila_melanogasterlG029944lGNP_995904.1:84 |
| tribe850 | 79 | 1 | +Drosophila_melanogasterlG000115lGNP_001014579.1+Drosophila_melanogasterlG000116lGNP_001014580.1+Drosophila_melanogasterlG015168lGNP_523990.1:79 |
| tribe852 | 78 | 1 | +Drosophila_melanogasterlG017397lGNP_573385.1:78 |
| tribe858 | 81 | 2 | +Drosophila_melanogasterlG014044lGNP_477003.1:76 |
| tribe893 | 78 | 1 | +Drosophila_melanogasterlG022210lGNP_649645.1:78 |
| tribe895 | 82 | 1 | +Drosophila_melanogasterlG020168lGNP_611604.1:82 |
| tribe896 | 86 | 1 | +Drosophila_melanogasterlG020738lGNP_647775.1:86 |
| tribe905 | 65 | 1 | +Drosophila_melanogasterlG000992lGNP_001036353.1+Drosophila_melanogasterlG000993lGNP_001036354.1+Drosophila_melanogasterlG024080lGNP_652035.1:65 |
| tribe906 | 74 | 2 | +Drosophila_melanogasterlG009351lGNP_001262085.1+Drosophila_melanogasterlG021841lGNP_649169.3:72 |
| tribe927 | 79 | 1 | +Drosophila_melanogasterlG023971lGNP_651885.1:79 |
| tribe930 | 80 | 1 | +Drosophila_melanogasterlG014980lGNP_523794.1+Drosophila_melanogasterlG025907lGNP_725894.1:80 |
| tribe942 | 81 | 1 | +Drosophila_melanogasterlG011067lGNP_001285378.1+Drosophila_melanogasterlG017273lGNP_573228.1:81 |
| u2snrnp | 81 | 1 | +Drosophila_melanogasterlG007157lGNP_001259814.1+Drosophila_melanogasterlG014208lGNP_477208.1:81 |
| vacaatpasepl21a | 78 | 1 | +Drosophila_melanogasterlG006166lGNP_001247111.1+Drosophila_melanogasterlG024057lGNP_652010.1:78 |
| vata | 82 | 5 | +Drosophila_melanogasterlG005196lGNP_001246015.1+Drosophila_melanogasterlG007751lGNP_001260424.1+Drosophila_melanogasterlG007752lGNP_001260425.1+Drosophila_melanogasterlG007753lGNP_001260426.1+Drosophila_melanogasterlG014762lGNP_523560.2+Drosophila_melanogasterlG018489lGNP_609595.1+Drosophila_melanogasterlG024055lGNP_652004.2+Drosophila_melanogasterlG024862lGNP_723775.1+Drosophila_melanogasterlG024863lGNP_723776.1:69 |
| vatb | 85 | 2 | +Drosophila_melanogasterlG003698lGNP_001163597.1+Drosophila_melanogasterlG013969lGNP_476908.1+Drosophila_melanogasterlG028297lGNP_731726.1:84 |
| vatc | 82 | 2 | +Drosophila_melanogasterlG014257lGNP_477266.1+Drosophila_melanogasterlG017436lGNP_599140.1:80 |

| | | | |
|---|---|---|---|
| vate | 81 | 1 | +Drosophila_melanogasterlG012858lGNP_001287182.1+Drosophila_melanogasterlG015389lGNP_524237.1+Drosophila_melanogasterlG028010lGNP_730957.1:81 |
| vatpased | 82 | 1 | +Drosophila_melanogasterlG024045lGNP_651987.1:82 |
| vdac2 | 85 | 1 | +Drosophila_melanogasterlG000798lGNP_001033899.1+Drosophila_melanogasterlG005153lGNP_001245961.1+Drosophila_melanogasterlG007695lGNP_001260365.1+Drosophila_melanogasterlG013890lGNP_476813.1+Drosophila_melanogasterlG017413lGNP_599110.1:85 |
| w09c | 83 | 1 | +Drosophila_melanogasterlG007677lGNP_001260347.1+Drosophila_melanogasterlG014347lGNP_477379.1:83 |

**Table S8.** Detection of paralogs in the 200 slowest-evolving gene matrix of Howard *et al.* (2020).

| Locus | Number of sequences with at least one hit | Number of orthogroups detected | Mappings in *D. melanogaster* proteome |
|---|---|---|---|
| OG10224 | 83 | 3 | +Drosophila_melanogasterlG005983lGNP_001246916.1+Drosophila_melanogasterlG015382lGNP_524230.2:78 |
| OG1026 | 77 | 1 | +Drosophila_melanogasterlG009653lGNP_001262397.1+Drosophila_melanogasterlG028140lGNP_731308.1:77 |
| OG10391 | 70 | 1 | +Drosophila_melanogasterlG028412lGNP_731983.1:70 |
| OG10592 | 83 | 3 | +Drosophila_melanogasterlG005344lGNP_001246192.1+Drosophila_melanogasterlG015947lGNP_524874.2:67 |
| OG10605 | 78 | 1 | +Drosophila_melanogasterlG000743lGNP_001033818.1+Drosophila_melanogasterlG010448lGNP_001284755.1+Drosophila_melanogasterlG014357lGNP_477390.1+Drosophila_melanogasterlG026272lGNP_726690.1+Drosophila_melanogasterlG026273lGNP_726691.1+Drosophila_melanogasterlG026274lGNP_726692.1+Drosophila_melanogasterlG026275lGNP_726693.1+Drosophila_melanogasterlG026276lGNP_726694.1+Drosophila_melanogasterlG026277lGNP_726695.1:78 |
| OG10726 | 75 | 1 | +Drosophila_melanogasterlG011003lGNP_001285313.1+Drosophila_melanogasterlG017164lGNP_573097.1+Drosophila_melanogasterlG026754lGNP_727921.1:75 |
| OG10928 | 74 | 1 | +Drosophila_melanogasterlG017999lGNP_608996.1:74 |
| OG11399 | 73 | 1 | +Drosophila_melanogasterlG015866lGNP_524779.1:73 |
| OG1161 | 83 | 3 | +Drosophila_melanogasterlG003840lGNP_001163753.1+Drosophila_melanogasterlG012515lGNP_001286835.1+Drosophila_melanogasterlG014981lGNP_523795.2+Drosophila_melanogasterlG015025lGNP_523842.2+Drosophila_melanogasterlG015438lGNP_524290.2+Drosophila_melanogasterlG023742lGNP_651606.2+Drosophila_melanogasterlG025908lGNP_725896.1:79 |
| OG11675 | 68 | 1 | +Drosophila_melanogasterlG023689lGNP_651538.1:68 |
| OG11754 | 80 | 2 | +Drosophila_melanogasterlG013864lGNP_476778.1:74 |
| OG1177 | 79 | 1 | +Drosophila_melanogasterlG015110lGNP_523931.1:79 |
| OG1184 | 82 | 2 | +Drosophila_melanogasterlG013858lGNP_476772.1+Drosophila_melanogasterlG015184lGNP_524009.2+Drosophila_melanogasterlG015414lGNP_524264.1+Drosophila_melanogasterlG015445lGNP_524297.1:79 |
| OG11903 | 80 | 1 | +Drosophila_melanogasterlG022661lGNP_650214.1+Drosophila_melanogasterlG028298lGNP_731727.2:80 |
| OG12034 | 82 | 2 | +Drosophila_melanogasterlG009831lGNP_001262578.1+Drosophila_melanogasterlG009832lGNP_001262579.1+Drosophila_melanogasterlG028398lGNP_731941.1:78 |
| OG12043 | 83 | 3 | +Drosophila_melanogasterlG009831lGNP_001262578.1+Drosophila_melanogasterlG009832lGNP_001262579.1+Drosophila_melanogasterlG028398lGNP_731941.1:78 |
| OG12103 | 75 | 1 | +Drosophila_melanogasterlG015813lGNP_524719.1:75 |
| OG1215 | 81 | 1 | +Drosophila_melanogasterlG026041lGNP_726243.1:81 |
| OG12175 | 83 | 1 | +Drosophila_melanogasterlG005421lGNP_001246276.1+Drosophila_melanogasterlG005422lGNP_001246277.1+Drosophila_melanogasterlG012021lGNP_001286337.1+Drosophila_melanogasterlG014899lGNP_523710.1+Drosophila_melanogasterlG025527lGNP_725120.1:83 |
| OG12223 | 77 | 1 | +Drosophila_melanogasterlG020399lGNP_611878.1+Drosophila_melanogasterlG026124lGNP_726411.1:77 |
| OG12247 | 78 | 1 | +Drosophila_melanogasterlG014010lGNP_476964.1+Drosophila_melanogasterlG025922lGNP_725943.1:78 |
| OG1234 | 67 | 3 | +Drosophila_melanogasterlG002702lGNP_001138070.1+Drosophila_melanogasterlG002703lGNP_001138071.1+Drosophila_melanogasterlG002704lGNP_001138072.1+Drosophila_melanogasterlG006224lGN |

| | | | |
|---|---|---|---|
| | | | P_001247176.1+Drosophila_melanogasterlG006225lGNP_001247177.1+Drosophila_melanogasterlG006226lGNP_001247178.1+Drosophila_melanogasterlG006227lGNP_001247179.1+Drosophila_melanogasterlG009944lGNP_001262694.1+Drosophila_melanogasterlG009945lGNP_001262695.1+Drosophila_melanogasterlG023017lGNP_650681.2+Drosophila_melanogasterlG023715lGNP_651569.1+Drosophila_melanogasterlG028543lGNP_732293.1+Drosophila_melanogasterlG028544lGNP_732294.1+Drosophila_melanogasterlG028545lGNP_732295.1+Drosophila_melanogasterlG028546lGNP_732296.1+Drosophila_melanogasterlG028990lGNP_733215.1+Drosophila_melanogasterlG028991lGNP_733216.1+Drosophila_melanogasterlG028992lGNP_733217.1+Drosophila_melanogasterlG028993lGNP_733218.1+Drosophila_melanogasterlG028994lGNP_733219.1:53 |
| OG12579 | 80 | 1 | +Drosophila_melanogasterlG010687lGNP_001284995.1+Drosophila_melanogasterlG015957lGNP_524884.1+Drosophila_melanogasterlG016143lGNP_536352.1+Drosophila_melanogasterlG026490lGNP_727218.1:80 |
| OG1269 | 76 | 4 | +Drosophila_melanogasterlG004933lGNP_001245715.1+Drosophila_melanogasterlG004934lGNP_001245716.1+Drosophila_melanogasterlG004935lGNP_001245717.1+Drosophila_melanogasterlG006411lGNP_001247377.1+Drosophila_melanogasterlG006412lGNP_001247378.2+Drosophila_melanogasterlG006974lGNP_001259622.1+Drosophila_melanogasterlG014597lGNP_523373.2+Drosophila_melanogasterlG015712lGNP_524600.3+Drosophila_melanogasterlG026798lGNP_727985.2+Drosophila_melanogasterlG026799lGNP_727986.2:64 |
| OG12776 | 73 | 1 | +Drosophila_melanogasterlG017857lGNP_608830.3:73 |
| OG1279 | 82 | 3 | +Drosophila_melanogasterlG012005lGNP_001286321.1+Drosophila_melanogasterlG012006lGNP_001286322.1+Drosophila_melanogasterlG014343lGNP_477375.1+Drosophila_melanogasterlG015723lGNP_524611.1+Drosophila_melanogasterlG025509lGNP_725085.1+Drosophila_melanogasterlG029180lGNP_733449.1+Drosophila_melanogasterlG030286lGNP_996315.1+Drosophila_melanogasterlG030287lGNP_996316.1:78 |
| OG12847 | 83 | 4 | +Drosophila_melanogasterlG023236lGNP_650961.2:73 |
| OG12881 | 80 | 1 | +Drosophila_melanogasterlG012094lGNP_001286410.1+Drosophila_melanogasterlG019604lGNP_610939.2:80 |
| OG12931 | 78 | 1 | +Drosophila_melanogasterlG000790lGNP_001033885.1+Drosophila_melanogasterlG011433lGNP_001285747.1+Drosophila_melanogasterlG013986lGNP_476938.1:78 |
| OG13008 | 75 | 1 | +Drosophila_melanogasterlG014528lGNP_511117.1:75 |
| OG13036 | 76 | 1 | +Drosophila_melanogasterlG013750lGNP_476636.1:76 |
| OG13068 | 75 | 1 | +Drosophila_melanogasterlG017146lGNP_573076.1+Drosophila_melanogasterlG026743lGNP_727896.1+Drosophila_melanogasterlG026744lGNP_727897.1+Drosophila_melanogasterlG030404lGNP_996448.1+Drosophila_melanogasterlG030405lGNP_996449.1:75 |
| OG13076 | 76 | 1 | +Drosophila_melanogasterlG019715lGNP_611074.1:76 |
| OG13084 | 81 | 1 | +Drosophila_melanogasterlG013921lGNP_476853.1+Drosophila_melanogasterlG025591lGNP_725235.1:81 |
| OG13130 | 77 | 3 | +Drosophila_melanogasterlG019763lGNP_611136.1:32 |
| OG13134 | 79 | 2 | +Drosophila_melanogasterlG014464lGNP_511049.1:76 |
| OG13144 | 83 | 1 | +Drosophila_melanogasterlG005421lGNP_001246276.1+Drosophila_melanogasterlG005422lGNP_001246277.1+Drosophila_melanogasterlG012021lGNP_001286337.1+Drosophila_melanogasterlG014899lGNP_523710.1+Drosophila_melanogasterlG025527lGNP_725120.1:83 |
| OG1317 | 83 | 3 | +Drosophila_melanogasterlG003840lGNP_001163753.1+Drosophila_melanogasterlG012515lGNP_001286835.1+Drosophila_melanogasterlG014981lGNP_523795.2+Drosophila_melanogasterlG015025lGNP_523842.2+Drosophila_melanogasterlG015438lGNP_524290.2+Drosophila_melanogasterlG023742lGNP_651606.2+Drosophila_melanogasterlG025908lGNP_725896.1:79 |
| OG1323 | 79 | 1 | +Drosophila_melanogasterlG018799lGNP_609965.1:79 |

| | | | |
|---|---|---|---|
| OG1325 | 78 | 1 | +Drosophila_melanogasterlG003781lGNP_001163686.1+Drosophila_melanogasterlG023299lGNP_651049.3+Drosophila_melanogasterlG028742lGNP_732744.1:78 |
| OG13309 | 83 | 3 | +Drosophila_melanogasterlG014590lGNP_523366.2:80 |
| OG13352 | 81 | 1 | +Drosophila_melanogasterlG013059lGNP_001287386.1+Drosophila_melanogasterlG015820lGNP_524728.2+Drosophila_melanogasterlG030219lGNP_996231.1:81 |
| OG1337 | 83 | 3 | +Drosophila_melanogasterlG003840lGNP_001163753.1+Drosophila_melanogasterlG012515lGNP_001286835.1+Drosophila_melanogasterlG014981lGNP_523795.2+Drosophila_melanogasterlG015025lGNP_523842.2+Drosophila_melanogasterlG015438lGNP_524290.2+Drosophila_melanogasterlG023742lGNP_651606.2+Drosophila_melanogasterlG025908lGNP_725896.1:79 |
| OG1348 | 81 | 4 | +Drosophila_melanogasterlG009368lGNP_001262104.1+Drosophila_melanogasterlG009369lGNP_001262105.1+Drosophila_melanogasterlG021874lGNP_649210.1+Drosophila_melanogasterlG027810lGNP_730517.1+Drosophila_melanogasterlG027811lGNP_730518.1+Drosophila_melanogasterlG027812lGNP_730519.1+Drosophila_melanogasterlG027813lGNP_730520.1+Drosophila_melanogasterlG029459lGNP_788547.1:70 |
| OG13634 | 79 | 2 | +Drosophila_melanogasterlG012619lGNP_001286940.1+Drosophila_melanogasterlG020882lGNP_647959.1:76 |
| OG13832 | 83 | 2 | +Drosophila_melanogasterlG013858lGNP_476772.1:82 |
| OG13968 | 78 | 1 | +Drosophila_melanogasterlG009293lGNP_001262025.1+Drosophila_melanogasterlG013388lGNP_001303372.1+Drosophila_melanogasterlG021760lGNP_649070.1:78 |
| OG14123 | 75 | 1 | +Drosophila_melanogasterlG014998lGNP_523813.1:75 |
| OG1423 | 80 | 1 | +Drosophila_melanogasterlG013165lGNP_001287493.1+Drosophila_melanogasterlG015606lGNP_524469.2:80 |
| OG1427 | 69 | 2 | +Drosophila_melanogasterlG020715lGNP_647746.1+Drosophila_melanogasterlG030211lGNP_996221.1+Drosophila_melanogasterlG030212lGNP_996222.1:62 |
| OG14312 | 76 | 1 | +Drosophila_melanogasterlG012411lGNP_001286731.1+Drosophila_melanogasterlG020236lGNP_611685.1:76 |
| OG14521 | 73 | 1 | +Drosophila_melanogasterlG000167lGNP_001014633.2:73 |
| OG14607 | 68 | 1 | +Drosophila_melanogasterlG018211lGNP_609246.1:68 |
| OG1480 | 78 | 3 | +Drosophila_melanogasterlG000152lGNP_001014617.1+Drosophila_melanogasterlG009775lGNP_001262520.1+Drosophila_melanogasterlG009776lGNP_001262521.1+Drosophila_melanogasterlG009777lGNP_001262522.1+Drosophila_melanogasterlG009778lGNP_001262523.1+Drosophila_melanogasterlG009779lGNP_001262524.1+Drosophila_melanogasterlG015483lGNP_524336.2+Drosophila_melanogasterlG028310lGNP_731762.1+Drosophila_melanogasterlG028311lGNP_731763.1+Drosophila_melanogasterlG028312lGNP_731764.1:58 |
| OG15046 | 70 | 3 | +Drosophila_melanogasterlG004610lGNP_001189293.1+Drosophila_melanogasterlG015649lGNP_524514.2+Drosophila_melanogasterlG028926lGNP_733133.1+Drosophila_melanogasterlG028927lGNP_733134.1+Drosophila_melanogasterlG028928lGNP_733135.1:10 |
| OG1506 | 82 | 2 | +Drosophila_melanogasterlG009840lGNP_001262587.1+Drosophila_melanogasterlG028418lGNP_731993.1+Drosophila_melanogasterlG028419lGNP_731994.1:70 |
| OG15319 | 77 | 1 | +Drosophila_melanogasterlG002938lGNP_001162738.1+Drosophila_melanogasterlG015806lGNP_524709.1+Drosophila_melanogasterlG026672lGNP_727690.1+Drosophila_melanogasterlG026673lGNP_727692.1+Drosophila_melanogasterlG026674lGNP_727693.1:77 |
| OG15504 | 80 | 1 | +Drosophila_melanogasterlG007358lGNP_001260018.1+Drosophila_melanogasterlG013862lGNP_476776.1:80 |
| OG1569 | 82 | 5 | +Drosophila_melanogasterlG029994lGNP_995971.2:1 |
| OG1603 | 62 | 3 | +Drosophila_melanogasterlG007886lGNP_001260565.1+Drosophila_melanogasterlG013883lGNP_476804.1+Drosophila_melanogasterlG013973lGNP_476922.1:41 |

| | | | |
|---|---|---|---|
| OG1605 | 79 | 1 | +Drosophila_melanogaster\|G022419\|NP_649906.1:79 |
| OG16210 | 78 | 1 | +Drosophila_melanogaster\|G000565\|NP_001027283.1+Drosophila_melanogaster\|G000569\|NP_001027287.1+Drosophila_melanogaster\|G000573\|NP_001027291.1+Drosophila_melanogaster\|G000578\|NP_001027296.1+Drosophila_melanogaster\|G000582\|NP_001027300.1+Drosophila_melanogaster\|G000587\|NP_001027305.1+Drosophila_melanogaster\|G000592\|NP_001027310.1+Drosophila_melanogaster\|G000597\|NP_001027315.1+Drosophila_melanogaster\|G000602\|NP_001027320.1+Drosophila_melanogaster\|G000607\|NP_001027325.1+Drosophila_melanogaster\|G000612\|NP_001027330.1+Drosophila_melanogaster\|G000617\|NP_001027335.1+Drosophila_melanogaster\|G000622\|NP_001027340.1+Drosophila_melanogaster\|G000627\|NP_001027345.1+Drosophila_melanogaster\|G000632\|NP_001027350.1+Drosophila_melanogaster\|G000637\|NP_001027355.1+Drosophila_melanogaster\|G000642\|NP_001027360.1+Drosophila_melanogaster\|G000647\|NP_001027365.1+Drosophila_melanogaster\|G000652\|NP_001027370.1+Drosophila_melanogaster\|G000657\|NP_001027375.1+Drosophila_melanogaster\|G000662\|NP_001027380.1+Drosophila_melanogaster\|G000667\|NP_001027385.1+Drosophila_melanogaster\|G025143\|NP_724342.1:78 |
| OG1624 | 80 | 1 | +Drosophila_melanogaster\|G007612\|NP_001260276.1+Drosophila_melanogaster\|G011457\|NP_001285771.1+Drosophila_melanogaster\|G014723\|NP_523517.1+Drosophila_melanogaster\|G024731\|NP_723438.1:80 |
| OG1625 | 73 | 3 | +Drosophila_melanogaster\|G020852\|NP_647918.2+Drosophila_melanogaster\|G024039\|NP_651978.2:52 |
| OG16557 | 80 | 1 | +Drosophila_melanogaster\|G027584\|NP_729866.1:80 |
| OG1684 | 82 | 1 | +Drosophila_melanogaster\|G008840\|NP_001261559.1+Drosophila_melanogaster\|G015146\|NP_523968.1:82 |
| OG17040 | 78 | 1 | +Drosophila_melanogaster\|G000565\|NP_001027283.1+Drosophila_melanogaster\|G000569\|NP_001027287.1+Drosophila_melanogaster\|G000573\|NP_001027291.1+Drosophila_melanogaster\|G000578\|NP_001027296.1+Drosophila_melanogaster\|G000582\|NP_001027300.1+Drosophila_melanogaster\|G000587\|NP_001027305.1+Drosophila_melanogaster\|G000592\|NP_001027310.1+Drosophila_melanogaster\|G000597\|NP_001027315.1+Drosophila_melanogaster\|G000602\|NP_001027320.1+Drosophila_melanogaster\|G000607\|NP_001027325.1+Drosophila_melanogaster\|G000612\|NP_001027330.1+Drosophila_melanogaster\|G000617\|NP_001027335.1+Drosophila_melanogaster\|G000622\|NP_001027340.1+Drosophila_melanogaster\|G000627\|NP_001027345.1+Drosophila_melanogaster\|G000632\|NP_001027350.1+Drosophila_melanogaster\|G000637\|NP_001027355.1+Drosophila_melanogaster\|G000642\|NP_001027360.1+Drosophila_melanogaster\|G000647\|NP_001027365.1+Drosophila_melanogaster\|G000652\|NP_001027370.1+Drosophila_melanogaster\|G000657\|NP_001027375.1+Drosophila_melanogaster\|G000662\|NP_001027380.1+Drosophila_melanogaster\|G000667\|NP_001027385.1+Drosophila_melanogaster\|G025143\|NP_724342.1:78 |
| OG17078 | 64 | 1 | +Drosophila_melanogaster\|G001374\|NP_001096936.1+Drosophila_melanogaster\|G006759\|NP_001259394.1+Drosophila_melanogaster\|G006760\|NP_001259395.1+Drosophila_melanogaster\|G006761\|NP_001259396.1+Drosophila_melanogaster\|G016784\|NP_572609.1:64 |
| OG17091 | 73 | 1 | +Drosophila_melanogaster\|G022210\|NP_649645.1:73 |
| OG17098 | 82 | 3 | +Drosophila_melanogaster\|G000574\|NP_001027292.1+Drosophila_melanogaster\|G000583\|NP_001027301.1+Drosophila_melanogaster\|G000588\|NP_001027306.1+Drosophila_melanogaster\|G000593\|NP_001027311.1+Drosophila_melanogaster\|G000598\|NP_001027316.1+Drosophila_melanogaster\|G000603\|NP_001027321.1+Drosophila_melanogaster\|G000608\|NP_001027326.1+Drosophila_melanogaster\|G000613\|NP_001027331.1+Drosophila_melanogaster\|G000618\|NP_001027336.1+Drosophila_melanogaster\|G000623\|NP_001027341.1+Drosophila_melanogaster\|G000628\|NP_001027346.1+Drosophila_melanogaster\|G000633\|NP_001027351.1+Drosophila_melanoga |

<table>
<tr><td></td><td></td><td></td><td>ster|G000638|GNP_001027356.1+Drosophila_melanogaster|G000643|GNP_001027361.1+Drosophila_melanogaster|G000663|GNP_001027381.1+Drosophila_melanogaster|G000668|GNP_001027386.1+Drosophila_melanogaster|G025144|GNP_724343.1:73</td></tr>
<tr><td>OG1726</td><td>81</td><td>2</td><td>+Drosophila_melanogaster|G024565|GNP_723089.1+Drosophila_melanogaster|G024566|GNP_723090.1+Drosophila_melanogaster|G024567|GNP_723091.1:77</td></tr>
<tr><td>OG1727</td><td>73</td><td>2</td><td>+Drosophila_melanogaster|G012943|GNP_001287269.1+Drosophila_melanogaster|G013943|GNP_476880.1+Drosophila_melanogaster|G013944|GNP_476881.1+Drosophila_melanogaster|G017414|GNP_599111.1+Drosophila_melanogaster|G017415|GNP_599112.1+Drosophila_melanogaster|G017416|GNP_599113.1+Drosophila_melanogaster|G028193|GNP_731451.1+Drosophila_melanogaster|G028194|GNP_731452.1+Drosophila_melanogaster|G028195|GNP_731453.1:58</td></tr>
<tr><td>OG17291</td><td>77</td><td>1</td><td>+Drosophila_melanogaster|G010447|GNP_001284754.1+Drosophila_melanogaster|G016215|GNP_569852.1:77</td></tr>
<tr><td>OG17319</td><td>76</td><td>1</td><td>+Drosophila_melanogaster|G026570|GNP_727447.1:76</td></tr>
<tr><td>OG17437</td><td>73</td><td>1</td><td>+Drosophila_melanogaster|G020942|GNP_648037.1:73</td></tr>
<tr><td>OG1753</td><td>83</td><td>2</td><td>+Drosophila_melanogaster|G005102|GNP_001245907.1+Drosophila_melanogaster|G007455|GNP_001260117.1+Drosophila_melanogaster|G013711|GNP_476595.1+Drosophila_melanogaster|G024583|GNP_723137.1+Drosophila_melanogaster|G024584|GNP_723138.1+Drosophila_melanogaster|G024585|GNP_723139.1:72</td></tr>
<tr><td>OG17636</td><td>70</td><td>1</td><td>+Drosophila_melanogaster|G009199|GNP_001261931.1+Drosophila_melanogaster|G021581|GNP_648849.3:70</td></tr>
<tr><td>OG17794</td><td>67</td><td>1</td><td>+Drosophila_melanogaster|G015861|GNP_524774.1:67</td></tr>
<tr><td>OG18162</td><td>75</td><td>1</td><td>+Drosophila_melanogaster|G007859|GNP_001260537.1+Drosophila_melanogaster|G011726|GNP_001286042.1+Drosophila_melanogaster|G014796|GNP_523595.1+Drosophila_melanogaster|G025038|GNP_724109.1+Drosophila_melanogaster|G025039|GNP_724110.1:75</td></tr>
<tr><td>OG18359</td><td>77</td><td>1</td><td>+Drosophila_melanogaster|G014980|GNP_523794.1+Drosophila_melanogaster|G025907|GNP_725894.1:77</td></tr>
<tr><td>OG19046</td><td>72</td><td>1</td><td>+Drosophila_melanogaster|G020752|GNP_647792.1:72</td></tr>
<tr><td>OG1937</td><td>69</td><td>2</td><td>+Drosophila_melanogaster|G020715|GNP_647746.1+Drosophila_melanogaster|G030211|GNP_996221.1+Drosophila_melanogaster|G030212|GNP_996222.1:62</td></tr>
<tr><td>OG19418</td><td>72</td><td>1</td><td>+Drosophila_melanogaster|G018848|GNP_610024.1:72</td></tr>
<tr><td>OG1953</td><td>83</td><td>1</td><td>+Drosophila_melanogaster|G015463|GNP_524316.1+Drosophila_melanogaster|G028229|GNP_731548.2:83</td></tr>
<tr><td>OG1954</td><td>79</td><td>2</td><td>+Drosophila_melanogaster|G010799|GNP_001285108.1+Drosophila_melanogaster|G016839|GNP_572686.1+Drosophila_melanogaster|G022448|GNP_649938.2+Drosophila_melanogaster|G028180|GNP_731401.3:68</td></tr>
<tr><td>OG19655</td><td>63</td><td>4</td><td>+Drosophila_melanogaster|G005975|GNP_001246908.1+Drosophila_melanogaster|G022084|GNP_649483.1+Drosophila_melanogaster|G027963|GNP_730861.1:29</td></tr>
<tr><td>OG197</td><td>80</td><td>1</td><td>+Drosophila_melanogaster|G003921|GNP_001188532.1+Drosophila_melanogaster|G003922|GNP_001188533.1+Drosophila_melanogaster|G006547|GNP_001259164.1+Drosophila_melanogaster|G006548|GNP_001259165.1+Drosophila_melanogaster|G006549|GNP_001259166.1+Drosophila_melanogaster|G006550|GNP_001259167.1+Drosophila_melanogaster|G014434|GNP_477484.2+Drosophila_melanogaster|G014435|GNP_477485.1+Drosophila_melanogaster|G026326|GNP_726784.1:80</td></tr>
<tr><td>OG19715</td><td>71</td><td>1</td><td>+Drosophila_melanogaster|G011423|GNP_001285737.1+Drosophila_melanogaster|G018149|GNP_609179.2:71</td></tr>
<tr><td>OG1983</td><td>80</td><td>2</td><td>+Drosophila_melanogaster|G011344|GNP_001285657.1+Drosophila_melanogaster|G017991|GNP_608987.1+Drosophila_melanogaster|G019846|GNP_611238.2:71</td></tr>
<tr><td>OG19839</td><td>66</td><td>1</td><td>+Drosophila_melanogaster|G000531|GNP_001027236.1+Drosophila_melanogaster|G004152|GNP_001188778.1:66</td></tr>
<tr><td>OG19883</td><td>73</td><td>1</td><td>+Drosophila_melanogaster|G015605|GNP_524467.1:73</td></tr>
</table>

| | | | |
|---|---|---|---|
| OG19978 | 78 | 1 | +Drosophila_melanogasterlG022365lGNP_649840.1:78 |
| OG19983 | 59 | 1 | +Drosophila_melanogasterlG003273lGNP_001163119.1+Drosophila_melanogasterlG019390lGNP_610691.1:59 |
| OG20031 | 74 | 1 | +Drosophila_melanogasterlG007871lGNP_001260550.2+Drosophila_melanogasterlG015787lGNP_524687.1+Drosophila_melanogasterlG025054lGNP_724149.1:74 |
| OG2006 | 82 | 3 | +Drosophila_melanogasterlG005102lGNP_001245907.1+Drosophila_melanogasterlG007455lGNP_001260117.1+Drosophila_melanogasterlG013711lGNP_476595.1+Drosophila_melanogasterlG022324lGNP_649788.2+Drosophila_melanogasterlG024583lGNP_723137.1+Drosophila_melanogasterlG024584lGNP_723138.1+Drosophila_melanogasterlG024585lGNP_723139.1:69 |
| OG20114 | 72 | 1 | +Drosophila_melanogasterlG015562lGNP_524421.1:72 |
| OG20215 | 77 | 1 | +Drosophila_melanogasterlG015462lGNP_524315.2+Drosophila_melanogasterlG028227lGNP_731544.1:77 |
| OG2044 | 78 | 1 | +Drosophila_melanogasterlG001197lGNP_001036624.1+Drosophila_melanogasterlG012827lGNP_001287151.1+Drosophila_melanogasterlG012828lGNP_001287152.1+Drosophila_melanogasterlG012829lGNP_001287153.1+Drosophila_melanogasterlG015988lGNP_524918.1+Drosophila_melanogasterlG027923lGNP_730774.1+Drosophila_melanogasterlG027924lGNP_730775.1:78 |
| OG20658 | 74 | 1 | +Drosophila_melanogasterlG015980lGNP_524910.1:74 |
| OG20743 | 70 | 1 | +Drosophila_melanogasterlG018689lGNP_609836.1:70 |
| OG2110 | 81 | 2 | +Drosophila_melanogasterlG012716lGNP_001287039.1+Drosophila_melanogasterlG016547lGNP_572308.3+Drosophila_melanogasterlG021317lGNP_648525.1:71 |
| OG21140 | 65 | 1 | +Drosophila_melanogasterlG006731lGNP_001259363.1+Drosophila_melanogasterlG006732lGNP_001259364.1+Drosophila_melanogasterlG006733lGNP_001259365.1+Drosophila_melanogasterlG015766lGNP_524662.1+Drosophila_melanogasterlG026514lGNP_727293.1:65 |
| OG21314 | 82 | 2 | +Drosophila_melanogasterlG028218lGNP_731510.1:74 |
| OG2243 | 64 | 1 | +Drosophila_melanogasterlG022081lGNP_649476.1:64 |
| OG22471 | 80 | 1 | +Drosophila_melanogasterlG011668lGNP_001285984.1+Drosophila_melanogasterlG014183lGNP_477176.1:80 |
| OG22573 | 79 | 5 | +Drosophila_melanogasterlG008553lGNP_001261269.1:62 |
| OG2280 | 75 | 4 | +Drosophila_melanogasterlG030145lGNP_996145.1:49 |
| OG2332 | 78 | 5 | +Drosophila_melanogasterlG003862lGNP_001163778.1+Drosophila_melanogasterlG004633lGNP_001189321.1+Drosophila_melanogasterlG004634lGNP_001189322.1+Drosophila_melanogasterlG004635lGNP_001189323.1+Drosophila_melanogasterlG004636lGNP_001189324.1+Drosophila_melanogasterlG004637lGNP_001189325.1+Drosophila_melanogasterlG004638lGNP_001189326.1+Drosophila_melanogasterlG010346lGNP_001263115.1+Drosophila_melanogasterlG010347lGNP_001263116.1+Drosophila_melanogasterlG010348lGNP_001263117.1+Drosophila_melanogasterlG023911lGNP_651813.2+Drosophila_melanogasterlG029125lGNP_733386.1+Drosophila_melanogasterlG029126lGNP_733387.1+Drosophila_melanogasterlG029127lGNP_733388.1+Drosophila_melanogasterlG029128lGNP_733389.2+Drosophila_melanogasterlG029129lGNP_733390.1:59 |
| OG2366 | 61 | 1 | +Drosophila_melanogasterlG015478lGNP_524331.2:61 |
| OG2424 | 74 | 1 | +Drosophila_melanogasterlG027996lGNP_730935.1:74 |
| OG2429 | 82 | 3 | +Drosophila_melanogasterlG013582lGNP_001303567.1+Drosophila_melanogasterlG016111lGNP_525090.1+Drosophila_melanogasterlG030414lGNP_996459.1+Drosophila_melanogasterlG030415lGNP_996460.1+Drosophila_melanogasterlG030416lGNP_996461.1+Drosophila_melanogasterlG030417lGNP_996462.1:78 |
| OG24382 | 67 | 1 | +Drosophila_melanogasterlG023553lGNP_651364.1:67 |
| OG2478 | 82 | 3 | +Drosophila_melanogasterlG016669lGNP_572452.1:48 |

| | | | |
|---|---|---|---|
| OG2501 | 72 | 1 | +Drosophila_melanogasterlG014473lGNP_511058.1:72 |
| OG2506 | 75 | 4 | +Drosophila_melanogasterlG009600lGNP_001262340.1:56 |
| OG2545 | 75 | 5 | +Drosophila_melanogasterlG010113lGNP_001262872.1:46 |
| OG2575 | 78 | 1 | +Drosophila_melanogasterlG009226lGNP_001261958.1+Drosophila_melanogasterlG015282lGNP_524117.1:78 |
| OG2599 | 80 | 3 | +Drosophila_melanogasterlG024039lGNP_651978.2:44 |
| OG26549 | 60 | 1 | +Drosophila_melanogasterlG001537lGNP_001097135.1:60 |
| OG2685 | 82 | 6 | +Drosophila_melanogasterlG006772lGNP_001259407.1+Drosophila_melanogasterlG010160lGNP_001262919.1+Drosophila_melanogasterlG015621lGNP_524484.1+Drosophila_melanogasterlG015830lGNP_524738.1+Drosophila_melanogasterlG015990lGNP_524921.1+Drosophila_melanogasterlG016002lGNP_524937.1+Drosophila_melanogasterlG026557lGNP_727418.1:72 |
| OG2704 | 82 | 2 | +Drosophila_melanogasterlG016146lGNP_536733.1:77 |
| OG2774 | 82 | 3 | +Drosophila_melanogasterlG010363lGNP_001263132.1+Drosophila_melanogasterlG015714lGNP_524602.1+Drosophila_melanogasterlG029150lGNP_733414.1+Drosophila_melanogasterlG029151lGNP_733415.1:52 |
| OG2986 | 81 | 3 | +Drosophila_melanogasterlG011411lGNP_001285724.1+Drosophila_melanogasterlG011412lGNP_001285725.1+Drosophila_melanogasterlG013884lGNP_476805.1:72 |
| OG3062 | 82 | 3 | +Drosophila_melanogasterlG002937lGNP_001162737.1+Drosophila_melanogasterlG010854lGNP_001285163.1+Drosophila_melanogasterlG010855lGNP_001285164.1+Drosophila_melanogasterlG014549lGNP_511140.1+Drosophila_melanogasterlG026647lGNP_727631.1+Drosophila_melanogasterlG026648lGNP_727632.1:75 |
| OG3080 | 83 | 1 | +Drosophila_melanogasterlG007554lGNP_001260218.1+Drosophila_melanogasterlG011410lGNP_001285723.1+Drosophila_melanogasterlG014259lGNP_477269.1:83 |
| OG3144 | 68 | 1 | +Drosophila_melanogasterlG005010lGNP_001245797.1+Drosophila_melanogasterlG005011lGNP_001245798.1+Drosophila_melanogasterlG011214lGNP_001285526.1+Drosophila_melanogasterlG017560lGNP_608461.1:68 |
| OG3185 | 83 | 1 | +Drosophila_melanogasterlG017917lGNP_608905.1:83 |
| OG3204 | 82 | 2 | +Drosophila_melanogasterlG011177lGNP_001285489.1+Drosophila_melanogasterlG011178lGNP_001285490.1+Drosophila_melanogasterlG013585lGNP_001303570.1+Drosophila_melanogasterlG015886lGNP_524803.1+Drosophila_melanogasterlG026924lGNP_728342.1:71 |
| OG3318 | 77 | 4 | +Drosophila_melanogasterlG008920lGNP_001261640.1+Drosophila_melanogasterlG008921lGNP_001261641.1+Drosophila_melanogasterlG008922lGNP_001261643.1+Drosophila_melanogasterlG008923lGNP_001261644.1+Drosophila_melanogasterlG012688lGNP_001287010.1+Drosophila_melanogasterlG012689lGNP_001287011.1+Drosophila_melanogasterlG012690lGNP_001287012.1+Drosophila_melanogasterlG021184lGNP_648345.1+Drosophila_melanogasterlG027439lGNP_729528.2:65 |
| OG3411 | 82 | 3 | +Drosophila_melanogasterlG009890lGNP_001262639.1+Drosophila_melanogasterlG014403lGNP_477442.1:54 |
| OG3672 | 81 | 3 | +Drosophila_melanogasterlG006647lGNP_001259272.1+Drosophila_melanogasterlG006648lGNP_001259273.1+Drosophila_melanogasterlG006649lGNP_001259274.1+Drosophila_melanogasterlG014476lGNP_511061.1:63 |
| OG375 | 62 | 1 | +Drosophila_melanogasterlG009696lGNP_001262441.1+Drosophila_melanogasterlG022462lGNP_649954.1:62 |
| OG38 | 72 | 2 | +Drosophila_melanogasterlG001411lGNP_001096987.2+Drosophila_melanogasterlG014535lGNP_511124.1:29 |
| OG39 | 76 | 1 | +Drosophila_melanogasterlG001417lGNP_001096993.1+Drosophila_melanogasterlG001418lGNP_001096994.1+Drosophila_melanogasterlG010991lGNP_001285300.1+Drosophila_melanogasterlG014078lGNP_477042.1+Drosophila_melanogasterlG026747lGNP_727901.1+Dro |

| | | | |
|---|---|---|---|
| | | | sophila_melanogaster\|G030407\|GNP_996451.1+Drosophila_melanogaster\|G030408\|GNP_996452.1:76 |
| OG4231 | 67 | 1 | +Drosophila_melanogaster\|G023732\|GNP_651595.1:67 |
| OG4438 | 71 | 1 | +Drosophila_melanogaster\|G013916\|GNP_476848.1:71 |
| OG4500 | 75 | 1 | +Drosophila_melanogaster\|G007679\|GNP_001260349.1+Drosophila_melanogaster\|G013950\|GNP_476888.1:75 |
| OG4615 | 71 | 9 | +Drosophila_melanogaster\|G009046\|GNP_001261772.1:4 |
| OG4636 | 69 | 1 | +Drosophila_melanogaster\|G012193\|GNP_001286511.1+Drosophila_melanogaster\|G013909\|GNP_476838.1:69 |
| OG4733 | 76 | 2 | +Drosophila_melanogaster\|G028553\|GNP_732312.1:74 |
| OG4754 | 73 | 1 | +Drosophila_melanogaster\|G007218\|GNP_001259877.1+Drosophila_melanogaster\|G014046\|GNP_477005.1:73 |
| OG4810 | 72 | 3 | +Drosophila_melanogaster\|G017464\|GNP_608335.1+Drosophila_melanogaster\|G026908\|GNP_728284.1:63 |
| OG4960 | 80 | 1 | +Drosophila_melanogaster\|G012732\|GNP_001287055.1+Drosophila_melanogaster\|G015223\|GNP_524053.2+Drosophila_melanogaster\|G027585\|GNP_729871.1:80 |
| OG5235 | 80 | 3 | +Drosophila_melanogaster\|G025435\|GNP_724887.2+Drosophila_melanogaster\|G025436\|GNP_724888.2:21 |
| OG5237 | 81 | 1 | +Drosophila_melanogaster\|G015819\|GNP_524726.1+Drosophila_melanogaster\|G027101\|GNP_728756.1:81 |
| OG5248 | 76 | 2 | +Drosophila_melanogaster\|G028553\|GNP_732312.1:74 |
| OG5265 | 82 | 1 | +Drosophila_melanogaster\|G007634\|GNP_001260303.1+Drosophila_melanogaster\|G011471\|GNP_001285785.1+Drosophila_melanogaster\|G013939\|GNP_476874.1:82 |
| OG5408 | 79 | 1 | +Drosophila_melanogaster\|G015215\|GNP_524043.1:79 |
| OG5777 | 81 | 2 | +Drosophila_melanogaster\|G014487\|GNP_511073.1:76 |
| OG5834 | 76 | 5 | +Drosophila_melanogaster\|G011117\|GNP_001285428.1:62 |
| OG5895 | 79 | 2 | +Drosophila_melanogaster\|G014991\|GNP_523805.1:77 |
| OG6030 | 72 | 2 | +Drosophila_melanogaster\|G006454\|GNP_001259063.1:70 |
| OG6174 | 76 | 1 | +Drosophila_melanogaster\|G018369\|GNP_609444.1:76 |
| OG627 | 79 | 6 | +Drosophila_melanogaster\|G009839\|GNP_001262586.1+Drosophila_melanogaster\|G010830\|GNP_001285139.1+Drosophila_melanogaster\|G012098\|GNP_001286414.1+Drosophila_melanogaster\|G014542\|GNP_511132.2+Drosophila_melanogaster\|G014928\|GNP_523741.2+Drosophila_melanogaster\|G015233\|GNP_524063.1+Drosophila_melanogaster\|G015486\|GNP_524339.1+Drosophila_melanogaster\|G015502\|GNP_524356.1+Drosophila_melanogaster\|G015611\|GNP_524474.1+Drosophila_melanogaster\|G015883\|GNP_524798.2+Drosophila_melanogaster\|G015993\|GNP_524927.2+Drosophila_melanogaster\|G022659\|GNP_650209.1+Drosophila_melanogaster\|G026626\|GNP_727563.1+Drosophila_melanogaster\|G026627\|GNP_727564.1+Drosophila_melanogaster\|G026628\|GNP_727565.1+Drosophila_melanogaster\|G027609\|GNP_729940.1+Drosophila_melanogaster\|G027610\|GNP_729941.1+Drosophila_melanogaster\|G028271\|GNP_731651.1+Drosophila_melanogaster\|G028296\|GNP_731716.1+Drosophila_melanogaster\|G028415\|GNP_731987.1+Drosophila_melanogaster\|G028416\|GNP_731988.1+Drosophila_melanogaster\|G028417\|GNP_731989.1+Drosophila_melanogaster\|G029548\|GNP_788663.1+Drosophila_melanogaster\|G029562\|GNP_788679.1+Drosophila_melanogaster\|G029563\|GNP_788680.1:69 |
| OG6367 | 83 | 1 | +Drosophila_melanogaster\|G010802\|GNP_001285111.1+Drosophila_melanogaster\|G010803\|GNP_001285112.1+Drosophila_melanogaster\|G024031\|GNP_651969.1+Drosophila_melanogaster\|G026596\|GNP_727499.1:83 |
| OG6853 | 83 | 3 | +Drosophila_melanogaster\|G014179\|GNP_477171.1+Drosophila_melanogaster\|G029262\|GNP_788057.2:72 |
| OG6865 | 77 | 1 | +Drosophila_melanogaster\|G004154\|GNP_001188780.1:77 |

| | | | |
|---|---|---|---|
| OG7 | 62 | 1 | +Drosophila_melanogasterlG019427lGNP_610735.1:62 |
| OG7027 | 79 | 1 | +Drosophila_melanogasterlG014178lGNP_477170.1+Drosophila_melanogasterlG017433lGNP_599137.1:79 |
| OG7268 | 77 | 1 | +Drosophila_melanogasterlG007157lGNP_001259814.1+Drosophila_melanogasterlG014208lGNP_477208.1:77 |
| OG7313 | 76 | 4 | +Drosophila_melanogasterlG003705lGNP_001163604.1+Drosophila_melanogasterlG029555lGNP_788670.1:52 |
| OG7350 | 79 | 1 | +Drosophila_melanogasterlG008043lGNP_001260732.1+Drosophila_melanogasterlG014119lGNP_477090.1:79 |
| OG7467 | 68 | 1 | +Drosophila_melanogasterlG028983lGNP_733208.1:68 |
| OG7628 | 78 | 1 | +Drosophila_melanogasterlG011553lGNP_001285869.1+Drosophila_melanogasterlG014180lGNP_477172.1:78 |
| OG7696 | 80 | 2 | +Drosophila_melanogasterlG011049lGNP_001285360.1+Drosophila_melanogasterlG011050lGNP_001285361.1+Drosophila_melanogasterlG011051lGNP_001285362.1+Drosophila_melanogasterlG014605lGNP_523382.1:79 |
| OG7796 | 79 | 1 | +Drosophila_melanogasterlG006396lGNP_001247362.1+Drosophila_melanogasterlG013266lGNP_001287596.1+Drosophila_melanogasterlG013267lGNP_001287597.1+Drosophila_melanogasterlG023853lGNP_651740.1:79 |
| OG7828 | 81 | 1 | +Drosophila_melanogasterlG008183lGNP_001260878.1+Drosophila_melanogasterlG019336lGNP_610630.1:81 |
| OG7843 | 78 | 2 | +Drosophila_melanogasterlG021155lGNP_648308.1:7 |
| OG791 | 68 | 3 | +Drosophila_melanogasterlG009839lGNP_001262586.1+Drosophila_melanogasterlG010830lGNP_001285139.1+Drosophila_melanogasterlG012098lGNP_001286414.1+Drosophila_melanogasterlG014542lGNP_511132.2+Drosophila_melanogasterlG014928lGNP_523741.2+Drosophila_melanogasterlG015233lGNP_524063.1+Drosophila_melanogasterlG015486lGNP_524339.1+Drosophila_melanogasterlG015502lGNP_524356.1+Drosophila_melanogasterlG015611lGNP_524474.1+Drosophila_melanogasterlG015883lGNP_524798.2+Drosophila_melanogasterlG015993lGNP_524927.2+Drosophila_melanogasterlG022659lGNP_650209.1+Drosophila_melanogasterlG026626lGNP_727563.1+Drosophila_melanogasterlG026627lGNP_727564.1+Drosophila_melanogasterlG026628lGNP_727565.1+Drosophila_melanogasterlG027609lGNP_729940.1+Drosophila_melanogasterlG027610lGNP_729941.1+Drosophila_melanogasterlG028271lGNP_731651.1+Drosophila_melanogasterlG028296lGNP_731716.1+Drosophila_melanogasterlG028415lGNP_731987.1+Drosophila_melanogasterlG028416lGNP_731988.1+Drosophila_melanogasterlG028417lGNP_731989.1+Drosophila_melanogasterlG029548lGNP_788663.1+Drosophila_melanogasterlG029562lGNP_788679.1+Drosophila_melanogasterlG029563lGNP_788680.1:61 |
| OG8046 | 83 | 1 | +Drosophila_melanogasterlG010802lGNP_001285111.1+Drosophila_melanogasterlG010803lGNP_001285112.1+Drosophila_melanogasterlG024031lGNP_651969.1+Drosophila_melanogasterlG026596lGNP_727499.1:83 |
| OG8056 | 81 | 3 | +Drosophila_melanogasterlG015666lGNP_524533.1+Drosophila_melanogasterlG028995lGNP_733222.1+Drosophila_melanogasterlG028996lGNP_733223.1:46 |
| OG8066 | 81 | 3 | +Drosophila_melanogasterlG008531lGNP_001261247.1+Drosophila_melanogasterlG013998lGNP_476950.1:68 |
| OG8215 | 72 | 1 | +Drosophila_melanogasterlG021380lGNP_648603.1:72 |
| OG8244 | 82 | 1 | +Drosophila_melanogasterlG013746lGNP_476631.1+Drosophila_melanogasterlG029972lGNP_995941.1:82 |
| OG8257 | 68 | 1 | +Drosophila_melanogasterlG018336lGNP_609402.1:68 |
| OG845 | 79 | 4 | +Drosophila_melanogasterlG005196lGNP_001246015.1+Drosophila_melanogasterlG007751lGNP_001260424.1+Drosophila_melanogasterlG007752lGNP_001260425.1+Drosophila_melanogasterlG007753lGNP_001260426.1+Drosophila_melanogasterlG014762lGNP_523560.2+Drosophila_melanogasterlG018489lGNP_609595.1+Drosophila_melanogasterlG024055lGNP_652004.2+Drosophila_melanogasterlG02486 |

| | | | |
|---|---|---|---|
| | | | 2lGNP_723775.1+Drosophila_melanogasterlG024863lGNP_723776.1:67 |
| OG847 | 58 | 1 | +Drosophila_melanogasterlG014529lGNP_511118.2:58 |
| OG8481 | 81 | 1 | +Drosophila_melanogasterlG014124lGNP_477098.1+Drosophila_melanogasterlG017431lGNP_599135.1+Drosophila_melanogasterlG017432lGNP_599136.1+Drosophila_melanogasterlG025743lGNP_725524.1+Drosophila_melanogasterlG029906lGNP_995849.1+Drosophila_melanogasterlG029907lGNP_995850.1:81 |
| OG85 | 78 | 1 | +Drosophila_melanogasterlG007180lGNP_001259837.1+Drosophila_melanogasterlG017613lGNP_608534.2:78 |
| OG8590 | 74 | 2 | +Drosophila_melanogasterlG023530lGNP_651330.1:72 |
| OG8624 | 80 | 1 | +Drosophila_melanogasterlG009555lGNP_001262295.1+Drosophila_melanogasterlG022149lGNP_649568.1:80 |
| OG8657 | 77 | 1 | +Drosophila_melanogasterlG012900lGNP_001287225.1+Drosophila_melanogasterlG012901lGNP_001287226.1+Drosophila_melanogasterlG022307lGNP_649769.1:77 |
| OG8718 | 72 | 1 | +Drosophila_melanogasterlG016656lGNP_572438.1+Drosophila_melanogasterlG026496lGNP_727233.1+Drosophila_melanogasterlG026497lGNP_727234.1:72 |
| OG8850 | 81 | 1 | +Drosophila_melanogasterlG014124lGNP_477098.1+Drosophila_melanogasterlG017431lGNP_599135.1+Drosophila_melanogasterlG017432lGNP_599136.1+Drosophila_melanogasterlG025743lGNP_725524.1+Drosophila_melanogasterlG029906lGNP_995849.1+Drosophila_melanogasterlG029907lGNP_995850.1:81 |
| OG8895 | 83 | 3 | +Drosophila_melanogasterlG001973lGNP_001097667.1+Drosophila_melanogasterlG001974lGNP_001097668.2+Drosophila_melanogasterlG009486lGNP_001262225.1+Drosophila_melanogasterlG014003lGNP_476955.1+Drosophila_melanogasterlG027909lGNP_730757.1+Drosophila_melanogasterlG027910lGNP_730758.1+Drosophila_melanogasterlG027911lGNP_730759.1+Drosophila_melanogasterlG027912lGNP_730760.1:80 |
| OG9020 | 80 | 2 | +Drosophila_melanogasterlG004370lGNP_001189023.1+Drosophila_melanogasterlG008579lGNP_001261295.1+Drosophila_melanogasterlG013925lGNP_476857.1:77 |
| OG9104 | 79 | 2 | +Drosophila_melanogasterlG012686lGNP_001287008.1+Drosophila_melanogasterlG015180lGNP_524004.2+Drosophila_melanogasterlG027431lGNP_729506.1:75 |
| OG9191 | 58 | 1 | +Drosophila_melanogasterlG014457lGNP_477509.1:58 |
| OG9251 | 67 | 1 | +Drosophila_melanogasterlG014280lGNP_477296.1+Drosophila_melanogasterlG024794lGNP_723586.1+Drosophila_melanogasterlG024795lGNP_723587.1:67 |
| OG9287 | 83 | 4 | +Drosophila_melanogasterlG014938lGNP_523751.2+Drosophila_melanogasterlG025703lGNP_725452.1+Drosophila_melanogasterlG025704lGNP_725453.1+Drosophila_melanogasterlG025705lGNP_725454.1+Drosophila_melanogasterlG025706lGNP_725455.1:58 |
| OG9408 | 83 | 3 | +Drosophila_melanogasterlG006478lGNP_001259087.1+Drosophila_melanogasterlG015740lGNP_524631.1:61 |
| OG9477 | 76 | 2 | +Drosophila_melanogasterlG015096lGNP_523917.2:2 |
| OG9573 | 76 | 1 | +Drosophila_melanogasterlG018775lGNP_609936.1:76 |
| OG9600 | 83 | 3 | +Drosophila_melanogasterlG007692lGNP_001260362.1+Drosophila_melanogasterlG011498lGNP_001285814.1+Drosophila_melanogasterlG014150lGNP_477137.1+Drosophila_melanogasterlG024808lGNP_723616.1:72 |
| OG9650 | 75 | 1 | +Drosophila_melanogasterlG028731lGNP_732717.1+Drosophila_melanogasterlG028732lGNP_732718.1+Drosophila_melanogasterlG028733lGNP_732719.1+Drosophila_melanogasterlG030243lGNP_996265.1:75 |
| OG9660 | 79 | 1 | +Drosophila_melanogasterlG012294lGNP_001286614.1+Drosophila_melanogasterlG014089lGNP_477054.1:79 |
| OG9704 | 73 | 1 | +Drosophila_melanogasterlG021066lGNP_648201.1:73 |
| OG9778 | 70 | 1 | +Drosophila_melanogasterlG005301lGNP_001246149.1+Drosophila_melanogasterlG018997lGNP_610224.1:70 |

| | | | |
|---|---|---|---|
| OG9785 | 83 | 3 | +Drosophila_melanogaster\|G015263\|GNP_524098.2:65 |
| OG9939 | 79 | 1 | +Drosophila_melanogaster\|G017650\|GNP_608575.1:79 |

**Table S9.** Chimeric terminals created for fusion of Matrix 3 with published morphological matrices.

*Morphological matrix of Bicknell et al. (2019):*

Pycnogonum_litorale = Pycnogonum_litorale
Limulus_polyphemus = Limulus_polyphemus
Tachypleus_gigas = Tachypleus_gigas
Tachypleus_tridentatus = Tachypleus_tridentatus
Carcinoscorpius_rotundicauda = Carcinoscorpius_rotundicauda
Centruroides_vittatus = Centruroides_sculpturatus
Androctonus_australis = Androctonus_australis
Caddo_agilis = Caddo agilis
Chileogovea_oedipus = Chileogovea_oedipus
Cyphophthalmus_duricorius = Siro_boyerae
Leiobunum_rotundum = Leiobunum_verrucosum
Gonyleptes_horridus = Gonyleptes_fragilis
Sclerobunus_robustus = Sclerobunus_robustus
Neobisium_maritimum = Novobisium_sp
Chelifer_cancroides = Cheliferidae_sp
Eremocosta_striata = Eremobates_sp
Galeodes_armeniacus = Galeodes_sp
Eukoenenia_mirabilis = Espelaea_UWisc
Neocarus_texanus = Neoacarus_sp
Amblyomma_americanum = Amblyomma_americanum
Allothrombium_simoni = Dinothrombium_tinctorium
Cryptocellus_goodnighti = Cryptocellus_sp_IZ143922
Ricinoides_atewa = Ricinoides_atewa
Liphistius_malayanus = Liphistius_malayanus
Aphonopelma_hentzi = Aphonopelma_hentzi
Hypochilus_pococki = Hypochilus_pococki
Cupienius_getazi = Cupiennius_sp
Phrynus_marginemaculatus = Phrynus_marginemaculatus
Charinus_victori = Charinus_ioaniticus
Mastigoproctus_giganteus = Mastigoproctus_giganteus
Schizomus_crassicaudatus = Stenochrus_portoricensis

*Morphological matrix of Siveter et al. (2018):*

Peripatus = Epiperipatus sp.
Euperipatoides = Opisthopatus kwazululandi
Ammothella = Tanystylum orbiculare
Limulus = Limulus polyphemus
Carcinoscorpius = Carcinoscorpius rotundicauda

Centruroides = (Centruroides sculpturatus)
Heterometrus = Pandinus imperator
Caddo = Caddo agilis
Nipponopsalis = Trogulus
Siro = Siro
Equitius = Larifuga
Idiogaryos = Sternophoridae
Eremocosta = Eremobates_sp
Amblyomma = Amblyomma_americanum
Archegozetes = Archogozetes_longisetosus
Opilioacarus = Adenacarus_sp
Cryptocellus = Cryptocellus_sp_IZ-143922
Prokoenenia = Espelaea_UWisc
Liphistius = Liphistius_malayanus
Aphonopelma = Aphonopelma_hentzi
Cupienius = Cupiennius_sp
Stenochrus = Stenochrus_portoricensis
Mastigoproctus = Mastigoproctus_giganteus
Phrynus = Phrynus marginemaculatus
Scutigera = Scutigera
Lithobius = Lithobius
Craterostigmus = Craterostigmus
Scolopendra = Alipes
Pachymerium = Strigamia
Scutigerella = Symphylella
Pauropus = Pauropus
Narceus = Narceus
Vargula = Eusarsiella
Daphnia = Daphnia_pulex
Calanus = Eurytemora
Orchestia = Hyalella
Tomocerus = Folsomia
Locusta = Gryllus
Drosophila = Drosophila

**File S1. Morphological characters and states.**

1 Number of head shield segments
states: five [cephalosoma/proterosoma]; seven [prosomal shield]
2 Ophthalmic ridges
states: absent; present.
3 Pleural margin of prosomal shield
states: absent; present
4 Cardiac lobe
states: absent; present
5 Prosomal repugnatorial glands
states: absent present,
6 Ozophores
states: absent; present
7 Cucullus
states: absent; present
8 Width of sternal region
states: broad throughout; narrow anteriorly; narrow posteriorly; narrow throughout
9 Division of prosomal sternum
states: undivided; divided
10 Cephalic doublure
states: absent; present
11 Lines demarcating meso- and metapeltidium on the prosomal shield
states: absent; present
12 Genal spines
states: absent; present
13 Anterior median projection of prosomal dorsal shield
states: absent; present
14 Trapezoidal projection of anterior carapace margin
states: absent; present
15 Keels between the median and lateral eyes on the prosomal dorsal shield
states: absent; present
16 Proboscis
states: absent; present,
17 Direction of mouth opening
states: directed anteroventrally; directed posteroventrally
18 Labium or tritosternum
states: absent; present
19 Epistomal-labral plate
states: absent; present
20 Formation of the ventroposterior wall of pre-oral chamber
states: formed by labium; formed by palpal coxae
21 Pedipalpal endite outgrown,

states: absent; present
22 Fused pedipalpal endites
states: absent; present
23 Leg 1 endite outgrown
states: absent; present
24 Fused leg 1 endites
states: absent; present
25 Leg 2 endite outgrown
states: absent; present
26 Fused leg 2 endites
states: absent; present
27 Palate plate
states:  absent; present
28 Filtering preoral setae
states: absent; present
29 Three-branched epistomal skeleton
states: absent; present
30 Intercheliceral epipharyngeal sclerite
states: absent; present
31  Epipharyngeal sclerite
states: absent; large, projecting posteriorly
32 Metasoma
states:  absent; present
33 Prosoma and opisthosoma form a single functional unit
states:  absent; present
34 Metasoma length
states: pygidium; five segments; nine segments
35 Well-developed post-anal telson
states:  absent; present,
36 Pygidium transformed into anal tubercle
states: absent; present,
37 Presence absence of flagellate telson
states: absent; present,
38 Vesicle and aculeus on the telson
states: absent; present
39 Specialized male postanal flagellum
states: absent; present,
40 Number_of_deutocerebral_appendage_articles,
states:  more than 3; 3; 2
41 Basal suture in proximal cheliceral segment
states: absent; present
42 Cheliceral_PD_axis_with_point_of_inflection
states: absent; present
43 Cheliceral teeth

states: absent; present
44 Distal-most cheliceral tooth
states: 1; 2
45 Position of the cheliceral apotele articulation
states: articulates ventrally; articulates dorsally; articulates laterally
46 Distal chelicera type
states: chelate 'clasp-knife' 'type'; Prostigmata styliform or *Anystys*-like chelicerae,
47 Diaphanous cheliceral teeth
states: absent; present
48 Angle of cheliceral articulation
states: parallel to sagittal plane; oblique to sagittal plane; parallel to transverse plane; parallel to coronal plane
49 Chelicerae project beyond anterior prosomal shield margin
states: absent; present
50 Naked cheliceral fang
states: absent; present
51 Plagula ventralis
states: absent; present
52 Cheliceral venom gland;
states: absent; present
53 Endocephalic spinning apparatus
states: absent; present
54 Cheliceral flagellum
states: absent; present
55 Chelicero-carapacal articulation
states: absent; present
56 Mesal fusion of chelicerae
states: absent; chelicerae proximally fused
57 Shape of movable digit of chelicerae
states: *Anystis* type (Acarifomes); styliform,
58 Cheliceral serrula
states: absent; present
59 Morphology of the cheliceral serrula
states: rounded; tooth-like; hyaline
60 Presence/absence cheliceral brush
states: absent; present
61 Fusion of palpal coxae
states: free; fused medially
62 Gnathosoma
states: absent; present
63 Subcapitular rutella
states: absent; present
64 Morphology of palpal chelae

states: leg like; subraptorial chelate; scorpionoid
65 Apophyses on patella and tibia of pedipalp
states: absent; present
66 Modified patellar apophysis of male pedipalp
states: absent; present
67 Plane of motion of pedipalps
states: vertical; horizontal
68 Prominent dorsal flange on the pedipalp trochanter
states: absent; present
69 Dorsal row of femoral spines
states: absent; present
70 Presence/absence ventral apophysis on palpal trochanter
states: absent; present
71 Spine-like ventral apophysis on palpal trochanter
states: absent; present
72 Palpal cleaning organ
states: absent; present
73 Pedipalpal venom glands
states: absent; present
74 Fusion level of palpal apotele
states: differentiated from tarsus; not differentiated from tarsus,
75 Adhesive palpal organ
states: absent; present
76 Dorsal row of spines on pedipalp patella
states: absent; present
77 Three principal spines on pedipalp patella with spine 1 large
states: absent; present
78 Patellar spines decreasing in length proximally
states: absent; present
79 Reduced proximal spine from three-spined palpal patella'
states: absent; present
80 Pedipalpal megaspines forming a distal catching basket
states: absent; present (phrynicid hand)
81 Spines on palpal tarsus
states: absent; present
82 Fusion of palpal tibia and tarsus
states: unfused; fused
83 Ovigers
states: absent present,
84 Gnathobases
states: present absent,
85 Elongated (antenniform) first leg
states: absent present,
86 Leg 1 sternocoxal articulation

states: absent present,
87 Leg 1 apotele
states: present; absent
88 Segmentation of leg 1 tibia
states: unmodified; 16 segments ;up to 23 segments; 25 segments; more than 25 segments
89 Elongation of leg 2
states: unmodified; elongate
90 Enlargement of coxa 2
states: unmodified; enlarged
91 Presence of exopods
states: retained on more than one prosomal limb; on sixth prosomal limb; absent,
92 Morphology of coxo-trochanteral joint
states: simple; complex
93 Femoral division in legs 3 and 4
states: present; absent
94 Types of femoropatella articulation
states: transverse hinge; bicondylar articulation; monocondylar articulation,
95 Type of patellotibial articulation
states: monocondylar; hinge bicondylar
96 Auxiliary posterior articulation on patellatibial articulation
states: absent; present
97 Appendages of post-oral somites III–V with fused tibia and tarsus
states: absent; present
98 Division of tarsus
states: tarsus divided into basi- and telotarsus; tarsus undivided
99 Metatarsus at least ca. 1.5 times tarsus length
states: absent; present,
100 Telotarsi of walking legs 2–4 with three tarsomeres
states: absent; present
101 Apotele morphology
states: a simple cone or blade; a medial piece, comprising a claw or pulvillus; more frequently bearing a pair of lateral claws
102 Pulvillus
states: absent; present
103 Chelate legs
states: absent; present
104 Divided claws
states: absent; present
105 Single tarsal claws on legs 1 and 2 and double tarsal claws on legs 3 and 4
states: absent; present
106 Plate-like opisthosomal appendages
states: absent; present
107 Limb VI

states: unmodified; pusher; paddle
108 Number of body segments
states: twenty; nineteen; eighteen; seventeen; sixteen; fifteen; fourteen; thirteen; twelve; eight segments or less
109 Width of prosoma-opisthosoma junction
states: broad xiphosuran cephalothorax; narrow (pedicel)
110 Length of first opisthosomal tergite
states: unmodified; very short
111 Six abbreviated opisthosomal tergites
states: absent; present
112 Opisthosomal sternite 1
states: present; absent
113 Triangular opisthosomal sternite 1
states: present; absent
114 Locking mechanism between opisthosoma and prosoma
states: absent; present
115 Fusion of opisthosomal tergites 7-10
states: separate; fused into a single plate
116 Opisthosomal tergites with a distinct axial region (cf. trilobite)
states: absent; present
117 Division on posterior opisthosomal tergites
states: absent; present
118 Fused opisthosomal tergites
states: absent; present
119 Fused opisthosomal tergites medially divided
states: absent; present
120 Number of diplotergites
states: absent; one; more than one
121 Fused opisthosomal segments
states: absent; buckler thoracatron,
122 Appendages on opisthosomal segment 1
states: present; absent
123 Median abdominal appendage
states: absent; present
124 Genital operculum formed by the fusion of the two anterior-most abdominal appendages
states: absent; present
125 Appendages on opisthosomal segments at any developmental stage
states: absent; present
126 Overlap between the genital operculum and the sternite of third opisthosomal segment
states: absent; present
127 Opisthosomal silk glands and spigots
states: absent; present

128 Opisthosomal spinnerets
states: absent; present
129 Pygidial defensive secretions
states: absent; present
130 Genital acetabula
states: absent present
131 Number of genital acetabula
states: three; two; more than three
132 Presence/absence dorsal anal operculum
states: absent; present
133 Lateral eyes
states: absent; present
134 Morphology of lateral eye lenses
states: compound; five or more pairs of lenses; three primary pairs [excluding any microlenses]; two pairs; one pair; no lenses
135 Arrangement of lateral eye rhabdomeres in cross-section
states: net-like; star-shaped
136 Number of median eyes
states: four; two or three; absent
137 Organization of retinula cells of median eyes
states: organized into closed rhabdomes; organized into a network of rhabdomeres; disorganized inverse retina
138 Slit sense organs
states: absent; present
139 Trichobothria
states: absent; present
140 Tibial trichobothria with 2-1-1-1 distribution
states: absent; present
141 Pro-dorsal trichobothria
states: absent; present,
142 Pectines
states: absent; present
143 Malleoli
states: absent; present
144 Tarsal organ on leg I
states: absent; present
145 Tarsal organ on leg II
states: absent; present
146 Opisthosomal ganglia in adults
states: absent; present
147 Perineural membrane enveloping arterial sinus
states: present; absent
148 Intercheliceral median organ
states: absent; present

149 Filamentous gills
states: absent; present
150 Lamellate breathing structures; book gills or lungs.
states: absent; present
151 Tubular tracheae
states: absent; present
152 Book lung/gill on 2nd (i.e. genital) opisthosomal segment
states: present; absent
153 Book lung/gill on 3rd (i.e. postgenital) opisthosomal segment
states: present; absent,
154 Book lung/gill on 4th to 7th opisthosomal segment
states: present absent,
155 Stigmata
states: absent; present
156 Spines on book lung lamellar margins
states: absent; present
157 Shape of pillars of the haemolymph spaces inside the gill/lung lamellae'
states: at least two perikarya meeting midway in the haemolymph space; pillars including a strong axis of microtubules
158 Location of prosomal spiracles
states: absent; between the coxae of the second and third walking legs; associated with coxae of third and fourth walking leg; between cheliceral basis; brachypyline oribatid tracheal system
159 Location of opisthosomal spiracles
states: absent; paired ventral stigmata on genital segment; paired ventral stigmata on 3rd and 4th opisthosomal segments; four pairs of dorsal stigmata on the anterior opisthosoma
160 Kiemenplatten
states: absent; present
161 Postcerebral crop and proventriculus
states: reduced; present
162 Well-developed sucking stomach
states: absent; present
163 Endosternite
states: absent; present
164 Termination of anterior endosternal horn
states: terminating in muscular attachment to labrum; terminating in muscular attachment to palpal coxa
165 Fenestrate endosternite
states: absent; present
166 Malpighian tubules
states: absent; present
167 Coxal glands opening on proximal podomere of chelifore
states: absent; present

168 Presence/absenece of sacculus associated to coxal gland
states: absent; present
169 Coxal gland opening at the base of pedipalp,
states: absent; present
170 Coxal glands opening at base of leg 1
states: absent; present
171 Coxal glands opening on leg 3 segment
states: absent; present
172 Contribution of the coxal gland to saliva
states: absent; Buxtons group II coxal gland; coxal glands and saliva converging into the pre-oral chamber through external taenidia or gutters; podocephalic channel
173 Dorsomedian excretory organ
states: absent; present
174 Origin of lateral extrinsic precerebral pharyngeal muscle
states: arising from anterior endosternal horns; arising from medial surface of palpal coxae
175 Ventral extrinsic precerebral pharyngeal muscle and tergopharyngeal muscle of precerebral pharynx
states: absent; present
176 cheliceral tergal-deutomerite muscle
states: absent; present
177 Number of heads of lateral tergocheliceral muscle
states: one head; three heads
178 Paired muscle arising from posterior margin of anterior carapacal doublure and inserting on prosomal shield
states: absent; present
179 Posterior oblique muscles of box-truss axial muscle system (BTAMS) of postoral somites I–V
states: absent; present in one or more somites
180 Anterior oblique muscles of BTAMS posterior to postoral somite VI
states: absent; present
181 Intercoxal endosternal extensor muscles
states: absent; present
182 Endosternal dorsal suspensors of somites I and II
states: absent; present
183 Endosternal dorsal suspensor muscles in somite four with anterolateral carapacal insertion
states: absent; present
184 Endosternal dorsal suspensor muscle of somite five
states: absent; present
185 Attachment of ventral endosternal suspensor muscles
states: attaching primarily to sternum; attaching primarily to coxa of appendage of anteriorly adjacent somite

186 Palpal posteromedial tergocoxal muscle
states: present absent
187 Origin of palpal posteromedial endosternocoxal muscle
states: originates on endosternite, inserts on coxa; originates and inserts on coxa
188 Intracoxal muscle
states: absent; present
189 Size of insertion process of anteromedial tergocoxal muscle
states: weakly developed; large, well developed
190 Symmetry of femoropatellar flexor
states: symmetrical; asymmetrical
191 Insertion of pedal anterior femur–patella muscle
states: inserting primarily on patellar margin; inserting primarily on patellar plagula
192 Pedal posterior femeropatella-tibia muscle
states: present; absent
193 Pedal patellotibia-tarsus muscle
states: present; absent
194 Posterior transpatellar muscles insertion
states: 'dorsoposterior femur/posterior patella'; distal process of femur absent
195 Atellotibial extensor
states: absent; present
196 Position of the insertion of anterior transpatellar muscle
states: anterior/anteroventral; ventral/posteroventral; absent
197 Position of the anterior patellotibial muscle on tibia
states: anterior; ventral; absent
198 Posterior patellotibial muscle
states: absent; present
199 Origin of apotele depressor
states: tarsus; tibia
200 Patellar head of apotele depressor
states: absent; present
201 Origin on posterior patellar wall of the patellar head of apotele depressor
states: absent; present
202 Position of the attachments of opisthosomal posterior oblique axial muscles
states: tergal; pleural
203 Organization of opisthosomal pleural muscle
states: continuous; dorsoventral sheet divided into three components
204 Dorsal and ventral longitudinal muscles
states: spanning full length of opisthosoma; spanning first and last four opisthosomal somites
205 Internal fertilization
states: absent; present
206 Gonopore position
states: on limb bases; on second opisthosomal segment,

207 Gonopores on second opisthosomal segment
states: paired; unpaired
208 Anteriorly positioned gonopore
states: absent; present
209 Intromittent organ associated with genital region
states: absent; present
210 Penis/spermatopositor
states: spermatopositor; penis; acariforme aedagus
211 Male palpal organ
states: absent; present
212 Sperm transfer organ on leg 3
states: absent; present
213 Ovipositor
States: absent; present
214 Stalked spermatophore
states: absent; present
215 Spermatophore uptake
states: without mating; face-to-face uptake; mating parade,
216 Testis
states: glandular area unspecialised; glandular area distinctly larger
217 Tubular genital accessory glands
states: absent; present
218 Brood sac
states: absent; present
219 Sperm: Flagellum in the mature sperm
states: absent; present
220 Sperm: Coiled sperm cells
states: absent; present
221 Sperm: Acrosomal filament
states: absent; present
222 Sperm: Coiled acrosomal filament
states: absent; present
223 Sperm: Acrosomal filament piercing nucleus
ates: present; absent
224 Sperm: Microtubule arrangement in axoneme
states: 9+0; 9+1; 9+2; 9+3
225 Sperm: Helical nucleus
states: absent; present
226 Sperm: Shape of postcentriolar nucleus
states: unmodified; asymmetrical; elongate
227 Sperm: Depth of fossa implantation
states: shallow; deep
228 Sperm: Manchette of microtubules
states: absent; present

229 Sperm: Nuclear envelope
states: absent; present
230 Sperm: Persisting flagellar tunnel
states: absent; present
231 Sperm: Vacuolated sperm cells
states: absent; present
232 Yolk distribution of eggs
states: centrolecithal; isolecithal; telolecithal
233 Anatomical fate of embryonic growth zone
states: gives rise to prosoma and opisthosoma; gives rise to opisthosoma only
234 Hexapodal first instar
states: absent; present
235 Egg teeth on pedipalpal coxae
states: absent; present
236 Embryonic lateral organs
states: absent; present
237 Heteromorphic parasitic larvae
states: absent; present
238 Modified dispersal larval stage (hypopus)
states: absent; present
239 Anamorphic development with protonymphal stage
states: absent; present
240 Male oviger segments
states: 11; 10; 9; 7; 6; 4
241 Female oviger
states: absent; present
242 Terminal claw of oviger
states: absent; present
243 Auxiliary claws of propodus
states: absent; present
244 Cement glands
states: absent; present on femora and other podomeres
245 Abdominal rudiment
states: horizontal, parallel to trunk; erect diagonally or dorsally
246 Shape of proboscis
states: cylindrical; inflated proximally, acute distally; inflated distally; tapering or pipette-like
247 Proboscis position
states: frontal and fixed; in angle and movable; ventral and movable
248 Gonostome
states: closed; open
249 Ocularium
states: absent; present
250 Oviposition and copulatory ducts separate

states: absent; separated
251 Segmentation of anterior lateral spinnerets
states: indistinct; segments clearly defined
252 Segmentation of posterior lateral spinnerets
states: indistinct; up to four distinct segments; more than four distinct segments
253 Pedipalpal trichobothria type
states: Type A; Type B; Type C; Type D
254 Egg laying
states: absent; present
255 Vivaparous embryogenesis
states: apoikogenic; katoikogenic
256 Hemolymph vascular system
states: absent; present
257 Perineural vascular sheath
states: absent; present
258 R-cell terminals from lateral eyes and median eyes overlapping
states: absent; present
259 Arcuate body located apart from neuropils
states: absent; present

**File S2.** Morphological character matrix in Nexus format.

```
#NEXUS

BEGIN TAXA;
	TITLE Taxa;
	DIMENSIONS NTAX=517;
	TAXLABELS
		Haliestes Palaeopantopus Palaeoisopus Flagellopantopus Dibasterium
Offacolus Weinbergina Attercopus_fimbriunguis Permarachne_novokshonovi
Chimerarachne_yingi Idmonarachne Parastylonurus Mixopterus Eurypterus
Chasmataspis Octoberaspis Waeringoscorpio Pulmonoscorpius Palaeoscorpius
Compsoscorpius Proscorpius Palaeocharinus Anthracomartus Eophrynus Plesiosiro
Graeophonus Paracharonopsis Kronocharon Eophalangium Hastocularis Pycno
'Tanystylum_orbiculare_P02' Anoplo 'Phoxichilidium_tubilariae_P07'
'Nymphon_molleri_P06' 'Meridionale_flava_P04' 'Stylopallene_cheilorynchus_P05'
'Labidostommatidae_IZ-67347' 'Dinothrombium_tinctorium'
'Panonychus_citri_SRR341928' 'Tetranychus_cinnabarinus_SRR519097'
'Tetranychus_urticae' 'Rhizoglyphus_robini_SRR946953' 'Sarcoptes_scabei'
'Dermatophagoides_farinae_SRR1016494' 'Dermatophagoides_pteronyssinus'
'Euroglyphus_maynei' 'Hypochithonius_rufulus_SRR4039020'
'Steganacarus_magnus_SRR4039023' 'Achipteria_coleoptrata_SRR4039018'
'Alaskozetes_antarcticus' 'Hermannia_gibba_SRR4039019'
'Archegozetes_longisetosus_MCZ' 'Nothrus_palustris_SRR4039021'
'Platynothrus_peltifer_SRR4039022' 'Pseudotyrannochthonius_B_144011'
'Pseudotyrannochthonius_MCZ' 'Chthoniidae_JC022'
'Lagynochthonius_australicus_MCZ' 'Ephippiochthonius_tetrachelatus_MCZ_140293'
'Lechytia_hoffi_MCZ139248' 'Feaella_capensis_139240'
'Pseudogarypus_banksi_140301' 'Bochica_MCZ144332'
'Dhanus_sumatranus_MCZ49986' 'Indohya_Australia_151267'
'Ideobisium_crassimanum_MCZ' 'Parahya_submersa_139241' 'Gymnobisium_139238'
Microcreaginae 'Microbisium_brunneum_MCZ' 'Microbisium_60599'
'Novobisium_141855' 'Afrogarypus_MCZ139239' 'Geogarypus_maculatus_MCZ'
'Apolpium_MCZ144360' 'Garypus_californicus_MCZ' Synsphyronus
'Pseudogarypinus_frontalis_MCZ139246' 'Larca_granulata_MCZ'
'Protogarypinus_giganteus' 'Cheiridiidae_MCZ140294' 'Sternophoridae_133496'
'Cacodemonius_MCZ144363' 'Oratemnus_curtus_49983' 'Protochelifer_sp_MCZ'
'Cheliferidae_JC031_MCZ40982' 'Parachelifer_persimilis_MCZ139247'
'Lamprochernes_savignyi_MCZ' 'Conicochernes_crassus_MCZ49985'
'Hesperochernes_sp' 'Haplochernes_kraepeli_reassaembly' 'Chernetidae_MCZ142323'
'Chernetidae_sp_JC032' 'Ehan_MCZ74291' 'Espe_MCZ60890' 'Espelaea_UWisc'
'Galendromus_occidentalis' 'Gromphadorholaelaps_schaeferi_MCZ71202'
'Varroa_destructor_SRR3927486' 'Pneumolaelaps_niutirani_SRR10269756'
'Tropilaelaps_mercedesae' 'Adenacarus_sp' 'Neoacarus_sp_MCZ'
```

'Orthinodoros_rostratus_SRR1732011' 'IscaW_1_6'
'Haemaphysalis_longicornis_SRR7754709' 'Amblyomma_americanum'
'Dermacentor_silvarum' 'Hyalomma_excavatum_SRR3157672'
'Rhipicephalus_micropilus_SRR1186998' 'Siro_boyerae_SRR1145699'
'Leptopsalis_141287' 'Miopsalis_SRR5234539_peptides_iso'
'Metagovea_oviformis_MCZ136533' 'Metasiro_savannahensis_IZ-133799'
'Brasilogovea_microphaga_MCZ136559' 'Neogovea_matawai_MCZ133922'
'Parapurcellia_silvicola_MCZ134743' 'Purcellia_illustrans_MCZ49518'
'Chileogovea_oedipus_MCZ138106' 'Rakaia_minutissima_MCZ143240'
'Rakaia_stewartiensis_MCZ49801' 'Rakaia_magna_australis_MCZ29212'
'Rakaia_pauli_MCZ144621' 'Rakaia_pauli_Trini_CDhit' 'Pettalus_thwaitsei_MCZ132349'
'Austropurcellia_alata_MCZ141418' 'Austropurcellia_despectata_MCZ141417'
'Austropurcellia_cf_acuta_MCZ141419' 'Austropurcellia_acuta_MCZ44060'
'Austropurcellia_clousei_MCZ141420' 'Neopurcellia_salmoni_MCZ144625'
'Neopurcellia_salmoni_MCZ29310' 'Karripurcellia_peckorum_MCZ49989'
'Aoraki_141173' 'Aoraki_denticulata_Northern_MCZ141173'
'Aoraki_denticulata_Southern_MCZ141172' 'Aoraki_longitarsa_MCZ137238'
'Acropsopilio_neozealandiae_MCZ30457' 'Hesperonemastoma_modestum'
'Ortholasma_coronadense' 'Trogulus_martensi' 'Caddo_agilis' 'thrasychirus_49762'
'Forsteropsalis_pureora_MCZ29216' 'Pantopsalis_nz2016' 'mangatangi_133381'
'megalopsalis_133420' 'Diguetinus_143123' 'Phalangium_opilio_IZ-29189'
'Protolophus_singularis' 'prionostemma_144108' 'Leiobunum_verrucosum'
'paranelima_143124' 'Sclerobunus_robustus' 'Synthetonychia_glacialis_MCZ137212'
'Larifuga_capensis_IZ-49747' 'Nuncia_nz1211' Pristobunus
'Scotolemon_lespesi_MCZ43672' 'Sitalcina_lobata' 'Gnomulus_141286'
'Dibunus_141285' 'Epedanus_141288' 'Haasus_judaeus' 'Haasus_naasane' Dampetrus
'Metabiantes_sp_IZ-49748' 'Stygnomma_144060' pellobunus 'Kimulidae_143960'
'Tegipiolus_pachypus_MZSP71018' 'pellobunus_144055' 'Pachylicus_acutus_IZ-49749'
'maracaynatum_144047' Avima Karos chapolobunus 'Stygnoplus_143966' Protimesius
'Auranus_hoeferscovitorum_MCZ136531' 'Pickeliana_pickeli_MCZ139249'
'Gonycranaus_pluto_MCZ139251' 'Camarana_sp_MCZ139271' Pseudopachylus
'Bissula_spnov_MCZ139255' 'Pseudopachylus_sp_MCZ139280' 'Vonones_ornata_IZ-49750' 'Incasarcus_argenteus_MCZ139250' 'Quindina_limbata_MZSP71772'
Saramacia 'Ampycinae_sp_MCZ46455' 'Glysterus_sp_MCZ140069'
'Panalus_robustus_MZSP71019' 'Phalangodus_cottus_MZSP71020'
'Santinezia_144078' 'phareicranaus_136554' 'Pseudopucrolia_discrepans_MCZ139277'
'Sadocus_polyacanthus_MCZ138113' 'Eubalta_planiceps_MCZ49760'
'Metagyndes_innata' 'Hernandaria_una_MCZ139258'
'Graphinotus_therezopolis_MCZ139273' 'Meteusarcoides_139264'
'Meteusarcoides_sp_MZSP71024' 'Acutisoma_longipes_MCZ139261'
'Goniosoma_sp_MCZ139266' 'Roeweria_virescens_MCZ139279'
'Longiperna_sp_MCZ139260' iz139272 'Mitobates_sp_MCZ139263'
'Promitobates_ornatus_MZSP71022' 'Gonyleptellus_sp_MCZ139269'
'Gonyleptes_fragilis_MZSP71023' 'Pseudotrogulus_mirim_MCZ139270'

'Pseudotrogulus_sp_MCZ139278' 'Ampheres_leucopheus_MCZ139267' 'Acanthogonyleptes_aff_fulvigranulatus_MZSP71027' 'Gonyleptes_atrus_MZSP71025' 'Gonyleptes_sp_MCZ139262' 'Geraecormobius_bispinifrons_MCZ139274' 'Mischonyx_cuspidatus_MCZ139265' 'Sodreana_leprevosti_MCZ139256' 'Sodreana_sodrena_MCZ139268' 'Progonyleptoidellus_striatus_MZSP71021' 'Iporangaia_pustulosa_MCZ139275' 'Gonyleptoides_marumbiensis_MCZ139254' 'Neosadocus_sp_MCZ139257' 'Galeodes_sp' 'Solpugema_sp_IZ-49525' 'Eremobates_sp_IZ-49755' 'Eremobates_sp_new' Lpol Crot 'Tachypleus_tridentatus_2019G' Tgig 'Ricinoides_atewa' 'Ricinoides_karschii_IZ-130083' 'Pseudocellus_pearsei' 'Pseudocellus_sp_IZ-140060' 'Cryptocellus_sp_IZ-143922' 'Cryptocellus_becki_IZ-136532' 'Cryptocellus_sp_IZ-30913' ChaerilusSS TroglokhammouanusSS Vietbocap Lvar 'Mesobuthus_martensii' Cley BiisraFull CompsoI 'Csch_trinity' Aamox 'Androctonus_australisSS' HottentottaSS 'Oscr_trinity' AcraII 'Bisra_trinity' Bare Lheb LquiE Abal Bgig Grosphus ParabuthusSS Uoli Uvit Tsmi Tarc Tcos Tserrulatus Tlac Pdeb Jaga Jroc Hjun 'Rgar_trinity' Ccar Chentzi 'Centruroides_vittatus' CscuSS Iurus Bcor 'Bothriurus_burmeisteriSS' Centro Cerc Cque Csul Ckeyserlingii SuperSS AnuroctonusSS Brotheas ScorpiopsSS EuscorpiusSS Pdilutus 'Mega_gertschi' MegacormusSS Umordax BelisariusSS Haztecus HadrSS Hconcolorous Hspadix Uhuachuca Kboggerti Papacheanus Vcashi Pbae Smes Svachoni Vmexicanus Sgertschi Kacapulco Kchamela Mvariegatus Pschwenkmeyeri Pspinigerus Kpurepecha Ccoahuilae Moccidentalis Tintrepidus Uyaschenkoi 'Uro_elongatus' Urodacus 'Hormiops_davidovi' 'Hormurus_weberi' Liocheles 'Liocheles_sp_KhaoDeng' 'Nebo_hierochonticus' DiplocentrusSS 'Diplocentrus_zacatecanus' Kolotl 'Kolotl_poncei' 'Iomachus_politus' HadogenesSS 'Hadogenes_paucidens' 'Opistacanthus_asper' 'Opistacanthus_validus' 'Chiromachus_ochropus' OpisthacanthusSS 'Palaeocheloctonus_pauliani' 'Opistophthalmus_glabrifrons' PandinusSS 'Opistophthalmus_walhbergi' 'Opistophthalmus_boehmi' Sfuscus 'Cioa_Trinity' 'Cisr_Trinity' 'Dvar_IZ29740' 'Acor_IZ71252' 'Pmar_Trinity' 'Mastigoproctus_giganteus_new' 'Mlac_IZ71253' 'Draculoides_vinei_IZ-46896' 'Stenochrus_portoricensis_MCZ' 'Stenochrus_sp_IZ-74289' Liphistius 'Liphistius_sp2' 'Sphodros_rufipes' 'Megahexura_fulva' 'Aliatypus_coylei' 'Antrodiaetus_unicolor' 'Porrhothele_sp' 'Microhexura_montivaga' 'Macrothele_calpeiana' 'Paratropis_sp' 'Cyclocosmia_truncata' 'Hebestatis_theveneti' 'Idiops_bersebaensis' 'Aptostichus_stephencolberti' 'Promyrmekiaphila_clathrata' 'Brachythele_longitarsis' 'Pionothele_sp' 'Stanwellia_sp' 'Damarchus_sp' 'Trichopelma_laselva' 'Acanthoscurria_geniculata' 'A_hentzi_pooled' 'Aphonopelma_iviei' 'Filistata_insidiatrix' Kukulcaniahibernalis2 'Hypochilus_gertschi' 'Hypochilus_pococki' 'Calponia_harrisonfordi' 'Segestria_sp' 'Maoriata_sp' 'Orsolobidae_sp' Dysdera 'Ischnothyreus_sp' 'Opopaea_sp' 'Diguetia_sp' 'Pholcus_manueli' 'Pholcus_phalangioides' 'Loxosceles_deserta' 'Drymusa_sp' 'Periegops_suterii' 'Ochyrocera_sp' 'Scytodes_globula' 'Scytodes_thoracica' Archoleptoneta Calileptoneta Austrochilus 'Hickmania_clean' 'Progradungula_GOOD' 'Tarlina_sp' 'Gradungula_sorenseni' 'Pianoa_isolata' 'Austrarchaea_sp' 'Mecysmauchenius_clean' 'Huttonia_palpimanoides' 'Othiotops_birabeni'

'Palpimanus_gibbulus' 'Stegodyphus_sp' 'Megadictyna_thilenii' Nicodamidae2 'Nicodamidae_sp1' 'Mysmenella_sp' 'Novanapis_sp' 'Anelosimus_clean' Latrodectus 'Euryopis_sp' 'Parasteatoda_tepidariorum' 'Pararchaea_alba' 'Malkara_clean' 'Malkaridae_GH09' 'Perissopmeros_sp' 'Australomimetus_clean' 'Ero_leonina' 'Arkys_sp' Demadiana Leucage 'Meta_ovalis_good' 'Nanometa_sp' 'Tetragnatha_tantalus' 'Cepheia_clean' 'Mysmena_leichardti' 'Microdipoena_guttata' 'Spinanapis_sp' 'Tekelloides_sp2' 'Laminacauda_clean' Frontinella 'Novafroneta_sp' 'Synotaxus_sp' 'Nesticus_bishopi' 'Nesticus_cooperi' 'Meringa_sp' 'Runga_sp' 'Physoglenes_clean' 'Physoglenidae_clean' 'Ogulnius_sp' 'Baalzebub_sp' 'Theridiosoma_gemmosum' 'Theridiosoma_savannum' 'Trichonephila_clavipes_Genome' 'Micrathena_gracilis' 'Verrucosa_arenata' 'Cyrtophora_sp' Neoscona 'Gasteracantha_hasselti' 'Macracantha_arcuata' 'Menneus_sp' 'Deinopis_clean' 'Deinopis_longipes' 'Tamopsis_sp' 'Oecobius_navus' 'Oecobius_sp' 'Zosis_sp' 'Philoponella_herediae' 'Uloborus_glomosus' 'Cybaeodamus_taim' 'Forsterella_sp' 'Caayguara_clean' Amaurobius 'Callobius_sp' 'Neoramia_sp' Amphinecta 'Metaltella_simoni' Cambridgea 'Paramatachia_sp' 'Agelenopsis_emertoni_cdhitest.fasta.transdecoder' 'Agelenopsis_pennsylvanica' 'Calymmaria_clean' 'Calymmaria_persica' 'Cicurina_travisae' 'Cicurina_vobora' 'Homalonychus_theologus' 'Tengella_sp' 'Uliodon_sp' 'Misumenoides_formosipes' 'Sidymella_sp' 'Anahita_punctulata' 'Peucetia_longipalpis' 'Dolomedes_trinity' 'Pisaurina_mira' 'Cupiennius_sp' Allocosa 'Schizocosa_rovneri' 'Habronattus_signatus' 'Habronattus_ustulatus' 'Teminius_sp' 'Karaops_raveni' 'Falconina_gracilis' 'Nyssus_sp' 'Clubiona_clean' 'Rebilus_sp' 'Liocranidae_GOOD' 'Trachelas_tranquillus' 'Sergiolus_capulatus' 'Molycria_sp' Anzacia 'Lampona_sp' Goniotarbus Bornatarbus
;

END;

BEGIN CHARACTERS;
DIMENSIONS NCHAR=259;
FORMAT DATATYPE = STANDARD RESPECTCASE GAP = - MISSING = ? SYMBOLS = "  0 1 2 3 4 5 6 7 8 9 A";

MATRIX
Haliestes 00000000-0-000-1000-??????0?0??00-0-00000---200-1??0?0?0-0-00000000000-0??00-----?110000002?0???0000100000090------------1--0---?--00--??????0000??00201110-----??????????????????-------?-?????????????----??----00-????????????????????????????10?101?1?---??????
Palaeopantopus 0000?000-0-000-1200-???????????00-1-0000??--?????????????????????????????????????????????2??????????????????????????????---00---

???0?????????00?????0201110???????????????????????????????????????????
??????????-000???????????????????????????????????????????????

Palaeoisopus 0000?000-0-000-1200-???????????00-1-0?000---200-1????0?0-0-00000000000-0??00-----?110000002?0???0000100000800-010-----001000000??00--0?????0000??00201110----0????????????????????????????????????????????????-0000??????????????????????????0000?021?0?---??????

Flagellopantopus 0??0???0-0-0??-1200-???????????00-1-1?0-0---------???0------?000000000-0??00-----?1100?0002?0???0?00??0??00800-010-----001000000???0?--??????00?????0201110----0????????????????????????????????????????????????-0000???????????????????????????????010?0?---??????

Dibasterium 1000?000-00000-010?????????????00-1-000001---00-0????0?0-0-000?2000000-0?000-----0000?00000?1????1-00?100102??0000000--000001000??00--2-????00???000000000?--0????????????????????????????????????????????????-0??0?????????????????????????----?????0?---??????

Offacolus 1000?000-00110-0?0????????????00-1-000001---00-0????0?0-0-000?2000000-0?000-----00?0?000?0?1????1-000100105??0000000--000001000??00--2-????00???000000000?--0????????????????????????????????????????????????-0??0?????????????????????????----?????0?---??????

Weinbergina 1101?000-1000000?0????????????00-1-000101--200??????0?0-0-000?0000000-0?000-----0000?0000??1???01-0000001031?0000000--000001000???010??????00?????00000001?--0????????????????????????????????????????????????-0??0?????????????????????????----?????0?---??????

Attercopus_fimbriunguis
1????0????????????????????????????1?10?2?-??110??1?1?0??-0-0??????????????????????????????????????????????????????????????????1??????????????0??????0??????????????????????????????????????????????????????????????????????????????????????????????????----?????0?---??????

Permarachne_novokshonovi
1????0?????0???0???????????????10?1?10??????????????????????????????????????????????0??00?2?????00?0??0???0?2?0???0??0-?010??????????????????00?????0????????????????????????????????????????????????????????????????????????????????????????????????????????????----?????0???-??????

Chimerarachne_yingi
100??00100000000?1?0???????????100101002?-??11?11???00?0-??000000-0000-0??00----0001000000211000000010000?0?2000-00000-001001111?0-012????1??00?????000???????????????????????????????????????????????????????????10???????????????????????????----?????0?22-??????

Idmonarachne
100000010000?000???0??????????010000---20---11011???0000-0-0000000000-

0?000----00010000002?1???00101000010?200?0?0100-
?0100???0?????????????00?????0????????????????????????????????????????
???????????????????-0?0???????????????????????????----?????0?---??????

Parastylonurus
1000?00101000000?00????????????1011-000101--100-00???00-0-00000000000-
0?000-----0000?000020000001-0000--1000100000000-00011?000???010?1?00--
0000??00001000??--?????????????????????????????????????????????11?0-
0000??????????????????????????----?????0?---??????

Mixopterus 1000?00101000000?00????????????1011-
000101--100-00???00-0-00001000000-0?000-----0000?000020000001-0000--
1200100000100-00011?000???010?1?00--0000??00001000??--
?????????????????????????????????????????????11?0-
0000??????????????????????????----?????0?---??????

Eurypterus 1000?00101000000?00????????????1011-
000101--100-00???00-0-00000000000-0?000-----0000?000020000001-0000--
1200100000000-000111000???010?1?00--0000??00001000??--
1??????????1?????????????????????????????????11?0-
00001?????????????????????????----?????0?---??????

Chasmataspis
1001?00???010000???????????????1021-000???--????????????-
???????????????????-----?00?????01??????1-0001001000100000100-
01???0??0???010?1?????00????????????????0?????????????????????????????
???????????????????-0??0??????????????????????????----?????0?---??????

Octoberaspis
1100?00???010000???????????????1021-000???--????????????-
???????????????????-----?0?????????????????000001200100000000-
0001000?0???010?1?????00????????????????0?????????????????????????????
???????????????????-0??0??????????????????????????----?????0?---??????

Waeringoscorpio
1000?0010?000000???????????????1011-010???--????????????-???0003001000-
0?100----?0010???002?1???00?0100001000000000000-
001001??0???0???1??????0?????1????????????????????????????????????????
???????????????????-0000??????????????????????????----?????0?--???????

Pulmonoscorpius
1000?00100000000??????1111?????1011-010100--200310???0?0-0-00003001000-
0?100----?0010?00002?1???0000100001000000000000-0010010?0???011?1??0--
10?????0001101??--0?????????????????????????????????????????????1?0-
0000??????????????????????????----?????0?--???????

Palaeoscorpius
1000?0010??00000???????????????101?-?1?100--2??31????0?0-???0003001000-
??100----?0010?00002?1???0000100001000000000000-
001001??0???0?????????00?????000??01??????????????????????????????????
??????????????????0-0000??????????????????????????----?????0?--???????

Compsoscorpius
1000?001001000000?????1111?????1011-010100--20031????0?0-0-00003001000-
??100----?0010???002?1???00?0??0??1000000000000-
0010010?0???011?1??10?00?????000?1?1?????????????????????????????????
??????????????????0-0000??????????????????????????----?????0?--???????
Proscorpius 1000?0010?000000???????????????1011-
010100--20031????0?0-0-00003001000-??100----
?0010?00002?1???0000100001000000000000-
0010010?0???010?1?????00??????????????--
0?????????????????????????????????????????????0-
0000???????????????????????????----?????0?--???????
Palaeocharinus 1000?00101001000011000100??0101000-
0----20---110000000000-0-00002000000-00000----
000100000211100000010000101001-10100-101001?00???011?1?10-
?00?????000001100200??????????????-??????????????????????????????????-
0000???????????????????????????----?????0?---??????
Anthracomartus 1000?00100001000????0010???????00-
0----20---11000????000-0-00002000000-0?000----
00010?00002?1???0000100001010001-10100-
10100???0???01??1?????00?????0???????????????????????????-
????????????????????????????????-0000???????????????????????????----
?????00---??????
Eophrynus 1000?0010?0010-0???????????????00-0--
--20---11000????000-0-0000?000000-0?0?0----
?0010???002?1???0000??0??1010001-10100-00100???0???00--
1?????00?????0???????????????????????????-
????????????????????????????????-00?0???????????????????????????----
?????00---??????
Plesiosiro 1100?0011?001000??0?????????????00-0---
-20---11000????000-0-00000000000-0???0----
?0011??0002?1???0001?00??1010100100000-
00100?1?0???01??1?????00?????0?????1??????????????????????????????????
???????????????????-0000???????????????????????????----?????00---??????
Graeophonus
1000?00110000100????????????????10000---2?-0011001????000-
???00010000010??0010---00011???002?1???00011?0??1012000000000-
00100?1?0??-
01??1?????0??????0??????????????????????????????????????????????????????
?????????00????????????????????????????----?????00---??????
Paracharonopsis
1000?00110000100????????????????10000---20-??110010???000-
1?00001000001111?001100010011??2002?10??000111000101200000000 0-
001001100??-

012?1??1?00000???0?????????????????????????????????????????????????????
?????????00???????????????????????????----?????00---??????
Kronocharon
1000?00110000000?1?????????????10000---20-??110010???000-
1?00001001011 01?101111100011???002?10??00011100010120000000 00-
001001100??-
01??1??1?00000???0?????????????????????????????????????????????????????
?????????00???????????????????????????----?????00---??????
Eophalangium 1000??0??1100000???????????????01-
0----101--200-00?000?0-0-0000?????????????----
??010????020??????????????040000000000-0010-000000-
0???1?????0??????0211111--
??0????????????????????????????????????????????111101??1???????????????
????????????----????110---??????
Hastocularis 1000110??00000000???1?0010????01-0--
--101--200-0????0?0-0-0000?000000-????0----
0?010?00102?1???00001?0???040000000000-0010-00?000-
01??1?????0??????0?????????????????????????????????????????????????????
???111101?0???????????????????????????----???11?---??????
Pycno 00000000-0-000-1200-000000000??---0-000--
-----------0-------0000000000-00000-----011000000200???000010000-090-----------1--0--
-0--00--0300---000-000201110-----000--0-00??00-????-------?-???????????00----00----
00-0-000010--?00000?000000000001101010-10---0-00??
'Tanystylum_orbiculare_P02' 00000000-0-000-1000-000000000??-
--0-00010---2-0-10000000-0-00000000000-00000-----011000000200???000010000-
090-----------1--0---0--00--0300---000-000201110-----000--0100??00-????-------?-
???????????00----00----00-0-000010--?00000?0000000010010130-10---0-00??
Anoplo 00000000-0-000-1000-000000000??---0-
00010---200-10000000-0-00000000000-00000-----011000000200???000010000-090---
---------1--0---0--00--0300---000-000201110-----000--0100??00-????-------?-
???????????00----00----00-0-000010--?00000?0000000021000100-10---0-00??
'Phoxichilidium_tubilariae_P07' 00000000-0-000-1000-000000000??-
--0-00010---200-10000000-0-00000000000-00000-----011000000200???000010000-
090-----------1--0---0--00--0300---000-000201110-----000--0100??00-????-------?-
???????????00----00----00-0-000010--?00000?0000000021000100-10---0-00??
'Nymphon_molleri_P06' 00000000-0-000-1000-000000000??---
0-00010---200-10000000-0-00000000000-00000-----011000000200???000010000-090-
-----------1--0---0--00--0300---000-000201110-----000--0100??00-????-------?-
???????????00----00----00-0-000010--?00000?0000000010110100-10---0-00??
'Meridionale_flava_P04' 00000000-0-000-1000-000000000??---
0-00010---200-10000000-0-00000000000-00000-----011000000200???000010000-090-
-----------1--0---0--00--0300---000-000201110-----000--0100??00-????-------?-
???????????00----00----00-0-000010--?00000?0000000010101011-10---0-00??

'Stylopallene_cheilorynchus_P05' 00000000-0-000-1000-000000000??---0-00010---200-10000000-0-00000000000-00000-----011000000200???000010000-090------------1--0---0--00--0300---000-000201110-----000--0100??00-????-------?-??????????00----00----00-0-000010--?00000?0000000010101011-10---0-00??
'Labidostommatidae_IZ-67347' 00000000-0-00000001-1000000000-01-0----20---000--0000000-0-01100000000-00100----00010000020001001-010000?07000000000100010-000001101401?1101000000000211111--300001000000103100--0???????0??000000000010-1??1110-00011?1001-101-0--01-00110?001---------00---0-00??
'Dinothrombium_tinctorium' 00000000-0-000-0001-1000000000-01-0----20---020--000000010-01104000000-00100----00010000020001001-010000?08000000000100010-000000-01402?1101000000000211111--300001000000103100--0???????0??000000000010-1??1110-00001?1001-101-0--01-001100101---------00---0-00??
'Panonychus_citri_SRR341928' 00000000-0-000-0001-1000000000-01-0----20---020--000000110-01104000000-00100----00010000020101001-010000?08000000000100010-000000-01302?10--0000000211111--300001000000103100--0???????0??000000000010-1??1110-20000?1001-101-0--01-001100001---------00---0-00??
'Tetranychus_cinnabarinus_SRR519097' 00000000-0-000-0001-1000000000-01-0----20---020--000000110-01104000000-00100----00010000020101001-010000?08000000000100010-000000-01302?10--0000000211111--300001000000103100--0???????0??000000000010-1??1110-20000?1001-101-0--01-001100001---------00---0-00??
'Tetranychus_urticae' 00000000-0-000-0001-1000000000-01-0----20---020--000000110-01104000000-00100----00010000020101001-010000?08000000000100010-000000-01302?10--0000000211111--300001000000103100--0???????0??000000000010-1??1110-20000?1001-101-0--01-001100001---------00---0-00??
'Rhizoglyphus_robini_SRR946953' 00000000-0-000-0001-1000000000-01-0----20---000--0000000-0-01100000000-00100----00010000020101001-010000?06000000000100010-000001100--2-10--00000000201110--000001001000103000--0???????0??000000000010-1??1110-20000?1001-101-0--01-001101011---------00---0-00??
'Sarcoptes_scabei' 00000000-0-000-0001-1000000000-01-0----20---000--0000000-0-01100000000-00100----00010000020101001-010000?06000000000100010-000001100--2-10--00000000201110--000001001000103000--0???????0??000000000010-1??1110-20000?1001-101-0--01-001101011---------00---0-00??
'Dermatophagoides_farinae_SRR1016494' 00000000-0-000-0001-1000000000-01-0----20---000--0000000-0-01100000000-00100----00010000020101001-010000?06000000000100010-000001100--2-10--

00000000201110--000001001000103000--0???????0??000000000010-1??1110-20000?1001-101-0--01-001101011---------00---0-00??
'Dermatophagoides_pteronyssinus' 00000000-0-000-0001-1000000000-01-0----20---000--0000000-0-01100000000-00100----00010000020101001-010000?0600000000100010-000001100--2-10--00000000201110--000001001000103000--0???????0??000000000010-1??1110-20000?1001-101-0--01-001101011---------00---0-00??
'Euroglyphus_maynei' 00000000-0-000-0001-1000000000-01-0----20---000--0000000-0-01100000000-00100----00010000020101001-010000?0600000000100010-000001100--2-10--00000000201110--000001001000103000--0???????0??000000000010-1??1110-20000?1001-101-0--01-001101011---------00---0-00??
'Hypochithonius_rufulus_SRR4039020' 00000000-0-00000001-1000000000-01-0----20---000--0000000-0-01110000000-00100----00010000020001001-010000?0600000000100010-000001001301?1101000000000201110--000001000000103000--0???????0??000000000010-1??1110-10011?1001-101-0--01-001101001---------00---0-00??
'Steganacarus_magnus_SRR4039023' 00000000-0-00000001-1000000000-01-0----20---000--0000000-0-01110000000-00100----00010000020001001-010000?0600000000100010-000001001301?1101000000000201110--000001000000103000--0???????0??000000000010-1??1110-10011?1001-101-0--01-001101001---------00---0-00??
'Achipteria_coleoptrata_SRR4039018' 00000000-0-00000001-1000000000-01-0----20---000--0000000-0-01110000000-00100----00010000020001001-010000?0600000000100010-000001001301?1101000000000201110--000001000000103000--0???????0??000000000010-1??1110-10011?1001-101-0--01-001101001---------00---0-00??
'Alaskozetes_antarcticus' 00000000-0-00000001-1000000000-01-0----20---000--0000000-0-01110000000-00100----00010000020001001-010000?0600000000100010-000001001301?1101000000000201110--000001000000103000--0???????0??000000000010-1??1110-10011?1001-101-0--01-001101001---------00---0-00??
'Hermannia_gibba_SRR4039019' 00000000-0-00000001-1000000000-01-0----20---000--0000000-0-01110000000-00100----00010000020001001-010000?0600000000100010-000001001301?1101000000000201110--000001000000103000--0???????0??000000000010-1??1110-10011?1001-101-0--01-001101001---------00---0-00??
'Archegozetes_longisetosus_MCZ' 00000000-0-00000001-1000000000-01-0----20---000--0000000-0-01110000000-00100----00010000020001001-010000?0600000000100010-

000001001301?1101000000000201110--000001000000103000--
0???????0??000000000010-1??1110-10011?1001-101-0--01-001101001---------00---0-
00??
'Nothrus_palustris_SRR4039021' 00000000-0-00000001-
1000000000-01-0----20---000--0000000-0-01110000000-00100----
00010000000020001001-010000?06000000000100010-
000001001301?1101000000000201110--000001000000103000--
0???????0??000000000010-1??1110-10011?1001-101-0--01-001101001---------00---0-
00??
'Platynothrus_peltifer_SRR4039022' 00000000-0-00000001-
1000000000-01-0----20---000--0000000-0-01110000000-00100----
00010000000020001001-010000?06000000000100010-
000001001301?1101000000000201110--000001000000103000--
0???????0??000000000010-1??1110-10011?1001-101-0--01-001101001---------00---0-
00??
'Pseudotyrannochthonius_B_144011' 10000000-00000-0001-
1100000000-00-0----20---000010001100-0-00003001000-00100----
00010000020112002?011000?010000000000-0010-000000-01302-
11000001000211111--0200010000000010000--0??011000000000011121110100110-
00001000100111210000101100000l---------00---0-0001
'Pseudotyrannochthonius_MCZ' 10000000-00000-0001-
1100000000-00-0----20---000010001100-0-00003001000-00100----
00010000020112002?011000?010000000000-0010-000000-01302-
11000001000211111--0200010000000010000--0??011000000000011121110100110-
000010001001112100001011000001---------00---0-0001
'Chthoniidae_JC022' 10000000-00000-0001-1100000000-00-
0----20---000010001100-0-00003001000-00100----
00010000020112002?011000?010000000000-0010-000000-01302-
11000001000211111--0200010000000010000--0??011000000000011121110100110-
000010001001112100001011000001---------00---0-0001
'Lagynochthonius_australicus_MCZ' 10000000-00000-0001-
1100000000-00-0----20---000010001100-0-00003001000-00100----
00010000020112002?011000?010000000000-0010-000000-01302-
11000001000211111--0200010000000010000--0??011000000000011121110100110-
000010001001112100001011000001---------00---0-0001
'Ephippiochthonius_tetrachelatus_MCZ_140293' 10000000-00000-0001-
1100000000-00-0----20---000010001100-0-00003001000-00100----
00010000020112002?011000?010000000000-0010-000000-01302-
11000001000211111--0200010000000010000--0??011000000000011121110100110-
000010001001112100001011000001---------00---0-0001
'Lechytia_hoffi_MCZ139248' 10000000-00000-0001-1100000000-
00-0----20---000010001100-0-00003001000-00100----
00010000020112002?011000?010000000000-0010-000000-01302-

110000001000211111--0200010000000010000--0??0110000000000111211101001110-
000010001001112100001011000001---------00---0-0001
'Feaella_capensis_139240' 10000000-00010-0001-1100000000-00-0----20---000010001100-0-00003001000-00100----00010000020112001-011000?010000000000-0010-000000-01302-110000001000211111--0200010000000010000--0??0110000000000111211101001110-00001?001001112100001011000001---------00---0-0001
'Pseudogarypus_banksi_140301' 10000000-00010-0001-1100000000-00-0----20---000010001100-0-00003001000-00100----00010000020112001-011000?010000000000-0010-000000-01302-110000001000211111--0200010000000010000--0??0110000000000111211101001110-000010001001112100001011000001---------00---0-0001
'Bochica_MCZ144332' 10000000-00000-0001-1100000000-00-0----20---000010001110-0-00003001000-01100----00010000020112000011000?010000000000-0010-000000-01302-110000001000211111--0200010000000010000--0??0110000000000111211101001110-00001?001001112100001011000001---------00---0-0001
'Dhanus_sumatranus_MCZ49986' 10000000-00000-0001-1100000000-00-0----20---000010001110-0-00003001000-01100----00010000020112000011000?010000000000-0010-000000-01302-110000001000211111--0200010000000010000--0??0110000000000111211101001110-00001?001001112100001011000001---------00---0-0001
'Indohya_Australia_151267' 10000000-00000-0001-1100000000-00-0----20---000010001110-0-00003001000-01100----00010000020112000011000?010000000000-0010-000000-01302-110000001000211111--0200010000000010000--0??0110000000000111211101001110-00001?001001112100001011000001---------00---0-0001
'Ideobisium_crassimanum_MCZ' 10000000-00000-0001-1100000000-00-0----20---000010001110-0-00003001000-01100----00010000020112000011000?010000000000-0010-000000-01302-110000001000211111--0200010000000010000--0??0110000000000111211101001110-00001?001001112100001011000001---------00---0-0001
'Parahya_submersa_139241' 10000000-00000-0001-1100000000-00-0----20---000010001110-0-00003001000-01100----00010000020112000011000?010000000000-0010-000000-01302-110000001000211111--0200010000000010000--0??0110000000000111211101001110-00001?001001112100001011000001---------00---0-0001
'Gymnobisium_139238' 10000000-00000-0001-1100000000-00-0----20---000010001110-0-00003001000-01100----00010000020112000011000?010000000000-0010-000000-01302-110000001000211111--0200010000000010000--0??0110000000000111211101001110-00001?001001112100001011000001---------00---0-0001
Microcreaginae 10000000-00000-0001-1100000000-00-0----20---000010001110-0-00003001000-01100----

0001000000201120000011000?010000000000-0010-000000-01302-
11000000100021111--020001000000010000--0??0110000000000111211101001110-
0000100010011121000010110000001---------00---0-0001
'Microbisium_brunneum_MCZ' 10000000-00000-0001-
1100000000-00-0----20---000010001110-0-00003001000-01100----
0001000000201120000011000?010000000000-0010-000000-01302-
11000000100021111--020001000000010000--0??0110000000000111211101001110-
0000100010011121000010110000001---------00---0-0001
'Microbisium_60599' 10000000-00000-0001-1100000000-00-
0----20---000010001110-0-00003001000-01100----
0001000000201120000011000?010000000000-0010-000000-01302-
11000000100021111--020001000000010000--0??0110000000000111211101001110-
0000100010011121000010110000001---------00---0-0001
'Novobisium_141855' 10000000-00000-0001-1100000000-00-
0----20---000010001110-0-00003001000-01100----
0001000000201120000011000?010000000000-0010-000000-01302-
11000000100021111--020001000000010000--0??0110000000000111211101001110-
0000100010011121000010110000001---------00---0-0001
'Afrogarypus_MCZ139239' 10000000-00000-0001-1100000000-
00-0----20---000010001110-0-00003001000-01100----
0001000000201120000011000?010000000000-0010-000000-01302-
11000000100021111--020001000000010000--0??0110000000000111211101001110-
00001?0010011121000010110000001---------00---0-0001
'Geogarypus_maculatus_MCZ' 10000000-00000-0001-
1100000000-00-0----20---000010001110-0-00003001000-01100----
0001000000201120000011000?010000000000-0010-000000-01302-
11000000100021111--020001000000010000--0??0110000000000111211101001110-
00001?0010011121000010110000001---------00---0-0001
'Apolpium_MCZ144360' 10000000-00000-0001-1100000000-
00-0----20---000010001110-0-00003001000-01100----
0001000000201120000011000?010000000000-0010-000000-01302-
11000000100021111--020001000000010000--0??0110000000000111211101001110-
00001?0010011121000010110000001---------00---0-0001
'Garypus_californicus_MCZ' 10000000-00000-0001-1100000000-
00-0----20---000010001110-0-00003001000-01100----
0001000000201120000011000?010000000000-0010-000000-01302-
11000000100021111--020001000000010000--0??0110000000000111211101001110-
0000100010011121000010110000001---------00---0-0001
Synsphyronus 10000000-00000-0001-1100000000-00-0--
--20---000010001110-0-00003001000-01100----
0001000000201120000011000?010000000000-0010-000000-01302-
11000000100021111--020001000000010000--0??0110000000000111211101001110-
0000100010011121000010110000001---------00---0-0001

'Pseudogarypinus_frontalis_MCZ139246' 10000000-00000-0001-1100000000-00-0----20---000010001110-0-00003001000-01100----0001000000201120000011000?010000000000-0010-000000-01302-11000001000211111--020001000000010000--0??0110000000000111211101001110-0000100010011121000010110000001---------00---0-0001
'Larca_granulata_MCZ' 10000000-00000-0001-1100000000-00-0----20---000010001110-0-00003001000-01100----0001000000201120000011000?010000000000-0010-000000-01302-11000001000211111--020001000000010000--0??0110000000000111211101001110-00001?00100111210000101100000001---------00---0-0001
'Protogarypinus_giganteus' 10000000-00000-0001-1100000000-00-0----20---000010001110-0-00003001000-01100----0001000000201120000011000?010000000000-0010-000000-01302-11000001000211111--020001000000010000--0??0110000000000111211101001110-00001?00100111210000101100000001---------00---0-0001
'Cheiridiidae_MCZ140294' 10000000-00000-0001-1100000000-00-0----20---000010001110-0-00003001000-01100----0001000000201120000011000?010000000000-0010-000000-01302-11000001000211111--020001000000010000--0??0110000000000111211101001110-0000100010011121000010110000001---------00---0-0001
'Sternophoridae_133496' 10000000-00000-0001-1100000000-00-0----20---000010001110-0-00003001000-01100----0001000000201120000011000?010000000000-0010-000000-01302-11000001000211111--020001000000010000--0??0110000000000111211101001110-00001?00100111210000101100000001---------00---0-0001
'Cacodemonius_MCZ144363' 10000000-00000-0001-1100000000-00-0----20---000010001110-0-00003001000-01100----0001000000201120000011000?010000000000-0010-000000-01302-11000001000211111--020001000000010000--0??0110000000000111211101001110-00001?00100111210000101100000001---------00---0-0001
'Oratemnus_curtus_49983' 10000000-00000-0001-1100000000-00-0----20---000010001110-0-00003001000-01100----0001000000201120000011000?010000000000-0010-000000-01302-11000001000211111--020001000000010000--0??0110000000000111211101001110-000011001001112100001011000001---------00---0-0001
'Protochelifer_sp_MCZ' 10000000-00000-0001-1100000000-00-0----20---000010001110-0-00003001000-01100----0001000000201120000011000?010000000000-0010-000000-01302-11000001000211111--020001000000010000--0??0110000000000111211101001110-000011001001112100001011000001---------00---0-0001
'Cheliferidae_JC031_MCZ40982' 10000000-00000-0001-1100000000-00-0----20---000010001110-0-00003001000-01100----0001000000201120000011000?010000000000-0010-000000-01302-

1100000001000211111--020001000000010000--0??011000000000011211101001110-
000011001001112100001011000001---------00---0-0001
'Parachelifer_persimilis_MCZ139247' 10000000-00000-0001-
1100000000-00-0----20---000010001110-0-00003001000-01100----
0001000000201120000011000?010000000000-0010-000000-01302-
1100000001000211111--020001000000010000--0??011000000000011211101001110-
000011001001112100001011000001---------00---0-0001
'Lamprochernes_savignyi_MCZ' 10000000-00000-0001-
1100000000-00-0----20---000010001110-0-00003001000-01100----
0001000000201120000011000?010000000000-0010-000000-00--2-
1100000001000211111--020001000000010000--0??011000000000011211101001110-
000011001001112100001011000001---------00---0-00--
'Conicochernes_crassus_MCZ49985' 10000000-00000-0001-
1100000000-00-0----20---000010001110-0-00003001000-01100----
0001000000201120000011000?010000000000-0010-000000-00--2-
1100000001000211111--020001000000010000--0??011000000000011211101001110-
000011001001112100001011000001---------00---0-00--
'Hesperochernes_sp' 10000000-00000-0001-1100000000-00-
0----20---000010001110-0-00003001000-01100----
0001000000201120000011000?010000000000-0010-000000-00--2-
1100000001000211111--020001000000010000--0??011000000000011211101001110-
000011001001112100001011000001---------00---0-00--
'Haplochernes_kraepeli_reassaembly' 10000000-00000-0001-
1100000000-00-0----20---000010001110-0-00003001000-01100----
0001000000201120000011000?010000000000-0010-000000-00--2-
1100000001000211111--020001000000010000--0??011000000000011211101001110-
000011001001112100001011000001---------00---0-00--
'Chernetidae_MCZ142323' 10000000-00000-0001-1100000000-
00-0----20---000010001110-0-00003001000-01100----
0001000000201120000011000?010000000000-0010-000000-00--2-
1100000001000211111--020001000000010000--0??011000000000011211101001110-
000011001001112100001011000001---------00---0-00--
'Chernetidae_sp_JC032' 10000000-00000-0001-1100000000-
00-0----20---000010001110-0-00003001000-01100----
0001000000201120000011000?010000000000-0010-000000-00--2-
1100000001000211111--020001000000010000--0??011000000000011211101001110-
000011001001112100001011000001---------00---0-00--
'Ehan_MCZ74291' 0000000110-000-0000-
0000000001010010100111--200-10000000-0-00000000000-00000----
00011000002010000000010000?022000000000-00100?00000-00--2-01--0000101020---
-----00010000110010000001?0110000000000000000010-1001110-0000??000100---0--
00-0??0??001---------00---0-00--
'Espe_MCZ60890' 0000000110-000-0000-
0000000001010010100111--200-10000000-0-00000000000-00000----

00011000002010000000010000?022000000000-00100?00000-00--2-01--0000101020---
-----00010000110010000001?011000000000000000010-1001110-0000??000100---0--
00-0??0??001---------00---0-00--
'Espelaea_UWisc' 0000000110-000-0000-
0000000001010010100111--200-10000000-0-00000000000-00000----
00011000002010000000010000?022000000000-00100?00000-00--2-01--0000101020---
-----00010000110010000001?011000000000000000010-1001110-0000??000100---0--
00-0??0??001---------00---0-00--
'Galendromus_occidentalis' 10000001000000-0001-1000000000-
01-0----100--000--0000000-0-01100000000-00000----
00010000002010100??011000?0800000000000100010-000000-01402-10--
00100000211111--2000010010001000000-0??011000??000000000010-1001111-
00000-00010101-0--00-101100001---------00---0-00??
'Gromphadorholaelaps_schaeferi_MCZ71202' 10000001000000-0001-
1000000000-01-0----100--000--0000000-0-01100000000-00000----
00010000002010100??011000?0800000000000100010-000000-01402-10--
00100000211111--2000010010001000000-0??011000??000000000010-1001111-
00000-00010101-0--00-101100001---------00---0-00??
'Varroa_destructor_SRR3927486' 10000001000000-0001-
1000000000-01-0----100--000--0000000-0-01100000000-00000----
00010000002010100??011000?0800000000000100010-000000-01402-10--
00100000211111--2000010010001000000-0??011000??000000000010-1001111-
00000-00010101-0--00-101100001---------00---0-00??
'Pneumolaelaps_niutirani_SRR10269756' 10000001000000-0001-
1000000000-01-0----100--000--0000000-0-01100000000-00000----
00010000002010100??011000?0800000000000100010-000000-01402-10--
00100000211111--2000010010001000000-0??011000??000000000010-1001111-
00000-00010101-0--00-101100001---------00---0-00??
'Tropilaelaps_mercedesae' 10000001000000-0001-1000000000-
01-0----100--000--0000000-0-01100000000-00000----
00010000002010100??011000?0800000000000100010-000000-01402-10--
00100000211111--2000010010001000000-0??011000??000000000010-1001111-
00000-00010101-0--00-101100001---------00---0-00??
'Adenacarus_sp' 10000001000000-001101000000000-01-
0----100--000-00000000-0-01110000000-00000----
00011000002000100??011000?010000000000-0010-000000-01302-10--
00100000211111--0300010010001020000-0??011000??00000?00001?-1001110-
0001??00010101-0--00-101100001---------00---0-00??
'Neoacarus_sp_MCZ' 10000001000000-001101000000000-
01-0----100--000-00000000-0-01110000000-00000----
00011000002000100??011000?010000000000-0010-000000-01302-10--
00100000211111--0300010010001020000-0??011000??00000?00001?-1001110-
0001??00010101-0--00-101100001---------00---0-00??

'Orthinodoros_rostratus_SRR1732011' 10000001000000-0001-1000000000-01-0----100--000--0000000-0-01100000000-00000----00010000002010100??011000?0800000000100010-000000-01402-10--00100000211111--2000010010001000000-0??011000??000000000010-1001111-00000-00010101-0--00-101100001---------00---0-00??
'IscaW_1_6' 10000001000000-0001-1000000000-01-0----100--000--0000000-0-01100000000-00000----00010000002010100??011000?0800000000100010-000000-01402-10--00100000211111--2000010010001000000-0??011000??000000000010-1001111-00000-00010101-0--00-101100001---------00---0-00??
'Haemaphysalis_longicornis_SRR7754709' 10000001000000-0001-1000000000-01-0----100--000--0000000-0-01100000000-00000----00010000002010100??011000?0800000000100010-000000-01402-10--00100000211111--2000010010001000000-0??011000??000000000010-1001111-00000-00010101-0--00-101100001---------00---0-00??
'Amblyomma_americanum' 10000001000000-0001-1000000000-01-0----100--000--0000000-0-01100000000-00000----00010000002010100??011000?0800000000100010-000000-01402-10--00100000211111--2000010010001000000-0??011000??000000000010-1001111-00000-00010101-0--00-101100001---------00---0-00??
'Dermacentor_silvarum' 10000001000000-0001-1000000000-01-0----100--000--0000000-0-01100000000-00000----00010000002010100??011000?0800000000100010-000000-01402-10--00100000211111--2000010010001000000-0??011000??000000000010-1001111-00000-00010101-0--00-101100001---------00---0-00??
'Hyalomma_excavatum_SRR3157672' 10000001000000-0001-1000000000-01-0----100--000--0000000-0-01100000000-00000----00010000002010100??011000?0800000000100010-000000-01402-10--00100000211111--2000010010001000000-0??011000??000000000010-1001111-00000-00010101-0--00-101100001---------00---0-00??
'Rhipicephalus_micropilus_SRR1186998' 10000001000000-0001-1000000000-01-0----100--000--0000000-0-01100000000-00000----00010000002010100??011000?0800000000100010-000000-01402-10--00100000211111--2000010010001000000-0??011000??000000000010-1001111-00000-00010101-0--00-101100001---------00---0-00??
'Siro_boyerae_SRR1145699' 10001101000000-0000-1111000010-01-0----101--200-10000000-0-00000000000-00000----0001000000201120000010000?040000000000-0010-000000-00-?2-10--00001000211111--0100010000000100001-0??11100000000002-0001101001111110010-00011101000000-001000001--------100---0-00??
'Leptopsalis_141287' 10001101000000000-1111000010-01-0----101--200-10000000-0-00000000000-00000----0001000000201120000010000?040000000000-0010-000000-014?2-10--

00001000211111--0100010000000100001-0??11100000000002-
00011010011111110010-00011101000000-001000001--------100---0-00??
'Miopsalis_SRR5234539_peptides_iso' 1000110100000000000-
1111000010-01-0----101--200-10000000-0-00000000000-00000----
00010000002011200000100000?040000000000-0010-000000-014?2-10--
00001000211111--0100010000000100001-0??11100000000002-
00011010011111110010-00011101000000-001000001--------100---0-00??
'Metagovea_oviformis_MCZ136533' 10001101000000-0000-
1111000010-01-0----101--200-10000000-0-00000000000-00000----
00010000002011200000100000?040000000000-0010-000000-00-?2-10--
00001000211111--0100010000000100001-0??11100000000002-
00011010011111110010-00011101000000-001000001--------100---0-00??
'Metasiro_savannahensis_IZ-133799' 1000110100000000000-
1111000010-01-0----101--200-10000000-0-00000000000-00000----
00010000002011200000100000?040000000000-0010-000000-004?2-10--
00001000211111--0100010000000100001-0??11100000000002-
00011010011111110010-00011101000000-001000001--------100---0-00??
'Brasilogovea_microphaga_MCZ136559' 1000110100000000000-
1111000010-01-0----101--200-10000000-0-00000000000-00000----
00010000002011200000100000?040000000000-0010-000000-004?2-10--
00001000211111--0100010000000100001-0??11100000000002-
00011010011111110010-00011101000000-001000001--------100---0-00??
'Neogovea_matawai_MCZ133922' 10001101000000-0000-
1111000010-01-0----101--200-10000000-0-00000000000-00000----
00010000002011200000100000?040000000000-0010-000000-00-?2-10--
00001000211111--0100010000000100001-0??11100000000002-
00011010011111110010-00011101000000-001000001--------100---0-00??
'Parapurcellia_silvicola_MCZ134743' 1000110100000000000-
1111000010-01-0----101--200-10000000-0-00000000000-00000----
00010000002011200000100000?040000000000-0010-000000-014?2-10--
00001000211111--0100010000000100001-0??11100000000002-
00011010011111110010-00011101000000-001000001--------100---0-00??
'Purcellia_illustrans_MCZ49518' 1000110100000000000-
1111000010-01-0----101--200-10000000-0-00000000000-00000----
00010000002011200000100000?040000000000-0010-000000-014?2-10--
00001000211111--0100010000000100001-0??11100000000002-
00011010011111110010-00011101000000-001000001--------100---0-00??
'Chileogovea_oedipus_MCZ138106' 10001101000000-0000-
1111000010-01-0----101--200-10000000-0-00000000000-00000----
00010000002011200000100000?040000000000-0010-000000-01-?2-10--
00001000211111--0100010000000100001-0??11100000000002-
00011010011111110010-00011101000000-001000001--------100---0-00??
'Rakaia_minutissima_MCZ143240' 1000110100000000000-
1111000010-01-0----101--200-10000000-0-00000000000-00000----

00010000002011200000100000?040000000000-0010-000000-014?2-10--
00001000211111--0100010000000100001-0??11100000000002-
00011010011111110010-00011101000000-001000001--------100---0-00??
'Rakaia_stewartiensis_MCZ49801' 10001101000000000000-
1111000010-01-0----101--200-10000000-0-00000000000-00000----
00010000002011200000100000?040000000000-0010-000000-014?2-10--
00001000211111--0100010000000100001-0??11100000000002-
00011010011111110010-00011101000000-001000001--------100---0-00??
'Rakaia_magna_australis_MCZ29212' 10001101000000-0000-
1111000010-01-0----101--200-10000000-0-00000000000-00000----
00010000002011200000100000?040000000000-0010-000000-01-?2-10--
00001000211111--0100010000000100001-0??11100000000002-
00011010011111110010-00011101000000-001000001--------100---0-00??
'Rakaia_pauli_MCZ144621' 10001101000000000000-
1111000010-01-0----101--200-10000000-0-00000000000-00000----
00010000002011200000100000?040000000000-0010-000000-014?2-10--
00001000211111--0100010000000100001-0??11100000000002-
00011010011111110010-00011101000000-001000001--------100---0-00??
'Rakaia_pauli_Trini_CDhit' 10001101000000000000-1111000010-
01-0----101--200-10000000-0-00000000000-00000----
00010000002011200000100000?040000000000-0010-000000-014?2-10--
00001000211111--0100010000000100001-0??11100000000002-
00011010011111110010-00011101000000-001000001--------100---0-00??
'Pettalus_thwaitsei_MCZ132349' 10001101000000-0000-
1111000010-01-0----101--200-10000000-0-00000000000-00000----
00010000002011200000100000?040000000000-0010-000000-01-?2-10--
00001000211111--0100010000000100001-0??11100000000002-
00011010011111110010-00011101000000-001000001--------100---0-00??
'Austropurcellia_alata_MCZ141418' 10001101000000000000-
1111000010-01-0----101--200-10000000-0-00000000000-00000----
00010000002011200000100000?040000000000-0010-000000-014?2-10--
00001000211111--0100010000000100001-0??11100000000002-
00011010011111110010-00011101000000-001000001--------100---0-00??
'Austropurcellia_despectata_MCZ141417' 10001101000000000000-
1111000010-01-0----101--200-10000000-0-00000000000-00000----
00010000002011200000100000?040000000000-0010-000000-014?2-10--
00001000211111--0100010000000100001-0??11100000000002-
00011010011111110010-00011101000000-001000001--------100---0-00??
'Austropurcellia_cf_acuta_MCZ141419' 10001101000000-0000-
1111000010-01-0----101--200-10000000-0-00000000000-00000----
00010000002011200000100000?040000000000-0010-000000-01-?2-10--
00001000211111--0100010000000100001-0??11100000000002-
00011010011111110010-00011101000000-001000001--------100---0-00??

'Austropurcellia_acuta_MCZ44060'                100011010000000000-
1111000010-01-0----101--200-10000000-0-00000000000-00000----
000100000020112000010000?040000000000-0010-000000-014?2-10--
00001000211111--01000100000001000001-0??11100000000002-
000110100111111110010-00011101000000-001000001--------100---0-00??
'Austropurcellia_clousei_MCZ141420'             100011010000000000-
1111000010-01-0----101--200-10000000-0-00000000000-00000----
000100000020112000010000?040000000000-0010-000000-014?2-10--
00001000211111--01000100000001000001-0??11100000000002-
000110100111111110010-00011101000000-001000001--------100---0-00??
'Neopurcellia_salmoni_MCZ144625'               10001101000000-0000-
1111000010-01-0----101--200-10000000-0-00000000000-00000----
000100000020112000010000?040000000000-0010-000000-01-?2-10--
00001000211111--01000100000001000001-0??11100000000002-
000110100111111110010-00011101000000-001000001--------100---0-00??
'Neopurcellia_salmoni_MCZ29310'                10001101000000-0000-
1111000010-01-0----101--200-10000000-0-00000000000-00000----
000100000020112000010000?040000000000-0010-000000-01-?2-10--
00001000211111--01000100000001000001-0??11100000000002-
000110100111111110010-00011101000000-001000001--------100---0-00??
'Karripurcellia_peckorum_MCZ49989'              100011010000000000-
1111000010-01-0----101--200-10000000-0-00000000000-00000----
000100000020112000010000?040000000000-0010-000000-014?2-10--
00001000211111--01000100000001000001-0??11100000000002-
000110100111111110010-00011101000000-001000001--------100---0-00??
'Aoraki_141173'                                100011010000000000-1111000010-01-0-
---101--200-10000000-0-00000000000-00000----
000100000020112000010000?040000000000-0010-000000-014?2-10--
00001000211111--01000100000001000001-0??11100000000002-
000110100111111110010-00011101000000-001000001--------100---0-00??
'Aoraki_denticulata_Northern_MCZ141173'        10001101000000-0000-
1111000010-01-0----101--200-10000000-0-00000000000-00000----
000100000020112000010000?040000000000-0010-000000-01-?2-10--
00001000211111--01000100000001000001-0??11100000000002-
000110100111111110010-00011101000000-001000001--------100---0-00??
'Aoraki_denticulata_Southern_MCZ141172'         100011010000000000-
1111000010-01-0----101--200-10000000-0-00000000000-00000----
000100000020112000010000?040000000000-0010-000000-014?2-10--
00001000211111--01000100000001000001-0??11100000000002-
000110100111111110010-00011101000000-001000001--------100---0-00??
'Aoraki_longitarsa_MCZ137238'                  100011010000000000-
1111000010-01-0----101--200-10000000-0-00000000000-00000----
000100000020112000010000?040000000000-0010-000000-014?2-10--

00001000211111--0100010000000100001-0??11100000000002-
0001101001111110010-00011101000000-001000001--------100---0-00??
'Acropsopilio_neozealandiae_MCZ30457' 10001001000000-0000-
1111000011001-0----101--200-10000000-0-00000000000-00100----
0001000010201120000010000?040000000000-0010-000000-00--1010--
00001000211111--0100010000000100001-
0??1110000000000100001101001111010010-00011100-0--00-001000001--------010---
0-0000
'Hesperonemastoma_modestum' 10001001000000-0000-
1111000011001-0----101--201-10000000-0-00000000000-00100----
0001000010201120000010000?040000000000-0010-000000-00--1010--
00001000211111--0100010000000100001-
0??1110000000000100001101001111010010-00011100-0--00-001000001--------010---
0-0000
'Ortholasma_coronadense' 10001001000000-0000-
1111000011001-0----101--201-10000000-0-00000000000-00100----
0001000010201120000010000?040000000000-0010-000000-00--1010--
00001000211111--0100010000000100001-
0??1110000000000100001101001111010010-00011100-0--00-001000001--------010---
0-0000
'Trogulus_martensi' 10001001000000-0000-1111000011001-
0----101--201-10000000-0-00000000000-00100----
0001000010201120000010000?040000000000-0010-000000-00--1010--
00001000211111--0100010000000100001-
0??1110000000000100001101001111010010-00011100-0--00-001000001--------010---
0-0000
'Caddo_agilis' 10001001001000-0000-1111000011001-0---
-101--200-10000000-0-00000000000-00000----
0001000010201120000010000?040000000000-0010-000000-00--1010--
00001000211111--0100010000000100001-
0??1110000000000100001101001111010010-00011100-0--00-001000001--------010---
0-0000
'thrasychirus_49762' 10001001001000-0000-1111000011001-
0----101--200-10000000-0-00000000000-00000----
0001000010201120000010000?040000000000-0010-000000-00--1010--
00001000211111--0100010000000100001-
0??1110000000000100001101001111010010-00011100-0--00-001000001--------010---
0-0000
'Forsteropsalis_pureora_MCZ29216' 10001001001000-0000-
1111000011001-0----101--200-10000000-0-00000000000-00000----
0001000010201120000010000?040000000000-0010-000000-00--1010--
00001000211111--0100010000000100001-
0??1110000000000100001101001111010010-00011100-0--00-001000001--------010---
0-0000

'Pantopsalis_nz2016' 10001001001000-0000-1111000011001-
0----101--200-10000000-0-00000000000-00000----
0001000010201120000010000?040000000000-0010-000000-00--1010--
00001000211111--0100010000000100001-
0??11100000000001000011010011110100010-00011100-0--00-001000001--------010---
0-0000
'mangatangi_133381' 10001001001000-0000-
1111000011001-0----101--200-10000000-0-00000000000-00000----
0001000010201120000010000?040000000000-0010-000000-00--1010--
00001000211111--0100010000000100001-
0??11100000000001000011010011110100010-00011100-0--00-001000001--------010---
0-0000
'megalopsalis_133420' 10001001001000-0000-
1111000011001-0----101--200-10000000-0-00000000000-00000----
0001000010201120000010000?040000000000-0010-000000-00--1010--
00001000211111--0100010000000100001-
0??11100000000001000011010011110100010-00011100-0--00-001000001--------010---
0-0000
'Diguetinus_143123' 10001001001000-0000-1111000011001-
0----101--200-10000000-0-00000000000-00000----
0001000010201120000010000?040000000000-0010-000000-00--1010--
00001000211111--0100010000000100001-
0??11100000000001000011010011110100010-00011100-0--00-001000001--------010---
0-0000
'Phalangium_opilio_IZ-29189' 10001001001000-0000-
1111000011001-0----101--200-10000000-0-00000000000-00000----
0001000010201120000010000?040000000000-0010-000000-00--1010--
00001000211111--0100010000000100001-
0??11100000000001000011010011110100010-00011100-0--00-001000001--------010---
0-0000
'Protolophus_singularis' 10001001001000-0000-1111000011001-
0----101--200-10000000-0-00000000000-00000----
0001000010201120000010000?040000000000-0010-000000-00--1010--
00001000211111--0100010000000100001-
0??11100000000001000011010011110100010-00011100-0--00-001000001--------010---
0-0000
'prionostemma_144108' 10001001001000-0000-
1111000011001-0----101--200-10000000-0-00000000000-00000----
0001000010201120000010000?040000000000-0010-000000-00--1010--
00001000211111--0100010000000100001-
0??11100000000001000011010011110100010-00011100-0--00-001000001--------010---
0-0000
'Leiobunum_verrucosum' 10001001001000-0000-
1111000011001-0----101--200-10000000-0-00000000000-00000----

0001000010201120000010000?040000000000-0010-000000-00--1010--
00001000211111--0100010000000100001-
0??111000000000010000110100111010010-00011100-0--00-001000001--------010---
0-0000
'paranelima_143124' 10001001001000-0000-
1111000011001-0----101--200-10000000-0-00000000000-00000----
0001000010201120000010000?040000000000-0010-000000-00--1010--
00001000211111--0100010000000100001-
0??111000000000010000110100111010010-00011100-0--00-001000001--------010---
0-0000
'Sclerobunus_robustus' 10001001000000-0000-
1111000011001-0----101--200-10000000-0-00001000000-00000----
0001000010201120000010001?040000000000-0010-000000-00--1010--
00001000211111--0100010000000100001-
0??111000000000010000110100111010010-00011100-0--00-001000001--------010---
0-0000
'Synthetonychia_glacialis_MCZ137212' 10001001000000-0000-
1111000011001-0----101--200-10000000-0-00001000000-00000----
0001000010201120000010001?040000000000-0010-000000-00--1010--
00001000211111--0100010000000100001-
0??111000000000010000110100111010010-00011100-0--00-001000001--------000---
0-0000
'Larifuga_capensis_IZ-49747' 10001001000000-0000-
1111000011001-0----101--200-10000000-0-00001000000-00000----
0001000010201120000010001?040000000000-0010-000000-00--1010--
00001000211111--0100010000000100001-
0??111000000000010000110100111010010-00011100-0--00-001000001--------010---
0-0000
'Nuncia_nz1211' 10001001000000-0000-1111000011001-
0----101--200-10000000-0-00001000000-00000----
0001000010201120000010001?040000000000-0010-000000-00--1010--
00001000211111--0100010000000100001-
0??111000000000010000110100111010010-00011100-0--00-001000001--------010---
0-0000
Pristobunus 10001001000000-0000-1111000011001-0---
-101--200-10000000-0-00001000000-00000----
0001000010201120000010001?040000000000-0010-000000-00--1010--
00001000211111--0100010000000100001-
0??111000000000010000110100111010010-00011100-0--00-001000001--------010---
0-0000
'Scotolemon_lespesi_MCZ43672' 10001001000000-0000-
1111000011001-0----101--200-10000000-0-00001000000-00000----
0001000010201120000010001?040000000000-0010-000000-00--1010--
00001000211111--0100010000000100001-

0??1110000000000100001101001111010010-00011100-0--00-001000001--------010---
0-0000
'Sitalcina_lobata' 10001001000000-0000-1111000011001-0--
--101--200-10000000-0-00001000000-00000----
00010000102011200000100001?040000000000-0010-000000-00--1010--
00001000211111--010001000000010000 1-
0??1110000000000100001101001111010010-00011100-0--00-001000001--------010---
0-0000
'Gnomulus_141286' 10001001000000-0000-
1111000011001-0----101--200-10000000-0-00001000000-00000----
00010000102011200000100001?040000000000-0010-000000-00--1010--
00001000211111--0100010000000100001-
0??1110000000000100001101001111010010-00011100-0--00-001000001--------010---
0-0000
'Dibunus_141285' 10001001000000-0000-1111000011001-
0----101--200-10000000-0-00001000000-00000----
00010000102011200000100001?040000000000-0010-000000-00--1010--
00001000211111--0100010000000100001-
0??1110000000000100001101001111010010-00011100-0--00-001000001--------000---
0-0000
'Epedanus_141288' 10001001000000-0000-
1111000011001-0----101--200-10000000-0-00001000000-00000----
00010000102011200000100001?040000000000-0010-000000-00--1010--
00001000211111--0100010000000100001-
0??1110000000000100001101001111010010-00011100-0--00-001000001--------010---
0-0000
'Haasus_judaeus' 10001001000000-0000-1111000011001-
0----101--200-10000000-0-00001000000-00000----
00010000102011200000100001?040000000000-0010-000000-00--1010--
00001000211111--0100010000000100001-
0??1110000000000100001101001111010010-00011100-0--00-001000001--------010---
0-0000
'Haasus_naasane' 10001001000000-0000-1111000011001-
0----101--200-10000000-0-00001000000-00000----
00010000102011200000100001?040000000000-0010-000000-00--2-10--
00001000211111--0100010000000100001-
0??1110000000000100001101001111010010-00011100-0--00-001000001--------010---
0-00--
Dampetrus 10001001000000-0000-1111000011001-0--
--101--200-10000000-0-00001000000-00000----
00010000102011200000100001?040000000000-0010-000000-00--1010--
00001000211111--0100010000000100001-
0??1110000000000100001101001111010010-00011100-0--00-001000001--------010---
0-0000

'Metabiantes_sp_IZ-49748' 10001001000000-0000-1111000011001-0----101--200-10000000-0-00001000000-00000----00010000102011200000100001?040000000000-0010-000000-00--1010--00001000211111--0100010000000100001-0??1110000000000100001101001111010010-00011100-0--00-001000001--------000---0-0000
'Stygnomma_144060' 10001001000000-0000-1111000011001-0----101--200-10000000-0-00001000000-00000----00010000102011200000100001?040000000000-0010-000000-00--1010--00001000211111--0100010000000100001-0??1110000000000100001101001111010010-00011100-0--00-001000001--------000---0-0000
pellobunus 10001001000000-0000-1111000011001-0----101--200-10000000-0-00001000000-00000----00010000102011200000100001?040000000000-0010-000000-00--1010--00001000211111--0100010000000100001-0??1110000000000100001101001111010010-00011100-0--00-001000001--------010---0-0000
'Kimulidae_143960' 10001001000000-0000-1111000011001-0----101--200-10000000-0-00001000000-00000----00010000102011200000100001?040000000000-0010-000000-00--1010--00001000211111--0100010000000100001-0??1110000000000100001101001111010010-00011100-0--00-001000001--------010---0-0000
'Tegipiolus_pachypus_MZSP71018' 10001001000000-0000-1111000011001-0----101--200-10000000-0-00001000000-00000----00010000102011200000100001?040000000000-0010-000000-00--1010--00001000211111--0100010000000100001-0??1110000000000100001101001111010010-00011100-0--00-001000001--------010---0-0000
'pellobunus_144055' 10001001000000-0000-1111000011001-0----101--200-10000000-0-00001000000-00000----00010000102011200000100001?040000000000-0010-000000-00--1010--00001000211111--0100010000000100001-0??1110000000000100001101001111010010-00011100-0--00-001000001--------010---0-0000
'Pachylicus_acutus_IZ-49749' 10001001000000-0000-1111000011001-0----101--200-10000000-0-00001000000-00000----00010000102011200000100001?040000000000-0010-000000-00--1010--00001000211111--0100010000000100001-0??1110000000000100001101001111010010-00011100-0--00-001000001--------010---0-0000
'maracaynatum_144047' 10001001000000-0000-1111000011001-0----101--200-10000000-0-00001000000-00000----

0001000010201120000010001?040000000000-0010-000000-00--1010--
00001000211111--0100010000000100001-
0??111000000000010000110100111010010-00011100-0--00-001000001--------010---
0-0000
Avima 10001001000000-0000-1111000011001-0----
101--200-10000000-0-00001000000-00000----
0001000010201120000010001?040000000000-0010-000000-00--1010--
00001000211111--0100010000000100001-
0??111000000000010000110100111010010-00011100-0--00-001000001--------010---
0-0000
Karos 10001001000000-0000-1111000011001-0----
101--200-10000000-0-00001000000-00000----
0001000010201120000010001?040000000000-0010-000000-00--1010--
00001000211111--0100010000000100001-
0??111000000000010000110100111010010-00011100-0--00-001000001--------010---
0-0000
chapolobunus 10001001000000-0000-1111000011001-0-
---101--200-10000000-0-00001000000-00000----
0001000010201120000010001?040000000000-0010-000000-00--1010--
00001000211111--0100010000000100001-
0??111000000000010000110100111010010-00011100-0--00-001000001--------010---
0-0000
'Stygnoplus_143966' 10001001000000-0000-
1111000011001-0----101--200-10000000-0-00001000000-00000----
0001000010201120000010001?040000000000-0010-000000-00--1010--
00001000211111--0100010000000100001-
0??111000000000010000110100111010010-00011100-0--00-001000001--------000---
0-0000
Protimesius 10001001000000-0000-1111000011001-0---
-101--200-10000000-0-00001000000-00000----
0001000010201120000010001?040000000000-0010-000000-00--1010--
00001000211111--0100010000000100001-
0??111000000000010000110100111010010-00011100-0--00-001000001--------000---
0-0000
'Auranus_hoeferscovitorum_MCZ136531' 10001001000000-0000-
1111000011001-0----101--200-10000000-0-00001000000-00000----
0001000010201120000010001?040000000000-0010-000000-00--1010--
00001000211111--0100010000000100001-
0??111000000000010000110100111010010-00011100-0--00-001000001--------010---
0-0000
'Pickeliana_pickeli_MCZ139249' 10001001000000-0000-
1111000011001-0----101--200-10000000-0-00001000000-00000----
0001000010201120000010001?040000000000-0010-000000-00--1010--
00001000211111--0100010000000100001-

0??11100000000000100001101001111010010-00011100-0--00-001000001--------010---
0-0000
'Gonycranaus_pluto_MCZ139251' 10001001000000-0000-
1111000011001-0----101--200-10000000-0-00001000000-00000----
0001000010201120000010001?040000000000-0010-000000-00--1010--
00001000211111--0100010000000100001-
0??11100000000000100001101001111010010-00011100-0--00-001000001--------010---
0-0000
'Camarana_sp_MCZ139271' 10001001000000-0000-
1111000011001-0----101--200-10000000-0-00001000000-00000----
0001000010201120000010001?040000000000-0010-000000-00--1010--
00001000211111--0100010000000100001-
0??11100000000000100001101001111010010-00011100-0--00-001000001--------010---
0-0000
Pseudopachylus 10001001000000-0000-1111000011001-
0----101--200-10000000-0-00001000000-00000----
0001000010201120000010001?040000000000-0010-000000-00--1010--
00001000211111--0100010000000100001-
0??11100000000000100001101001111010010-00011100-0--00-001000001--------010---
0-0000
'Bissula_spnov_MCZ139255' 10001001000000-0000-
1111000011001-0----101--200-10000000-0-00001000000-00000----
0001000010201120000010001?040000000000-0010-000000-00--1010--
00001000211111--0100010000000100001-
0??11100000000000100001101001111010010-00011100-0--00-001000001--------010---
0-0000
'Pseudopachylus_sp_MCZ139280' 10001001000000-0000-
1111000011001-0----101--200-10000000-0-00001000000-00000----
0001000010201120000010001?040000000000-0010-000000-00--1010--
00001000211111--0100010000000100001-
0??11100000000000100001101001111010010-00011100-0--00-001000001--------010---
0-0000
'Vonones_ornata_IZ-49750' 10001001000000-0000-
1111000011001-0----101--200-10000000-0-00001000000-00000----
0001000010201120000010001?040000000000-0010-000000-00--1010--
00001000211111--0100010000000100001-
0??11100000000000100001101001111010010-00011100-0--00-001000001--------010---
0-0000
'Incasarcus_argenteus_MCZ139250' 10001001000000-0000-
1111000011001-0----101--200-10000000-0-00001000000-00000----
0001000010201120000010001?040000000000-0010-000000-00--1010--
00001000211111--0100010000000100001-
0??11100000000000100001101001111010010-00011100-0--00-001000001--------010---
0-0000

'Quindina_limbata_MZSP71772' 10001001000000-0000-1111000011001-0----101--200-10000000-0-00001000000-00000----00010000102011200000010001?040000000000-0010-000000-00--1010--00001000211111--010001000000100001-0??111000000000010000110100111010010-00011100-0--00-001000001--------010---0-0000
Saramacia 10001001000000-0000-1111000011001-0----101--200-10000000-0-00001000000-00000----00010000102011200000010001?040000000000-0010-000000-00--1010--00001000211111--010001000000100001-0??111000000000010000110100111010010-00011100-0--00-001000001--------010---0-0000
'Ampycinae_sp_MCZ46455' 10001001000000-0000-1111000011001-0----101--200-10000000-0-00001000000-00000----00010000102011200000010001?040000000000-0010-000000-00--1010--00001000211111--010001000000100001-0??111000000000010000110100111010010-00011100-0--00-001000001--------010---0-0000
'Glysterus_sp_MCZ140069' 10001001000000-0000-1111000011001-0----101--200-10000000-0-00001000000-00000----00010000102011200000010001?040000000000-0010-000000-00--1010--00001000211111--010001000000100001-0??111000000000010000110100111010010-00011100-0--00-001000001--------010---0-0000
'Panalus_robustus_MZSP71019' 10001001000000-0000-1111000011001-0----101--200-10000000-0-00001000000-00000----00010000102011200000010001?040000000000-0010-000000-00--1010--00001000211111--010001000000100001-0??111000000000010000110100111010010-00011100-0--00-001000001--------010---0-0000
'Phalangodus_cottus_MZSP71020' 10001001000000-0000-1111000011001-0----101--200-10000000-0-00001000000-00000----00010000102011200000010001?040000000000-0010-000000-00--1010--00001000211111--010001000000100001-0??111000000000010000110100111010010-00011100-0--00-001000001--------010---0-0000
'Santinezia_144078' 10001001000000-0000-1111000011001-0----101--200-10000000-0-00001000000-00000----00010000102011200000010001?040000000000-0010-000000-00--1010--00001000211111--010001000000100001-0??111000000000010000110100111010010-00011100-0--00-001000001--------010---0-0000
'phareicranaus_136554' 10001001000000-0000-1111000011001-0----101--200-10000000-0-00001000000-00000----

0001000010201120000010001?040000000000-0010-000000-00--1010--
00001000211111--0100010000000100001-
0??1110000000000100001101001111010010-00011100-0--00-001000001--------010---
0-0000
'Pseudopucrolia_discrepans_MCZ139277' 10001001000000-0000-
1111000011001-0----101--200-10000000-0-00001000000-00000----
0001000010201120000010001?040000000000-0010-000000-00--1010--
00001000211111--0100010000000100001-
0??1110000000000100001101001111010010-00011100-0--00-001000001--------010---
0-0000
'Sadocus_polyacanthus_MCZ138113' 10001001000000-0000-
1111000011001-0----101--200-10000000-0-00001000000-00000----
0001000010201120000010001?040000000000-0010-000000-00--1010--
00001000211111--0100010000000100001-
0??1110000000000100001101001111010010-00011100-0--00-001000001--------010---
0-0000
'Eubalta_planiceps_MCZ49760' 10001001000000-0000-
1111000011001-0----101--200-10000000-0-00001000000-00000----
0001000010201120000010001?040000000000-0010-000000-00--1010--
00001000211111--0100010000000100001-
0??1110000000000100001101001111010010-00011100-0--00-001000001--------010---
0-0000
'Metagyndes_innata’ 10001001000000-0000-
1111000011001-0----101--200-10000000-0-00001000000-00000----
0001000010201120000010001?040000000000-0010-000000-00--1010--
00001000211111--0100010000000100001-
0??1110000000000100001101001111010010-00011100-0--00-001000001--------010---
0-0000
'Hernandaria_una_MCZ139258' 10001001000000-0000-
1111000011001-0----101--200-10000000-0-00001000000-00000----
0001000010201120000010001?040000000000-0010-000000-00--1010--
00001000211111--0100010000000100001-
0??1110000000000100001101001111010010-00011100-0--00-001000001--------010---
0-0000
'Graphinotus_therezopolis_MCZ139273' 10001001000000-0000-
1111000011001-0----101--200-10000000-0-00001000000-00000----
0001000010201120000010001?040000000000-0010-000000-00--1010--
00001000211111--0100010000000100001-
0??1110000000000100001101001111010010-00011100-0--00-001000001--------010---
0-0000
'Meteusarcoides_139264' 10001001000000-0000-
1111000011001-0----101--200-10000000-0-00001000000-00000----
0001000010201120000010001?040000000000-0010-000000-00--1010--
00001000211111--0100010000000100001-

0??111000000000001000011010011110100 10-00011100-0--00-001000001--------010---
0-0000
'Meteusarcoides_sp_MZSP71024' 10001001000000-0000-
1111000011001-0----101--200-10000000-0-00001000000-00000----
00010000102011200000010001?040000000000-0010-000000-00--1010--
00001000211111--0100010000000100001-
0??111000000000001000011010011110100 10-00011100-0--00-001000001--------010---
0-0000
'Acutisoma_longipes_MCZ139261' 10001001000000-0000-
1111000011001-0----101--200-10000000-0-00001000000-00000----
00010000102011200000010001?040000000000-0010-000000-00--1010--
00001000211111--0100010000000100001-
0??111000000000001000011010011110100 10-00011100-0--00-001000001--------010---
0-0000
'Goniosoma_sp_MCZ139266' 10001001000000-0000-
1111000011001-0----101--200-10000000-0-00001000000-00000----
00010000102011200000010001?040000000000-0010-000000-00--1010--
00001000211111--0100010000000100001-
0??111000000000001000011010011110100 10-00011100-0--00-001000001--------010---
0-0000
'Roeweria_virescens_MCZ139279' 10001001000000-0000-
1111000011001-0----101--200-10000000-0-00001000000-00000----
00010000102011200000010001?040000000000-0010-000000-00--1010--
00001000211111--0100010000000100001-
0??111000000000001000011010011110100 10-00011100-0--00-001000001--------010---
0-0000
'Longiperna_sp_MCZ139260' 10001001000000-0000-
1111000011001-0----101--200-10000000-0-00001000000-00000----
00010000102011200000010001?040000000000-0010-000000-00--1010--
00001000211111--0100010000000100001-
0??111000000000001000011010011110100 10-00011100-0--00-001000001--------010---
0-0000
iz139272 10001001000000-0000-1111000011001-0----
101--200-10000000-0-00001000000-00000----
00010000102011200000010001?040000000000-0010-000000-00--1010--
00001000211111--0100010000000100001-
0??111000000000001000011010011110100 10-00011100-0--00-001000001--------010---
0-0000
'Mitobates_sp_MCZ139263' 10001001000000-0000-
1111000011001-0----101--200-10000000-0-00001000000-00000----
00010000102011200000010001?040000000000-0010-000000-00--1010--
00001000211111--0100010000000100001-
0??111000000000001000011010011110100 10-00011100-0--00-001000001--------010---
0-0000

'Promitobates_ornatus_MZSP71022' 10001001000000-0000-
1111000011001-0----101--200-10000000-0-00001000000-00000----
0001000010201120000010001?040000000000-0010-000000-00--1010--
00001000211111--0100010000000100001-
0??1110000000000100001101001111010010-00011100-0--00-001000001--------010---
0-0000
'Gonyleptellus_sp_MCZ139269' 10001001000000-0000-
1111000011001-0----101--200-10000000-0-00001000000-00000----
0001000010201120000010001?040000000000-0010-000000-00--1010--
00001000211111--0100010000000100001-
0??1110000000000100001101001111010010-00011100-0--00-001000001--------010---
0-0000
'Gonyleptes_fragilis_MZSP71023' 10001001000000-0000-
1111000011001-0----101--200-10000000-0-00001000000-00000----
0001000010201120000010001?040000000000-0010-000000-00--1010--
00001000211111--0100010000000100001-
0??1110000000000100001101001111010010-00011100-0--00-001000001--------010---
0-0000
'Pseudotrogulus_mirim_MCZ139270' 10001001000000-0000-
1111000011001-0----101--200-10000000-0-00001000000-00000----
0001000010201120000010001?040000000000-0010-000000-00--1010--
00001000211111--0100010000000100001-
0??1110000000000100001101001111010010-00011100-0--00-001000001--------010---
0-0000
'Pseudotrogulus_sp_MCZ139278' 10001001000000-0000-
1111000011001-0----101--200-10000000-0-00001000000-00000----
0001000010201120000010001?040000000000-0010-000000-00--1010--
00001000211111--0100010000000100001-
0??1110000000000100001101001111010010-00011100-0--00-001000001--------010---
0-0000
'Ampheres_leucopheus_MCZ139267' 10001001000000-0000-
1111000011001-0----101--200-10000000-0-00001000000-00000----
0001000010201120000010001?040000000000-0010-000000-00--1010--
00001000211111--0100010000000100001-
0??1110000000000100001101001111010010-00011100-0--00-001000001--------010---
0-0000
'Acanthogonyleptes_aff_fulvigranulatus_MZSP71027' 10001001000000-0000-
1111000011001-0----101--200-10000000-0-00001000000-00000----
0001000010201120000010001?040000000000-0010-000000-00--1010--
00001000211111--0100010000000100001-
0??1110000000000100001101001111010010-00011100-0--00-001000001--------010---
0-0000
'Gonyleptes_atrus_MZSP71025' 10001001000000-0000-
1111000011001-0----101--200-10000000-0-00001000000-00000----

0001000010201120000010001?040000000000-0010-000000-00--1010--
00001000211111--0100010000000100001-
0??1110000000000100001101001111010010-00011100-0--00-001000001--------010---
0-0000
'Gonyleptes_sp_MCZ139262' 10001001000000-0000-
1111000011001-0----101--200-10000000-0-00001000000-00000----
0001000010201120000010001?040000000000-0010-000000-00--1010--
00001000211111--0100010000000100001-
0??1110000000000100001101001111010010-00011100-0--00-001000001--------010---
0-0000
'Geraecormobius_bispinifrons_MCZ139274' 10001001000000-0000-
1111000011001-0----101--200-10000000-0-00001000000-00000----
0001000010201120000010001?040000000000-0010-000000-00--1010--
00001000211111--0100010000000100001-
0??1110000000000100001101001111010010-00011100-0--00-001000001--------010---
0-0000
'Mischonyx_cuspidatus_MCZ139265' 10001001000000-0000-
1111000011001-0----101--200-10000000-0-00001000000-00000----
0001000010201120000010001?040000000000-0010-000000-00--1010--
00001000211111--0100010000000100001-
0??1110000000000100001101001111010010-00011100-0--00-001000001--------010---
0-0000
'Sodreana_leprevosti_MCZ139256' 10001001000000-0000-
1111000011001-0----101--200-10000000-0-00001000000-00000----
0001000010201120000010001?040000000000-0010-000000-00--1010--
00001000211111--0100010000000100001-
0??1110000000000100001101001111010010-00011100-0--00-001000001--------010---
0-0000
'Sodreana_sodrena_MCZ139268' 10001001000000-0000-
1111000011001-0----101--200-10000000-0-00001000000-00000----
0001000010201120000010001?040000000000-0010-000000-00--1010--
00001000211111--0100010000000100001-
0??1110000000000100001101001111010010-00011100-0--00-001000001--------010---
0-0000
'Progonyleptoidellus_striatus_MZSP71021' 10001001000000-0000-
1111000011001-0----101--200-10000000-0-00001000000-00000----
0001000010201120000010001?040000000000-0010-000000-00--1010--
00001000211111--0100010000000100001-
0??1110000000000100001101001111010010-00011100-0--00-001000001--------010---
0-0000
'Iporangaia_pustulosa_MCZ139275' 10001001000000-0000-
1111000011001-0----101--200-10000000-0-00001000000-00000----
0001000010201120000010001?040000000000-0010-000000-00--1010--
00001000211111--0100010000000100001-

0??1110000000000100001101001111010010-00011100-0--00-001000001--------010---0-0000
'Gonyleptoides_marumbiensis_MCZ139254' 10001001000000-0000-1111000011001-0----101--200-10000000-0-00001000000-00000----0001000010201120000010001?040000000000-0010-000000-00--1010--00001000211111--0100010000000100001-0??1110000000000100001101001111010010-00011100-0--00-001000001--------010---0-0000
'Neosadocus_sp_MCZ139257' 10001001000000-0000-1111000011001-0----101--200-10000000-0-00001000000-00000----0001000010201120000010001?040000000000-0010-000000-00--1010--00001000211111--0100010000000100001-0??1110000000000100001101001111010010-00011100-0--00-001000001--------010---0-0000
'Galeodes_sp' 00000000-0-00000001-1000000100-00-0----20---000010000010-0-00000000000-00010----0001100000200210000011010?022000000000-0010-100000-01301110--01001000211111--120000--10110010-0--0-------0-?000002-1111101001110-00000-10010101-0--01-001001001---------00---0-0000
'Solpugema_sp_IZ-49525' 00000000-0-00000001-1000000100-00-0----20---000010000010-0-00000000000-00010----0001101000200210000011010?022000000000-0010-100000-01301110--01001000211111--120000--10110010-0--0-------0-?000002-1111101001110-00000-10010101-0--01-001001001---------00---0-0000
'Eremobates_sp_IZ-49755' 00000000-0-00000001-1000000100-00-0----20---000010000010-0-00000000000-00010----0001100000200210000011010?022000000000-0010-100000-01301110--01001000211111--120000--10110010-0--0-------0-?000002-1111101001110-00000-10010101-0--01-001001001---------00---0-0000
'Eremobates_sp_new' 00000000-0-00000001-1000000100-00-0----20---000010000010-0-00000000000-00010----0001100000200210000011010?022000000000-0010-100000-01301110--01001000211111--120000--10110010-0--0-------0-?000002-1111101001110-00000-10010101-0--01-001001001---------00---0-0000
Lpol 11110000-1010000100-1010100100-00-1-000101--200-00000000-0-00002000000-00000-----00000000010100011-0001001131-0000000--02000100000-01011200--0000100000100001--01010000000100000-0010000000?000000000000-0000100-00000-00001111000000?001000000---------00---0-1010
Crot 11110000-1010000100-1010100100-00-1-000101--200-00000000-0-00002000000-00000-----00000000010100011-0001001131-0000000--02000100000-01011200--0000100000100001--01010000000100000-0010000000?000000000000-0000100-00000-00001111000000?001000000---------00---0-1010

'Tachypleus_tridentatus_2019G' 11110000-1010000100-1010100100-00-1-000101--200-00000000-0-00002000000-00000-----0000000010100011-0001001131-0000000--02000100000-01011200--0000100000100001--01010000000100000-0010000000?000000000000-0000100-00000-00001111000000?001000000---------00---0-1010
Tgig 11110000-1010000100-1010100100-00-1-000101--200-00000000-0-00002000000-00000-----0000000010100011-0001001131-0000000--02000100000-01011200--0000100000100001--01010000000100000-0010000000?000000000000-0000100-00000-00001111000000?001000000---------00---0-1010
'Ricinoides_atewa' 10000011000000-001000000000100-10000---20---100200000000-0-01002000000-00000----01010000112000100000010000?010000010100-20100000000-015-2-10--00110000211111--200001001000102000--0??0100?0?0000002-21010-1001110-00100-0000011?200010000?100001---------00---0-00??
'Ricinoides_karschii_IZ-130083' 10000011000000-001000000000100-10000---20---100200000000-0-01002000000-00000----01010000112000100000010000?010000010100-20100000000-015-2-10--00110000211111--200001001000102000--0??0100?0?0000002-21010-1001110-00100-0000011?200010000?100001---------00---0-00??
'Pseudocellus_pearsei' 10000011000000-001000000000100-10000---20---100200000000-0-01002000000-00000----01010000112000100000010000?010000010100-20100000000-015-2-10--00110000211111--200001001000102000--0??0100?0?0000002-21010-1001110-00100-0000011?200010000?100001---------00---0-00??
'Pseudocellus_sp_IZ-140060' 10000011000000-001000000000100-10000---20---100200000000-0-01002000000-00000----01010000112000100000010000?010000010100-20100000000-015-2-10--00110000211111--200001001000102000--0??0100?0?0000002-21010-1001110-00100-0000011?200010000?100001---------00---0-00??
'Cryptocellus_sp_IZ-143922' 10000011000000-001000000000100-10000---20---100200000000-0-01002000000-00000----01010000112000100000010000?010000010100-20100000000-015-2-10--00110000211111--200001001000102000--0??0100?0?0000002-21010-1001110-00100-0000011?200010000?100001---------00---0-00??
'Cryptocellus_becki_IZ-136532' 10000011000000-001000000000100-10000---20---100200000000-0-01002000000-00000----01010000112000100000010000?010000010100-20100000000-015-2-10--00110000211111--200001001000102000--0??0100?0?0000002-21010-1001110-00100-0000011?200010000?100001---------00---0-00??
'Cryptocellus_sp_IZ-30913' 10000011000000-001000000000100-10000---20---100200000000-0-01002000000-00000----01010000112000100000010000?010000010100-20100000000-015-2-10--

00110000211111--200001001000102000--0??0100?0?0000002-21010-1001110-00100-0000011?200010000?100001---------00---0-00??
ChaerilusSS 1000000100100000000-0010110010-1011-010100--200310000000-0-00003001000-00100----?0010000002011200000100001000000000000-00100100000-011110110010??110000110110--00010010000100001-010110000000000001111111101001110-000011000011012100001010000001---------00--2101111
TroglokhammouanusSS 1000000100100000000-0010110010-1011-010100--200310000000-0-00003001000-00100----?0010000002011200000100001000000000000-00100100000-014110110010??110000110110--00010010000100001-010110000000000001111111101001110-000011000011012100001010000001---------00--3101111
Vietbocap 1000000100100000000-0010110010-1011-010100--200310000000-0-00003001000-00100----?0010000002011200000100001000000000000-00100100000-014110110010??110000110110--00010010000100001-010110000000000001111111101001110-000011000011012100001010000001---------00--3101111
Lvar 1000000100100000000-0010110010-1011-010100--200310000000-0-00003001000-00100----?0010000002011200000100001000000000000-00100100000-011110110010??110000110110--00010010000100001-010110000000000001111111101001110-000011000011012100001010000001---------00--0101111
'Mesobuthus_martensii' 1000000100100000000-0010110010-1011-010100--200310000000-0-00003001000-00100----?0010000002011200000100001000000000000-00100100000-011110110010??110000110110--00010010000100001-010110000000000001111111101001110-000011000011012100001010000001---------00--0101111
Cley 1000000100100000000-0010110010-1011-010100--200310000000-0-00003001000-00100----?0010000002011200000100001000000000000-00100100000-011110110010??110000110110--00010010000100001-010110000000000001111111101001110-000011000011012100001010000001---------00--0101111
BiisraFull 1000000100100000000-0010110010-1011-010100--200310000000-0-00003001000-00100----?0010000002011200000100001000000000000-00100100000-011110110010??110000110110--00010010000100001-010110000000000001111111101001110-000011000011012100001010000001---------00--0101111

Compsol 1000000100100000000-0010110010-1011-
010100--200310000000-0-00003001000-00100----
?0010000002011200000100001000000000000-00100100000-
011110110010??110000110110--00010010000100001-
0101100000000000111111101001110-000011000011012100001010000001---------00-
-0101111
'Csch_trinity' 1000000100100000000-0010110010-1011-
010100--200310000000-0-00003001000-00100----
?0010000002011200000100001000000000000-00100100000-
011110110010??110000110110--00010010000100001-
0101100000000000111111101001110-000011000011012100001010000001---------00-
-0101111
Aamox 1000000100100000000-0010110010-1011-
010100--200310000000-0-00003001000-00100----
?0010000002011200000100001000000000000-00100100000-
011110110010??110000110110--00010010000100001-
0101100000000000111111101001110-000011000011012100001010000001---------00-
-0101111
'Androctonus_australisSS' 1000000100100000000-0010110010-
1011-010100--200310000000-0-00003001000-00100----
?0010000002011200000100001000000000000-00100100000-
011110110010??110000110110--00010010000100001-
0101100000000000111111101001110-000011000011012100001010000001---------00-
-0101111
HottentottaSS 1000000100100000000-0010110010-1011-
010100--200310000000-0-00003001000-00100----
?0010000002011200000100001000000000000-00100100000-
011110110010??110000110110--00010010000100001-
0101100000000000111111101001110-000011000011012100001010000001---------00-
-0101111
'Oscr_trinity' 1000000100100000000-0010110010-1011-
010100--200310000000-0-00003001000-00100----
?0010000002011200000100001000000000000-00100100000-
011110110010??110000110110--00010010000100001-
0101100000000000111111101001110-000011000011012100001010000001---------00-
-0101111
AcraII 1000000100100000000-0010110010-1011-
010100--200310000000-0-00003001000-00100----
?0010000002011200000100001000000000000-00100100000-
011110110010??110000110110--00010010000100001-
0101100000000000111111101001110-000011000011012100001010000001---------00-
-0101111
'Bisra_trinity' 1000000100100000000-0010110010-1011-
010100--200310000000-0-00003001000-00100----

?0010000002011200000100001000000000000-00100100000-
011110110010??110000110110--00010010000100001-
0101100000000000111111101001110-00001100001101210000101000001---------00-
-0101111
Bare 100000010010000000-0010110010-1011-
010100--200310000000-0-00003001000-00100----
?0010000002011200000100001000000000000-00100100000-
011110110010??110000110110--00010010000100001-
0101100000000000111111101001110-00001100001101210000101000001---------00-
-0101111
Lheb 100000010010000000-0010110010-1011-
010100--200310000000-0-00003001000-00100----
?0010000002011200000100001000000000000-00100100000-
011110110010??110000110110--00010010000100001-
0101100000000000111111101001110-00001100001101210000101000001---------00-
-0101111
LquiE 100000010010000000-0010110010-1011-
010100--200310000000-0-00003001000-00100----
?0010000002011200000100001000000000000-00100100000-
011110110010??110000110110--00010010000100001-
0101100000000000111111101001110-00001100001101210000101000001---------00-
-0101111
Abal 100000010010000000-0010110010-1011-
010100--200310000000-0-00003001000-00100----
?0010000002011200000100001000000000000-00100100000-
011110110010??110000110110--00010010000100001-
0101100000000000111111101001110-00001100001101210000101000001---------00-
-0101111
Bgig 100000010010000000-0010110010-1011-
010100--200310000000-0-00003001000-00100----
?0010000002011200000100001000000000000-00100100000-
011110110010??110000110110--00010010000100001-
0101100000000000111111101001110-00001100001101210000101000001---------00-
-0101111
Grosphus 100000010010000000-0010110010-1011-
010100--200310000000-0-00003001000-00100----
?0010000002011200000100001000000000000-00100100000-
011110110010??110000110110--00010010000100001-
0101100000000000111111101001110-00001100001101210000101000001---------00-
-0101111
ParabuthusSS 100000010010000000-0010110010-
1011-010100--200310000000-0-00003001000-00100----
?0010000002011200000100001000000000000-00100100000-
011110110010??110000110110--00010010000100001-

0101100000000000111111101001110-0000110000110121000010100000001---------00--0101111

Uoli 1000000100100000000-0010110010-1011-010100--200310000000-0-00003001000-00100----?001000000201120000010000100000000000-00100100000-011110110010??110000110110--00010010000100001-0101100000000000111111101001110-0000110000110121000010100000001---------00--0101111

Uvit 1000000100100000000-0010110010-1011-010100--200310000000-0-00003001000-00100----?001000000201120000010000100000000000-00100100000-011110110010??110000110110--00010010000100001-0101100000000000111111101001110-0000110000110121000010100000001---------00--0101111

Tsmi 1000000100100000000-0010110010-1011-010100--200310000000-0-00003001000-00100----?001000000201120000010000100000000000-00100100000-011110110010??110000110110--00010010000100001-0101100000000000111111101001110-0000110000110121000010100000001---------00--0101111

Tarc 1000000100100000000-0010110010-1011-010100--200310000000-0-00003001000-00100----?001000000201120000010000100000000000-00100100000-011110110010??110000110110--00010010000100001-0101100000000000111111101001110-0000110000110121000010100000001---------00--0101111

Tcos 1000000100100000000-0010110010-1011-010100--200310000000-0-00003001000-00100----?001000000201120000010000100000000000-00100100000-011110110010??110000110110--00010010000100001-0101100000000000111111101001110-0000110000110121000010100000001---------00--0101111

Tserrulatus 1000000100100000000-0010110010-1011-010100--200310000000-0-00003001000-00100----?001000000201120000010000100000000000-00100100000-011110110010??110000110110--00010010000100001-0101100000000000111111101001110-0000110000110121000010100000001---------00--0101111

Tlac 1000000100100000000-0010110010-1011-010100--200310000000-0-00003001000-00100----?001000000201120000010000100000000000-00100100000-011110110010??110000110110--00010010000100001-0101100000000000111111101001110-0000110000110121000010100000001---------00--0101111

Pdeb 1000000100100000000-0010110010-1011-
010100--200310000000-0-00003001000-00100----
?00100000020112000001000010000000000000-00100100000-
011110110010??110000110110--00010010000100001-
0101100000000000011111110100111O-0000110000110121000010100000001---------00-
-0101111
Jaga 1000000100100000000-0010110010-1011-
010100--200310000000-0-00003001000-00100----
?00100000020112000001000010000000000000-00100100000-
011110110010??110000110110--00010010000100001-
0101100000000000011111110100111O-0000110000110121000010100000001---------00-
-0101111
Jroc 1000000100100000000-0010110010-1011-
010100--200310000000-0-00003001000-00100----
?00100000020112000001000010000000000000-00100100000-
011110110010??110000110110--00010010000100001-
0101100000000000011111110100111O-0000110000110121000010100000001---------00-
-0101111
Hjun 1000000100100000000-0010110010-1011-
010100--200310000000-0-00003001000-00100----
?00100000020112000001000010000000000000-00100100000-
011110110010??110000110110--00010010000100001-
0101100000000000011111110100111O-0000110000110121000010100000001---------00-
-0101111
'Rgar_trinity' 1000000100100000000-0010110010-1011-
010100--200310000000-0-00003001000-00100----
?00100000020112000001000010000000000000-00100100000-
011110110010??110000110110--00010010000100001-
0101100000000000011111110100111O-0000110000110121000010100000001---------00-
-0101111
Ccar 1000000100100000000-0010110010-1011-
010100--200310000000-0-00003001000-00100----
?00100000020112000001000010000000000000-00100100000-
011110110010??110000110110--00010010000100001-
0101100000000000011111110100111O-0000110000110121000010100000001---------00-
-0101111
Chentzi 1000000100100000000-0010110010-1011-
010100--200310000000-0-00003001000-00100----
?00100000020112000001000010000000000000-00100100000-
011110110010??110000110110--00010010000100001-
0101100000000000011111110100111O-0000110000110121000010100000001---------00-
-0101111
'Centruroides_vittatus' 1000000100100000000-0010110010-
1011-010100--200310000000-0-00003001000-00100----

?001000000201120000010000100000000000-00100100000-
011110110010??110000110110--00010010000100001-
0101100000000000111111101001110-0000110000110121000010100000001---------00-
-0101111
CscuSS 1000000100100000000-0010110010-1011-
010100--200310000000-0-00003001000-00100----
?001000000201120000010000100000000000-00100100000-
011110110010??110000110110--00010010000100001-
0101100000000000111111101001110-0000110000110121000010100000001---------00-
-0101111
Iurus 1000000100100000000-0010110010-1011-
010100--200310000000-0-00003001000-00100----
?001000000201120000010000100000000000-00100100000-
011110110010??110000110110--00010010000100001-
0101100000000000111111101001110-0000110000110121000010100000001---------00-
-1101111
Bcor 1000000100100000000-0010110010-1011-
010100--200310000000-0-00003001000-00100----
?001000000201120000010000100000000000-00100100000-
011110110010??110000110110--00010010000100001-
0101100000000000111111101001110-0000110000110121000010100000001---------00-
-1101111
'Bothriurus_burmeisteriSS' 1000000100100000000-0010110010-
1011-010100--200310000000-0-00003001000-00100----
?001000000201120000010000100000000000-00100100000-
011110110010??110000110110--00010010000100001-
0101100000000000111111101001110-0000110000110121000010100000001---------00-
-1101111
Centro 1000000100100000000-0010110010-1011-
010100--200310000000-0-00003001000-00100----
?001000000201120000010000100000000000-00100100000-
011110110010??110000110110--00010010000100001-
0101100000000000111111101001110-0000110000110121000010100000001---------00-
-1101111
Cerc 1000000100100000000-0010110010-1011-
010100--200310000000-0-00003001000-00100----
?001000000201120000010000100000000000-00100100000-
011110110010??110000110110--00010010000100001-
0101100000000000111111101001110-0000110000110121000010100000001---------00-
-1101111
Cque 1000000100100000000-0010110010-1011-
010100--200310000000-0-00003001000-00100----
?001000000201120000010000100000000000-00100100000-
011110110010??110000110110--00010010000100001-

01011000000000000111111101001110-000011000011012100001010000001---------00--1101111

Csul 1000000100100000000-0010110010-1011-010100--200310000000-0-00003001000-00100----?0010000002011200000100001000000000000-00100100000-011110110010??110000110110--00010010000100001-01011000000000000111111101001110-000011000011012100001010000001---------00--1101111

Ckeyserlingii 1000000100100000000-0010110010-1011-010100--200310000000-0-00003001000-00100----?0010000002011200000100001000000000000-00100100000-011110110010??110000110110--00010010000100001-01011000000000000111111101001110-000011000011012100001010000001---------00--1101111

SuperSS 1000000100100000000-0010110010-1011-010100--200310000000-0-00003001000-00100----?0010000002011200000100001000000000000-00100100000-011110110010??110000110110--00010010000100001-01011000000000000111111101001110-000011000011012100001010000001---------00--1101111

AnuroctonusSS 1000000100100000000-0010110010-1011-010100--200310000000-0-00003001000-00100----?0010000002011200000100001000000000000-00100100000-011110110010??110000110110--00010010000100001-01011000000000000111111101001110-000011000011012100001010000001---------00--1101111

Brotheas 1000000100100000000-0010110010-1011-010100--200310000000-0-00003001000-00100----?0010000002011200000100001000000000000-00100100000-011110110010??110000110110--00010010000100001-01011000000000000111111101001110-000011000011012100001010000001---------00--1101111

ScorpiopsSS 1000000100100000000-0010110010-1011-010100--200310000000-0-00003001000-00100----?0010000002011200000100001000000000000-00100100000-011110110010??110000110110--00010010000100001-01011000000000000111111101001110-000011000011012100001010000001---------00--1101111

EuscorpiusSS 1000000100100000000-0010110010-1011-010100--200310000000-0-00003001000-00100----?0010000002011200000100001000000000000-00100100000-011110110010??110000110110--00010010000100001-01011000000000000111111101001110-000011000011012100001010000001---------00--1101111

Pdilutus 1000000100100000000-0010110010-1011-010100--200310000000-0-00003001000-00100----?00100000020112000001000010000000000000-00100100000-011110110010??110000110110--00010010000100001-010110000000000001111111010011110-000011000011012100001010000001---------00--1101111
'Mega_gertschi' 1000000100100000000-0010110010-1011-010100--200310000000-0-00003001000-00100----?00100000020112000001000010000000000000-00100100000-011110110010??110000110110--00010010000100001-010110000000000001111111010011110-000011000011012100001010000001---------00--1101111
MegacormusSS 1000000100100000000-0010110010-1011-010100--200310000000-0-00003001000-00100----?00100000020112000001000010000000000000-00100100000-011110110010??110000110110--00010010000100001-010110000000000001111111010011110-000011000011012100001010000001---------00--1101111
Umordax 1000000100100000000-0010110010-1011-010100--200310000000-0-00003001000-00100----?00100000020112000001000010000000000000-00100100000-011110110010??110000110110--00010010000100001-010110000000000001111111010011110-000011000011012100001010000001---------00--1101111
BelisariusSS 1000000100100000000-0010110010-1011-010100--200310000000-0-00003001000-00100----?00100000020112000001000010000000000000-00100100000-011110110010??110000110110--00010010000100001-010110000000000001111111010011110-000011000011012100001010000001---------00--11011--
Haztecus 1000000100100000000-0010110010-1011-010100--200310000000-0-00003001000-00100----?00100000020112000001000010000000000000-00100100000-011110110010??110000110110--00010010000100001-010110000000000001111111010011110-000011000011012100001010000001---------00--1101111
HadrSS 1000000100100000000-0010110010-1011-010100--200310000000-0-00003001000-00100----?00100000020112000001000010000000000000-00100100000-011110110010??110000110110--00010010000100001-010110000000000001111111010011110-000011000011012100001010000001---------00--1101111
Hconcolorous 1000000100100000000-0010110010-1011-010100--200310000000-0-00003001000-00100----

?0010000002011200000100001000000000000-00100100000-
011110110010??110000110110--00010010000100001-
01011000000000001111111010011110-00001100001101210000101000001---------00-
-1101111
Hspadix 10000001001000000000-0010110010-1011-
010100--200310000000-0-00003001000-00100----
?0010000002011200000100001000000000000-00100100000-
011110110010??110000110110--00010010000100001-
01011000000000001111111010011110-00001100001101210000101000001---------00-
-1101111
Uhuachuca 10000001001000000000-0010110010-1011-
010100--200310000000-0-00003001000-00100----
?0010000002011200000100001000000000000-00100100000-
011110110010??110000110110--00010010000100001-
01011000000000001111111010011110-00001100001101210000101000001---------00-
-1101111
Kboggerti 10000001001000000000-0010110010-1011-
010100--200310000000-0-00003001000-00100----
?0010000002011200000100001000000000000-00100100000-
011110110010??110000110110--00010010000100001-
01011000000000001111111010011110-00001100001101210000101000001---------00-
-1101111
Papacheanus 10000001001000000000-0010110010-
1011-010100--200310000000-0-00003001000-00100----
?0010000002011200000100001000000000000-00100100000-
011110110010??110000110110--00010010000100001-
01011000000000001111111010011110-00001100001101210000101000001---------00-
-1101111
Vcashi 10000001001000000000-0010110010-1011-
010100--200310000000-0-00003001000-00100----
?0010000002011200000100001000000000000-00100100000-
011110110010??110000110110--00010010000100001-
01011000000000001111111010011110-00001100001101210000101000001---------00-
-1101111
Pbae 10000001001000000000-0010110010-1011-
010100--200310000000-0-00003001000-00100----
?0010000002011200000100001000000000000-00100100000-
011110110010??110000110110--00010010000100001-
01011000000000001111111010011110-00001100001101210000101000001---------00-
-1101111
Smes 10000001001000000000-0010110010-1011-
010100--200310000000-0-00003001000-00100----
?0010000002011200000100001000000000000-00100100000-
011110110010??110000110110--00010010000100001-

0101100000000000111111101001110-00001100001101210000101000000l---------00--1101111
Svachoni 1000000100100000000-0010110010-1011-010100--200310000000-0-00003001000-00100----?0010000002011200000100001000000000000-00100100000-011110110010??110000110110--00010010000100001-0101100000000000111111101001110-0000110000110121000010100000001---------00--1101111
Vmexicanus 1000000100100000000-0010110010-1011-010100--200310000000-0-00003001000-00100----?0010000002011200000100001000000000000-00100100000-011110110010??110000110110--00010010000100001-0101100000000000111111101001110-0000110000110121000010100000001---------00--1101111
Sgertschi 1000000100100000000-0010110010-1011-010100--200310000000-0-00003001000-00100----?0010000002011200000100001000000000000-00100100000-011110110010??110000110110--00010010000100001-0101100000000000111111101001110-0000110000110121000010100000001---------00--1101111
Kacapulco 1000000100100000000-0010110010-1011-010100--200310000000-0-00003001000-00100----?0010000002011200000100001000000000000-00100100000-011110110010??110000110110--00010010000100001-0101100000000000111111101001110-0000110000110121000010100000001---------00--1101111
Kchamela 1000000100100000000-0010110010-1011-010100--200310000000-0-00003001000-00100----?0010000002011200000100001000000000000-00100100000-011110110010??110000110110--00010010000100001-0101100000000000111111101001110-0000110000110121000010100000001---------00--1101111
Mvariegatus 1000000100100000000-0010110010-1011-010100--200310000000-0-00003001000-00100----?0010000002011200000100001000000000000-00100100000-011110110010??110000110110--00010010000100001-0101100000000000111111101001110-0000110000110121000010100000001---------00--1101111
Pschwenkmeyeri 1000000100100000000-0010110010-1011-010100--200310000000-0-00003001000-00100----?0010000002011200000100001000000000000-00100100000-011110110010??110000110110--00010010000100001-0101100000000000111111101001110-0000110000110121000010100000001---------00--1101111

Pspinigerus 1000000100100000000-0010110010-1011-
010100--200310000000-0-00003001000-00100----
?0010000002011200000100001000000000000-00100100000-
011110110010??110000110110--00010010000100001-
010110000000000011111101001110-000011000011012100001010000001---------00-
-1101111
Kpurepecha 1000000100100000000-0010110010-1011-
010100--200310000000-0-00003001000-00100----
?0010000002011200000100001000000000000-00100100000-
011110110010??110000110110--00010010000100001-
010110000000000011111101001110-000011000011012100001010000001---------00-
-1101111
Ccoahuilae 1000000100100000000-0010110010-1011-
010100--200310000000-0-00003001000-00100----
?0010000002011200000100001000000000000-00100100000-
011110110010??110000110110--00010010000100001-
010110000000000011111101001110-000011000011012100001010000001---------00-
-1101111
Moccidentalis 1000000100100000000-0010110010-1011-
010100--200310000000-0-00003001000-00100----
?0010000002011200000100001000000000000-00100100000-
011110110010??110000110110--00010010000100001-
010110000000000011111101001110-000011000011012100001010000001---------00-
-1101111
Tintrepidus 1000000100100000000-0010110010-1011-
010100--200310000000-0-00003001000-00100----
?0010000002011200000100001000000000000-00100100000-
011110110010??110000110110--00010010000100001-
010110000000000011111101001110-000011000011012100001010000001---------00-
-1101111
Uyaschenkoi 1000000100100000000-0010110010-1011-
010100--200310000000-0-00003001000-00100----
?0010000002011200000100001000000000000-00100100000-
011110110010??110000110110--00010010000100001-
010110000000000011111101001110-000011000011012100001010000001---------00-
-1111111
'Uro_elongatus' 1000000100100000000-0010110010-1011-
010100--200310000000-0-00003001000-00100----
?0010000002011200000100001000000000000-00100100000-
011110110010??110000110110--00010010000100001-
010110000000000011111101001110-000011000011012100001010000001---------00-
-1111111
Urodacus 1000000100100000000-0010110010-1011-
010100--200310000000-0-00003001000-00100----

?001000000201120000010000100000000000-00100100000-
011110110010??110000110110--00010010000100001-
0101100000000000111111101001110-00001100001101210000101000001---------00-
-1111111
'Hormiops_davidovi' 10000001001000000000-0010110010-
1011-010100--200310000000-0-00003001000-00100----
?001000000201120000010000100000000000-00100100000-
011110110010??110000110110--00010010000100001-
0101100000000000111111101001110-00001100001101210000101000001---------00-
-1111111
'Hormurus_weberi' 10000001001000000000-0010110010-
1011-010100--200310000000-0-00003001000-00100----
?001000000201120000010000100000000000-00100100000-
011110110010??110000110110--00010010000100001-
0101100000000000111111101001110-00001100001101210000101000001---------00-
-1111111
Liocheles 10000001001000000000-0010110010-1011-
010100--200310000000-0-00003001000-00100----
?001000000201120000010000100000000000-00100100000-
011110110010??110000110110--00010010000100001-
0101100000000000111111101001110-00001100001101210000101000001---------00-
-1111111
'Liocheles_sp_KhaoDeng' 10000001001000000000-0010110010-
1011-010100--200310000000-0-00003001000-00100----
?001000000201120000010000100000000000-00100100000-
011110110010??110000110110--00010010000100001-
0101100000000000111111101001110-00001100001101210000101000001---------00-
-1111111
'Nebo_hierochonticus' 10000001001000000000-0010110010-
1011-010100--200310000000-0-00003001000-00100----
?001000000201120000010000100000000000-00100100000-
011110110010??110000110110--00010010000100001-
0101100000000000111111101001110-00001100001101210000101000001---------00-
-1111111
DiplocentrusSS 10000001001000000000-0010110010-
1011-010100--200310000000-0-00003001000-00100----
?001000000201120000010000100000000000-00100100000-
011110110010??110000110110--00010010000100001-
0101100000000000111111101001110-00001100001101210000101000001---------00-
-1111111
'Diplocentrus_zacatecanus' 10000001001000000000-0010110010-
1011-010100--200310000000-0-00003001000-00100----
?001000000201120000010000100000000000-00100100000-
011110110010??110000110110--00010010000100001-

0101100000000000111111101001110-0000110000110121000010100000001---------00--1111111
Kolotl 1000000100100000000-0010110010-1011-010100--200310000000-0-00003001000-00100----?0010000002011200000100001000000000000-00100100000-011110110010??110000110110--00010010000100001-0101100000000000111111101001110-0000110000110121000010100000001---------00--1111111
'Kolotl_poncei' 1000000100100000000-0010110010-1011-010100--200310000000-0-00003001000-00100----?0010000002011200000100001000000000000-00100100000-011110110010??110000110110--00010010000100001-0101100000000000111111101001110-0000110000110121000010100000001---------00--1111111
'Iomachus_politus' 1000000100100000000-0010110010-1011-010100--200310000000-0-00003001000-00100----?0010000002011200000100001000000000000-00100100000-011110110010??110000110110--00010010000100001-0101100000000000111111101001110-0000110000110121000010100000001---------00--1111111
HadogenesSS 1000000100100000000-0010110010-1011-010100--200310000000-0-00003001000-00100----?0010000002011200000100001000000000000-00100100000-011110110010??110000110110--00010010000100001-0101100000000000111111101001110-0000110000110121000010100000001---------00--1111111
'Hadogenes_paucidens' 1000000100100000000-0010110010-1011-010100--200310000000-0-00003001000-00100----?0010000002011200000100001000000000000-00100100000-011110110010??110000110110--00010010000100001-0101100000000000111111101001110-0000110000110121000010100000001---------00--1111111
'Opistacanthus_asper' 1000000100100000000-0010110010-1011-010100--200310000000-0-00003001000-00100----?0010000002011200000100001000000000000-00100100000-011110110010??110000110110--00010010000100001-0101100000000000111111101001110-0000110000110121000010100000001---------00--1111111
'Opistacanthus_validus' 1000000100100000000-0010110010-1011-010100--200310000000-0-00003001000-00100----?0010000002011200000100001000000000000-00100100000-011110110010??110000110110--00010010000100001-0101100000000000111111101001110-0000110000110121000010100000001---------00--1111111

'Chiromachus_ochropus' 1000000100100000000-0010110010-1011-010100--200310000000-0-00003001000-00100----?001000000201120000010000100000000000-00100100000-011110110010??110000110110--0001001000010000 1-0101100000000000011111110100111 0-000011000011012100001010000001---------00--1111111
OpisthacanthusSS 1000000100100000000-0010110010-1011-010100--200310000000-0-00003001000-00100----?001000000201120000010000100000000000-00100100000-011110110010??110000110110--00010010000100001-010110000000000001111111010011 10-000011000011012100001010000001---------00--1111111
'Palaeocheloctonus_pauliani' 1000000100100000000-0010110010-1011-010100--200310000000-0-00003001000-00100----?001000000201120000010000100000000000-00100100000-011110110010??110000110110--00010010000100001-0101100000000000011111110100111 0-000011000011012100001010000001---------00--1111111
'Opistophthalmus_glabrifrons' 1000000100100000000-0010110010-1011-010100--200310000000-0-00003001000-00100----?001000000201120000010000100000000000-00100100000-011110110010??110000110110--00010010000100001-0101100000000000011111110100111 0-000011000011012100001010000001---------00--1111111
PandinusSS 1000000100100000000-0010110010-1011-010100--200310000000-0-00003001000-00100----?001000000201120000010000100000000000-00100100000-011110110010??110000110110--00010010000100001-0101100000000000011111110100111 0-000011000011012100001010000001---------00--1111111
'Opistophthalmus_walhbergi' 1000000100100000000-0010110010-1011-010100--200310000000-0-00003001000-00100----?001000000201120000010000100000000000-00100100000-011110110010??110000110110--00010010000100001-0101100000000000011111110100111 0-000011000011012100001010000001---------00--1111111
'Opistophthalmus_boehmi' 1000000100100000000-0010110010-1011-010100--200310000000-0-00003001000-00100----?001000000201120000010000100000000000-00100100000-011110110010??110000110110--00010010000100001-0101100000000000011111110100111 0-000011000011012100001010000001---------00--1111111
Sfuscus 1000000100100000000-0010110010-1011-010100--200310000000-0-00003001000-00100----

?001000000201120000010000100000000000-00100100000-011110110010??110000110110--00010010000100001-0101100000000000111111101001110-0000110000110121000010100000001---------00--1111111
'Cioa_Trinity'
1000000110000000010110000000001110000---20-01110010100000-10000010010110100011000100111?20021100100011100010120000000000-00100110000-01201011000000010000001110--0011101000112011-1110011111??111110000010-1111110-000011011001113110100001011001---------00---0-1111
'Cisr_Trinity' 1000000110000000010110000000001110000---20-01110010100000-10000010010110100011000100111?20021100100011100010120000000000-00100110000-01201011000000010000001110--0011101000112011-1110011111??111110000010-1111110-000011011001113110100001011001---------00---0-1111
'Dvar_IZ29740'
1000000110000000010110000000001110000---20-11110010100000-10000010010110100011000100111?20021100100011100010120000000000-00100110000-01201011000000010000001110--0011101000102011-1110011111??111110000010-1111110-000011011001113110100001011001---------00---0-1111
'Acor_IZ71252'
1000000110000000010110000000001110000---20-11110010100000-10000010010110100011000100111?20021100100011100010120000000000-00100110000-01201011000000010000001110--0011101000102011-1110011111??111110000010-1111110-000011011001113110100001011001---------00---0-1111
'Pmar_Trinity'
1000000110000000010110000000001110000---20-11110010100000-10000010010110100011000100111?20021100100011100010120000000000-00100110000-01201011000000010000001110--0011101000102011-1110011111??111110000010-1111110-000011011001113110100001011001---------00---0-1111
'Mastigoproctus_giganteus_new'
100000011?0000100101100000000011100101002 1---110010100000-0-01001101100-00100----00011110002110010001100001012000100000-00100110010-01201011100000110000001110--0001111000102011-1110011111111111100000111111110-000012011001113100100001001001---------00---0-1111
'Mlac_IZ71253'
100000011?0000100101100000000011100101002 1---110010100000-0-01001101100-00100----00011110002110010001100001012000100000-00100110010-01201011100000110000001110--0001111000102011-

1110011111111111100000111111110-00001201100111310010000100100 1---------00---0-1111
'Draculoides_vinei_IZ-46896' 0000000110-010-001011000000001110010101 21---110010100000-1111001000000-00100----0001111000211001000110000101200 0100000-00100110010-00--2-111000001100000111 10--000111000102011-?1100111111011111 2-2011111111110-0000120110011131011 0000100?001---------00---0-1111
'Stenochrus_portoricensis_MCZ' 0000000110-010-001011000000001110010101 21---110010100000-1111001000000-00100----0001111000211001000110000101200 0100000-00100110010-00--2-111000001100000111 10--000111000102011-?1100111111011111 2-2011111111110-0000120110011131011 0000100?001---------00---0-1111
'Stenochrus_sp_IZ-74289' 0000000110-010-001011000000001110010101 21---110010100000-1111001000000-00100----0001111000211001000110000101200 0100000-00100110010-00--2-111000001100000111 10--000111000102011-?1100111111011111 2-2011111111110-0000120110011131011 0000100?001---------00---0-1111
Liphistius 100000010000000001001000001001110001---20---110111110000-0-00000000000-00000----0001000000211000001010000101200 0000000-00100111100-01201111000011010000001110--0011001000112000-00100110000000000000000110100 1110-01000-000001113111100001010001---------0022-0-1111
'Liphistius_sp2'
100000010000000001001000001001110001---20---110111110000-0-00000000000-00000----0001000000211000001010000101200 0000000-00100111100-01201111000011010000001110--0011001000112000-00100110000000000000000110100 1110-01000-000001113111100001010001---------0022-0-1111
'Sphodros_rufipes'
100000010000000001001000001001?10001---20---110011110000-0-00000000000-00000----0001000000211000001010000101200 00000001000100111100-01201111000011110000001110--0011001000112000-00100110000000000000000110100 1110-01000-000001113111100001010001---------000?-0-1111
'Megahexura_fulva'
100000010000000001001000001001?10001---20---110011110000-0-00000000000-00000----0001000000211000001010000101200 00000001000100111100-01201111000011110000001110--0011001000112000-00100110000000000000000110100 1110-01000-000001113111100001010001---------0002-0-1111
'Aliatypus_coylei'
100000010000000001001000001001?10001---20---110011110000-0-00000000000-00000----0001000000211000001010000101200 00000001000100111100-

01201111000011110000001110--0011001000112000-
0010011000000000000000001101001110-01000-000001113111100001010001---------
0000-0-1111
'Antrodiaetus_unicolor'
1000000100000000001001000001001?10001---20---110011110000-0-00000000000-
00000----00010000002110000010100001012000000001000100111100-
01201111000011110000001110--0011001000112000-
0010011000000000000000001101001110-01000-000001113111100001010001---------
0001-0-1111
'Porrhothele_sp'
1000000100000000001001000001001010001---20---110011110000-0-00000000000-
00000----00010000002110000010100001012000000001000100111100-
01201111000011110000001110--0011001000112000-
0010011000000000000000001101001110-01000-000001113111100001010001---------
0001-0-1111
'Microhexura_montivaga'
1000000100000000001001000001001010001---20---110011110000-0-00000000000-
00000----00010000002110000010100001012000000001000100111100-
01201111000011110000001110--0011001000112000-
0010011000000000000000001101001110-01000-000001113111100001010001---------
0001-0-1111
'Macrothele_calpeiana'
1000000100000000001001000001001010001---20---110011110000-0-00000000000-
00000----00010000002110000010100001012000000001000100111100-
01201111000011110000001110--0011001000112000-
0010011000000000000000001101001110-01000-000001113111100001010001---------
0001-0-1111
'Paratropis_sp'
1000000100000000001001000001001010001---20---110011110000-0-00000000000-
00000----00010000002110000010100001012000000001000100111100-
01201111000011110000001110--0011001000112000-
0010011000000000000000001101001110-01000-000001113111100001010001---------
0001-0-1111
'Cyclocosmia_truncata'
1000000100000000001001000001001010001---20---110011110000-0-00000000000-
00000----00010000002110000010100001012000000001000100111100-
01201111000011110000001110--0011001000112000-
0010011000000000000000001101001110-01000-000001113111100001010001---------
0000-0-1111
'Hebestatis_theveneti'
1000000100000000001001000001001010001---20---110011110000-0-00000000000-
00000----00010000002110000010100001012000000001000100111100-
01201111000011110000001110--0011001000112000-

0010011000000000000000001101001110-01000-000001113111100001010001---------
0000-0-1111
'Idiops_bersebaensis'
1000000100000000001001000001001010001---20---110011110000-0-00000000000-
00000----00010000002110000010100001012000000001000100111100-
012011110000111100000001110--001100100011 2000-
0010011000000000000000001101001110-01000-000001113111100001010001---------
0000-0-1111
'Aptostichus_stephencolberti'
1000000100000000001001000001001010001---20---110011110000-0-00000000000-
00000----00010000002110000010100001012000000001000100111100-
012011110000111100000001110--001100100011 2000-
0010011000000000000000001101001110-01000-000001113111100001010001---------
0000-0-1111
'Promyrmekiaphila_clathrata'
1000000100000000001001000001001010001---20---110011110000-0-00000000000-
00000----00010000002110000010100001012000000001000100111100-
012011110000111100000001110--001100100011 2000-
0010011000000000000000001101001110-01000-000001113111100001010001---------
0000-0-1111
'Brachythele_longitarsis'
1000000100000000001001000001001010001---20---110011110000-0-00000000000-
00000----00010000002110000010100001012000000001000100111100-
012011110000111100000001110--001100100011 2000-
0010011000000000000000001101001110-01000-000001113111100001010001---------
0001-0-1111
'Pionothele_sp'
1000000100000000001001000001001010001---20---110011110000-0-00000000000-
00000----00010000002110000010100001012000000001000100111100-
012011110000111100000001110--001100100011 2000-
0010011000000000000000001101001110-01000-000001113111100001010001---------
0000-0-1111
'Stanwellia_sp'
1000000100000000001001000001001010001---20---110011110000-0-00000000000-
00000----00010000002110000010100001012000000001000100111100-
012011110000111100000001110--001100100011 2000-
0010011000000000000000001101001110-01000-000001113111100001010001---------
0001-0-1111
'Damarchus_sp'
1000000100000000001001000001001010001---20---110011110000-0-00000000000-
00000----00010000002110000010100001012000000001000100111100-
012011110000111100000001110--001100100011 2000-
0010011000000000000000001101001110-01000-000001113111100001010001---------
0001-0-1111

'Trichopelma_laselva'
1000000100000000010010000010010100001---20---110011110000-0-00000000000-
00000----000100000021100000101000010120000000001000100111100-
0120111100001111000000111 0--0011001000112000-
00100110000000000000000110100111 0-01000-000001113111100001010001---------
0001-0-1111
'Acanthoscurria_geniculata'
1000000100000000010010000010010100001---20---110011110000-0-00000000000-
00000----000100000021100000101000010120000000001000100111100-
0120111100001111000000111 0--0011001000112000-
00100110000000000000000110100111 0-01000-000001113111100001010001---------
0001-0-1111
'A_hentzi_pooled'
1000000100000000010010000010010100001---20---110011110000-0-00000000000-
00000----000100000021100000101000010120000000001000100111100-
0120111100001111000000111 0--0011001000112000-
00100110000000000000000110100111 0-01000-000001113111100001010001---------
0001-0-1111
'Aphonopelma_iviei'
1000000100000000010010000010010100001---20---110011110000-0-00000000000-
00000----000100000021100000101000010120000000001000100111100-
0120111100001111000000111 0--0011001000112000-
00100110000000000000000110100111 0-01000-000001113111100001010001---------
0001-0-1111
'Filistata_insidiatrix'
10000001000000000100100000100 1?10001---20---110211110000-0-00000000000-
00000----000100000021100000101000010120000000001000100111100-
0120111100001101000101111 0--0011001000102000-
00100100000000000000000110100111 0-01000-000001113111100001010001---------
0000-0-1111
Kukulcaniahibernalis2
10000001000000000100100000100 1?10001---20---110211110000-0-00000000000-
00000----000100000021100000101000010120000000001000100111100-
0120111100001101000101111 0--0011001000102000-
00100100000000000000000110100111 0-01000-000001113111100001010001---------
0000-0-1111
'Hypochilus_gertschi'
10000001000000000100100000100 1?10001---20---110211110000-0-00000000000-
00000----000100000021100000101000010120000000001000100111100-
0120111100001101000101111 0--0011001000102000-
00100100000000000000000110100111 0-01000-000001113111100001010001---------
0000-0-1111
'Hypochilus_pococki'
10000001000000000100100000100 1?10001---20---110211110000-0-00000000000-

00000----00010000002110000010100001012000000001000100111100-
01201111000011010001011110--0011001000102000-
00100100000000000000001101001110-01000-000001113111100001010001---------
0000-0-1111
'Calponia_harrisonfordi'
100000010000000001001000001001?10001---20---110211110000-0-00000000000-
00000----00010000002110000010100001012000000001000100111100-
01201111000011010001011110--0011001000102000-
00100100000000000000001101001110-01000-000001113111100001010001---------
0000-0-1111
'Segestria_sp'
100000010000000001001000001001?10001---20---110211110000-0-00000000000-
00000----00010000002110000010100001012000000001000100111100-
01201111000011010001011110--0011001000102000-
00100100000000000000001101001110-01000-000001113111100001010001---------
0000-0-1111
'Maoriata_sp'
100000010000000001001000001001?10001---20---110211110000-0-00000000000-
00000----00010000002110000010100001012000000001000100111100-
01201111000011010001011110--0011001000102000-
00100100000000000000001101001110-01000-000001113111100001010001---------
0000-0-1111
'Orsolobidae_sp'
100000010000000001001000001001?10001---20---110211110000-0-00000000000-
00000----00010000002110000010100001012000000001000100111100-
01201111000011010001011110--0011001000102000-
00100100000000000000001101001110-01000-000001113111100001010001---------
0000-0-1111
Dysdera
100000010000000001001000001001?10001---20---110211110000-0-00000000000-
00000----00010000002110000010100001012000000001000100111100-
01201111000011010001011110--0011001000102000-
00100100000000000000001101001110-01000-000001113111100001010001---------
0000-0-1111
'Ischnothyreus_sp'
100000010000000001001000001001?10001---20---110211110000-0-00000000000-
00000----00010000002110000010100001012000000001000100111100-
01201111000011010001011110--0011001000102000-
00100100000000000000001101001110-01000-000001113111100001010001---------
0000-0-1111
'Opopaea_sp'
100000010000000001001000001001?10001---20---110211110000-0-00000000000-
00000----00010000002110000010100001012000000001000100111100-
01201111000011010001011110--0011001000102000-

00100100000000000000001101001110-01000-000001113111100001010001---------
0000-0-1111
'Diguetia_sp'
100000010000000001001000001001?10001---20---110211110000-0-00000000000-
00000----0001000000021100000101000010120000000001000100111100-
01201111000011010001011110--0011001000102000-
00100100000000000000001101001110-01000-000001113111100001010001---------
0000-0-1111
'Pholcus_manueli'
100000010000000001001000001001?10001---20---110211110000-0-00000000000-
00000----0001000000021100000101000010120000000001000100111100-
01201111000011010001011110--0011001000102000-
00100100000000000000001101001110-01000-000001113111100001010001---------
0000-0-1111
'Pholcus_phalangioides'
100000010000000001001000001001?10001---20---110211110000-0-00000000000-
00000----0001000000021100000101000010120000000001000100111100-
01201111000011010001011110--0011001000102000-
00100100000000000000001101001110-01000-000001113111100001010001---------
0000-0-1111
'Loxosceles_deserta'
100000010000000001001000001001?10001---20---110211110000-0-00000000000-
00000----0001000000021100000101000010120000000001000100111100-
01201111000011010001011110--0011001000102000-
00100100000000000000001101001110-01000-000001113111100001010001---------
0000-0-1111
'Drymusa_sp'
100000010000000001001000001001?10001---20---110211110000-0-00000000000-
00000----0001000000021100000101000010120000000001000100111100-
01201111000011010001011110--0011001000102000-
00100100000000000000001101001110-01000-000001113111100001010001---------
0000-0-1111
'Periegops_suterii'
100000010000000001001000001001?10001---20---110211110000-0-00000000000-
00000----0001000000021100000101000010120000000001000100111100-
01201111000011010001011110--0011001000102000-
00100100000000000000001101001110-01000-000001113111100001010001---------
0000-0-1111
'Ochyrocera_sp'
100000010000000001001000001001?10001---20---110211110000-0-00000000000-
00000----0001000000021100000101000010120000000001000100111100-
01201111000011010001011110--0011001000102000-
00100100000000000000001101001110-01000-000001113111100001010001---------
0000-0-1111

'Scytodes_globula'
1000000100000000001001000001001?10001---20---110211110000-0-00000000000-00000----000100000021100000101000010120000000001000100111100-012011110000110100010111110--0011001000102000-0010010000000000000000011010011 10-01000-000001113111100001010001---------0000-0-1111

'Scytodes_thoracica'
1000000100000000001001000001001?10001---20---110211110000-0-00000000000-00000----000100000021100000101000010120000000001000100111100-012011110000110100010111110--0011001000102000-001001000000000000000001101001110-01000-000001113111100001010001---------0000-0-1111

Archoleptoneta
1000000100000000001001000001001?10001---20---110211110000-0-00000000000-00000----000100000021100000101000010120000000001000100111100-012011110000110100010111110--0011001000102000-001001000000000000000001101001110-01000-000001113111100001010001---------0000-0-1111

Calileptoneta
1000000100000000001001000001001?10001---20---110211110000-0-00000000000-00000----000100000021100000101000010120000000001000100111100-012011110000110100010111110--0011001000102000-001001000000000000000001101001110-01000-000001113111100001010001---------0000-0-1111

Austrochilus
1000000100000000001001000001001?10001---20---110211110000-0-00000000000-00000----000100000021100000101000010120000000001000100111100-012011110000110100010111110--0011001000102000-001001000000000000000001101001110-01000-000001113111100001010001---------0000-0-1111

'Hickmania_clean'
1000000100000000001001000001001?10001---20---110211110000-0-00000000000-00000----000100000021100000101000010120000000001000100111100-012011110000110100010111110--0011001000102000-001001000000000000000001101001110-01000-000001113111100001010001---------0000-0-1111

'Progradungula_GOOD'
1000000100000000001001000001001?10001---20---110211110000-0-00000000000-00000----000100000021100000101000010120000000001000100111100-012011110000110100010111110--0011001000102000-001001000000000000000001101001110-01000-000001113111100001010001---------0000-0-1111

'Tarlina_sp' 1000000100000000001001000001001?10001---20---110211110000-0-00000000000-00000----

000100000021100000101000010120000000001000100111100-
01201111000011010001011110--0011001000102000-
0010010000000000000000001101001110-01000-000001113111100001010001---------
0000-0-1111
'Gradungula_sorenseni'
1000000100000000001001000001001?10001---20---110211110000-0-00000000000-
00000----000100000021100000101000010120000000001000100111100-
01201111000011010001011110--0011001000102000-
0010010000000000000000001101001110-01000-000001113111100001010001---------
0000-0-1111
'Pianoa_isolata'
1000000100000000001001000001001?10001---20---110211110000-0-00000000000-
00000----000100000021100000101000010120000000001000100111100-
01201111000011010001011110--0011001000102000-
0010010000000000000000001101001110-01000-000001113111100001010001---------
0000-0-1111
'Austrarchaea_sp'
1000000100000000001001000001001?10001---20---110211110000-0-00000000000-
00000----000100000021100000101000010120000000001000100111100-
01201111000011010001011110--0011001000102000-
0010010000000000000000001101001110-01000-000001113111100001010001---------
0000-0-1111
'Mecysmauchenius_clean'
1000000100000000001001000001001?10001---20---110211110000-0-00000000000-
00000----000100000021100000101000010120000000001000100111100-
01201111000011010001011110--0011001000102000-
0010010000000000000000001101001110-01000-000001113111100001010001---------
0000-0-1111
'Huttonia_palpimanoides'
1000000100000000001001000001001?10001---20---110211110000-0-00000000000-
00000----000100000021100000101000010120000000001000100111100-
01201111000011010001011110--0011001000102000-
0010010000000000000000001101001110-01000-000001113111100001010001---------
0000-0-1111
'Othiotops_birabeni'
1000000100000000001001000001001?10001---20---110211110000-0-00000000000-
00000----000100000021100000101000010120000000001000100111100-
01201111000011010001011110--0011001000102000-
0010010000000000000000001101001110-01000-000001113111100001010001---------
0000-0-1111
'Palpimanus_gibbulus'
1000000100000000001001000001001?10001---20---110211110000-0-00000000000-
00000----000100000021100000101000010120000000001000100111100-
01201111000011010001011110--0011001000102000-

0010010000000000000000001101001110-01000-000001113111100001010001---------
0000-0-1111
'Stegodyphus_sp'
1000000100000000001001000001001?10001---20---110211110000-0-00000000000-
00000----000100000021100000101000010120000000001000100111100-
01201111000011010001011110--0011001000102000-
0010010000000000000000001101001110-01000-000001113111100001010001---------
0100-0-1111
'Megadictyna_thilenii'
1000000100000000001001000001001?10001---20---110211110000-0-00000000000-
00000----000100000021100000101000010120000000001000100111100-
01201111000011010001011110--0011001000102000-
0010010000000000000000001101001110-01000-000001113111100001010001---------
0100-0-1111
Nicodamidae2
1000000100000000001001000001001?10001---20---110211110000-0-00000000000-
00000----000100000021100000101000010120000000001000100111100-
01201111000011010001011110--0011001000102000-
0010010000000000000000001101001110-01000-000001113111100001010001---------
0100-0-1111
'Nicodamidae_sp1'
1000000100000000001001000001001?10001---20---110211110000-0-00000000000-
00000----000100000021100000101000010120000000001000100111100-
01201111000011010001011110--0011001000102000-
0010010000000000000000001101001110-01000-000001113111100001010001---------
0100-0-1111
'Mysmenella_sp'
1000000100000000001001000001001?10001---20---110211110000-0-00000000000-
00000----000100000021100000101000010120000000001000100111100-
01201111000011010001011110--0011001000102000-
0010010000000000000000001101001110-01000-000001113111100001010001---------
0100-0-1111
'Novanapis_sp'
1000000100000000001001000001001?10001---20---110211110000-0-00000000000-
00000----000100000021100000101000010120000000001000100111100-
01201111000011010001011110--0011001000102000-
0010010000000000000000001101001110-01000-000001113111100001010001---------
0100-0-1111
'Anelosimus_clean'
1000000100000000001001000001001?10001---20---110211110000-0-00000000000-
00000----000100000021100000101000010120000000001000100111100-
01201111000011010001011110--0011001000102000-
0010010000000000000000001101001110-01000-000001113111100001010001---------
0100-0-1111

Latrodectus
1000000100000000001001000001001?10001---20---110211110000-0-00000000000-
00000----00010000002110000010100001012000000001000100111100-
01201111000011010001011110--0011001000102000-
0010010000000000000000001101001110-01000-000001113111100001010001---------
0100-0-1111
'Euryopis_sp'
1000000100000000001001000001001?10001---20---110211110000-0-00000000000-
00000----00010000002110000010100001012000000001000100111100-
01201111000011010001011110--0011001000102000-
0010010000000000000000001101001110-01000-000001113111100001010001---------
0100-0-1111
'Parasteatoda_tepidariorum'
1000000100000000001001000001001?10001---20---110211110000-0-00000000000-
00000----00010000002110000010100001012000000001000100111100-
01201111000011010001011110--0011001000102000-
0010010000000000000000001101001110-01000-000001113111100001010001---------
0100-0-1111
'Pararchaea_alba'
1000000100000000001001000001001?10001---20---110211110000-0-00000000000-
00000----00010000002110000010100001012000000001000100111100-
01201111000011010001011110--0011001000102000-
0010010000000000000000001101001110-01000-000001113111100001010001---------
0100-0-1111
'Malkara_clean'
1000000100000000001001000001001?10001---20---110211110000-0-00000000000-
00000----00010000002110000010100001012000000001000100111100-
01201111000011010001011110--0011001000102000-
0010010000000000000000001101001110-01000-000001113111100001010001---------
0100-0-1111
'Malkaridae_GH09'
1000000100000000001001000001001?10001---20---110211110000-0-00000000000-
00000----00010000002110000010100001012000000001000100111100-
01201111000011010001011110--0011001000102000-
0010010000000000000000001101001110-01000-000001113111100001010001---------
0100-0-1111
'Perissopmeros_sp'
1000000100000000001001000001001?10001---20---110211110000-0-00000000000-
00000----00010000002110000010100001012000000001000100111100-
01201111000011010001011110--0011001000102000-
0010010000000000000000001101001110-01000-000001113111100001010001---------
0100-0-1111
'Australomimetus_clean'
1000000100000000001001000001001?10001---20---110211110000-0-00000000000-

00000----000100000021100000101000010120000000001000100111100-
01201111000011010001011110--0011001000102000-
0010010000000000000000001101001110-01000-000001113111100001010001---------
0100-0-1111
'Ero_leonina'
100000010000000001001000001001?10001---20---110211110000-0-00000000000-
00000----000100000021100000101000010120000000001000100111100-
01201111000011010001011110--0011001000102000-
0010010000000000000000001101001110-01000-000001113111100001010001---------
0100-0-1111
'Arkys_sp' 100000010000000001001000001001?10001---20---110211110000-0-00000000000-00000----
000100000021100000101000010120000000001000100111100-
01201111000011010001011110--0011001000102000-
0010010000000000000000001101001110-01000-000001113111100001010001---------
0100-0-1111
Demadiana
100000010000000001001000001001?10001---20---110211110000-0-00000000000-
00000----000100000021100000101000010120000000001000100111100-
01201111000011010001011110--0011001000102000-
0010010000000000000000001101001110-01000-000001113111100001010001---------
0100-0-1111
Leucage
100000010000000001001000001001?10001---20---110211110000-0-00000000000-
00000----000100000021100000101000010120000000001000100111100-
01201111000011010001011110--0011001000102000-
0010010000000000000000001101001110-01000-000001113111100001010001---------
0100-0-1111
'Meta_ovalis_good'
100000010000000001001000001001?10001---20---110211110000-0-00000000000-
00000----000100000021100000101000010120000000001000100111100-
01201111000011010001011110--0011001000102000-
0010010000000000000000001101001110-01000-000001113111100001010001---------
0100-0-1111
'Nanometa_sp'
100000010000000001001000001001?10001---20---110211110000-0-00000000000-
00000----000100000021100000101000010120000000001000100111100-
01201111000011010001011110--0011001000102000-
0010010000000000000000001101001110-01000-000001113111100001010001---------
0100-0-1111
'Tetragnatha_tantalus'
100000010000000001001000001001?10001---20---110211110000-0-00000000000-
00000----000100000021100000101000010120000000001000100111100-
01201111000011010001011110--0011001000102000-

0010010000000000000000001101001110-01000-000001113111100001010001---------
0100-0-1111
'Cepheia_clean'
100000010000000000100100000100 1?10001---20---110211110000-0-00000000000-
00000----00010000002110000010100001012000000000100010011 1100-
01201111000011010001011110--0011001000102000-
0010010000000000000000001101001110-01000-000001113111100001010001---------
0100-0-1111
'Mysmena_leichardti'
1000000100000000001001000001001?10001---20---110211110000-0-00000000000-
00000----000100000021100000101000010120000000001000100111100-
01201111000011010001011110--0011001000102000-
0010010000000000000000001101001110-01000-000001113111100001010001---------
0100-0-1111
'Microdipoena_guttata'
1000000100000000001001000001001?10001---20---110211110000-0-00000000000-
00000----000100000021100000101000010120000000001000100111100-
01201111000011010001011110--0011001000102000-
0010010000000000000000001101001110-01000-000001113111100001010001---------
0100-0-1111
'Spinanapis_sp'
1000000100000000001001000001001?10001---20---110211110000-0-00000000000-
00000----000100000021100000101000010120000000001000100111100-
01201111000011010001011110--0011001000102000-
0010010000000000000000001101001110-01000-000001113111100001010001---------
0100-0-1111
'Tekelloides_sp2'
1000000100000000001001000001001?10001---20---110211110000-0-00000000000-
00000----000100000021100000101000010120000000001000100111100-
01201111000011010001011110--0011001000102000-
0010010000000000000000001101001110-01000-000001113111100001010001---------
0100-0-1111
'Laminacauda_clean'
1000000100000000001001000001001?10001---20---110211110000-0-00000000000-
00000----000100000021100000101000010120000000001000100111100-
01201111000011010001011110--0011001000102000-
0010010000000000000000001101001110-01000-000001113111100001010001---------
0100-0-1111
Frontinella 1000000100000000001001000001001?10001-
--20---110211110000-0-00000000000-00000----
000100000021100000101000010120000000001000100111100-
01201111000011010001011110--0011001000102000-
0010010000000000000000001101001110-01000-000001113111100001010001---------
0100-0-1111

'Novafroneta_sp'
1000000100000000001001000001001?10001---20---110211110000-0-00000000000-
00000----00010000002110000010100001012000000001000100111100-
0120111100001101000101111O--0011001000102000-
001001000000000000000001101001110-01000-0000011131111000010100001---------
0100-0-1111
'Synotaxus_sp'
1000000100000000001001000001001?10001---20---110211110000-0-00000000000-
00000----00010000002110000010100001012000000001000100111100-
0120111100001101000101111O--0011001000102000-
001001000000000000000001101001110-01000-0000011131111000010100001---------
0100-0-1111
'Nesticus_bishopi'
1000000100000000001001000001001?10001---20---110211110000-0-00000000000-
00000----00010000002110000010100001012000000001000100111100-
0120111100001101000101111O--0011001000102000-
001001000000000000000001101001110-01000-0000011131111000010100001---------
0100-0-1111
'Nesticus_cooperi'
1000000100000000001001000001001?10001---20---110211110000-0-00000000000-
00000----00010000002110000010100001012000000001000100111100-
0120111100001101000101111O--0011001000102000-
001001000000000000000001101001110-01000-0000011131111000010100001---------
0100-0-1111
'Meringa_sp'
1000000100000000001001000001001?10001---20---110211110000-0-00000000000-
00000----00010000002110000010100001012000000001000100111100-
0120111100001101000101111O--0011001000102000-
001001000000000000000001101001110-01000-0000011131111000010100001---------
0100-0-1111
'Runga_sp'
1000000100000000001001000001001?10001---20---110211110000-0-00000000000-
00000----00010000002110000010100001012000000001000100111100-
0120111100001101000101111O--0011001000102000-
001001000000000000000001101001110-01000-0000011131111000010100001---------
0100-0-1111
'Physoglenes_clean'
1000000100000000001001000001001?10001---20---110211110000-0-00000000000-
00000----00010000002110000010100001012000000001000100111100-
0120111100001101000101111O--0011001000102000-
001001000000000000000001101001110-01000-0000011131111000010100001---------
0100-0-1111
'Physoglenidae_clean'
1000000100000000001001000001001?10001---20---110211110000-0-00000000000-

00000----00010000002110000010100001012000000001000100111100-
01201111000011010001011110--0011001000102000-
00100100000000000000001101001110-01000-000001113111100001010001---------
0100-0-1111
'Ogulnius_sp'
1000000100000000001001000001001?10001---20---110211110000-0-00000000000-
00000----00010000002110000010100001012000000001000100111100-
01201111000011010001011110--0011001000102000-
00100100000000000000001101001110-01000-000001113111100001010001---------
0100-0-1111
'Baalzebub_sp'
1000000100000000001001000001001?10001---20---110211110000-0-00000000000-
00000----00010000002110000010100001012000000001000100111100-
01201111000011010001011110--0011001000102000-
00100100000000000000001101001110-01000-000001113111100001010001---------
0100-0-1111
'Theridiosoma_gemmosum'
1000000100000000001001000001001?10001---20---110211110000-0-00000000000-
00000----00010000002110000010100001012000000001000100111100-
01201111000011010001011110--0011001000102000-
00100100000000000000001101001110-01000-000001113111100001010001---------
0100-0-1111
'Theridiosoma_savannum'
1000000100000000001001000001001?10001---20---110211110000-0-00000000000-
00000----00010000002110000010100001012000000001000100111100-
01201111000011010001011110--0011001000102000-
00100100000000000000001101001110-01000-000001113111100001010001---------
0100-0-1111
'Trichonephila_clavipes_Genome'
1000000100000000001001000001001?10001---20---110211110000-0-00000000000-
00000----00010000002110000010100001012000000001000100111100-
01201111000011010001011110--0011001000102000-
00100100000000000000001101001110-01000-000001113111100001010001---------
0100-0-1111
'Micrathena_gracilis'
1000000100000000001001000001001?10001---20---110211110000-0-00000000000-
00000----00010000002110000010100001012000000001000100111100-
01201111000011010001011110--0011001000102000-
00100100000000000000001101001110-01000-000001113111100001010001---------
0100-0-1111
'Verrucosa_arenata'
1000000100000000001001000001001?10001---20---110211110000-0-00000000000-
00000----00010000002110000010100001012000000001000100111100-
01201111000011010001011110--0011001000102000-

0010010000000000000000001101001110-01000-000001113111100001010001---------
0100-0-1111

'Cyrtophora_sp'

1000000100000000001001000001001?10001---20---110211110000-0-00000000000-
00000----00010000002110000010100001012000000001000100111100-
01201111000011010001011110--0011001000102000-
0010010000000000000000001101001110-01000-000001113111100001010001---------
0100-0-1111

Neoscona

1000000100000000001001000001001?10001---20---110211110000-0-00000000000-
00000----00010000002110000010100001012000000001000100111100-
01201111000011010001011110--0011001000102000-
0010010000000000000000001101001110-01000-000001113111100001010001---------
0100-0-1111

'Gasteracantha_hasselti'

1000000100000000001001000001001?10001---20---110211110000-0-00000000000-
00000----00010000002110000010100001012000000001000100111100-
01201111000011010001011110--0011001000102000-
0010010000000000000000001101001110-01000-000001113111100001010001---------
0100-0-1111

'Macracantha_arcuata'

1000000100000000001001000001001?10001---20---110211110000-0-00000000000-
00000----00010000002110000010100001012000000001000100111100-
01201111000011010001011110--0011001000102000-
0010010000000000000000001101001110-01000-000001113111100001010001---------
0100-0-1111

'Menneus_sp'

1000000100000000001001000001001?10001---20---110211110000-0-00000000000-
00000----00010000002110000010100001012000000001000100111100-
01201111000011010001011110--0011001000102000-
0010010000000000000000001101001110-01000-000001113111100001010001---------
0100-0-1111

'Deinopis_clean'

1000000100000000001001000001001?10001---20---110211110000-0-00000000000-
00000----00010000002110000010100001012000000001000100111100-
01201111000011010001011110--0011001000102000-
0010010000000000000000001101001110-01000-000001113111100001010001---------
0100-0-1111

'Deinopis_longipes'

1000000100000000001001000001001?10001---20---110211110000-0-00000000000-
00000----00010000002110000010100001012000000001000100111100-
01201111000011010001011110--0011001000102000-
0010010000000000000000001101001110-01000-000001113111100001010001---------
0100-0-1111

'Tamopsis_sp'
100000010000000001001000001001?10001---20---110211110000-0-00000000000-00000----00010000002110000010100001012000000001000100111100-012011110000110100010111110--0011001000102000-0010010000000000000000001101001110-01000-00000111311110000101000 1---------0100-0-1111
'Oecobius_navus'
100000010000000001001000001001?10001---20---110211110000-0-00000000000-00000----00010000002110000010100001012000000001000100111100-012011110000110100010111110--0011001000102000-0010010000000000000000001101001110-01000-000001113111100001010001---------0100-0-1111
'Oecobius_sp'
100000010000000001001000001001?10001---20---110211110000-0-00000000000-00000----00010000002110000010100001012000000001000100111100-012011110000110100010111110--0011001000102000-0010010000000000000000001101001110-01000-000001113111100001010001---------0100-0-1111
'Zosis_sp' 100000010000000001001000001001?10001---20---110211100000-0-00000000000-00000----00010000002110000010100001012000000001000100111100-012011110000110100010111110--0011001000102000-0010010000000000000000001101001110-01000-000001113111100001010001---------0100-0-1111
'Philoponella_herediae'
100000010000000001001000001001?10001---20---110211100000-0-00000000000-00000----00010000002110000010100001012000000001000100111100-012011110000110100010111110--0011001000102000-0010010000000000000000001101001110-01000-000001113111100001010001---------0100-0-1111
'Uloborus_glomosus'
100000010000000001001000001001?10001---20---110211100000-0-00000000000-00000----00010000002110000010100001012000000001000100111100-012011110000110100010111110--0011001000102000-0010010000000000000000001101001110-01000-000001113111100001010001---------0100-0-1111
'Cybaeodamus_taim'
100000010000000001001000001001?10001---20---110211110000-0-00000000000-00000----00010000002110000010100001012000000001000100111100-012011110000110100010111110--0011001000102000-0010010000000000000000001101001110-01000-000001113111100001010001---------0100-0-1111
'Forsterella_sp'
100000010000000001001000001001?10001---20---110211110000-0-00000000000-

00000----00010000002110000010100001012000000001000100111100-
01201111000011010001011110--0011001000102000-
001001000000000000000001101001110-01000-000001113111100001010001---------
0100-0-1111
'Caayguara_clean'
100000010000000001001000001001?10001---20---110211110000-0-00000000000-
00000----00010000002110000010100001012000000001000100111100-
01201111000011010001011110--0011001000102000-
001001000000000000000001101001110-01000-000001113111100001010001---------
0100-0-1111
Amaurobius
100000010000000001001000001001?10001---20---110211110000-0-00000000000-
00000----00010000002110000010100001012000000001000100111100-
01201111000011010001011110--0011001000102000-
001001000000000000000001101001110-01000-000001113111100001010001---------
0100-0-1111
'Callobius_sp'
100000010000000001001000001001?10001---20---110211110000-0-00000000000-
00000----00010000002110000010100001012000000001000100111100-
01201111000011010001011110--0011001000102000-
001001000000000000000001101001110-01000-000001113111100001010001---------
0100-0-1111
'Neoramia_sp'
100000010000000001001000001001?10001---20---110211110000-0-00000000000-
00000----00010000002110000010100001012000000001000100111100-
01201111000011010001011110--0011001000102000-
001001000000000000000001101001110-01000-000001113111100001010001---------
0100-0-1111
Amphinecta
100000010000000001001000001001?10001---20---110211110000-0-00000000000-
00000----00010000002110000010100001012000000001000100111100-
01201111000011010001011110--0011001000102000-
001001000000000000000001101001110-01000-000001113111100001010001---------
0100-0-1111
'Metaltella_simoni'
100000010000000001001000001001?10001---20---110211110000-0-00000000000-
00000----00010000002110000010100001012000000001000100111100-
01201111000011010001011110--0011001000102000-
001001000000000000000001101001110-01000-000001113111100001010001---------
0100-0-1111
Cambridgea
100000010000000001001000001001?10001---20---110211110000-0-00000000000-
00000----00010000002110000010100001012000000001000100111100-
01201111000011010001011110--0011001000102000-

001001000000000000000001101001110-01000-000001113111100001010001---------
0100-0-1111

'Paramatachia_sp'
1000000100000000001001000001001?10001---20---110211110000-0-00000000000-
00000----0001000000211000001010000101200000000100010011110 0-
012011110000110100010111 10--0011001000102000-
001001000000000000000001101001110-01000-000001113111100001010001---------
0100-0-1111

'Agelenopsis_emertoni_cdhitest.fasta.transdecoder'1000000100000000001001000001001?10001---20---110211110000-0-00000000000-00000----
0001000000211000001010000101200000000100010011110 0-
012011110000110100010111 10--0011001000102000-
001001000000000000000001101001110-01000-000001113111100001010001---------
0100-0-1111

'Agelenopsis_pennsylvanica'
1000000100000000001001000001001?10001---20---110211110000-0-00000000000-
00000----0001000000211000001010000101200000000100010011110 0-
012011110000110100010111 10--0011001000102000-
001001000000000000000001101001110-01000-000001113111100001010001---------
0100-0-1111

'Calymmaria_clean'
1000000100000000001001000001001?10001---20---110211110000-0-00000000000-
00000----0001000000211000001010000101200000000100010011110 0-
012011110000110100010111 10--0011001000102000-
001001000000000000000001101001110-01000-000001113111100001010001---------
0100-0-1111

'Calymmaria_persica'
1000000100000000001001000001001?10001---20---110211110000-0-00000000000-
00000----0001000000211000001010000101200000000100010011110 0-
012011110000110100010111 10--0011001000102000-
001001000000000000000001101001110-01000-000001113111100001010001---------
0100-0-1111

'Cicurina_travisae'
1000000100000000001001000001001?10001---20---110211110000-0-00000000000-
00000----0001000000211000001010000101200000000100010011110 0-
012011110000110100010111 10--0011001000102000-
001001000000000000000001101001110-01000-000001113111100001010001---------
0100-0-1111

'Cicurina_vobora'
1000000100000000001001000001001?10001---20---110211110000-0-00000000000-
00000----0001000000211000001010000101200000000100010011110 0-
012011110000110100010111 10--0011001000102000-
001001000000000000000001101001110-01000-000001113111100001010001---------
0100-0-1111

'Homalonychus_theologus'
1000000100000000001001000001001?10001---20---110211110000-0-00000000000-
00000----00010000002110000010100001012000000001000100111100-
01201111000011010001011110--0011001000102000-
0010010000000000000000001101001110-01000-000001113111100001010001---------
0100-0-1111
'Tengella_sp'
1000000100000000001001000001001?10001---20---110211110000-0-00000000000-
00000----00010000002110000010100001012000000001000100111100-
01201111000011010001011110--0011001000102000-
0010010000000000000000001101001110-01000-000001113111100001010001---------
0100-0-1111
'Uliodon_sp'
1000000100000000001001000001001?10001---20---110211110000-0-00000000000-
00000----00010000002110000010100001012000000001000100111100-
01201111000011010001011110--0011001000102000-
0010010000000000000000001101001110-01000-000001113111100001010001---------
0100-0-1111
'Misumenoides_formosipes'
1000000100000000001001000001001?10001---20---110211110000-0-00000000000-
00000----00010000002110000010100001012000000001000100111100-
01201111000011010001011110--0011001000102000-
0010010000000000000000001101001110-01000-000001113111100001010001---------
0100-0-1111
'Sidymella_sp'
1000000100000000001001000001001?10001---20---110211110000-0-00000000000-
00000----00010000002110000010100001012000000001000100111100-
01201111000011010001011110--0011001000102000-
0010010000000000000000001101001110-01000-000001113111100001010001---------
0100-0-1111
'Anahita_punctulata'
1000000100000000001001000001001?10001---20---110211110000-0-00000000000-
00000----00010000002110000010100001012000000001000100111100-
01201111000011010001011110--0011001000102000-
0010010000000000000000001101001110-01000-000001113111100001010001---------
0100-0-1111
'Peucetia_longipalpis'
1000000100000000001001000001001?10001---20---110211110000-0-00000000000-
00000----00010000002110000010100001012000000001000100111100-
01201111000011010001011110--0011001000102000-
0010010000000000000000001101001110-01000-000001113111100001010001---------
0100-0-1111
'Dolomedes_trinity'
1000000100000000001001000001001?10001---20---110211110000-0-00000000000-

00000----0001000000211000001010000101200000000100010011100-
0120111100001101000101111O--0011001000102000-
0010010000000000000000110100111O-01000-0000011131111000010100O1---------
0100-0-1111
'Pisaurina_mira'
1000000100000000001001000001001?10001---20---110211110000-0-00000000000-
00000----0001000000211000001010000101200000000100010011100-
012011110000110100010111110--0011001000102000-
00100100000000000000001101001110-01000-00000111311110000101000 1---------
0100-0-1111
'Cupiennius_sp'
1000000100000000001001000001001?10001---20---110211110000-0-00000000000-
00000----0001000000211000001010000101200000000100010011100-
012011110000110100010111110--0011001000102000-
00100100000000000000001101001110-01000-0000011131111000010100O1---------
0100-0-1111
Allocosa 1000000100000000001001000001001?10001-
--20---110211110000-0-00000000000-00000----
0001000000211000001010000101200000000100010011100-
012011110000110100010111110--0011001000102000-
00100100000000000000001101001110-01000-000001113111100001010001---------
0100-0-1111
'Schizocosa_rovneri'
1000000100000000001001000001001?10001---20---110211110000-0-00000000000-
00000----0001000000211000001010000101200000000100010011100-
012011110000110100010111110--0011001000102000-
00100100000000000000001101001110-01000-000001113111100001010001---------
0100-0-1111
'Habronattus_signatus'
1000000100000000001001000001001?10001---20---110211110000-0-00000000000-
00000----0001000000211000001010000101200000000100010011100-
012011110000110100010111110--0011001000102000-
00100100000000000000001101001110-01000-000001113111100001010001---------
0100-0-1111
'Habronattus_ustulatus'
1000000100000000001001000001001?10001---20---110211110000-0-00000000000-
00000----0001000000211000001010000101200000000100010011100-
012011110000110100010111110--0011001000102000-
00100100000000000000001101001110-01000-000001113111100001010001---------
0100-0-1111
'Teminius_sp'
1000000100000000001001000001001?10001---20---110211110000-0-00000000000-
00000----0001000000211000001010000101200000000100010011100-
012011110000110100010111110--0011001000102000-

0010010000000000000000001101001110-01000-000001113111100001010001---------
0100-0-1111
'Karaops_raveni'
1000000100000000010010000001001?10001---20---110211110000-0-00000000000-
00000----000100000021100000101000010120000000001000100111100-
01201111000011010001011110--0011001000102000-
0010010000000000000000001101001110-01000-000001113111100001010001---------
0100-0-1111
'Falconina_gracilis'
1000000100000000010010000001001?10001---20---110211110000-0-00000000000-
00000----000100000021100000101000010120000000001000100111100-
01201111000011010001011110--0011001000102000-
0010010000000000000000001101001110-01000-000001113111100001010001---------
0100-0-1111
'Nyssus_sp'
1000000100000000010010000001001?10001---20---110211110000-0-00000000000-
00000----000100000021100000101000010120000000001000100111100-
01201111000011010001011110--0011001000102000-
0010010000000000000000001101001110-01000-000001113111100001010001---------
0100-0-1111
'Clubiona_clean'
1000000100000000010010000001001?10001---20---110211110000-0-00000000000-
00000----000100000021100000101000010120000000001000100111100-
01201111000011010001011110--0011001000102000-
0010010000000000000000001101001110-01000-000001113111100001010001---------
0100-0-1111
'Rebilus_sp'
1000000100000000010010000001001?10001---20---110211110000-0-00000000000-
00000----000100000021100000101000010120000000001000100111100-
01201111000011010001011110--0011001000102000-
0010010000000000000000001101001110-01000-000001113111100001010001---------
0100-0-1111
'Liocranidae_GOOD'
1000000100000000010010000001001?10001---20---110211110000-0-00000000000-
00000----000100000021100000101000010120000000001000100111100-
01201111000011010001011110--0011001000102000-
0010010000000000000000001101001110-01000-000001113111100001010001---------
0100-0-1111
'Trachelas_tranquillus'
1000000100000000010010000001001?10001---20---110211110000-0-00000000000-
00000----000100000021100000101000010120000000001000100111100-
01201111000011010001011110--0011001000102000-
0010010000000000000000001101001110-01000-000001113111100001010001---------
0100-0-1111

'Sergiolus_capulatus'
10000001000000000100100000 1001?10001---20---110211110000-0-00000000000-
00000----000100000021100000101000010120000000001000100111100-
0120111100001101000101111 0--0011001000102000-
001001000000000000000001101001110-01000-000001113111100001010001---------
0100-0-1111
'Molycria_sp'
10000001000000000100100000 1001?10001---20---110211110000-0-00000000000-
00000----000100000021100000101000010120000000001000100111100-
0120111100001101000101111 0--0011001000102000-
001001000000000000000001101001110-01000-000001113111100001010001---------
0100-0-1111
Anzacia 10000001000000000100100000 1001?10001-
--20---110211110000-0-00000000000-00000----
000100000021100000101000010120000000001000100111100-
0120111100001101000101111 0--0011001000102000-
001001000000000000000001101001110-01000-000001113111100001010001---------
0100-0-1111
'Lampona_sp'
10000001000000000100100000 1001?10001---20---110211110000-0-00000000000-
00000----000100000021100000101000010120000000001000100111100-
0120111100001101000101111 0--0011001000102000-
001001000000000000000001101001110-01000-000001113111100001010001---------
0100-0-1111
Goniotarbus 1000?0011?000000???????????0???00-
000--?????????????????-
???00?0??????????????????010???002?1???00?0100???040010?010???0010-
???0???113?1?????00?????021------
?????????????????????????????????????????????????????0000??????????????
????????????---------0?---??????
Bornatarbus 1000?0011?000000???????????0???00-
000--?????????????????-
???00?0??????????????????010???002?1???00?0100???040010?000???0010-
???0???113?1?????00?????021------
?????????????????????????????????????????????????????0000??????????????
????????????---------0?---??????

;

END;
